# Cycloruthenated Imines: A Step into the Nanomolar Region

**DOI:** 10.3390/molecules31020315

**Published:** 2026-01-16

**Authors:** Arsenii A. Vasil’ev, Ivan I. Troshin, Pavel G. Shangin, Ksenia M. Voroshilkina, Ilya A. Shutkov, Alexey A. Nazarov, Aleksei V. Medved’ko

**Affiliations:** 1 N.D. Zelinsky Institute of Organic Chemistry RAS, 47 Leninsky Prospect, 119991 Moscow, Russia; 2 Chemistry Department, M.V. Lomonosov Moscow State University, Leninskie Gory 1-3, 119234 Moscow, Russia; 3 Department of Chemistry and Technology of Biologically Active Compounds, Medicinal and Organic Chemistry, Institute of Fine Chemical Technologies, MIREA-Russian Technological University, 86 Vernadsky Avenue, 119571 Moscow, Russia

**Keywords:** cycloruthenated complexes, imines, benzene, thiophene, cytotoxicity

## Abstract

A new series of promising and easily accessible antiproliferative agents based on cycloruthenated imines of benzene and thiophene carbaldehydes has been developed and fully characterized using UV-Vis spectroscopy, X-ray diffraction, NMR, HRMS, and cyclic voltammetry. The biological activity of these compounds was tested against A2780, cisplatin-resistant A2780, and HEK293 cell lines, and they exhibited nanomolar IC_50_ values. They also showed a selectivity index of up to 2.5, indicating their potential as promising antiproliferative compounds.

## 1. Introduction

Despite global efforts to treat cancer, global mortality rates and the number of new cases of cancer continue to rise steadily. Malignant tumors remain a key factor limiting life expectancy. Chemotherapy is a traditional and widely used method of cancer treatment. Among the most popular anticancer drugs are platinum compounds: cisplatin, carboplatin and others. They have proven their effectiveness; however, despite their advantages, they can cause serious side effects, including hepatotoxicity, neurotoxicity, ototoxicity and others [1,2]. Therefore, the scientific community is faced with the task of creating analogs of platinum compounds devoid of the above-mentioned disadvantages. Other transition metals, such as copper, iridium, osmium, gold and ruthenium compounds, are actively studied as anticancer agents [3,4]. Ruthenium is the most promising in this series, since it has broad coordination capabilities, unique mechanisms of anticancer activity in living cells, and its compounds are more accessible for commercial use than iridium or platinum precursors. Several families of anticancer drugs have already been developed based on ruthenium, including multilayer complexes such as NAMI [5] and RAPTA [6] type complexes. Photosensitizers based on ruthenium complexes can also be used for photodynamic cancer therapy [7,8]. Many ruthenium cyclometallated compounds containing a metal–carbon bond have been described in the literature. They are often used as dyes for DSSC [9], as well as oxidation [10] and cross-coupling [11] catalysts. At the same time, examples of the use of cycloruthenated complexes as anticancer drugs are also found in the literature [12,13,14,15,16,17,18,19,20,21]. In most cases, such compounds consist of an aryl moiety directly linked to the ruthenium atom via a Ru-C bond and one or more heteroaryl rings that are linked to the aryl moiety. The donor nitrogen atom is usually located inside the heteroaryl ring [22]. Several years ago, we published a paper in which we synthesized ruthenium cyclometallated compounds containing a thiophene moiety [23] to study them as dyes for DSSCs. One of these compounds was an imine based on thiophene-2-carbaldehyde. Compared to the typical complexes described above, imines are much easier to synthesize, and the variability of their structure, due to the availability of a wide range of aldehydes and anilines, is much higher than for traditional cyclometallated compounds. A preliminary study of their anticancer properties showed that they are an order of magnitude more cytotoxic than the model drug cisplatin [24]. Interestingly, the IC_50_ values for typical ruthenium complexes are in the region of a few μmol/L [12], whereas the resulting imine complexes showed activities at the level of tenths of μmol/L. To our surprise, there were extremely few examples of cycloruthenated imines in the literature, even for the simplest imine obtained from benzaldehyde and aniline. Not to mention the fact that the biological activity of these derivatives has not been studied at all. The goal of this work was to expand the scaffold of cycloruthenated imines not only to the thiophene series, but also to the previously unstudied benzaldehyde series. Furthermore, to demonstrate the potential influence of imine structure on the biological properties of the complexes, complexes with various substituents on the aniline ring were prepared.

## 2. Results and Discussion

### 2.1. Synthesis

**Scheme 1:**
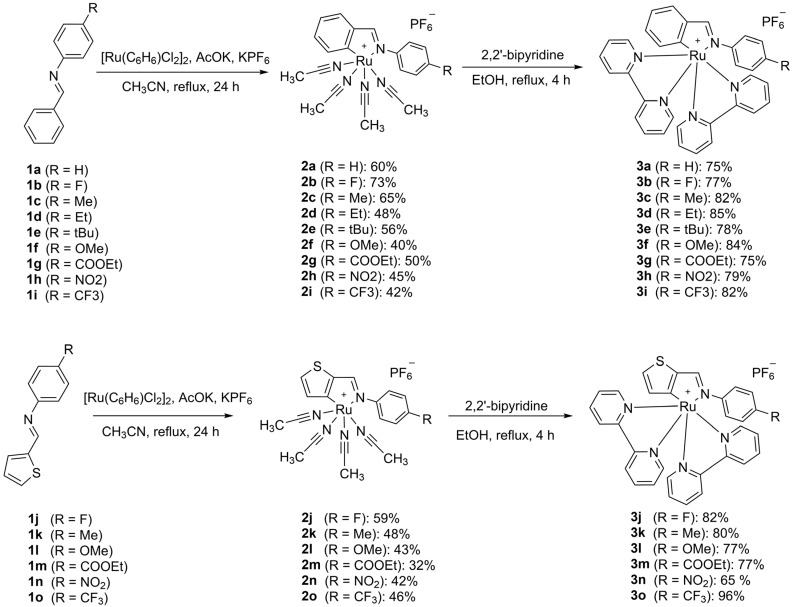
Preparation of ruthenium complexes.

All intermediate compounds and target complexes were obtained using methods similar to those described previously [24] (Scheme 1). Cycloruthenated complexes **3a**–**o** were synthesized with moderate to good yields by a two-step procedure by cyclometallation of imines and chelation of the resulting acetonitrile complexes **2a**–**o**. The remaining coordination sites were occupied by 2,2′-bipyridines to stabilize ruthenium in the (II) oxidation state, while aromatic ligands increase lipophilicity, facilitating transport across biological membranes. It is believed that ruthenium anticancer agents can bind to nuclear DNA via intercalation interactions; therefore, the presence of aromatic ligands enables stacking interactions between the ruthenium ring and nitrogenous base pairs.

### 2.2. Crystallography

**Figure 1:**
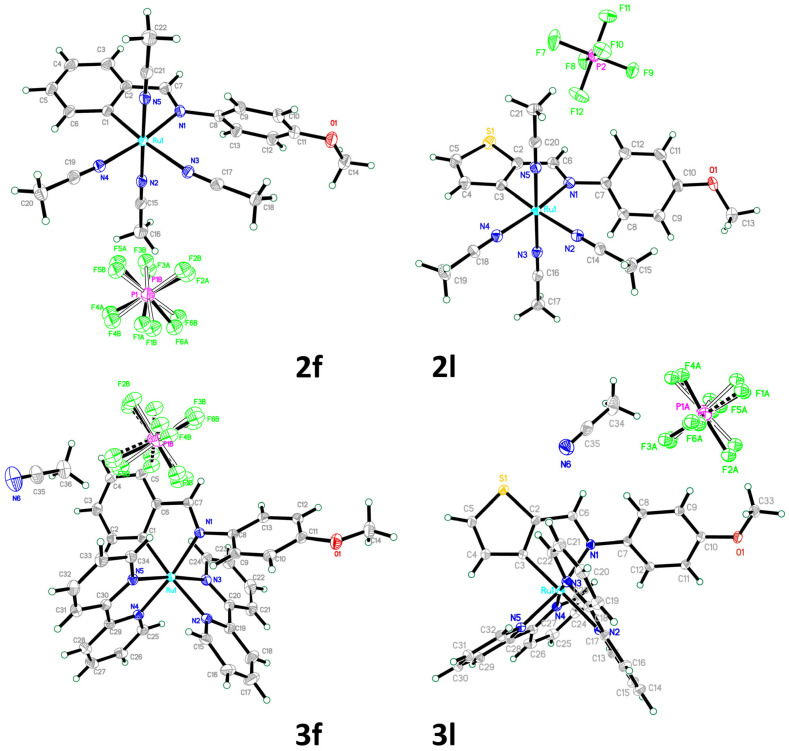
Crystal structures of **2f**, **2l**, **3f·CH_3_CN**, **3l·CH_3_CN**.

From a crystallographic point of view, the obtained complexes have a similar structure (Table S1, Figure 1 and Figures S31–S53). Thus, the lengths of the Ru–C bonds are in the range of 2.008–2.032 Å, Ru–N(aniline)—2.062–2.123 Å, Ru-N, located opposite the Ru–C bond—2.132–2.172 Å (Table 1). The plane of the aniline ring is inclined relative to the plane of the five-membered ring with the ruthenium atom with the dihedral angle 40.65–65.66°. The sign of this angle is not characteristic, either for acetonitrile, or for bipyridine complexes. It is worth noting that the formation of the bipyridine complex decreases the modulus of the dihedral angle by an average of 10° (cf. **2a** and **3a**, **2b** and **3b**, **2e** and **3e**, **2f** and **3f**). In bipyridine complexes, one fragment is more distorted than the other. The dihedral angle between the two pyridine rings ranges from 1.85° to 10.77°. The dihedral angle of the second fragment is approximately 1°.

### 2.3. UV-Vis Examination

**Figure 2:**
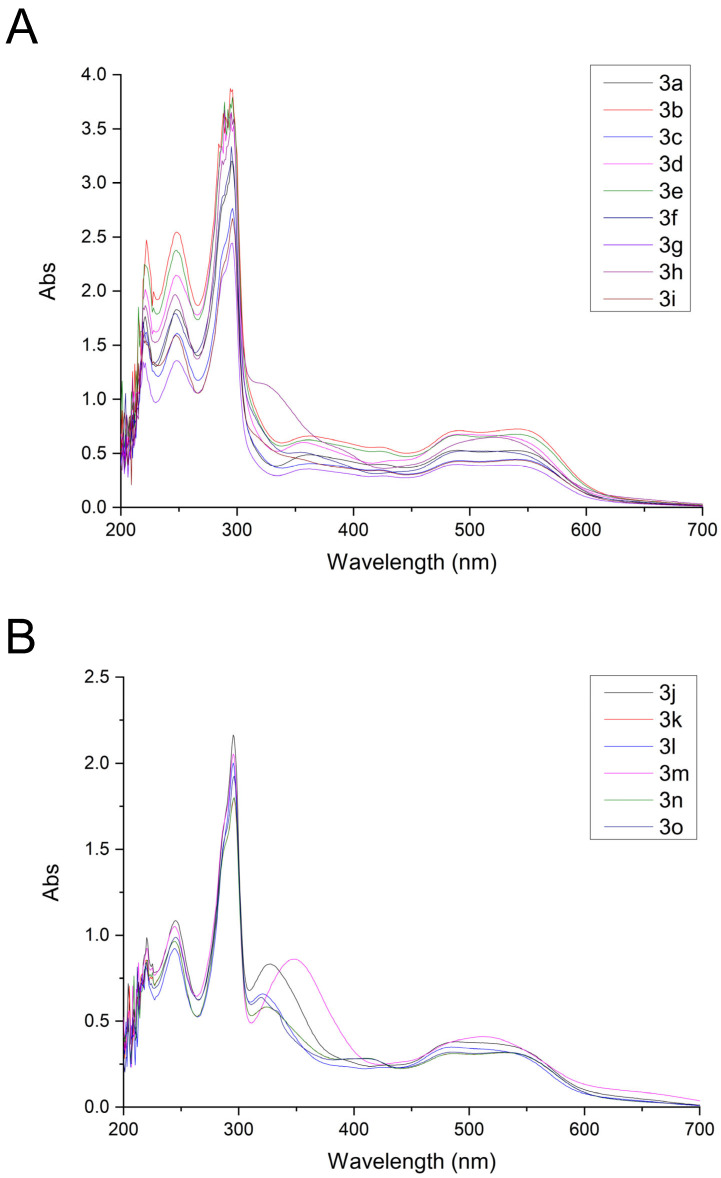
UV-vis spectra of **3a**–**3i** (**A**) and **3j**–**3o** (**B**) in acetonitrile, concentration 3.4 × 10^−5^ M.

UV spectra were recorded for complexes **3a**–**3o** in acetonitrile (Figure 2) to determine their optical properties. All absorption spectra in acetonitrile exhibit two intensity bands: 250–300 nm (corresponding to the absorption bands of aromatic systems) and 450–600 nm (arising after metal coordination with 2,2′-bipyridine) which are typical for cycloruthenated compounds [23]. This can be visually observed: during the reaction, acetonitriles are replaced by bipyridines, and the compounds change from orange to dark purple.

Stability of **3a**–**o** was also assessed in 0.5% DMSO solution in aqueous phosphate buffer (pH 7.4, 37 °C, 100 μM NaCl, 77.4 μM Na_2_HPO_4_, 22.6 μM NaH_2_PO_4_) or saline by comparing the UV spectrum of solutions of complexes **3a**–**o**, recorded immediately after solution preparation, and the UV spectrum of the same solution after 18 h of incubation at room temperature (Figures S69 and S70). The spectra were compatible for all the complexes, indicating that they were sufficiently stable. A slight change in the intensity of signals is due to the slow degradation of ruthenium complexes with the formation of aqua- and oxo-complexes, as well as partial precipitation, which is typical for ruthenium compounds [25].

### 2.4. Electrochemistry

The E^1/2^_ox1_ potentials of benzaldehyde-based complexes are 8–22 mV higher than those of thiophenecarbaldehyde-based complexes. At the same time, compared to the previously described imine complex [23] but with carboxyl groups, the E^1/2^_ox1_ potentials of **3i**–**3m**, **3o** are somewhat lower (77–127 mV versus 150 mV [23]), with the exception of the complex with the nitro group **3n**, which has a potential of 161 mV. However, the most common values for cycloruthenated phenylpyridines [26] and phenylimidazoles [27] are in the range of 50–250 mV, so the resulting complexes fit well within this range. The introduction of electron-withdrawing substituents increases the E^1/2^_ox1_ value of the Ru^2+^/Ru^3+^ transition by an average of 40–60 mV (Table 2, Figures S52–S66). The electron-donating methoxy group (**3f** and **3l**) weakly reduces this potential. When comparing the nitro group in the thiophene ring [24] with the nitro group in the aniline ring, the latter shifts the oxidation potential less strongly (257 mV vs. 215 mV). Overall, the oxidation potentials for the phenyl and thiophene derivatives are close to each other, except for the ethoxycarbonyl derivatives **3g** and **3m**, for which the potential differs by almost a factor of two.

### 2.5. Biological Study

The in vitro antiproliferative activity of the synthesized Ru-organometallic compounds and several starting ligands was evaluated against three human cell lines using the MTT assay. We selected a small panel of cell lines that provided insights into the efficacy and potential therapeutic utility of the compounds. The panel includes the A2780 human ovarian carcinoma cell line, which is sensitive to cisplatin, its cisplatin-resistant counterpart, A2780cis, also a non-malignant human embryonic kidney cell line, HEK293. The use of A2780 and A2780cis paired cell lines allowed us to directly assess the ability of the compounds to overcome resistance mechanisms. The inclusion of the HEK293 cell line can give us preliminary indication of selectivity against non-cancerous cells. Cisplatin, a clinically used drug, was used as a positive control in all experiments. All studies were performed in triplicate and repeated in three independent experiments. The results, expressed as the half-maximal inhibitory concentration after 72 h incubation (IC_50_), are summarized in Table 3.

Based on the initial analysis of the data, we can conclude that the IC_50_ values for all Ru compounds fall within the medium to high nanomolar range, suggesting a high level of cytotoxicity against both malignant and non-malignant cells. Moreover, the organometallic compounds appear to be several times more cytotoxic than cisplatin, approximately 10 times or more, although their selectivity for cancer cells is only moderate. The selectivity index for these compounds does not exceed 2.5, compared to 8.3 for cisplatin. The data on the Ru^2+^/Ru^3+^ transition potential correlate rather weakly with the cytotoxicity data. It can only be noted that the higher the oxidation potential E^1/2^_ox1_, the higher the IC_50_ value.

The presence of a ruthenium atom in a complex determines its biological activity. The data show that ligands such as **1b**, **1f**, and **1g** do not exhibit any cytotoxicity, but the cytotoxicity of N-benzylideneaniline complexes (**3b**–**i**) and thiophenylimine compounds (**3j**–**o**) against A2780 is virtually identical. On the other hand, thiophenylidenimine ruthenacycles (against A2780cis) are several times more cytotoxic than N-benzylideneaniline complexes.

The cytotoxicity of the obtained complexes toward A2780 cells is several times greater than that of the cycloruthenated complexes described in the literature. For example, for cycloruthenated benzimidazoles, pyrazoles, and phenylpyridines, IC_50_ values range from 130–210 nmol/L [28] and can reach tens and hundreds of μmol/L [29,30]. Interestingly, the selectivity index of the compounds already described is not possible due to the lack of data on their toxicity toward non-cancerous cells.

If we look at the structure of the ligands, we can draw the following conclusions about their activity: the electron-donating OMe group slightly increases cytotoxicity, while the acceptors NO_2_ and CF_3_ reduce cytotoxicity. At the same time, alkyl substituents (except for tBu) increase the cytotoxicity of both the benzylideneaniline and thiophenylidenimine families of complexes. This can be attributed to their increased lipophilicity. So, this makes cycloruthenated imines a promising treatment for cisplatin-resistant tumors.

## 3. Experimental Section

All starting materials were purchased from ABCR (Karlsruhe, Germany) and SigmaAldrich (St. Louis, MO, USA) and used without additional purification. Silica gel for column chromatography (fraction 0.040-0.063 mm) was purchased from Carl Roth (Karlsruhe, Germany). NMR spectra were recorded on Bruker AVANCE 300 and Bruker II 600 spectrometers (Billerica, MA, USA). Residual signals of the solvents served as internal standards. HRMS-ESI spectra were recorded on Bruker MicroTOF II mass spectrometer (Billerica, MA, USA).

**Single crystal X-ray crystallographic data and refinement details.**

X-ray diffraction data for **2a**, **3e**, **3m** and **3o** were collected at 100 K on a Bruker Quest D8 diffractometer (Billerica, MA, USA) equipped with a Photon-III area-detector (graphite monochromator, shutterless φ- and ω-scan technique), using Mo K_α_-radiation. The intensity data were integrated by the SAINT program [31] and were corrected for absorption and decay using SADABS [32]. The structure was solved by direct methods using SHELXT [33] and refined on F^2^ using SHELXL-2018 [34] in the OLEX2 program [35]. All non-hydrogen atoms were refined with individual anisotropic displacement parameters. All hydrogen atoms were placed in ideal calculated positions and refined as riding atoms with relative isotropic displacement parameters. The SHELXTL program suite [31] was used for molecular graphics.

X-ray diffraction data were collected at 100K on a four-circle Rigaku Synergy S diffractometer (Wroclaw, Poland) equipped with a HyPix600HE area-detector (kappa geometry, shutterless ω-scan technique), using monochromatized Cu K_α_-radiation (for compounds **1n**, **2b**, **2d**, **2f**–**2l**, **2n**, **3a**, **3b**, **3f**, **3h**, **3j**) and using monochromatized Mo K_α_-radiation (for compounds **2e**, **3c**). The intensity data were integrated and corrected for absorption and decay by the CrysAlisPro program [36]. The structure was solved by direct methods using SHELXT [33] and refined on F^2^ using SHELXL-2018 [34] in the OLEX2 program [35]. All non-hydrogen atoms were refined with individual anisotropic displacement parameters. All hydrogen atoms were placed in ideal calculated positions and refined as riding atoms with relative isotropic displacement parameters. The SHELXTL program suite [31] was used for molecular graphics.

The structures of **2a**, **2b**, **3c** and **3m** contained unresolved/highly disordered acetonitrile and ether molecules in the crystal channels, which were removed by the SQUEEZE method [37] implemented in the OLEX2 program [35].

**Cyclic voltammetry** **.**

The electrochemical oxidation and reduction behavior of the compounds under discussion was investigated by cyclic voltammetry using an IPC-Pro-MF potentiostat from Econix (Sheffield, UK). The preparation of solutions and all measurements were performed in an argon-filled glovebox at a water and oxygen level of no more than 0.1 ppm. Before use, acetonitrile (HPLC grade, Acros, Waltham, MA, USA) with an initial water content of no more than 100 ppm was stored over molecular sieves (4 Å) pre-dried under oil pump vacuum at 200–250 °C for 4 h. Bu_4_NPF_6_ (Sigma Aldrich, St. Louis, MO, USA) was dried under oil pump vacuum at 80 °C for 4 h. The water content in the 0.1 M Bu_4_NPF_6_/acetonitrile system used did not exceed 20 ppm after that, which was monitored by Karl Fischer titration using a Mettler-Toledo C10SD titrator (Zurich, Switzerland). The compounds dissolved in 5 mL of the background electrolyte were electrochemically analyzed in a standard conical three-electrode glass cell. The working electrode was a 1.7 mm diameter glassy carbon disk electrode placed in PTFE. Before use, it was polished with sandpaper and GOI paste until a mirror shine was achieved. The auxiliary electrode was a platinum wire pre-calcined in a gas burner flame. The potentials of the processes under study were measured relative to a silver wire coated with AgCl (achieved by galvanostatic anodizing in a 5% hydrochloric acid solution) separated from the main solution by an electrochemical bridge filled with an auxiliary electrolyte. Separately, under similar conditions, the oxidation curves of ferrocene were recorded, and the given values were corrected according to the oxidation potential of the latter.

**Cells and MTT assay** **.**

The human A2780 ovarian adenocarcinoma, A2780cis cisplatin resistant ovarian adenocarcinoma, HEK293 human embryonic kidney cell lines were obtained from the European collection of authenticated cell cultures (ECACC; Salisbury, UK). Cells were grown in a RPMI 1640 (Gibco™, Cork, Ireland) cell medium (A2780, A2780cis), or DMEM (Gibco™, Ireland) cell medium (HEK293) supplemented with 10% fetal bovine serum (Gibco™, São Paulo, Brazil). The cells were cultured in an incubator at 37 °C in a humidified 5% CO_2_ atmosphere and were subcultured two times a week. The antiproliferative activity was studied by MTT assays as published previously [38].

### 3.1. General Procedure for Compounds ***2a**–**2o***

Imine **1** (0.40 mmol, 2 eq) dissolved in acetonitrile (15 mL) was added to a three-necked flask equipped with a reflux condenser with [Ru(C_6_H_6_)Cl_2_]_2_ (0.20 mmol, 1 eq), potassium hexafluorophosphate (0.80 mmol, 4 eq), potassium acetate (0.60 mmol, 3 eq) and refluxed in an argon atmosphere for 24 h. The reaction mixture was evaporated, residue was dissolved in DCM (15 mL) and of water (10 mL), organic layer was separated, aqueous layer was washed with DCM (5 mL), organic phases were combined, dried over sodium sulfate and evaporated to dryness. Crude product was purified with column chromatography on silica gel (CHCl_3_:CH_3_CN 10:1). Crystals suitable for X-ray analysis were grown by diethyl ether vapor diffusion on acetonitrile solution of complexes.

Tetrakis(acetonitrile)[N-((phenyl-κC^2^)methyliden]aniline-κN]ruthenium(II) hexafluorophosphate (**2a**).

Orange powder. Yield 60%.

^1^H NMR (300 MHz, Acetonitrile-*d*_3_) δ 8.46 (s, 1H), 8.03 (d, *J* = 7.5 Hz, 1H), 7.64 (dd, *J* = 7.5, 1.1 Hz, 1H), 7.49–7.42 (m, 2H), 7.39–7.34 (m, 1H), 7.34–7.28 (m, 2H), 7.13 (td, *J* = 7.4, 1.5 Hz, 1H), 6.96 (td, *J* = 7.3, 1.2 Hz, 1H), 2.51 (s, 3H), 2.12 (s, 6H), 1.96 (s, 3H).

^13^C NМR (76 MHz, Acetonitrile-*d*_3_): 192.60, 177.60, 153.08, 150.51, 139.14, 130.67, 129.74, 127.62, 125.01, 123.68, 122.79, 121.55, 66.29, 15.65, 4.40, 3.91, 1.77.

HRMS-ESI: calc for [C_19_H_19_N_4_Ru-CH_3_CN]^+^ 405.0647, found 405.0643.

Tetrakis(acetonitrile)[N-((phenyl-κC^2^)methyliden]-4-fluoroaniline-κN]ruthenium(II) hexafluorophosphate (**2b**).

Orange powder. Yield 73%.

^1^H NMR (300 MHz, Acetonitrile-*d*_3_) δ 8.45 (s, 1H), 8.02 (d, *J* = 7.5 Hz, 1H), 7.63 (d, *J* = 7.4 Hz, 1H), 7.35–7.28 (m, 2H), 7.22–7.10 (m, 3H), 6.96 (t, *J* = 7.3 Hz, 1H), 2.51 (s, 3H), 2.12 (s, 6H), 1.96 (s, 3H).

^13^C NМR (151 MHz, Acetonitrile-*d*_3_): 192.79, 177.94, 163.01, 161.40, 150.41, 149.52, 139.17, 130.80, 129.70, 125.52, 125.05, 122.87, 121.59, 116.34, 116.19, 4.38, 3.91, 1.76.

HRMS-ESI: calc for [C_19_H_18_FN_14_Ru-CH_3_CN]^+^ 423.0534, found 423.0568.

Tetrakis(acetonitrile)[N-((phenyl-κC^2^)methyliden]-4-methylaniline-κN]ruthenium(II) hexafluorophosphate (**2c**).

Orange powder. Yield 65%.

^1^H NMR (300 MHz, Acetonitrile-*d*_3_) δ 8.43 (s, 1H), 8.01 (d, *J* = 7.5 Hz, 1H), 7.61 (dd, *J* = 7.5, 1.1 Hz, 1H), 7.26 (d, *J* = 8.3 Hz, 2H), 7.19 (d, *J* = 8.4 Hz, 2H), 7.12 (td, *J* = 7.4, 1.5 Hz, 1H), 6.96 (td, *J* = 7.3, 1.1 Hz, 1H), 2.51 (s, 3H), 2.39 (s, 3H), 2.11 (s, 6H), 1.96 (s, 3H).

^13^C NМR (151 MHz, Acetonitrile-*d*_3_): 192.35, 177.15, 150.76, 150.54, 139.10, 137.55, 130.48, 130.16, 129.49, 124.92, 123.50, 122.72, 121.53, 21.07, 4.40, 3.92, 1.77.

HRMS-ESI: calc for [C_20_H_21_N_4_Ru-CH_3_CN]^+^ 419.0822, found 419.0814.

Tetrakis(acetonitrile)[N-((phenyl-κC^2^)methyliden]-4-ethylaniline-κN]ruthenium(II) hexafluorophosphate (**2d**)

Orange powder. Yield 48%.

^1^H NMR (300 MHz, Acetonitrile-*d*_3_) δ 8.44 (s, 1H), 8.01 (d, *J* = 7.5 Hz, 1H), 7.63–7.58 (m, 1H), 7.29 (d, *J* = 8.2 Hz, 2H), 7.21 (d, *J* = 8.3 Hz, 2H), 7.12 (t, *J* = 7.3 Hz, 1H), 6.96 (t, *J* = 7.3 Hz, 1H), 2.70 (q, *J* = 7.6 Hz, 2H), 2.51 (s, 3H), 2.12 (s, 6H), 1.96 (s, 3H), 1.26 (t, *J* = 7.6 Hz, 3H).

^13^C NМR (151 MHz, Acetonitrile-*d*_3_): 192.35, 177.18, 150.93, 150.54, 143.98, 139.10, 130.50, 129.51, 129.04, 124.94, 123.59, 122.73, 121.53, 29.06, 16.21, 4.40, 3.93.

HRMS-ESI: calc for [C_21_H_23_N_4_Ru-CH_3_CN]^+^ 433.1210, found 433.1215.

Tetrakis(acetonitrile)[N-((phenyl-κC^2^)methyliden]-4-(tert-butyl)aniline-κN]ruthenium(II) hexafluorophosphate (**2e**).

Orange powder. Yield 56%.

^1^H NMR (300 MHz, Acetonitrile-*d*_3_) δ 8.45 (s, 1H), 8.01 (d, *J* = 7.4 Hz, 1H), 7.62 (d, *J* = 7.4 Hz, 1H), 7.48 (d, *J* = 8.5 Hz, 2H), 7.24 (d, *J* = 8.4 Hz, 2H), 7.12 (t, *J* = 7.3 Hz, 1H), 6.96 (t, *J* = 7.3 Hz, 1H), 2.51 (s, 3H), 2.12 (s, 6H), 1.96 (s, 3H), 1.37 (s, 9H).

^13^C NМR (151 MHz, Acetonitrile-*d*_3_): 192.39, 177.21, 150.69, 139.11, 130.53, 129.53, 126.54, 124.95, 123.27, 122.75, 121.55, 35.25, 31.63, 4.38, 3.91.

HRMS-ESI: calc for [C_23_H_27_N_4_Ru-CH_3_CN]^+^ 315.6547, found 315.6548.

Tetrakis(acetonitrile)[N-((phenyl-κC^2^)methyliden]-4-methoxyaniline-κN]ruthenium(II) hexafluorophosphate (**2f**).

Orange powder. Yield 40%.

^1^H NMR (300 MHz, Acetonitrile-*d*_3_) δ 8.42 (s, 1H), 8.01 (d, *J* = 7.5 Hz, 1H), 7.61 (d, *J* = 7.4 Hz, 1H), 7.26 (d, *J* = 8.8 Hz, 2H), 7.11 (t, *J* = 7.3 Hz, 1H), 7.00–6.92 (m, 3H), 3.84 (s, 3H), 2.51 (s, 3H), 2.11 (s, 6H), 1.96 (s, 3H).

^13^C NМR (151 MHz, Acetonitrile-*d*_3_): 192.15, 176.81, 159.47, 150.56, 146.51, 139.06, 130.36, 129.41, 124.74, 122.70, 121.52, 114.70, 56.24, 3.94.

HRMS-ESI: calc for [C_19_H_18_N_4_ORu]^+^ 419.0812, found 419.0814.

Tetrakis(acetonitrile)[N-((phenyl-κC^2^)methyliden]-4-ethoxycarbonylaniline-κN]ruthenium(II) hexafluorophosphate (**2g**).

Orange powder. Yield 50%.

^1^H NMR (300 MHz, Acetonitrile-*d*_3_) δ 8.50 (s, 1H), 8.10–8.02 (m, 3H), 7.67 (d, *J* = 7.3 Hz, 1H), 7.38 (d, *J* = 8.4 Hz, 2H), 7.14 (t, *J* = 7.4 Hz, 1H), 6.98 (t, *J* = 7.3 Hz, 1H), 4.37 (q, *J* = 7.1 Hz, 2H), 2.52 (s, 3H), 2.12 (s, 6H), 1.96 (s, 3H), 1.38 (t, *J* = 7.1 Hz, 3H).

^13^C NМR (151 MHz, Acetonitrile-*d*_3_): 193.68, 178.55, 166.80, 156.85, 150.40, 139.24, 131.27, 130.99, 129.94, 125.19, 124.02, 122.98, 121.67, 61.96, 14.64, 4.40, 3.92, 1.77.

HRMS-ESI: calc for [C_22_H_23_N_4_O_2_Ru-CH_3_CN]^+^ 477.0853, found 477.0859.

Tetrakis(acetonitrile)[N-((phenyl-κC^2^)methyliden]-4-nitroaniline-κN]ruthenium(II) hexafluorophosphate (**2h**).

Orange powder. Yield 45%.

^1^H NMR (300 MHz, Acetonitrile-*d*_3_) δ 8.54 (s, 1H), 8.33–8.27 (m, 2H),

8.06 (d, *J* = 7.5 Hz, 1H), 7.71 (dd, *J* = 7.5, 1.1 Hz, 1H), 7.51–7.46 (m, 2H), 7.16 (td, *J* = 7.3, 1.4 Hz, 1H), 6.99 (td, *J* = 7.4, 1.1 Hz, 1H), 2.52 (s, 3H), 2.13 (s, 6H), 1.96 (s, 3H).^13^C NМR (151 MHz, Acetonitrile-d3): 194.61, 179.53, 158.41, 150.29, 147.18, 139.31, 131.79, 130.23, 125.41, 124.96, 123.15, 121.79, 4.42, 3.96, 1.77.

HRMS-ESI: calc for [C_19_H_18_N_5_O_2_Ru-CH_3_CN]^+^ 435.0765, found 435.0766.

Tetrakis(acetonitrile)[N-((phenyl-κC^2^)methyliden]-4-(trifluoromethyl)aniline-κN]ruthenium(II) hexafluorophosphate (**2i**).

Orange powder. Yield 42%.

^1^H NMR (300 MHz, Acetonitrile-*d*_3_) δ 8.51 (s, 1H), 8.04 (d, *J* = 7.5 Hz, 1H), 7.77 (d, *J* = 8.4 Hz, 2H), 7.68 (d, *J* = 7.5 Hz, 1H), 7.46 (d, *J* = 8.3 Hz, 2H), 7.15 (t, *J* = 7.4 Hz, 1H), 6.98 (t, *J* = 7.4 Hz, 1H), 2.52 (s, 3H), 2.12 (s, 6H), 1.96 (s, 3H).

^13^C NМR (151 MHz, Acetonitrile-*d*_3_): 193.69, 178.95, 156.18, 150.34, 139.25, 131.35, 130.02, 126.97, 125.23, 124.60, 123.02, 121.70, 4.42, 3.95, 1.77.

HRMS-ESI: calc for [C_20_H_18_F_3_N_4_Ru-CH_3_CN]^+^ 473.0528, 473.0524.

Tetrakis(acetonitrile)[N-((thiophene-2-yl-κC^2^)methyliden]-4-fluoroaniline-κN]ruthenium(II) hexafluorophosphate (**2j**).

Orange powder. Yield 59%.

^1^H NMR (300 MHz, Acetonitrile-*d*_3_) δ 8.38 (s, 1H), 7.82 (d, *J* = 4.6 Hz, 1H), 7.63 (d, *J* = 4.1 Hz, 1H), 7.34–7.27 (m, 2H), 7.20–7.13 (m, 2H), 2.51 (s, 3H), 2.12 (s, 6H), 1.96 (s, 3H).

^13^C NMR (76 MHz, Acetonitrile-*d*_3_) δ 202.67, 168.15, 163.47, 160.26, 149.81, 149.78, 138.48, 137.04, 134.08, 125.66, 125.55, 125.23, 123.44, 116.33, 116.04, 31.61, 30.35, 4.32, 3.89, 1.77.

HRMS-ESI: calc for [C_19_H_19_BrN_5_RuS-CH_3_CN]^+^ 470.0387, found 470.0397.

Tetrakis(acetonitrile)[N-((thiophene-2-yl-κC^2^)methyliden]-4-methylaniline-κN]ruthenium(II) hexafluorophosphate (**2k**).

Orange powder. Yield 48%.

^1^H NMR (300 MHz, Acetonitrile-*d*_3_) δ 8.38 (s, 1H), 7.80 (d, *J* = 4.6 Hz, 1H), 7.62 (d, *J* = 4.6 Hz, 1H), 7.25–7.16 (m, 4H), 2.51 (s, 3H), 2.38 (s, 3H), 2.12 (s, 6H), 1.96 (s, 3H).

^13^C NMR (75 MHz, Acetonitrile-*d*_3_) δ 201.70, 167.47, 151.03, 138.48, 136.98, 136.91, 133.58, 130.16, 125.11, 123.73, 123.29, 21.05, 4.34, 3.91, 1.77.

HRMS-ESI: calc for [C_19_H_19_BrN_5_RuS-CH_3_CN]^+^ 425.0372, found 425.0362.

Tetrakis(acetonitrile)[N-((thiophene-2-yl-κC^2^)methyliden]-4-methoxyaniline-κN]ruthenium(II) hexafluorophosphate (**2l**).

Orange powder. Yield 43%.

^1^H NMR (300 MHz, Acetonitrile-*d*_3_) δ 8.39 (s, 1H), 7.82 (d, *J* = 4.6 Hz, 1H), 7.64 (d, *J* = 4.6 Hz, 1H), 7.30–7.23 (m, 2H), 7.02–6.96 (m, 2H), 3.86 (s, 3H), 2.54 (s, 3H), 2.14 (s, 6H), 1.99 (s, 3H).

^13^C NMR (76 MHz, Acetonitrile-*d*_3_) δ 201.18, 167.17, 159.05, 146.78, 138.41, 136.93, 133.36, 132.27, 129.73, 125.06, 124.87, 123.27, 114.69, 56.19, 4.34, 3.92.

HRMS-ESI: calc for [C_19_H_19_BrN_5_RuS-CH_3_CN]^+^ 482.0587, found 482.0595.

Tetrakis(acetonitrile)[N-((thiophene-2-yl-κC^2^)methyliden]-4-ethoxycarbonylaniline-κN]ruthenium(II) hexafluorophosphate (**2m**).

Orange powder. Yield 32%.

^1^H NMR (300 MHz, Acetonitrile-*d*_3_) δ 8.45 (s, 1H), 8.05 (d, *J* = 8.5 Hz, 2H), 7.88 (d, *J* = 4.7 Hz, 1H), 7.66 (d, *J* = 4.7 Hz, 1H), 7.39 (d, *J* = 8.4 Hz, 2H), 4.36 (q, *J* = 7.1 Hz, 2H), 2.52 (s, 3H), 2.12 (s, 6H), 1.96 (s, 3H), 1.38 (t, *J* = 7.1 Hz, 3H).

^13^C NMR (76 MHz, Acetonitrile-*d*_3_) δ 204.97, 168.66, 166.87, 157.31, 138.94, 137.13, 135.02, 130.96, 129.02, 125.33, 124.17, 123.56, 61.85, 14.63, 4.37, 3.92.

HRMS-ESI: calc for [C_19_H_19_BrN_5_RuS-CH_3_CN]^+^ 524.0694, found 524.0695.

Tetrakis(acetonitrile)[N-((thiophene-2-yl-κC^2^)methyliden]-4-nitroaniline-κN]ruthenium(II) hexafluorophosphate (**2n**).

Orange powder. Yield 42%.

^1^H NMR (300 MHz, Acetonitrile-*d*_3_) δ 8.50 (s, 1H), 8.27 (d, *J* = 9.0 Hz, 2H), 7.92 (d, *J* = 4.7 Hz, 1H), 7.68 (d, *J* = 4.7 Hz, 1H), 7.49 (d, *J* = 9.0 Hz, 2H), 2.52 (s, 3H), 2.12 (s, 6H), 1.96 (s, 3H).

^13^C NMR (76 MHz, Acetonitrile-*d*_3_) δ 207.50, 169.44, 159.04, 146.53, 139.30, 137.28, 136.07, 125.48, 125.39, 124.97, 123.77, 31.59, 30.34, 4.37, 3.94, 1.77.

HRMS-ESI: calc for [C_19_H_19_BrN_5_RuS-CH_3_CN]^+^ 497.0332, found 497.0344.

Tetrakis(acetonitrile)[N-((thiophene-2-yl-κC^2^)methyliden]-4-(trifluoromethyl)aniline-κN]ruthenium(II) hexafluorophosphate (**2o**).

Orange powder. Yield 46%.

^1^H NMR (300 MHz, Acetonitrile-*d*_3_) δ 8.46 (s, 1H), 7.88 (d, *J* = 4.6 Hz, 1H), 7.74 (d, *J* = 8.3 Hz, 2H), 7.66 (d, *J* = 4.6 Hz, 1H), 7.46 (d, *J* = 8.2 Hz, 2H), 2.52 (s, 3H), 2.12 (s, 6H), 1.96 (s, 3H).

^13^C NMR (76 MHz, Acetonitrile-*d*_3_) δ 205.10, 169.01, 156.59, 138.89, 137.13, 135.13, 128.29, 127.86, 126.88 (q, *J* = 3.8 Hz), 125.36, 124.75, 123.60, 4.38, 3.94.

HRMS-ESI: calc for [C_19_H_19_BrN_5_RuS-CH_3_CN]^+^ 520.0356, found 520.0367.

### 3.2. General Procedure for Compounds ***3a**–**3o***

Complex **2** (0.30 mmol, 1 eq) and 2,2′-bipyridine (0.60 mmol, 2 eq) were introduced into a flask equipped with a reflux condenser, ethanol (18 mL) was added, and the mixture was refluxed in an argon atmosphere for 4 h. The reaction mixture was evaporated and purified with column chromatography on silica gel (CHCl_3_:CH_3_CN 10:1). The product was dissolved in minimal volume of CH_3_CN and added dropwise to large excess of diethyl ether under vigorous stirring. The precipitate was filtered, washed with diethyl ether and dried in vacuo for 3 h. Crystals suitable for X-ray analysis were grown by diethyl ether vapor diffusion on acetonitrile solution of complexes.

Bis(2,2′-bipiridine)[N-((phenyl-κC^2^)methyliden]aniline-κN]ruthenium(II) hexafluorophosphate (**3a**).

Dark purple powder. Yield 75%.

^1^H NMR (300 MHz, Acetonitrile-*d*_3_) δ 8.73 (s, 1H), 8.63 (d, *J* = 5.7 Hz, 1H), 8.33 (d, *J* = 8.2 Hz, 2H), 8.13 (d, *J* = 8.2 Hz, 1H), 8.00 (dd, *J* = 12.8, 6.9 Hz, 2H), 7.84 (p, *J* = 7.7 Hz, 3H), 7.78–7.64 (m, 4H), 7.40 (t, *J* = 6.6 Hz, 1H), 7.31 (t, *J* = 6.6 Hz, 1H), 7.19 (t, *J* = 6.5 Hz, 2H), 7.02–6.81 (m, 5H), 6.53 (dd, *J* = 12.5, 7.3 Hz, 3H).

^13^C NМR (151 MHz, Acetonitrile-*d*_3_): 201.76, 176.18, 158.77, 158.01, 157.64, 155.56, 155.43, 153.29, 151.82, 151.24, 150.07, 149.36, 136.77, 136.36, 136.09, 135.10, 134.81, 131.82, 130.09, 129.47, 127.43, 127.39, 127.35, 127.16, 127.04, 124.01, 123.67, 123.21, 122.64, 121.63.

CHN calc. for С_33_H_26_N_5_RuPF_6_·0.2C_4_H_10_O: C: 53.29; H: 3.53; N: 9.31. Obs. C: 53.29; H: 3.52; N: 9.39.

HRMS-ESI: calc for [C_33_H_26_N_5_Ru]^+^ 594.1235, found 594.1228.

Bis(2,2′-bipiridine)[N-((phenyl-κC^2^)methyliden]-4-fluoroaniline-κN]ruthenium(II) hexafluorophosphate (**3b**).

Dark purple powder. Yield 77%.

^1^H NMR (300 MHz, Acetonitrile-*d*_3_) δ 8.72 (s, 1H), 8.59 (d, *J* = 5.7 Hz, 1H), 8.34 (d, *J* = 8.1 Hz, 2H), 8.15 (d, *J* = 8.1 Hz, 1H), 8.05 (d, *J* = 8.2 Hz, 1H), 7.96 (d, *J* = 5.6 Hz, 1H), 7.84 (p, *J* = 7.2 Hz, 3H), 7.78–7.70 (m, 3H), 7.66 (d, *J* = 5.3 Hz, 1H), 7.39 (t, *J* = 6.6 Hz, 1H), 7.31 (t, *J* = 6.7 Hz, 1H), 7.20 (q, *J* = 6.2 Hz, 2H), 6.87 (p, *J* = 7.4 Hz, 2H), 6.66 (t, *J* = 8.7 Hz, 2H), 6.58–6.47 (m, 3H).

^13^C NМR (151 MHz, Acetonitrile-*d*_3_): 201.80, 176.52, 162.38, 160.77, 158.72, 157.99, 157.62, 155.57, 155.44, 153.27, 151.29, 150.10, 149.28, 148.17, 136.88, 136.38, 136.16, 135.16, 134.87, 131.91, 130.17, 127.53, 127.51, 127.35, 127.19, 124.44, 124.38, 124.05, 123.74, 123.32, 121.68, 116.05, 115.90.

CHN calc. for C_33_H_25_N_5_RuPF_7_: C: 52.39; H: 3.33; N: 9.26. Obs. C: 52.23; H: 3.21; N: 9.09.

HRMS-ESI: calc for [C_33_H_25_FN_5_Ru]^+^ 612.1141, found 612.1142.

Bis(2,2′-bipiridine)[N-((phenyl-κC^2^)methyliden]-4-methylaniline-κN]ruthenium(II) hexafluorophosphate (**3c**).

Dark purple powder. Yield 82%.

^1^H NMR (300 MHz, Acetonitrile-*d*_3_) δ 8.70 (s, 1H), 8.61 (d, *J* = 5.7 Hz, 1H), 8.33 (d, *J* = 8.2 Hz, 2H), 8.14 (d, *J* = 8.1 Hz, 1H), 8.04 (d, *J* = 8.1 Hz, 1H), 7.96 (d, *J* = 5.6 Hz, 1H), 7.88–7.78 (m, 3H), 7.76–7.68 (m, 3H), 7.66 (d, *J* = 5.3 Hz, 1H), 7.39 (t, *J* = 6.7 Hz, 1H), 7.30 (t, *J* = 6.7 Hz, 1H), 7.22–7.16 (m, 2H), 6.91–6.80 (m, 2H), 6.73 (d, *J* = 8.0 Hz, 2H), 6.52 (d, *J* = 7.1 Hz, 1H), 6.41 (d, *J* = 8.0 Hz, 2H), 2.12 (s, 3H).

^13^C NМR (76 MHz, Acetonitrile-*d*_3_): 201.38, 175.74, 158.79, 158.00, 157.64, 155.57, 155.41, 153.28, 151.20, 150.05, 149.59, 149.41, 137.03, 136.76, 136.35, 136.02, 135.05, 134.76, 131.59, 129.93, 129.88, 127.38, 127.34, 127.14, 123.99, 123.67, 123.26, 122.44, 121.65, 20.77.

CHN calc. for C_34_H_28_N_5_RuPF_6_: C: 54.26; H: 3.75; N: 9.30. Obs. C: 54.07; H: 3.70; N: 9.34.

HRMS-ESI: calc for [C_34_H_28_N_5_Ru]^+^ 608.1384, found 608.1385.

Bis(2,2′-bipiridine)[N-((phenyl-κC^2^)methyliden]-4-ethylaniline-κN]ruthenium(II) hexafluorophosphate (**3d**).

Dark purple powder. Yield 85%.

^1^H NMR (300 MHz, Acetonitrile-*d*_3_) δ 8.71 (s, 1H), 8.62 (d, *J* = 5.5 Hz, 1H), 8.33 (d, *J* = 8.3 Hz, 2H), 8.12 (d, *J* = 8.1 Hz, 1H), 8.02–7.95 (m, 2H), 7.89–7.78 (m, 3H), 7.76–7.63 (m, 4H), 7.39 (ddd, *J* = 7.3, 5.7, 1.4 Hz, 1H), 7.30 (ddd, *J* = 7.3, 5.7, 1.4 Hz, 1H), 7.19 (ddd, *J* = 7.5, 6.0, 1.1 Hz, 2H), 6.89 (td, *J* = 7.3, 1.5 Hz, 1H), 6.83 (td, *J* = 7.2, 1.7 Hz, 1H), 6.76–6.71 (m, 2H), 6.56–6.51 (m, 1H), 6.41–6.36 (m, 2H), 2.42 (q, *J* = 7.6 Hz, 2H), 1.05 (t, *J* = 7.6 Hz, 3H).

^13^C NМR (76 MHz, Acetonitrile-*d*_3_): 201.40, 175.63, 158.82, 158.01, 157.63, 155.59, 155.37, 153.30, 151.26, 150.04, 149.46, 143.50, 136.74, 136.30, 136.03, 135.02, 134.73, 131.59, 129.98, 128.73, 127.37, 127.33, 127.12, 124.00, 123.60, 123.19, 122.49, 121.61, 28.77, 16.25.

CHN calc. for C_35_H_30_N_5_RuPF_6_: C: 54.66; H: 3.94; N: 9.31. Obs. C: 54.87; H: 3.93; N: 9.13.

HRMS-ESI: calc for [C_35_H_30_N_5_Ru]^+^ 622.1548, found 622.1563.

Bis(2,2′-bipiridine)[N-((phenyl-κC^2^)methyliden]-4-(tert-butyl)aniline-κN]ruthenium(II) hexafluorophosphate (**3e**).

Dark purple powder. Yield 78%.

^1^H NMR (300 MHz, Acetonitrile-*d*_3_) δ 8.71 (s, 1H), 8.64 (d, *J* = 5.8 Hz, 1H), 8.34 (d, *J* = 8.1 Hz, 2H), 8.09 (d, *J* = 8.1 Hz, 1H), 7.98 (d, *J* = 5.7 Hz, 1H), 7.94 (d, *J* = 8.1 Hz, 1H), 7.89–7.77 (m, 4H), 7.73 (d, *J* = 7.3 Hz, 1H), 7.67 (t, *J* = 7.8 Hz, 1H), 7.62 (d, *J* = 5.3 Hz, 1H), 7.39 (t, *J* = 6.6 Hz, 1H), 7.30 (t, *J* = 6.6 Hz, 1H), 7.19 (q, *J* = 6.0 Hz, 2H), 6.92–6.81 (m, 4H), 6.55 (d, *J* = 7.2 Hz, 1H), 6.33 (d, *J* = 8.0 Hz, 2H), 1.15 (s, 9H).

^13^C NМR (76 MHz, Acetonitrile-*d*_3_): 201.27, 175.45, 158.88, 158.02, 157.63, 155.62, 155.34, 153.32, 151.33, 150.10, 150.05, 149.47, 148.76, 136.71, 136.28, 136.04, 134.97, 134.69, 131.53, 130.00, 127.37, 127.29, 127.25, 127.08, 126.15, 124.02, 123.52, 123.14, 122.09, 121.60, 34.87, 31.43.

CHN calc. for C_37_H_34_N_5_RuPF_6_: C: 55.92; H: 3.84; N: 8.73. Obs. C: 55.90; H: 4.24; N: 9.10.

HRMS-ESI: calc for [C_37_H_34_N_5_Ru]^+^ 650.1862, found 650.1855.

Bis(2,2′-bipiridine)[N-((phenyl-κC^2^)methyliden]-4-methoxyaniline-κN]ruthenium(II) hexafluorophosphate (**3f**).

Dark purple powder. Yield 84%.

^1^H NMR (300 MHz, Acetonitrile-*d*_3_) δ 8.69 (s, 1H), 8.60 (d, *J* = 5.0 Hz, 1H), 8.33 (d, *J* = 8.2 Hz, 2H), 8.14 (d, *J* = 8.1 Hz, 1H), 8.05 (d, *J* = 8.2 Hz, 1H), 7.98–7.94 (m, 1H), 7.88–7.78 (m, 3H), 7.76–7.70 (m, 3H), 7.68–7.65 (m, 1H), 7.39 (ddd, *J* = 7.3, 5.7, 1.4 Hz, 1H), 7.30 (ddd, *J* = 7.4, 5.7, 1.4 Hz, 1H), 7.23–7.16 (m, 2H), 6.88 (td, *J* = 7.2, 1.5 Hz, 1H), 6.82 (td, *J* = 7.2, 1.7 Hz, 1H), 6.55–6.50 (m, 1H), 6.45 (s, 4H), 3.62 (s, 3H).

^13^C NМR (151 MHz, Acetonitrile-*d*_3_): 201.23, 175.37, 158.88, 158.78, 158.02, 157.66, 155.63, 155.37, 153.29, 151.26, 150.04, 149.42, 145.19, 136.78, 136.28, 135.99, 135.03, 134.73, 131.49, 129.90, 127.40, 127.32, 127.15, 124.01, 123.67, 123.26, 121.61, 114.47, 56.07.

CHN calc. for C_34_H_28_N_5_ORuPF_6_: C: 53.13; H: 3.67; N: 9.11. Obs. C: 52.87; H: 3.76; N: 9.17.

HRMS-ESI: calc for [C_34_H_28_N_5_ORu]^+^ 624.1341, found 624.1327.

Bis(2,2′-bipiridine)[N-((phenyl-κC^2^)methyliden]-4-ethoxycarbonylaniline-κN]ruthenium(II) hexafluorophosphate (**3g**).

Dark purple powder. Yield 75%.

^1^H NMR (300 MHz, Acetonitrile-*d*_3_) δ 8.78 (s, 1H), 8.60 (dd, *J* = 5.7, 0.7 Hz, 1H), 8.34 (dq, *J* = 8.2, 1.2 Hz, 2H), 8.14 (d, *J* = 8.0 Hz, 1H), 8.02 (d, *J* = 8.2 Hz, 1H), 7.97–7.94 (m, 1H), 7.90–7.78 (m, 4H), 7.73–7.65 (m, 3H), 7.57–7.52 (m, 2H), 7.40 (ddd, *J* = 7.3, 5.7, 1.4 Hz, 1H), 7.32 (ddd, *J* = 7.3, 5.7, 1.4 Hz, 1H), 7.24–7.17 (m, 2H), 6.91 (td, *J* = 7.3, 1.5 Hz, 1H), 6.86 (td, *J* = 7.3, 1.7 Hz, 1H), 6.63–6.56 (m, 3H), 4.25 (q, *J* = 7.1 Hz, 2H), 1.30 (t, *J* = 7.1 Hz, 3H).

^13^C NМR (76 MHz, Acetonitrile-*d*_3_): 202.81, 177.17, 166.39, 158.66, 157.97, 157.62, 155.65, 155.49, 155.45, 153.33, 151.27, 150.09, 149.26, 136.91, 136.47, 136.26, 135.29, 134.99, 132.41, 130.66, 130.39, 129.05, 127.59, 127.55, 127.41, 127.22, 124.07, 123.79, 123.35, 122.99, 121.74, 61.90, 14.51.

CHN calc. for C_36_H_30_N_5_O_2_RuPF_6_·0.16CHCl_3_: C: 52.13; H:3.88; N: 8.71. Obs. C: 52.40; H:3.84; N: 8.73.

HRMS-ESI: calc for [C_36_H_30_N_5_O_2_Ru]^+^ 666.1447, found 666.1447.

Bis(2,2′-bipiridine)[N-((phenyl-κC^2^)methyliden]-4-nitroaniline-κN]ruthenium(II) hexafluorophosphate (**3h**).

Dark purple powder. Yield 79%.

^1^H NMR (300 MHz, Acetonitrile-*d*_3_) δ 8.85–8.78 (m, 1H), 8.60–8.53 (m, 1H), 8.38–8.31 (m, 2H), 8.15–8.09 (m, 1H), 8.04–7.98 (m, 1H), 7.93 (t, *J* = 5.6 Hz, 1H), 7.90–7.79 (m, 4H), 7.77–7.64 (m, 5H), 7.43–7.36 (m, 1H), 7.33 (t, *J* = 6.6 Hz, 1H), 7.27–7.17 (m, 2H), 6.96–6.83 (m, 2H), 6.72–6.64 (m, 2H), 6.63–6.57 (m, 1H).

^13^C NМR (151 MHz, Acetonitrile-*d*_3_): 203.76, 178.13, 158.61, 157.98, 157.63, 157.14, 155.56, 155.38, 153.38, 151.36, 150.08, 149.21, 146.42, 137.11, 136.56, 136.42, 135.47, 135.15, 132.94, 130.69, 127.83, 127.68, 127.46, 127.27, 125.01, 124.16, 124.13, 123.92, 123.57, 121.84.

CHN calc. for C_33_H_25_N_6_O_2_PF_6_Ru·0.44CHCl_3_·0.34C_4_H_10_O: C: 48.51; H: 3.38; N: 9.75. Obs. C: 48.51; H: 3.21; N: 9.75.

HRMS-ESI: calc for [C_33_H_25_N_6_O_2_Ru]^+^ 639.1086, found 639.1070.

Bis(2,2′-bipiridine)[N-((phenyl-κC^2^)methyliden]-4-(trifluoromethyl)aniline-κN]ruthenium(II) hexafluorophosphate (**3i**).

Dark purple powder. Yield 82%.

^1^H NMR (300 MHz, Acetonitrile-*d*_3_) δ 8.78 (s, 1H), 8.59 (d, *J* = 5.7 Hz, 1H), 8.34 (d, *J* = 8.1 Hz, 2H), 8.13 (d, *J* = 8.1 Hz, 1H), 8.02–7.95 (m, 2H), 7.91–7.78 (m, 4H), 7.74–7.63 (m, 3H), 7.40 (t, *J* = 6.7 Hz, 1H), 7.33 (t, *J* = 6.7 Hz, 1H), 7.25–7.17 (m, 4H), 6.89 (q, *J* = 6.6 Hz, 2H), 6.64 (d, *J* = 8.0 Hz, 2H), 6.59 (d, *J* = 6.9 Hz, 1H).

^13^C NМR (76 MHz, Acetonitrile-*d*_3_): 202.68, 177.44, 158.67, 157.98, 157.62, 155.55, 155.49, 153.33, 151.32, 150.15, 149.26, 136.90, 136.51, 136.32, 135.32, 135.03, 132.44, 130.46, 127.63, 127.40, 127.22, 126.69, 126.64, 126.59, 126.54, 124.09, 123.80, 123.52, 123.31, 121.79.

CHN calc. for C_34_H_25_N_5_RuPF_9_·0.16CH_3_CN: C: 50.47; H: 3.15; N: 8.85. Obs. C: 50.47; H: 3.15; N: 8.84.

HRMS-ESI: calc for [C_34_H_25_F_3_N_5_Ru]^+^ 662.1109, found 662.1100.

Bis(2,2′-bipiridine)[N-((thiophene-2-yl-κC^2^)methyliden]-4-fluoroaniline-κN]ruthenium(II) hexafluorophosphate (**3j**).

Dark purple powder. Yield 82%.

^1^H NMR (300 MHz, Acetonitrile-*d*_3_) δ 8.64 (s, 1H), 8.51 (d, *J* = 4.9 Hz, 1H), 8.32 (d, *J* = 8.1 Hz, 2H), 8.14 (d, *J* = 8.0 Hz, 1H), 8.07 (d, *J* = 8.2 Hz, 1H), 7.91–7.71 (m, 6H), 7.68 (d, *J* = 4.7 Hz, 1H), 7.61 (d, *J* = 4.7 Hz, 1H), 7.41 (ddd, 1H), 7.31 (ddd, 1H), 7.26–7.16 (m, 2H), 6.68–6.58 (m, 2H), 6.51–6.43 (m, 3H).

^13^C NMR (76 MHz, Acetonitrile-*d*_3_) δ 212.13, 166.43, 162.82, 159.60, 158.52, 158.39, 157.85, 155.74, 155.42, 153.95, 151.77, 150.03, 148.60, 148.56, 138.59, 136.93, 136.22, 135.82, 135.46, 135.18, 134.74, 127.58, 127.50, 127.31, 127.16, 124.61, 124.50, 124.03, 123.85, 123.69, 123.38, 116.06, 115.76.

CHN calc. for C_31_H_23_F_7_N_5_OPRuS: C: 48.82; H: 3.04; N: 9.18 Obs. C: 48.83; H: 3.04; N: 9.18.

HRMS-ESI: calc for [C_31_H_23_FN_5_ORuS]^+^ 618.0704, found 618.0706.

Bis(2,2′-bipiridine)[N-((thiophene-2-yl-κC^2^)methyliden]-4-methylaniline-κN]ruthenium(II) hexafluorophosphate (**3k**).

Dark purple powder. Yield 80%.

^1^H NMR (300 MHz, Acetonitrile-*d*_3_) δ 8.63 (s, 1H), 8.53 (d, *J* = 5.7 Hz, 1H), 8.31 (d, *J* = 8.1 Hz, 2H), 8.14 (d, *J* = 8.0 Hz, 1H), 8.06 (d, *J* = 8.1 Hz, 1H), 7.90–7.69 (m, 6H), 7.67–7.63 (m, 1H), 7.61 (d, *J* = 5.4 Hz, 1H), 7.44–7.36 (m, 1H), 7.33–7.26 (m, 1H), 7.19 (q, 2H), 6.71 (d, *J* = 8.1 Hz, 2H), 6.45 (d, *J* = 4.6 Hz, 1H), 6.39 (d, *J* = 8.3 Hz, 2H), 2.11 (s, 3H).

^13^C NMR (76 MHz, Acetonitrile-*d*_3_) δ 211.31, 165.81, 158.60, 158.40, 157.88, 155.77, 155.43, 153.94, 151.67, 149.99, 138.53, 136.81, 136.32, 136.08, 135.36, 135.33, 135.08, 134.70, 129.90, 127.44, 127.37, 127.28, 127.16, 123.99, 123.80, 123.65, 123.34, 122.66, 20.75.

CHN calc. for C_32_H_26_F_6_N_5_PRuS: C: 50.66; H: 3.45; N: 9.23 Obs. C: 50.48; H: 3.48; N: 9.20.

HRMS-ESI: calc for [C_32_H_26_N_5_RuS]^+^ 614.0955, found 614.0964.

Bis(2,2′-bipiridine)[N-((thiophene-2-yl-κC^2^)methyliden]-4-methoxyaniline-κN]ruthenium(II) hexafluorophosphate (**3l**).

Dark purple powder. Yield 77%.

^1^H NMR (300 MHz, Acetonitrile-*d*_3_) δ 8.61 (s, 1H), 8.53 (d, *J* = 4.9 Hz, 1H), 8.31 (d, *J* = 8.1 Hz, 2H), 8.14 (d, *J* = 8.1 Hz, 1H), 8.06 (d, *J* = 8.2 Hz, 1H), 7.90–7.70 (m, 6H), 7.64 (d, *J* = 4.6 Hz, 1H), 7.61 (d, *J* = 4.6 Hz, 1H), 7.40 (ddd, *J* = 7.3, 5.7, 1.4 Hz, 1H), 7.30 (ddd, *J* = 7.3, 5.7, 1.4 Hz, 1H), 7.25–7.15 (m, 2H), 6.45 (d, *J* = 4.6 Hz, 1H), 6.42 (br.s, 3H), 3.61 (s, 3H).

^13^C NMR (76 MHz, Acetonitrile-*d*_3_) δ 210.71, 165.46, 158.59, 158.44, 158.41, 157.88, 155.82, 155.39, 153.93, 151.72, 149.99, 145.56, 138.48, 136.82, 136.06, 135.32, 135.10, 135.04, 134.66, 127.45, 127.37, 127.27, 127.13, 123.99, 123.81, 123.61, 123.32, 114.48, 56.03, 15.64.

CHN calc. for C_32_H_26_F_6_N_5_OPRuS: C: 49.61; H: 3.38; N: 9.04 Obs. C: 49.60; H: 3.39; N: 9.08.

HRMS-ESI: calc for [C_32_H_26_N_5_ORuS]^+^ 630.0904, found 630.0912.

Bis(2,2′-bipiridine)[N-((thiophene-2-yl-κC^2^)methyliden]-4-ethoxycarbonylaniline-κN]ruthenium(II) hexafluorophosphate (**3m**).

Dark purple powder. Yield 77%.

^1^H NMR (300 MHz, Acetonitrile-*d*_3_) δ 8.72 (s, 1H), 8.50 (d, *J* = 4.9 Hz, 1H), 8.33 (d, *J* = 8.4 Hz, 2H), 8.13 (d, *J* = 8.1 Hz, 1H), 8.05 (d, *J* = 8.2 Hz, 1H), 7.92–7.70 (m, 7H), 7.63 (d, *J* = 4.7 Hz, 1H), 7.55–7.49 (m, 2H), 7.42 (ddd, *J* = 7.3, 5.7, 1.4 Hz, 1H), 7.32 (ddd, *J* = 7.3, 5.7, 1.4 Hz, 1H), 7.25 (ddd, *J* = 7.5, 5.4, 1.2 Hz, 1H), 7.19 (ddd, *J* = 7.3, 5.7, 1.4 Hz, 1H), 6.63–6.56 (m, 2H), 6.49 (d, *J* = 4.6 Hz, 1H), 4.24 (q, *J* = 7.1 Hz, 2H), 1.29 (t, *J* = 7.1 Hz, 3H).

^13^C NMR (76 MHz, Acetonitrile-*d*_3_) δ 166.89, 166.51, 158.46, 158.41, 157.89, 156.34, 155.61, 155.45, 154.08, 151.80, 150.05, 137.02, 136.80, 136.35, 135.63, 135.37, 134.88, 130.66, 128.29, 127.70, 127.53, 127.36, 127.25, 124.07, 123.90, 123.76, 123.46, 123.19, 61.80, 14.52.

CHN calc. for C_34_H_28_F_6_N_5_O_2_PRuS: C: 50.00; H: 3.46; N: 8.57 Obs. C: 50.03; H: 3.48; N: 8.56.

HRMS-ESI: calc for [C_34_H_28_N_5_O_2_RuS]^+^ 672.1010, found 672.1011.

Bis(2,2′-bipiridine)[N-((thiophene-2-yl-κC^2^)methyliden]-4-nitroaniline-κN]ruthenium(II) hexafluorophosphate (**3n**).

Dark purple powder. Yield 65%.

^1^H NMR (300 MHz, Acetonitrile-*d*_3_) δ 8.78 (s, 1H), 8.48 (d, *J* = 5.6 Hz, 1H), 8.34 (d, *J* = 7.6 Hz, 2H), 8.12 (d, *J* = 8.0 Hz, 1H), 8.05 (d, *J* = 8.2 Hz, 1H), 7.95–7.71 (m, 9H), 7.64 (d, *J* = 5.3 Hz, 1H), 7.43 (t, *J* = 6.6 Hz, 1H), 7.35–7.26 (m, 2H), 7.20 (t, *J* = 6.7 Hz, 1H), 6.68 (d, *J* = 9.0 Hz, 2H), 6.52 (d, *J* = 4.7 Hz, 1H).

^13^C NMR (76 MHz, Acetonitrile-*d*_3_) δ 217.12, 167.65, 158.41, 158.36, 158.11, 157.88, 155.48, 155.42, 154.14, 151.90, 149.98, 145.73, 139.52, 137.86, 137.24, 136.52, 135.82, 135.56, 135.02, 127.96, 127.64, 127.43, 127.30, 125.02, 124.13, 124.00, 123.95, 123.86, 123.67.

CHN calc. for C_31_H_23_F_6_N_6_O_2_PRuS: C: 47.15; H: 2.94; N: 10.64 Obs. C: 46.99; H: 2.96; N: 10.61.

HRMS-ESI: calc for [C_31_H_23_N_6_O_2_RuS]^+^ 645.0649, found 645.0666.

Bis(2,2′-bipiridine)[N-((thiophene-2-yl-κC^2^)methyliden]-4-(trifluoromethyl)aniline-κN]ruthenium(II) hexafluorophosphate (**3o**).

Dark purple powder. Yield 96%.

^1^H NMR (300 MHz, Acetonitrile-*d*_3_) δ 8.73 (s, 1H), 8.51 (d, *J* = 4.9 Hz, 1H), 8.33 (d, *J* = 7.2 Hz, 2H), 8.13 (d, *J* = 8.1 Hz, 1H), 8.04 (d, *J* = 8.2 Hz, 1H), 7.93–7.70 (m, 7H), 7.62 (d, *J* = 4.8 Hz, 1H), 7.42 (ddd, *J* = 7.4, 5.7, 1.4 Hz, 1H), 7.32 (ddd, *J* = 7.3, 5.7, 1.4 Hz, 1H), 7.27–7.17 (m, 4H), 6.63 (d, *J* = 8.0 Hz, 2H), 6.51 (d, *J* = 4.7 Hz, 1H).

^13^C NMR (76 MHz, Acetonitrile-*d*_3_) δ 214.53, 167.20, 158.45, 158.41, 157.87, 155.63, 155.49, 155.38, 154.06, 151.82, 150.06, 139.05, 137.01, 136.87, 136.40, 135.65, 135.38, 134.87, 127.73, 127.59, 127.37, 127.23, 126.64, 126.59, 126.54, 126.48, 124.09, 123.92, 123.76, 123.71, 123.41.

CHN calc. for C_32_H_23_F_9_N_5_PRuS: C: 47.30; H: 2.85; N: 8.62 Obs. C: 47.28; H: 2.79; N: 8.63.

HRMS-ESI: calc for [C_32_H_23_F_3_N_5_RuS]^+^ 668.0672, found 668.0656.

## 4. Conclusions

Two series of bisheteroleptic ruthenium(II) compounds containing different types of the simplest cyclometallated imines of benzaldehyde and 2-thiophenecarboxaldehyde were synthesized and characterized by NMR, MS, elemental analysis and UV-vis spectroscopy and cyclic voltammetry. The structure of 22 complexes described was proved by means of single-crystal X-ray data. Cytotoxic activity was measured on two cancer and one non-cancerous cell line, and the proportion of cell death was measured using the MTT assay. The cytotoxicity of all ruthenium complexes is significantly higher than that of cisplatin, but they have less selectivity.

## Figures and Tables

**Table 1:** Selected bond lengths and angles of ruthenium complexes.

	Aniline-(Ru–N) Dihedral Angle, °	Py-Py Dihedral Angle, °	Ru–N(Aniline), Å	Ru–N, Å ^a^	Ru–C, Å
**2a** (H)	+65.66		2.071	2.151	2.026
**2b** (F)	+58.36		2.065	2.155	2.035
**2d** (Et)	−52.43		2.069	2.142	2.027
**2e** (tBu)	−57.51		2.069	2.155	2.025
**2f** (OMe)	−61.74		2.066	2.152	2.019
**2g** (CO_2_Et) ^b^	−51.97		2.062	2.158	2.023
	+40.65		2.079	2.159	2.017
**2h** (NO_2_) ^b^	+42.47		2.096	2.157	2.019
	+41.62		2.071	2.172	2.008
**2i** (CF_3_)	−51.20		2.063	2.153	2.022
**2j** (F)	−47.59		2.102	2.139	2.019
**2k** (Me)	−49.30		2.096	2.136	2.022
**2l** (OMe) b	+52.94		2.093	2.132	2.018
	+41.76		2.114	2.139	2.015
**2n** (NO_2_)	−47.17		2.105	2.140	2.020
**3a** (H)	+45.61	4.22	2.111	2.146	2.031
**3b** (F)	+49.65	1.85	2.104	2.136	2.030
**3c** (Me)	+39.77	5.01	2.117	2.152	2.031
**3e** (tBu)	+48.29	7.07	2.085	2.146	2.029
**3f** (OMe)	−55.21	3.52	2.063	2.155	2.036
**3h** (NO_2_)	−52.22	3.96	2.091	2.156	2.031
**3j** (F)	+43.78	8.91	2.123	2.137	2.025
**3l** (OMe)	+48.94	10.77	2.117	2.148	2.010
**3m** (CO_2_Et)	−58.58	5.47	2.110	2.137	2.018
**3o** (CF_3_)	+55.99	3.79	2.106	2.127	2.019

^a^ located opposite the Ru–C bond. ^b^ two independent molecules.

**Table 2:** The E^1/2^ values (mV vs. Fc/Fc^+^) of reduction and oxidation of the studied compounds in 0.1 M Bu_4_NPF_6_/MeCN were determined as the half-sum of the potentials of the forward and reverse peaks of the CV curves recorded for solutions with a concentration of 2.5 × 10^−3^ M at a potential scan rate of 100 mV s^−1^ on a glassy carbon working electrode at 298 K (for irreversible processes marked with an asterisk, the peak potentials are given).

	${E_{p}(E}_{red2}^{1/2}),\;mV$	${E_{p}(E}_{red1}^{1/2}),\;mV$	${E_{p}(E}_{ox1}^{1/2}),\;mV$	${E_{p}(E}_{ox2}^{1/2}),\;mV$	Additional Peaks
**3a** (H)	−2277 (−2234)	−2022 (−1981)	139 (95)	1565	
**3b** (F)	−2264 (−2219)	−2010 (−1969)	155 (111)	1607	−2828
**3c** (Me)	−2015 (−1973)	−2268 (−2226)	144 (99)	1570	
**3d** (Et)	−2278 (−2233)	−2020 (−1981)	136 (92)	1551	−2905
**3e** (tBu)	−2280 (−2236)	−2026 (−1984)	128 (88)	1556	−2864
**3f** (OMe)	−2277 (−2235)	−2022 (−1984)	117 (78)	1352	
**3g** ^a^ (CO_2_Et)	−2242	−2009 (−1961)	189 (135)	1635	−2551 (C=O reduction)
**3h** ^b^ (NO_2_)	-	-	200 (161)	1679	−1507 (−1467) –NO_2_ reduction −1970
**3i** (CF_3_)	−2245 (−2198)	−1996 (−1959)	181 (139)	1611	−2640 (C–F cleavage)
**3j** (F)	−2245 (−2202)	−1997 (−1956)	145 (102)	1422	
**3k** (Me)	−2253 (−2213)	−2002 (−1962)	117 (77)	1364	−2909
**3l** (OMe)	−2256 (−2216)	−2003 (−1962)	107 (70)	1183 (1129)	−2874 (shoulder)
**3m** ^c^ (CO_2_Et)	−2226 (−2167)	−1989 (−1931)	78 (127)	1517	−2582 (C=O reduction)
**3n** ^d^ (NO_2_)	-	-	215 (165)	1545	−1534 (−1488) (–NO_2_ reduction)
**3o** (CF_3_)	−2213 (−2175)	−1976 (−1936)	181 (143)	1460	−2582 (C–F cleavage)

^a^ C=O reduction is reversible, but too distorted, also affects $E_{red1}^{1/2}$ peak reversibility. ^b^ After first reduction of –NO_2_ further curve is uninterpretable. ^c^ C=O reduction is reversible, but too distorted. ^d^ Very distorted $E_{red2}^{1/2}$ and $E_{red1}^{1/2}$.

**Table 3:** Antiproliferative activity of ruthenium complexes **3a**–**3o**, ligands **1b, 1f, 1g** and cisplatin against various human cancer cells R_f_ showed resistance coefficient (calculated as IC_50_ on A2780cis divided on IC_50_ on A2780 cell line). The results are the mean values ± SD of three independent experiments, each of which was done in triplicate. The Selectivity Index is calculated as IC50 on HEK293 divided on IC_50_ on A2780 cell line.

	IC_50_ (72 h)/nМ				
Compound	А2780	A2780Cis	Rf	HEK293	Selectivity Index, SI
Cisplatin	2640 ± 350	1560 ± 210	5.9	22,000 ± 4000	8.3
**3a** (H)	33 ± 7	30 ± 10	0.9	53 ± 4	1.6
**3b** (F)	90 ± 30	250 ± 60	2.8	100 ± 30	1.1
**3c** (Me)	70 ± 6	120 ± 10	1.7	70 ± 10	1.0
**3d** (Et)	45 ± 5	150 ± 10	3.3	80 ± 10	1.8
**3e** (tBu)	70 ± 30	170 ± 30	2.4	68 ± 6	1.0
**3f** (OMe)	65 ± 4	120 ± 40	1.8	64 ± 7	1.0
**3g** (CO_2_Et)	100 ± 20	230 ± 20	2.3	140 ± 20	1.4
**3h** (NO_2_)	250 ± 30	760 ± 80	3.0	370 ± 80	1.5
**3i** (CF_3_)	80 ± 10	240 ± 20	3.0	107 ± 8	1.3
**3j** (F)	43 ± 3	91 ± 9	2.1	60 ± 10	1.4
**3k** (Me)	30 ± 2	10 ± 0.4	0.3	70 ± 30	2.3
**3l** (OMe)	39 ± 3	39 ± 10	1.0	40 ± 10	1.0
**3m** (CO_2_Et)	57 ± 20	84 ± 12	1.5	140 ± 10	2.5
**3n** (NO_2_)	270 ± 4	960 ± 100	3.6	530 ± 30	2.0
**3o** (CF_3_)	96 ± 3	150 ± 5	6.4	160 ± 30	1.7
**1b** (F)	>200,000	>100,000	-	21,610	-
**1f** (OMe)	>200,000	>100,000	-	>200,000	-
**1g** (CO_2_Et)	42,090	>100,000	-	25,430	-

## Data Availability

Data is contained within the article and Supplementary Materials.

## Supplementary Materials

The following supporting information can be downloaded at: https://www.mdpi.com/article/10.3390/molecules31020315/s1, Vasiliev_SI_Rev3, Experimental details: pp. S2–S4; NMR spectra: pp. S5–S34; Crystallography: pp. S35–S52; Stability in PBS: pp. S53–S54.
