# Supplementary material for: Cycloruthenated Imines: A Step into the Nanomolar Region
Source: Molecules. 2026 Jan 16;31(2):315. doi: 10.3390/molecules31020315 (PMC12844335; doi:10.3390/molecules31020315)

checkCIF/PLATON report

Structure factors have been supplied for datablock(s) 1n

THIS REPORT IS FOR GUIDANCE ONLY. IF USED AS PART OF A REVIEW PROCEDURE FOR PUBLICATION, IT SHOULD NOT REPLACE THE EXPERTISE OF AN EXPERIENCED CRYSTALLOGRAPHIC REFEREE.

No syntax errors found. CIF dictionary Interpreting this report

Datablock: 1n

Bond precision:	C-C = 0.0019 A		Wavelength=1.54184
Cell:	a=9.2741(1)	b=11.9955(1)	c=18.4996(2)
	alpha=90	beta=92.689(1)	gamma=90
Temperature:	100 K		

	Calculated	Reported
Volume	2055.77(4)	2055.77(4)
Space group	C 2/c	C 1 2/c 1
Hall group	-C 2yc	-C 2yc
Moiety formula	C11 H8 N2 O2 S	C11 H8 N2 O2 S
Sum formula	C11 H8 N2 O2 S	C11 H8 N2 O2 S
Mr	232.25	232.25
Dx,g cm-3	1.501	1.501
Z	8	8
Mu (mm-1)	2.692	2.692
F000	960.0	960.0
F000'	965.39	
h,k,lmax	11,15,23	11,15,23
Nref	2249	2247
Tmin,Tmax	0.441,0.616	0.203,1.000
Tmin'	0.333	

Correction method= # Reported T Limits: Tmin=0.203 Tmax=1.000
AbsCorr = GAUSSIAN

Data completeness= 0.999 Theta(max)= 80.024

R(reflections)= 0.0336(2184) wR2(reflections)= 0.0922(2247)

S = 1.071 Npar= 146

The following ALERTS were generated. Each ALERT has the format
test-name_ALERT_alert-type_alert-level.
Click on the hyperlinks for more details of the test.

Alert level G

```
PLAT142_ALERT_4_G s.u. on b - Axis Small or Missing .............    0.00010 Ang.
PLAT912_ALERT_4_G Missing # of FCF Reflections Above STh/L=  0.600         2 Note
PLAT969_ALERT_5_G The 'Henn et al.' R-Factor-gap value ..........      3.825 Note
              Predicted wR2: Based on SigI**2  2.41 or SHELX Weight  8.61
PLAT978_ALERT_2_G Number C-C Bonds with Positive Residual Density.        11 Info
```

---

0 **ALERT level A** = Most likely a serious problem - resolve or explain
0 **ALERT level B** = A potentially serious problem, consider carefully
0 **ALERT level C** = Check. Ensure it is not caused by an omission or oversight
4 **ALERT level G** = General information/check it is not something unexpected

0 ALERT type 1 CIF construction/syntax error, inconsistent or missing data
1 ALERT type 2 Indicator that the structure model may be wrong or deficient
0 ALERT type 3 Indicator that the structure quality may be low
2 ALERT type 4 Improvement, methodology, query or suggestion
1 ALERT type 5 Informative message, check

It is advisable to attempt to resolve as many as possible of the alerts in all categories. Often the minor alerts point to easily fixed oversights, errors and omissions in your CIF or refinement strategy, so attention to these fine details can be worthwhile. It is up to the individual to critically assess their own results and, if necessary, seek expert advice.

PLATON version of 26/09/2025; check.def file version of 20/09/2025

---

duplicate check

A reduced cell check using CCDC's cellCheckCSD service has found that one or more structures in this CIF are similar to those previously published in the CSD or the ICSD.

DATABLOCK: 1n

- CSD Refcode: XECBOY

  [Cell Parameters for XECBOY: 9.2754,11.9983,18.4996(90,92.772,90)]

DATABLOCK: 1n

- Asiri, , Faidallah, , Ng, S. W. & Tiekink, E. R. T. (2012). *Acta Cryst.* E**68**, o2288-o2288

  [Cell: 9.2754,11.9983,18.4996(90.0000,92.7720,90.0000)]

Datablock 1n - ellipsoid plot

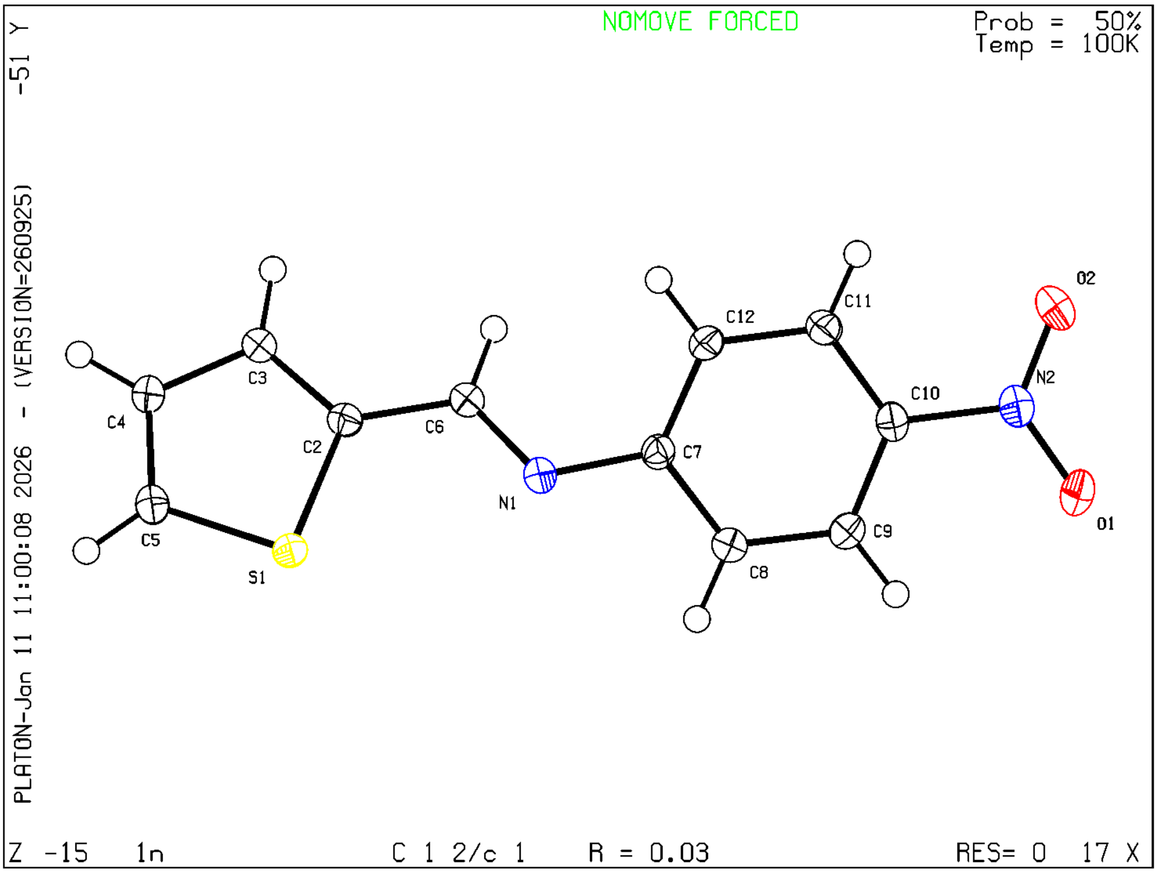

checkCIF/PLATON report

Structure factors have been supplied for datablock(s) 2a

THIS REPORT IS FOR GUIDANCE ONLY. IF USED AS PART OF A REVIEW PROCEDURE FOR PUBLICATION, IT SHOULD NOT REPLACE THE EXPERTISE OF AN EXPERIENCED CRYSTALLOGRAPHIC REFEREE.

No syntax errors found. CIF dictionary Interpreting this report

Datablock: 2a

Bond precision: C-C = 0.0023 A Wavelength=0.71073

Cell:	a=8.8365(3)	b=11.6174(4)	c=13.2729(5)
	alpha=102.941(1)	beta=102.131(1)	gamma=96.388(1)
Temperature:	100 K		

	Calculated	Reported
Volume	1280.25(8)	1280.25(8)
Space group	P -1	P -1
Hall group	-P 1	-P 1
Moiety formula	C21 H22 N5 Ru, F6 P [+ solvent]	C21 H22 N5 Ru, 1(F6 P)
Sum formula	C21 H22 F6 N5 P Ru [+ solvent]	C21.80 H23.20 F6 N5.40 P Ru
Mr	590.48	606.90
Dx,g cm-3	1.532	1.574
Z	2	2
Mu (mm-1)	0.736	0.739
F000	592.0	610.0
F000'	589.99	
h,k,lmax	13,17,20	13,17,20
Nref	9795	9771
Tmin,Tmax	0.699,0.780	0.518,0.566
Tmin'	0.592	

Correction method= # Reported T Limits: Tmin=0.518 Tmax=0.566
AbsCorr = MULTI-SCAN

Data completeness= 0.998   Theta(max)= 33.175

R(reflections)= 0.0288(9354)   wR2(reflections)= 0.0766(9771)

S = 1.054   Npar= 326

The following ALERTS were generated. Each ALERT has the format
test-name_ALERT_alert-type_alert-level.
Click on the hyperlinks for more details of the test.

Alert level C

```
PLAT911_ALERT_3_C Missing FCF Refl Between Thmin & STh/L=    0.600         14 Report
                 1  1  0,   0  3  0,  -1 -2  1,  -1 -1  1,   1 -1  1,   1  0  1,
                -2 -1  2,  -1 -1  2,   0  0  2,  -2  1  2,   1  1  2,   0 -1  3,
                 1 -1  3,   0  0  3,
PLAT913_ALERT_3_C Missing # of Very Strong Reflections in FCF ....          6 Note
                 1  1  0,   0  3  0,  -2 -1  2,  -1 -1  2,   0  0  2,   1 -1  3,
PLAT918_ALERT_3_C Reflection(s) with I(obs) much Smaller I(calc) .          1 Check
                -1  0  1,
```

Alert level G

```
FORMU01_ALERT_1_G  There is a discrepancy between the atom counts in the
            _chemical_formula_sum and _chemical_formula_moiety. This is
            usually due to the moiety formula being in the wrong format.
            Atom count from _chemical_formula_sum:   C21.8 H23.2 F6 N5.4 P1 Ru1
            Atom count from _chemical_formula_moiety:C21 H22 F6 N5 P1 Ru1
FORMU01_ALERT_2_G  There is a discrepancy between the atom counts in the
            _chemical_formula_sum and the formula from the _atom_site* data.
            Atom count from _chemical_formula_sum:C21.8 H23.2 F6 N5.4 P1 Ru1
            Atom count from the _atom_site data:  C21 H22 F6 N5 P1 Ru1
CELLZ01_ALERT_1_G Difference between formula and atom_site contents detected.
CELLZ01_ALERT_1_G ALERT: Large difference may be due to a
            symmetry error - see SYMMG tests
           From the CIF: _cell_formula_units_Z    2
           From the CIF: _chemical_formula_sum  C21.80 H23.20 F6 N5.40 P Ru
           TEST: Compare cell contents of formula and atom_site data

           atom    Z*formula  cif sites diff
           C         43.60     42.00    1.60
           H         46.40     44.00    2.40
           F         12.00     12.00   -0.00
           N         10.80     10.00    0.80
           P          2.00      2.00    0.00
           Ru         2.00      2.00    0.00
PLAT002_ALERT_2_G Number of Distance or Angle Restraints on AtSite         21 Note
PLAT019_ALERT_1_G _diffrn_measured_fraction_theta_full/*_max < 1.0      0.998 Report
PLAT041_ALERT_1_G Calc. and Reported SumFormula    Strings  Differ     Please Check
              Calc: C21 H22 F6 N5 P Ru
```

```
              Rep.: C21.80 H23.20 F6 N5.40 P Ru
PLAT042_ALERT_1_G Calc. and Reported MoietyFormula Strings  Differ     Please Check
              Calc: C21 H22 N5 Ru, F6 P
              Rep.: C21 H22 N5 Ru, 1(F6 P)
PLAT063_ALERT_4_G Crystal Size Possibly too Large for Beam Size ..       0.69 mm
PLAT154_ALERT_1_G The s.u.'s on the Cell Angles are Equal ..(Note)      0.001 Degree
PLAT171_ALERT_4_G The CIF-Embedded .res File Contains EADP Records          2 Report
PLAT176_ALERT_4_G The CIF-Embedded .res File Contains SADI Records          2 Report
PLAT191_ALERT_3_G A Non-default SADI Restraint Value has been used     0.0400 Report
PLAT232_ALERT_2_G Hirshfeld Test Diff (M-X)  Ru1      --N2       .       10.2 s.u.
PLAT232_ALERT_2_G Hirshfeld Test Diff (M-X)  Ru1      --N3       .       11.2 s.u.
PLAT232_ALERT_2_G Hirshfeld Test Diff (M-X)  Ru1      --N4       .       12.6 s.u.
PLAT232_ALERT_2_G Hirshfeld Test Diff (M-X)  Ru1      --N5       .       10.2 s.u.
PLAT302_ALERT_4_G Anion/Solvent/Minor-Residue Disorder (Resd    2)       100% Note
PLAT302_ALERT_4_G Anion/Solvent/Minor-Residue Disorder (Resd    3)       100% Note
PLAT302_ALERT_4_G Anion/Solvent/Minor-Residue Disorder (Resd    4)       100% Note
PLAT304_ALERT_4_G Non-Integer Number of Atoms in ..... (Resd    2)       2.99 Check
PLAT304_ALERT_4_G Non-Integer Number of Atoms in ..... (Resd    3)       2.02 Check
PLAT432_ALERT_2_G Short Inter X...Y Contact  F3A      ..C17      .       2.94 Ang.
                                  1-x,1-y,2-z  =      2_667 Check
PLAT605_ALERT_4_G Largest Solvent Accessible VOID in the Structure        104 A**3
PLAT811_ALERT_5_G No ADDSYM Analysis: Too Many Excluded Atoms ....          ! Info
PLAT860_ALERT_3_G Number of Least-Squares Restraints ............         784 Note
PLAT868_ALERT_4_G ALERTS Due to the Use of _smtbx_masks Suppressed          ! Info
PLAT910_ALERT_3_G Missing FCF Reflection(s) Below Theta(Min)[Deg]=       2.54 Note
                1  0  0,   0  1  0,   0 -1  1,   0  0  1,
PLAT912_ALERT_4_G Missing # of FCF Reflections Above STh/L=  0.600          5 Note
PLAT933_ALERT_2_G Number of HKL-OMIT Records in Embedded .res File          7 Note
               -1 -2  1,  -1 -1  1,   0 -1  3,   0  0  3,   1  0  1,   1  1  0,
                1  1  2,
PLAT969_ALERT_5_G The 'Henn et al.' R-Factor-gap value ..........      4.174 Note
              Predicted wR2: Based on SigI**2  1.84 or SHELX Weight  7.27
PLAT978_ALERT_2_G Number C-C Bonds with Positive Residual Density.          5 Info
```

```
   0 ALERT level A = Most likely a serious problem - resolve or explain
   0 ALERT level B = A potentially serious problem, consider carefully
   3 ALERT level C = Check. Ensure it is not caused by an omission or oversight
  32 ALERT level G = General information/check it is not something unexpected

   7 ALERT type 1 CIF construction/syntax error, inconsistent or missing data
   9 ALERT type 2 Indicator that the structure model may be wrong or deficient
   6 ALERT type 3 Indicator that the structure quality may be low
  11 ALERT type 4 Improvement, methodology, query or suggestion
   2 ALERT type 5 Informative message, check
```

It is advisable to attempt to resolve as many as possible of the alerts in all categories. Often the minor alerts point to easily fixed oversights, errors and omissions in your CIF or refinement strategy, so attention to these fine details can be worthwhile. It is up to the individual to critically assess their own results and, if necessary, seek expert advice.

PLATON version of 26/09/2025; check.def file version of 20/09/2025

duplicate check

No duplication found

Datablock 2a - ellipsoid plot

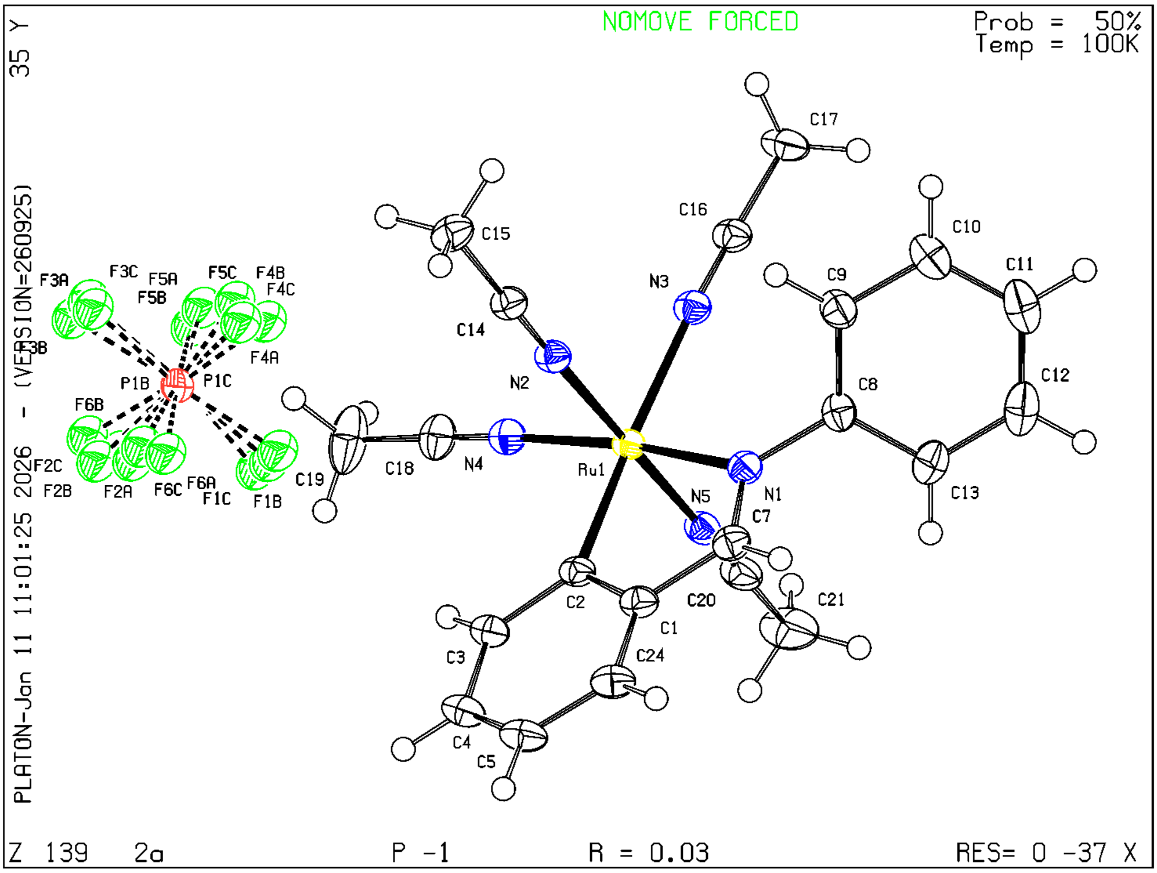

checkCIF/PLATON report

Structure factors have been supplied for datablock(s) 2b

THIS REPORT IS FOR GUIDANCE ONLY. IF USED AS PART OF A REVIEW PROCEDURE FOR PUBLICATION, IT SHOULD NOT REPLACE THE EXPERTISE OF AN EXPERIENCED CRYSTALLOGRAPHIC REFEREE.

No syntax errors found. CIF dictionary Interpreting this report

Datablock: 2b

Bond precision:	C-C = 0.0047 A		Wavelength=1.54184
Cell:	a=8.8149(2)	b=12.0070(2)	c=13.4216(2)
	alpha=104.459(2)	beta=102.197(2)	gamma=99.947(2)
Temperature:	100 K		

	Calculated	Reported
Volume	1305.79(5)	1305.79(4)
Space group	P -1	P -1
Hall group	-P 1	-P 1
Moiety formula	C21 H21 F N5 Ru, F6 P [+ solvent]	C21 H21 F N5 Ru, 1(F6 P)
Sum formula	C21 H21 F7 N5 P Ru [+ solvent]	C22 H22.50 F7 N5.50 P Ru
Mr	608.47	628.99
Dx,g cm-3	1.548	1.600
Z	2	2
Mu (mm-1)	6.070	6.097
F000	608.0	630.0
F000'	610.74	
h,k,lmax	11,15,17	11,15,17
Nref	5716	5649
Tmin,Tmax	0.216,0.401	0.238,0.843
Tmin'	0.095	

Correction method= # Reported T Limits: Tmin=0.238 Tmax=0.843
AbsCorr = GAUSSIAN

Data completeness= 0.988 Theta(max)= 80.124

R(reflections)= 0.0365(5539) wR2(reflections)= 0.0905(5649)

S = 1.036 Npar= 336

The following ALERTS were generated. Each ALERT has the format
test-name_ALERT_alert-type_alert-level.
Click on the hyperlinks for more details of the test.

Alert level C

```
PLAT250_ALERT_2_C Large U3/U1 Ratio for <U(i,j)> Tensor(Resd    1)        2.4 Note
PLAT911_ALERT_3_C Missing FCF Refl Between Thmin & STh/L=    0.600          5 Report
                1 -3 15,   0 -2 15,   1 -2 15,   0 -1 15,   0  0 15,
```

Alert level G

```
FORMU01_ALERT_1_G  There is a discrepancy between the atom counts in the
            _chemical_formula_sum and _chemical_formula_moiety. This is
            usually due to the moiety formula being in the wrong format.
            Atom count from _chemical_formula_sum:   C22 H22.5 F7 N5.5 P1 Ru1
            Atom count from _chemical_formula_moiety:C21 H21 F7 N5 P1 Ru1
FORMU01_ALERT_2_G  There is a discrepancy between the atom counts in the
            _chemical_formula_sum and the formula from the _atom_site* data.
            Atom count from _chemical_formula_sum:C22 H22.5 F7 N5.5 P1 Ru1
            Atom count from the _atom_site data:  C21 H21 F7 N5 P1 Ru1
CELLZ01_ALERT_1_G Difference between formula and atom_site contents detected.
CELLZ01_ALERT_1_G ALERT: Large difference may be due to a
            symmetry error - see SYMMG tests
           From the CIF: _cell_formula_units_Z    2
           From the CIF: _chemical_formula_sum  C22 H22.50 F7 N5.50 P Ru
           TEST: Compare cell contents of formula and atom_site data

           atom    Z*formula  cif sites diff
           C         44.00     42.00    2.00
           H         45.00     42.00    3.00
           F         14.00     14.00    0.00
           N         11.00     10.00    1.00
           P          2.00      2.00    0.00
           Ru         2.00      2.00    0.00
PLAT002_ALERT_2_G Number of Distance or Angle Restraints on AtSite         21 Note
PLAT041_ALERT_1_G Calc. and Reported SumFormula    Strings  Differ     Please Check
             Calc: C21 H21 F7 N5 P Ru
             Rep.: C22 H22.50 F7 N5.50 P Ru
PLAT042_ALERT_1_G Calc. and Reported MoietyFormula Strings  Differ     Please Check
             Calc: C21 H21 F N5 Ru, F6 P
             Rep.: C21 H21 F N5 Ru, 1(F6 P)
PLAT153_ALERT_1_G The s.u.'s on the Cell Axes   are Equal ..(Note)      0.0002 Ang.
PLAT154_ALERT_1_G The s.u.'s on the Cell Angles are Equal ..(Note)       0.002 Degree
```

```
PLAT171_ALERT_4_G The CIF-Embedded .res File Contains EADP Records          2 Report
PLAT176_ALERT_4_G The CIF-Embedded .res File Contains SADI Records         10 Report
PLAT191_ALERT_3_G A Non-default SADI Restraint Value has been used     0.0400 Report
PLAT191_ALERT_3_G A Non-default SADI Restraint Value has been used     0.0400 Report
PLAT191_ALERT_3_G A Non-default SADI Restraint Value has been used     0.0400 Report
PLAT191_ALERT_3_G A Non-default SADI Restraint Value has been used     0.0400 Report
PLAT230_ALERT_2_G Hirshfeld Test Diff for    N2       --C14      .        8.2 s.u.
PLAT230_ALERT_2_G Hirshfeld Test Diff for    N4       --C18      .        5.5 s.u.
PLAT231_ALERT_4_G Hirshfeld Test (Solvent)   P1B      --F2B      .       10.8 s.u.
PLAT231_ALERT_4_G Hirshfeld Test (Solvent)   P1B      --F3B      .       10.4 s.u.
PLAT231_ALERT_4_G Hirshfeld Test (Solvent)   P1B      --F4B      .       10.8 s.u.
PLAT231_ALERT_4_G Hirshfeld Test (Solvent)   P1B      --F5B      .       10.4 s.u.
PLAT231_ALERT_4_G Hirshfeld Test (Solvent)   P1B      --F6B      .       14.2 s.u.
PLAT231_ALERT_4_G Hirshfeld Test (Solvent)   P1B      --F7B      .       14.1 s.u.
PLAT232_ALERT_2_G Hirshfeld Test Diff (M-X)  Ru1      --N1       .        5.4 s.u.
PLAT302_ALERT_4_G Anion/Solvent/Minor-Residue Disorder (Resd    2)       100% Note
PLAT302_ALERT_4_G Anion/Solvent/Minor-Residue Disorder (Resd    3)       100% Note
PLAT302_ALERT_4_G Anion/Solvent/Minor-Residue Disorder (Resd    4)       100% Note
PLAT304_ALERT_4_G Non-Integer Number of Atoms in ..... (Resd    2)       4.12 Check
PLAT304_ALERT_4_G Non-Integer Number of Atoms in ..... (Resd    3)       1.41 Check
PLAT304_ALERT_4_G Non-Integer Number of Atoms in ..... (Resd    4)       1.46 Check
PLAT432_ALERT_2_G Short Inter X...Y Contact  F2C      ..C13      .       2.95 Ang.
                                                  x,-1+y,z  =      1_545 Check
PLAT605_ALERT_4_G Largest Solvent Accessible VOID in the Structure        121 A**3
PLAT811_ALERT_5_G No ADDSYM Analysis: Too Many Excluded Atoms ....          ! Info
PLAT860_ALERT_3_G Number of Least-Squares Restraints ............         172 Note
PLAT868_ALERT_4_G ALERTS Due to the Use of _smtbx_masks Suppressed          ! Info
PLAT912_ALERT_4_G Missing # of FCF Reflections Above STh/L=  0.600         62 Note
PLAT969_ALERT_5_G The 'Henn et al.' R-Factor-gap value ..........       3.138 Note
              Predicted wR2: Based on SigI**2  2.89 or SHELX Weight  8.74
PLAT978_ALERT_2_G Number C-C Bonds with Positive Residual Density.          0 Info
```

```
   0 ALERT level A = Most likely a serious problem - resolve or explain
   0 ALERT level B = A potentially serious problem, consider carefully
   2 ALERT level C = Check. Ensure it is not caused by an omission or oversight
  38 ALERT level G = General information/check it is not something unexpected

   7 ALERT type 1 CIF construction/syntax error, inconsistent or missing data
   8 ALERT type 2 Indicator that the structure model may be wrong or deficient
   6 ALERT type 3 Indicator that the structure quality may be low
  17 ALERT type 4 Improvement, methodology, query or suggestion
   2 ALERT type 5 Informative message, check
```

It is advisable to attempt to resolve as many as possible of the alerts in all categories. Often the minor alerts point to easily fixed oversights, errors and omissions in your CIF or refinement strategy, so attention to these fine details can be worthwhile. It is up to the individual to critically assess their own results and, if necessary, seek expert advice.

PLATON version of 26/09/2025; check.def file version of 20/09/2025

duplicate check

No duplication found

Datablock 2b - ellipsoid plot

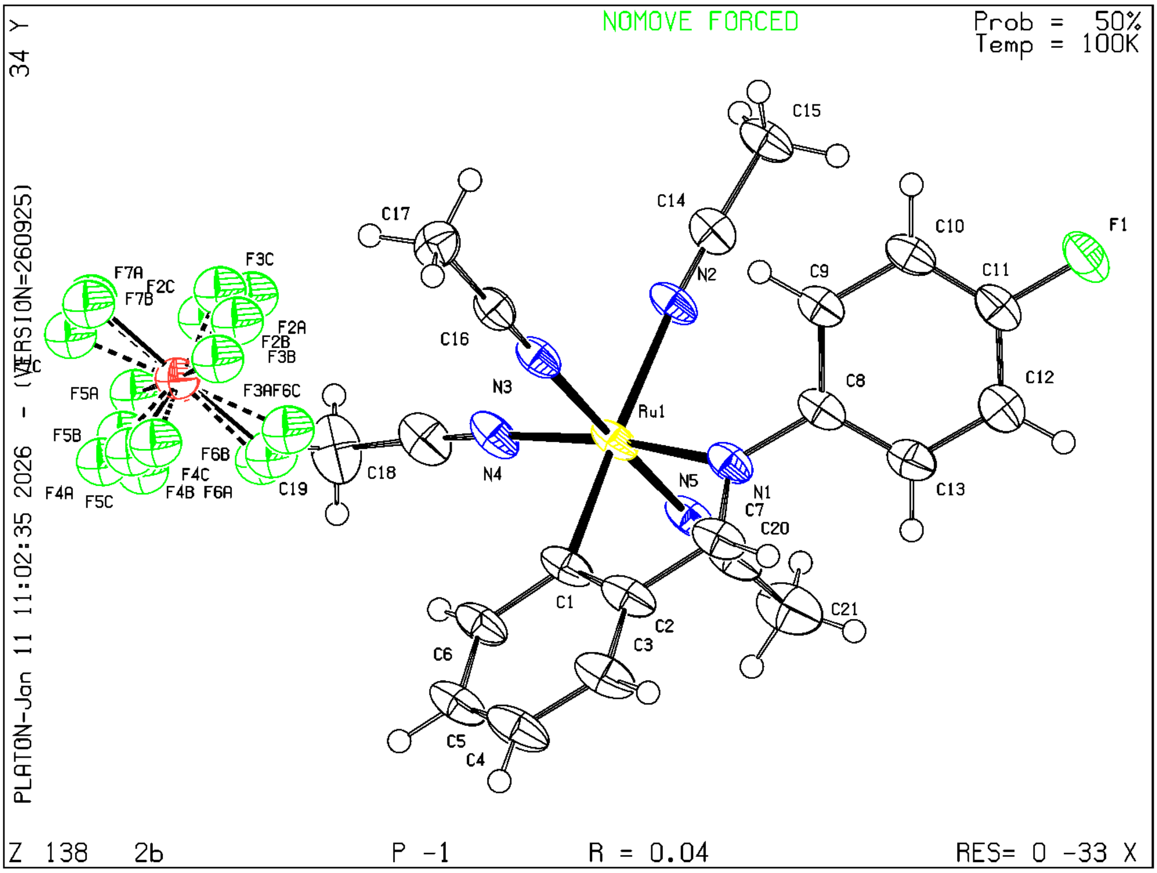

checkCIF/PLATON report

Structure factors have been supplied for datablock(s) 2d

THIS REPORT IS FOR GUIDANCE ONLY. IF USED AS PART OF A REVIEW PROCEDURE FOR PUBLICATION, IT SHOULD NOT REPLACE THE EXPERTISE OF AN EXPERIENCED CRYSTALLOGRAPHIC REFEREE.

No syntax errors found. CIF dictionary Interpreting this report

Datablock: 2d

```
Bond precision:    C-C = 0.0032 A           Wavelength=1.54184

Cell:             a=11.2595(1)     b=21.8514(1)        c=11.7257(1)
                  alpha=90         beta=114.749(1)     gamma=90
Temperature:      100 K

                 Calculated                    Reported
Volume           2619.96(4)                    2619.96(4)
Space group      P 21/c                        P 1 21/c 1
Hall group       -P 2ybc                       -P 2ybc
Moiety formula   C23 H26 N5 Ru, F6 P           C23 H26 N5 Ru, F6 P
Sum formula      C23 H26 F6 N5 P Ru            C23 H26 F6 N5 P Ru
Mr               618.53                        618.53
Dx,g cm-3        1.568                         1.568
Z                4                             4
Mu (mm-1)        6.003                         6.003
F000             1248.0                        1248.0
F000'            1253.33
h,k,lmax         14,27,14                      14,27,14
Nref             5713                          5703
Tmin,Tmax        0.173,0.458                   0.260,1.000
Tmin'            0.040

Correction method= # Reported T Limits: Tmin=0.260 Tmax=1.000
AbsCorr = GAUSSIAN
```

Data completeness= 0.998 Theta(max)= 80.037

R(reflections)= 0.0301(5581) wR2(reflections)= 0.0753(5703)

S = 1.051 Npar= 368

The following ALERTS were generated. Each ALERT has the format
test-name_ALERT_alert-type_alert-level.
Click on the hyperlinks for more details of the test.

Alert level G

```
PLAT002_ALERT_2_G Number of Distance or Angle Restraints on AtSite         28 Note
PLAT142_ALERT_4_G s.u. on b - Axis Small or Missing .............     0.00010 Ang.
PLAT143_ALERT_4_G s.u. on c - Axis Small or Missing .............     0.00010 Ang.
PLAT153_ALERT_1_G The s.u.'s on the Cell Axes   are Equal ..(Note)     0.0001 Ang.
PLAT171_ALERT_4_G The CIF-Embedded .res File Contains EADP Records          2 Report
PLAT174_ALERT_4_G The CIF-Embedded .res File Contains FLAT Records          2 Report
PLAT176_ALERT_4_G The CIF-Embedded .res File Contains SADI Records         48 Report
PLAT191_ALERT_3_G A Non-default SADI Restraint Value has been used     0.0400 Report
PLAT191_ALERT_3_G A Non-default SADI Restraint Value has been used     0.0400 Report
PLAT191_ALERT_3_G A Non-default SADI Restraint Value has been used     0.0400 Report
PLAT191_ALERT_3_G A Non-default SADI Restraint Value has been used     0.0400 Report
PLAT191_ALERT_3_G A Non-default SADI Restraint Value has been used     0.0400 Report
PLAT191_ALERT_3_G A Non-default SADI Restraint Value has been used     0.0400 Report
PLAT191_ALERT_3_G A Non-default SADI Restraint Value has been used     0.0400 Report
PLAT191_ALERT_3_G A Non-default SADI Restraint Value has been used     0.0400 Report
PLAT191_ALERT_3_G A Non-default SADI Restraint Value has been used     0.0400 Report
PLAT191_ALERT_3_G A Non-default SADI Restraint Value has been used     0.0400 Report
PLAT191_ALERT_3_G A Non-default SADI Restraint Value has been used     0.0400 Report
PLAT191_ALERT_3_G A Non-default SADI Restraint Value has been used     0.0400 Report
PLAT191_ALERT_3_G A Non-default SADI Restraint Value has been used     0.0400 Report
PLAT191_ALERT_3_G A Non-default SADI Restraint Value has been used     0.0400 Report
PLAT191_ALERT_3_G A Non-default SADI Restraint Value has been used     0.0400 Report
PLAT191_ALERT_3_G A Non-default SADI Restraint Value has been used     0.0400 Report
PLAT191_ALERT_3_G A Non-default SADI Restraint Value has been used     0.0400 Report
PLAT191_ALERT_3_G A Non-default SADI Restraint Value has been used     0.0400 Report
PLAT191_ALERT_3_G A Non-default SADI Restraint Value has been used     0.0400 Report
PLAT191_ALERT_3_G A Non-default SADI Restraint Value has been used     0.0400 Report
PLAT191_ALERT_3_G A Non-default SADI Restraint Value has been used     0.0400 Report
PLAT191_ALERT_3_G A Non-default SADI Restraint Value has been used     0.0400 Report
PLAT191_ALERT_3_G A Non-default SADI Restraint Value has been used     0.0400 Report
PLAT191_ALERT_3_G A Non-default SADI Restraint Value has been used     0.0400 Report
PLAT191_ALERT_3_G A Non-default SADI Restraint Value has been used     0.0400 Report
PLAT191_ALERT_3_G A Non-default SADI Restraint Value has been used     0.0400 Report
PLAT191_ALERT_3_G A Non-default SADI Restraint Value has been used     0.0400 Report
PLAT191_ALERT_3_G A Non-default SADI Restraint Value has been used     0.0400 Report
PLAT191_ALERT_3_G A Non-default SADI Restraint Value has been used     0.0400 Report
PLAT191_ALERT_3_G A Non-default SADI Restraint Value has been used     0.0400 Report
PLAT230_ALERT_2_G Hirshfeld Test Diff for    N3       --C18      .        5.9 s.u.
PLAT232_ALERT_2_G Hirshfeld Test Diff (M-X)  Ru1      --N2       .        7.1 s.u.
PLAT232_ALERT_2_G Hirshfeld Test Diff (M-X)  Ru1      --N5       .        5.8 s.u.
PLAT302_ALERT_4_G Anion/Solvent/Minor-Residue Disorder (Resd    2)       100% Note
PLAT302_ALERT_4_G Anion/Solvent/Minor-Residue Disorder (Resd    3)       100% Note
```

```
PLAT302_ALERT_4_G Anion/Solvent/Minor-Residue Disorder (Resd    4)       100% Note
PLAT302_ALERT_4_G Anion/Solvent/Minor-Residue Disorder (Resd    5)       100% Note
PLAT304_ALERT_4_G Non-Integer Number of Atoms in ..... (Resd    2)       2.57 Check
PLAT304_ALERT_4_G Non-Integer Number of Atoms in ..... (Resd    3)       1.51 Check
PLAT304_ALERT_4_G Non-Integer Number of Atoms in ..... (Resd    4)       1.72 Check
PLAT304_ALERT_4_G Non-Integer Number of Atoms in ..... (Resd    5)       1.20 Check
PLAT432_ALERT_2_G Short Inter X...Y Contact  F2C      ..C18      .       2.96 Ang.
                                                   1+x,y,z  =      1_655 Check
PLAT432_ALERT_2_G Short Inter X...Y Contact  F5C      ..C17      .       2.94 Ang.
                                                  x,y,-1+z  =      1_554 Check
PLAT432_ALERT_2_G Short Inter X...Y Contact  C16      ..C18      .       3.12 Ang.
                                                1-x,1-y,1-z  =      3_666 Check
PLAT811_ALERT_5_G No ADDSYM Analysis: Too Many Excluded Atoms ....          ! Info
PLAT860_ALERT_3_G Number of Least-Squares Restraints ............         131 Note
PLAT912_ALERT_4_G Missing # of FCF Reflections Above STh/L=  0.600         10 Note
PLAT969_ALERT_5_G The 'Henn et al.' R-Factor-gap value ..........       2.848 Note
              Predicted wR2: Based on SigI**2  2.64 or SHELX Weight  7.16
PLAT978_ALERT_2_G Number C-C Bonds with Positive Residual Density.          1 Info
```

```
  0 ALERT level A = Most likely a serious problem - resolve or explain
  0 ALERT level B = A potentially serious problem, consider carefully
  0 ALERT level C = Check. Ensure it is not caused by an omission or oversight
 56 ALERT level G = General information/check it is not something unexpected

  1 ALERT type 1 CIF construction/syntax error, inconsistent or missing data
  8 ALERT type 2 Indicator that the structure model may be wrong or deficient
 31 ALERT type 3 Indicator that the structure quality may be low
 14 ALERT type 4 Improvement, methodology, query or suggestion
  2 ALERT type 5 Informative message, check
```

It is advisable to attempt to resolve as many as possible of the alerts in all categories. Often the minor alerts point to easily fixed oversights, errors and omissions in your CIF or refinement strategy, so attention to these fine details can be worthwhile. It is up to the individual to critically assess their own results and, if necessary, seek expert advice.

PLATON version of 26/09/2025; check.def file version of 20/09/2025

---

duplicate check

No duplication found

Datablock 2d - ellipsoid plot

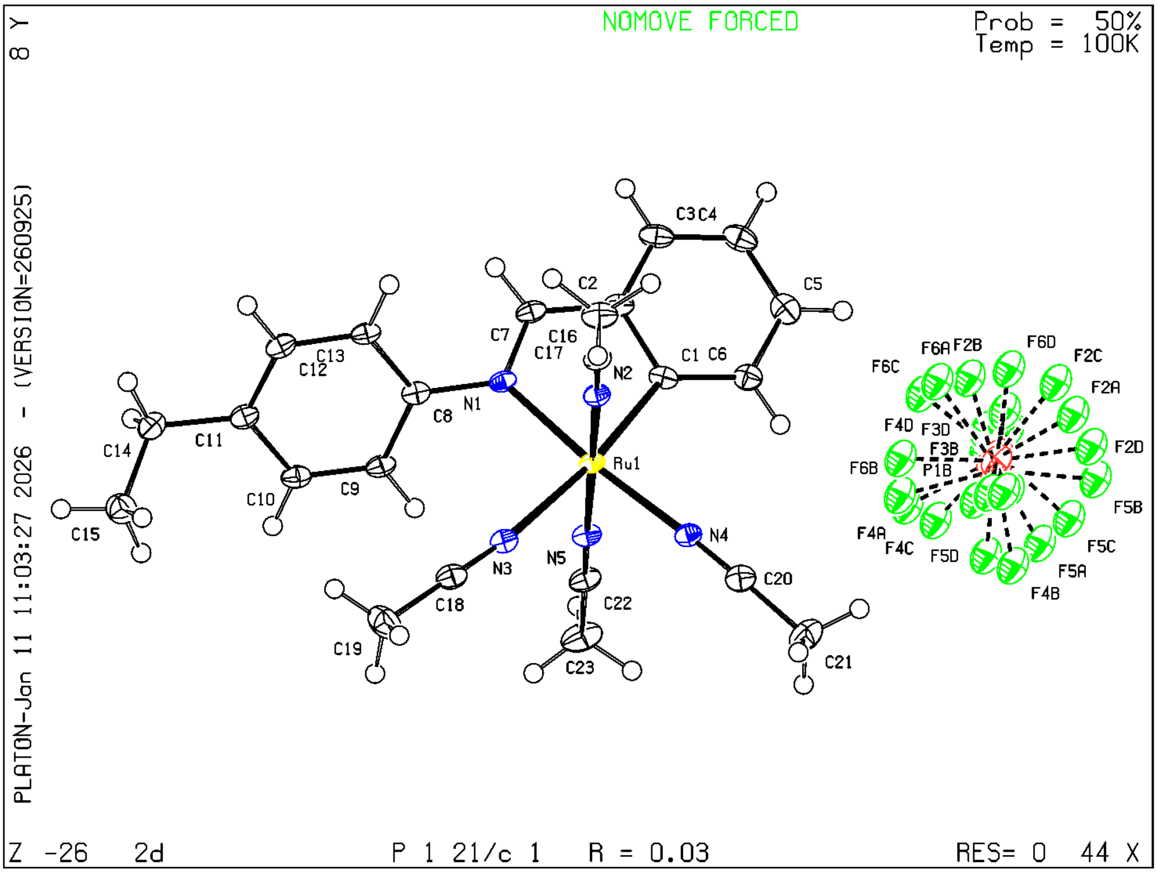

checkCIF/PLATON report

Structure factors have been supplied for datablock(s) 2e

THIS REPORT IS FOR GUIDANCE ONLY. IF USED AS PART OF A REVIEW PROCEDURE FOR PUBLICATION, IT SHOULD NOT REPLACE THE EXPERTISE OF AN EXPERIENCED CRYSTALLOGRAPHIC REFEREE.

No syntax errors found. CIF dictionary Interpreting this report

Datablock: 2e

```
Bond precision:    C-C = 0.0017 A           Wavelength=0.71073

Cell:           a=8.4588(1)       b=12.8083(1)      c=13.4176(1)
                alpha=92.106(1)   beta=101.528(1)   gamma=99.954(1)
Temperature:    100 K

                 Calculated                  Reported
Volume           1399.10(2)                  1399.10(2)
Space group      P -1                        P -1
Hall group       -P 1                        -P 1
Moiety formula   C25 H30 N5 Ru, F6 P         C25 H30 N5 Ru, F6 P
Sum formula      C25 H30 F6 N5 P Ru          C25 H30 F6 N5 P Ru
Mr               646.58                      646.58
Dx,g cm-3        1.535                       1.535
Z                2                           2
Mu (mm-1)        0.681                       0.681
F000             656.0                       656.0
F000'            654.00
h,k,lmax         13,20,21                    13,20,21
Nref             12607                       11863
Tmin,Tmax        0.856,0.947                 0.478,1.000
Tmin'            0.788

Correction method= # Reported T Limits: Tmin=0.478 Tmax=1.000
AbsCorr = GAUSSIAN
```

Data completeness= 0.941   Theta(max)= 35.304

R(reflections)= 0.0272(11024)   wR2(reflections)= 0.0696(11863)

S = 1.041   Npar= 359

The following ALERTS were generated. Each ALERT has the format
test-name_ALERT_alert-type_alert-level.
Click on the hyperlinks for more details of the test.

Alert level C

```
PLAT244_ALERT_4_C Low    'Solvent' Ueq as Compared to Neighbors of         P1 Check
```

Alert level G

```
PLAT002_ALERT_2_G Number of Distance or Angle Restraints on AtSite        10 Note
PLAT153_ALERT_1_G The s.u.'s on the Cell Axes   are Equal ..(Note)     0.0001 Ang.
PLAT154_ALERT_1_G The s.u.'s on the Cell Angles are Equal ..(Note)      0.001 Degree
PLAT171_ALERT_4_G The CIF-Embedded .res File Contains EADP Records          1 Report
PLAT176_ALERT_4_G The CIF-Embedded .res File Contains SADI Records          4 Report
PLAT191_ALERT_3_G A Non-default SADI Restraint Value has been used     0.0400 Report
PLAT230_ALERT_2_G Hirshfeld Test Diff for    C16      --C17      .        5.3 s.u.
PLAT232_ALERT_2_G Hirshfeld Test Diff (M-X)  Ru1      --N2       .       12.5 s.u.
PLAT232_ALERT_2_G Hirshfeld Test Diff (M-X)  Ru1      --N3       .       12.8 s.u.
PLAT232_ALERT_2_G Hirshfeld Test Diff (M-X)  Ru1      --N4       .       12.8 s.u.
PLAT232_ALERT_2_G Hirshfeld Test Diff (M-X)  Ru1      --N5       .       11.5 s.u.
PLAT232_ALERT_2_G Hirshfeld Test Diff (M-X)  Ru1      --C1       .        7.0 s.u.
PLAT301_ALERT_3_G Main Residue  Disorder .............(Resd    1)         10% Note
PLAT412_ALERT_2_G Short Intra XH3 .. XHn     H10      ..H25H     .       2.09 Ang.
                                                      x,y,z  =      1_555 Check
PLAT412_ALERT_2_G Short Intra XH3 .. XHn     H12      ..H24E     .       2.01 Ang.
                                                      x,y,z  =      1_555 Check
PLAT412_ALERT_2_G Short Intra XH3 .. XHn     H12      ..H24H     .       1.83 Ang.
                                                      x,y,z  =      1_555 Check
PLAT413_ALERT_2_G Short Inter XH3 .. XHn     H15A     ..H24I     .       2.12 Ang.
                                                    x,-1+y,z  =     1_545 Check
PLAT860_ALERT_3_G Number of Least-Squares Restraints ............          38 Note
PLAT910_ALERT_3_G Missing FCF Reflection(s) Below Theta(Min)[Deg]=       2.16 Note
                0  1  0,   0  0  1,
PLAT912_ALERT_4_G Missing # of FCF Reflections Above STh/L=  0.600        742 Note
PLAT969_ALERT_5_G The 'Henn et al.' R-Factor-gap value ..........       3.286 Note
              Predicted wR2: Based on SigI**2  2.12 or SHELX Weight  6.69
PLAT978_ALERT_2_G Number C-C Bonds with Positive Residual Density.         13 Info
```

---

0 **ALERT level A** = Most likely a serious problem - resolve or explain
0 **ALERT level B** = A potentially serious problem, consider carefully
1 **ALERT level C** = Check. Ensure it is not caused by an omission or oversight
22 **ALERT level G** = General information/check it is not something unexpected

2 ALERT type 1 CIF construction/syntax error, inconsistent or missing data

```
12 ALERT type 2 Indicator that the structure model may be wrong or deficient
 4 ALERT type 3 Indicator that the structure quality may be low
 4 ALERT type 4 Improvement, methodology, query or suggestion
 1 ALERT type 5 Informative message, check
```

It is advisable to attempt to resolve as many as possible of the alerts in all categories. Often the minor alerts point to easily fixed oversights, errors and omissions in your CIF or refinement strategy, so attention to these fine details can be worthwhile. It is up to the individual to critically assess their own results and, if necessary, seek expert advice.

PLATON version of 26/09/2025; check.def file version of 20/09/2025

---

duplicate check

No duplication found

Datablock 2e - ellipsoid plot

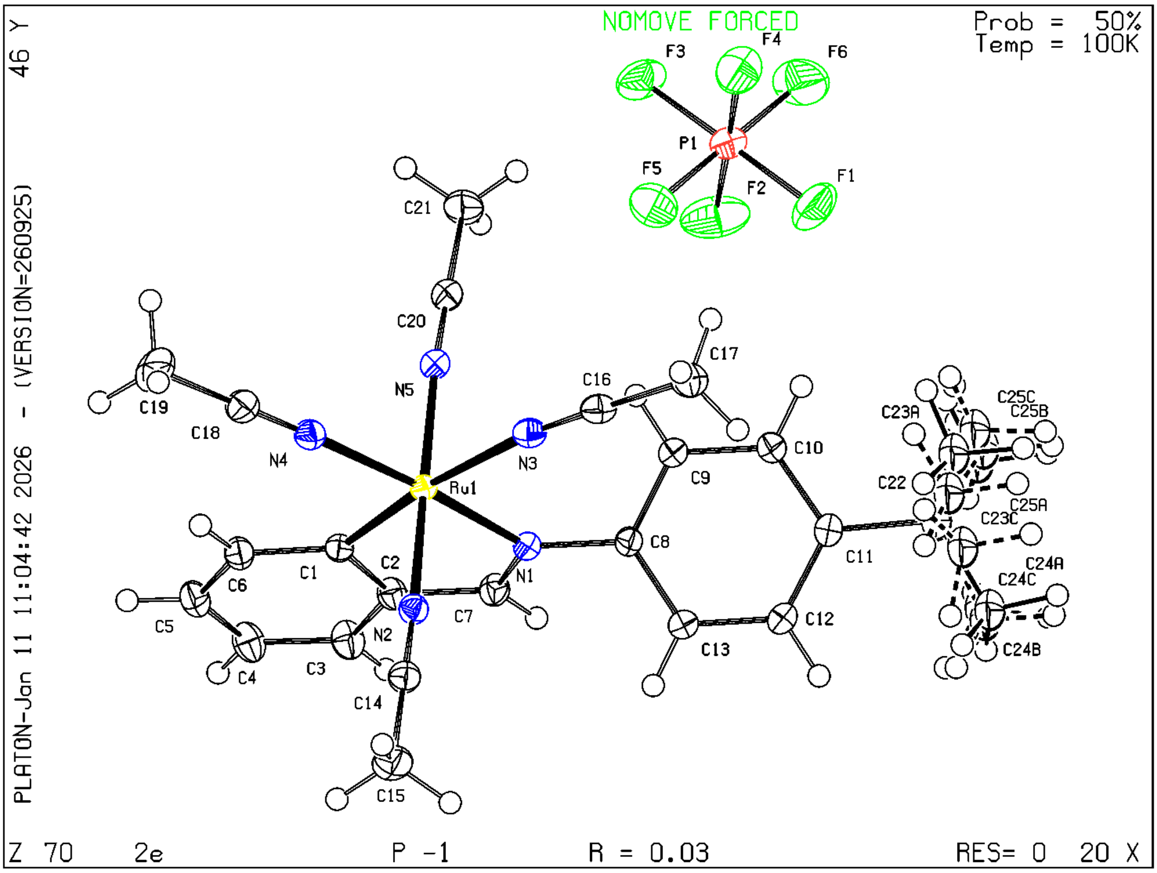

checkCIF/PLATON report

Structure factors have been supplied for datablock(s) 2f

THIS REPORT IS FOR GUIDANCE ONLY. IF USED AS PART OF A REVIEW PROCEDURE FOR PUBLICATION, IT SHOULD NOT REPLACE THE EXPERTISE OF AN EXPERIENCED CRYSTALLOGRAPHIC REFEREE.

No syntax errors found. CIF dictionary Interpreting this report

Datablock: 2f

```
Bond precision:    C-C = 0.0040 A            Wavelength=1.54184

Cell:           a=8.6453(1)        b=11.3212(1)       c=13.8756(2)
                alpha=107.633(1)   beta=103.622(1)    gamma=91.286(1)
Temperature:    100 K

                 Calculated                   Reported
Volume           1251.40(3)                   1251.40(3)
Space group      P -1                         P -1
Hall group       -P 1                         -P 1
Moiety formula   C22 H24 N5 O Ru, F6 P        C22 H24 N5 O Ru, F6 P
Sum formula      C22 H24 F6 N5 O P Ru         C22 H24 F6 N5 O P Ru
Mr               620.50                       620.50
Dx,g cm-3        1.647                        1.647
Z                2                            2
Mu (mm-1)        6.318                        6.318
F000             624.0                        624.0
F000'            626.73
h,k,lmax         11,14,17                     11,14,17
Nref             5483                         5411
Tmin,Tmax        0.045,0.283                  0.026,1.000
Tmin'            0.002

Correction method= # Reported T Limits: Tmin=0.026 Tmax=1.000
AbsCorr = GAUSSIAN
```

Data completeness= 0.987 Theta(max)= 80.408

R(reflections)= 0.0370(5386) wR2(reflections)= 0.0954(5411)

S = 1.110 Npar= 323

The following ALERTS were generated. Each ALERT has the format
test-name_ALERT_alert-type_alert-level.
Click on the hyperlinks for more details of the test.

Alert level C

```
PLAT911_ALERT_3_C Missing FCF Refl Between Thmin & STh/L=    0.600          5 Report
               -9 -2 11,  -9 -1 11,  -9  0 11,  -8 -2 13,  -8 -1 13,
PLAT977_ALERT_2_C Check Negative Difference Density on H22B      .      -0.31 eA-3
```

Alert level G

```
PLAT002_ALERT_2_G Number of Distance or Angle Restraints on AtSite         14 Note
PLAT063_ALERT_4_G Crystal Size Possibly too Large for Beam Size ..       0.88 mm
PLAT154_ALERT_1_G The s.u.'s on the Cell Angles are Equal ..(Note)      0.001 Degree
PLAT171_ALERT_4_G The CIF-Embedded .res File Contains EADP Records          2 Report
PLAT176_ALERT_4_G The CIF-Embedded .res File Contains SADI Records         10 Report
PLAT191_ALERT_3_G A Non-default SADI Restraint Value has been used     0.0400 Report
PLAT191_ALERT_3_G A Non-default SADI Restraint Value has been used     0.0400 Report
PLAT191_ALERT_3_G A Non-default SADI Restraint Value has been used     0.0400 Report
PLAT191_ALERT_3_G A Non-default SADI Restraint Value has been used     0.0400 Report
PLAT231_ALERT_4_G Hirshfeld Test (Solvent)   P1B      --F1B      .       13.9 s.u.
PLAT231_ALERT_4_G Hirshfeld Test (Solvent)   P1B      --F2B      .       17.6 s.u.
PLAT231_ALERT_4_G Hirshfeld Test (Solvent)   P1B      --F3B      .       13.6 s.u.
PLAT231_ALERT_4_G Hirshfeld Test (Solvent)   P1B      --F4B      .       17.3 s.u.
PLAT231_ALERT_4_G Hirshfeld Test (Solvent)   P1B      --F5B      .        9.1 s.u.
PLAT231_ALERT_4_G Hirshfeld Test (Solvent)   P1B      --F6B      .        9.3 s.u.
PLAT232_ALERT_2_G Hirshfeld Test Diff (M-X)  Ru1      --N2       .        6.7 s.u.
PLAT232_ALERT_2_G Hirshfeld Test Diff (M-X)  Ru1      --N5       .        6.1 s.u.
PLAT302_ALERT_4_G Anion/Solvent/Minor-Residue Disorder (Resd    2)       100% Note
PLAT302_ALERT_4_G Anion/Solvent/Minor-Residue Disorder (Resd    3)       100% Note
PLAT304_ALERT_4_G Non-Integer Number of Atoms in ..... (Resd    2)       3.79 Check
PLAT304_ALERT_4_G Non-Integer Number of Atoms in ..... (Resd    3)       3.21 Check
PLAT860_ALERT_3_G Number of Least-Squares Restraints ............          70 Note
PLAT912_ALERT_4_G Missing # of FCF Reflections Above STh/L=  0.600         66 Note
PLAT969_ALERT_5_G The 'Henn et al.' R-Factor-gap value ..........       3.467 Note
              Predicted wR2: Based on SigI**2  2.75 or SHELX Weight  8.60
PLAT978_ALERT_2_G Number C-C Bonds with Positive Residual Density.          0 Info
```

---

0 **ALERT level A** = Most likely a serious problem - resolve or explain
0 **ALERT level B** = A potentially serious problem, consider carefully
2 **ALERT level C** = Check. Ensure it is not caused by an omission or oversight
25 **ALERT level G** = General information/check it is not something unexpected

1 ALERT type 1 CIF construction/syntax error, inconsistent or missing data

```
 5 ALERT type 2 Indicator that the structure model may be wrong or deficient
 6 ALERT type 3 Indicator that the structure quality may be low
14 ALERT type 4 Improvement, methodology, query or suggestion
 1 ALERT type 5 Informative message, check
```

It is advisable to attempt to resolve as many as possible of the alerts in all categories. Often the minor alerts point to easily fixed oversights, errors and omissions in your CIF or refinement strategy, so attention to these fine details can be worthwhile. It is up to the individual to critically assess their own results and, if necessary, seek expert advice.

PLATON version of 26/09/2025; check.def file version of 20/09/2025

---

duplicate check

No duplication found

Datablock 2f - ellipsoid plot

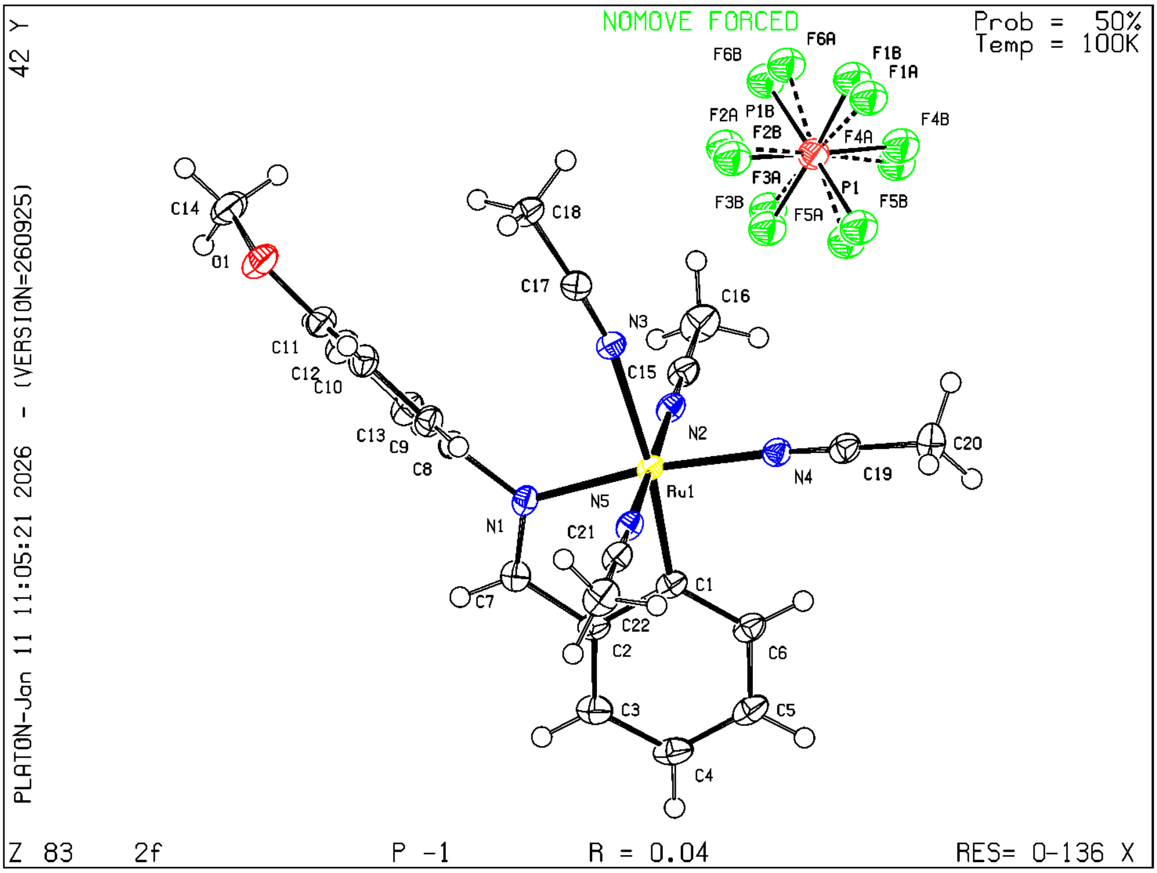

checkCIF/PLATON report

Structure factors have been supplied for datablock(s) 2g

THIS REPORT IS FOR GUIDANCE ONLY. IF USED AS PART OF A REVIEW PROCEDURE FOR PUBLICATION, IT SHOULD NOT REPLACE THE EXPERTISE OF AN EXPERIENCED CRYSTALLOGRAPHIC REFEREE.

No syntax errors found. CIF dictionary Interpreting this report

Datablock: 2g

Bond precision:	C-C = 0.0038 A		Wavelength=1.54184
Cell:	a=8.5446(1)	b=17.2889(1)	c=22.9894(2)
	alpha=105.803(1)	beta=96.587(1)	gamma=101.592(1)
Temperature:	100 K		

	Calculated	Reported
Volume	3148.99(6)	3148.99(5)
Space group	P -1	P -1
Hall group	-P 1	-P 1
Moiety formula	2(C24 H26 N5 O2 Ru), 2(F6 P), C4 H10 O, C2 H3 N	2(C24 H26 N5 O2 Ru), 2(F6 P), C4 H10 O, C2 H3 N
Sum formula	C54 H65 F12 N11 O5 P2 Ru2	C54 H65 F12 N11 O5 P2 Ru2
Mr	1440.40	1440.25
Dx,g cm-3	1.519	1.519
Z	2	2
Mu (mm-1)	5.149	5.148
F000	1464.1	1464.0
F000'	1470.27	
h,k,lmax	10,22,29	10,22,29
Nref	13754	13614
Tmin,Tmax	0.118,0.734	0.102,1.000
Tmin'	0.025	

Correction method= # Reported T Limits: Tmin=0.102 Tmax=1.000
AbsCorr = GAUSSIAN

Data completeness= 0.990 Theta(max)= 80.128

R(reflections)= 0.0344(13295) wR2(reflections)= 0.0888(13614)

S = 1.039 Npar= 834

The following ALERTS were generated. Each ALERT has the format
test-name_ALERT_alert-type_alert-level.
Click on the hyperlinks for more details of the test.

Alert level C

```
PLAT220_ALERT_2_C NonSolvent   Resd 1  C   Ueq(max)/Ueq(min) Range        3.4 Ratio
PLAT911_ALERT_3_C Missing FCF Refl Between Thmin & STh/L=    0.600          9 Report
                0 -4  8,  -9  5 13,  -9  6 13,  -9  7 13,  -9  4 14,  -9  5 14,
               -9  3 15,  -9  4 15,  -8  5 17,
PLAT977_ALERT_2_C Check Negative Difference Density on H40C      .      -0.32 eA-3
```

Alert level G

```
PLAT002_ALERT_2_G Number of Distance or Angle Restraints on AtSite       54 Note
PLAT068_ALERT_1_G Reported F000 Differs from Calcd (or Missing)...    Please Check
PLAT154_ALERT_1_G The s.u.'s on the Cell Angles are Equal ..(Note)     0.001 Degree
PLAT171_ALERT_4_G The CIF-Embedded .res File Contains EADP Records       14 Report
PLAT176_ALERT_4_G The CIF-Embedded .res File Contains SADI Records       72 Report
PLAT191_ALERT_3_G A Non-default SADI Restraint Value has been used    0.0400 Report
PLAT191_ALERT_3_G A Non-default SADI Restraint Value has been used    0.0400 Report
PLAT191_ALERT_3_G A Non-default SADI Restraint Value has been used    0.0400 Report
PLAT191_ALERT_3_G A Non-default SADI Restraint Value has been used    0.0400 Report
PLAT191_ALERT_3_G A Non-default SADI Restraint Value has been used    0.0400 Report
PLAT191_ALERT_3_G A Non-default SADI Restraint Value has been used    0.0400 Report
PLAT191_ALERT_3_G A Non-default SADI Restraint Value has been used    0.0400 Report
PLAT191_ALERT_3_G A Non-default SADI Restraint Value has been used    0.0400 Report
PLAT191_ALERT_3_G A Non-default SADI Restraint Value has been used    0.0400 Report
PLAT191_ALERT_3_G A Non-default SADI Restraint Value has been used    0.0400 Report
PLAT191_ALERT_3_G A Non-default SADI Restraint Value has been used    0.0400 Report
PLAT191_ALERT_3_G A Non-default SADI Restraint Value has been used    0.0400 Report
PLAT191_ALERT_3_G A Non-default SADI Restraint Value has been used    0.0400 Report
PLAT191_ALERT_3_G A Non-default SADI Restraint Value has been used    0.0400 Report
PLAT191_ALERT_3_G A Non-default SADI Restraint Value has been used    0.0400 Report
PLAT191_ALERT_3_G A Non-default SADI Restraint Value has been used    0.0400 Report
PLAT191_ALERT_3_G A Non-default SADI Restraint Value has been used    0.0400 Report
PLAT191_ALERT_3_G A Non-default SADI Restraint Value has been used    0.0400 Report
PLAT191_ALERT_3_G A Non-default SADI Restraint Value has been used    0.0400 Report
PLAT191_ALERT_3_G A Non-default SADI Restraint Value has been used    0.0400 Report
PLAT191_ALERT_3_G A Non-default SADI Restraint Value has been used    0.0400 Report
PLAT191_ALERT_3_G A Non-default SADI Restraint Value has been used    0.0400 Report
PLAT191_ALERT_3_G A Non-default SADI Restraint Value has been used    0.0400 Report
PLAT191_ALERT_3_G A Non-default SADI Restraint Value has been used    0.0400 Report
PLAT191_ALERT_3_G A Non-default SADI Restraint Value has been used    0.0400 Report
PLAT191_ALERT_3_G A Non-default SADI Restraint Value has been used    0.0400 Report
PLAT191_ALERT_3_G A Non-default SADI Restraint Value has been used    0.0400 Report
```

```
PLAT191_ALERT_3_G A Non-default SADI Restraint Value has been used     0.0400 Report
PLAT191_ALERT_3_G A Non-default SADI Restraint Value has been used     0.0400 Report
PLAT191_ALERT_3_G A Non-default SADI Restraint Value has been used     0.0400 Report
PLAT191_ALERT_3_G A Non-default SADI Restraint Value has been used     0.0400 Report
PLAT191_ALERT_3_G A Non-default SADI Restraint Value has been used     0.0400 Report
PLAT191_ALERT_3_G A Non-default SADI Restraint Value has been used     0.0400 Report
PLAT191_ALERT_3_G A Non-default SADI Restraint Value has been used     0.0400 Report
PLAT191_ALERT_3_G A Non-default SADI Restraint Value has been used     0.0400 Report
PLAT191_ALERT_3_G A Non-default SADI Restraint Value has been used     0.0400 Report
PLAT191_ALERT_3_G A Non-default SADI Restraint Value has been used     0.0400 Report
PLAT191_ALERT_3_G A Non-default SADI Restraint Value has been used     0.0400 Report
PLAT191_ALERT_3_G A Non-default SADI Restraint Value has been used     0.0400 Report
PLAT191_ALERT_3_G A Non-default SADI Restraint Value has been used     0.0400 Report
PLAT191_ALERT_3_G A Non-default SADI Restraint Value has been used     0.0400 Report
PLAT191_ALERT_3_G A Non-default SADI Restraint Value has been used     0.0400 Report
PLAT191_ALERT_3_G A Non-default SADI Restraint Value has been used     0.0400 Report
PLAT191_ALERT_3_G A Non-default SADI Restraint Value has been used     0.0400 Report
PLAT191_ALERT_3_G A Non-default SADI Restraint Value has been used     0.0400 Report
PLAT230_ALERT_2_G Hirshfeld Test Diff for    N7       --C41      .        5.1 s.u.
PLAT230_ALERT_2_G Hirshfeld Test Diff for    N10      --C47      .        5.2 s.u.
PLAT230_ALERT_2_G Hirshfeld Test Diff for    O4B      --C38      .        6.0 s.u.
PLAT230_ALERT_2_G Hirshfeld Test Diff for    N3       --C19      .        5.7 s.u.
PLAT231_ALERT_4_G Hirshfeld Test (Solvent)   P2A      --F7A      .       11.5 s.u.
PLAT231_ALERT_4_G Hirshfeld Test (Solvent)   P2A      --F8A      .       17.6 s.u.
PLAT231_ALERT_4_G Hirshfeld Test (Solvent)   P2A      --F9A      .        7.5 s.u.
PLAT231_ALERT_4_G Hirshfeld Test (Solvent)   P2A      --F10A     .       16.6 s.u.
PLAT231_ALERT_4_G Hirshfeld Test (Solvent)   P2A      --F11A     .        9.0 s.u.
PLAT231_ALERT_4_G Hirshfeld Test (Solvent)   P2A      --F12A     .       11.8 s.u.
PLAT232_ALERT_2_G Hirshfeld Test Diff (M-X)  Ru1      --N2       .        5.7 s.u.
PLAT232_ALERT_2_G Hirshfeld Test Diff (M-X)  Ru1      --N5       .        5.4 s.u.
PLAT301_ALERT_3_G Main Residue  Disorder .............(Resd    1)        13% Note
PLAT302_ALERT_4_G Anion/Solvent/Minor-Residue Disorder (Resd    3)       100% Note
PLAT302_ALERT_4_G Anion/Solvent/Minor-Residue Disorder (Resd    4)       100% Note
PLAT302_ALERT_4_G Anion/Solvent/Minor-Residue Disorder (Resd    5)       100% Note
PLAT302_ALERT_4_G Anion/Solvent/Minor-Residue Disorder (Resd    6)       100% Note
PLAT302_ALERT_4_G Anion/Solvent/Minor-Residue Disorder (Resd    7)       100% Note
PLAT302_ALERT_4_G Anion/Solvent/Minor-Residue Disorder (Resd    8)       100% Note
PLAT302_ALERT_4_G Anion/Solvent/Minor-Residue Disorder (Resd    9)       100% Note
PLAT304_ALERT_4_G Non-Integer Number of Atoms in ..... (Resd    3)       3.58 Check
PLAT304_ALERT_4_G Non-Integer Number of Atoms in ..... (Resd    4)       1.82 Check
PLAT304_ALERT_4_G Non-Integer Number of Atoms in ..... (Resd    5)       2.27 Check
PLAT304_ALERT_4_G Non-Integer Number of Atoms in ..... (Resd    6)       2.92 Check
PLAT304_ALERT_4_G Non-Integer Number of Atoms in ..... (Resd    7)       3.42 Check
PLAT304_ALERT_4_G Non-Integer Number of Atoms in ..... (Resd    8)       9.79 Check
PLAT304_ALERT_4_G Non-Integer Number of Atoms in ..... (Resd    9)       5.21 Check
PLAT811_ALERT_5_G No ADDSYM Analysis: Too Many Excluded Atoms ....          ! Info
PLAT860_ALERT_3_G Number of Least-Squares Restraints ............          99 Note
PLAT910_ALERT_3_G Missing FCF Reflection(s) Below Theta(Min)[Deg]=       2.74 Note
                0  0  1,
PLAT912_ALERT_4_G Missing # of FCF Reflections Above STh/L=  0.600        130 Note
PLAT969_ALERT_5_G The 'Henn et al.' R-Factor-gap value ..........       3.422 Note
              Predicted wR2: Based on SigI**2  2.60 or SHELX Weight  8.55
PLAT978_ALERT_2_G Number C-C Bonds with Positive Residual Density.          0 Info
```

---

0 **ALERT level A** = Most likely a serious problem - resolve or explain
0 **ALERT level B** = A potentially serious problem, consider carefully

```
  3 ALERT level C = Check. Ensure it is not caused by an omission or oversight
 85 ALERT level G = General information/check it is not something unexpected

  2 ALERT type 1 CIF construction/syntax error, inconsistent or missing data
 10 ALERT type 2 Indicator that the structure model may be wrong or deficient
 51 ALERT type 3 Indicator that the structure quality may be low
 23 ALERT type 4 Improvement, methodology, query or suggestion
  2 ALERT type 5 Informative message, check
```

It is advisable to attempt to resolve as many as possible of the alerts in all categories. Often the minor alerts point to easily fixed oversights, errors and omissions in your CIF or refinement strategy, so attention to these fine details can be worthwhile. It is up to the individual to critically assess their own results and, if necessary, seek expert advice.

PLATON version of 26/09/2025; check.def file version of 20/09/2025

---

duplicate check

No duplication found

Datablock 2g - ellipsoid plot

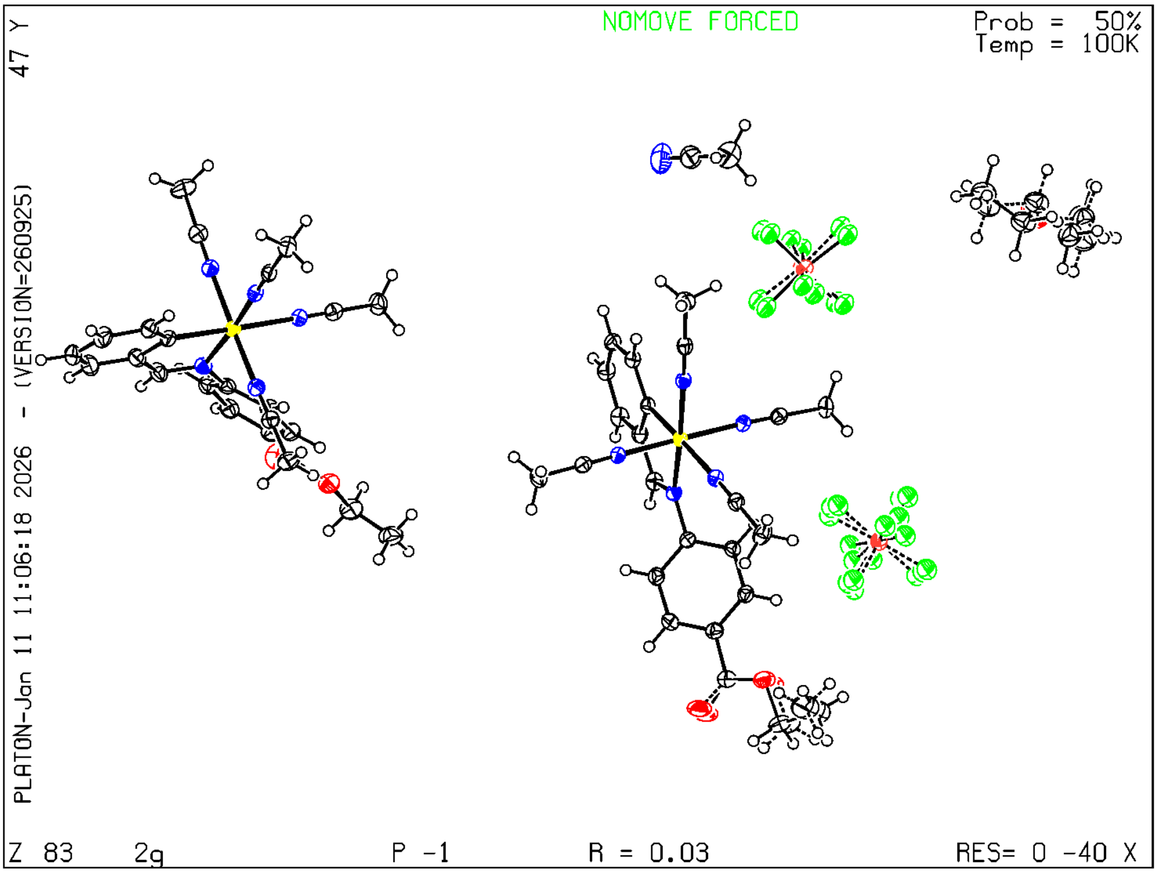

checkCIF/PLATON report

Structure factors have been supplied for datablock(s) 2h

THIS REPORT IS FOR GUIDANCE ONLY. IF USED AS PART OF A REVIEW PROCEDURE FOR PUBLICATION, IT SHOULD NOT REPLACE THE EXPERTISE OF AN EXPERIENCED CRYSTALLOGRAPHIC REFEREE.

No syntax errors found. CIF dictionary Interpreting this report

Datablock: 2h

```
Bond precision:   C-C = 0.0071 A          Wavelength=1.54184

Cell:             a=11.61256(4)    b=8.33269(3)      c=26.25801(10)
                  alpha=90         beta=93.8689(3)   gamma=90
Temperature:      100 K

                  Calculated                     Reported
Volume            2535.037(16)                   2535.036(16)
Space group       P c                            P 1 c 1
Hall group        P -2yc                         P -2yc
Moiety formula    C21 H21 N6 O2 Ru, F6 P         C21 H21 N6 O2 Ru, F6 P
Sum formula       C21 H21 F6 N6 O2 P Ru          C21 H21 F6 N6 O2 P Ru
Mr                635.48                         635.48
Dx,g cm-3         1.665                          1.665
Z                 4                              4
Mu (mm-1)         6.298                          6.298
F000              1272.0                         1272.0
F000'             1277.69
h,k,lmax          14,10,33                       14,10,33
Nref              11094[ 5552]                   10703
Tmin,Tmax         0.244,0.519                    0.190,1.000
Tmin'             0.049

Correction method= # Reported T Limits: Tmin=0.190 Tmax=1.000
AbsCorr = GAUSSIAN
```

Data completeness= 1.93/0.96 Theta(max)= 80.131

R(reflections)= 0.0306(10675) wR2(reflections)= 0.0793(10703)

S = 1.032 Npar= 661

The following ALERTS were generated. Each ALERT has the format
test-name_ALERT_alert-type_alert-level.
Click on the hyperlinks for more details of the test.

Alert level C

```
STRVA01_ALERT_4_C           Flack test results are ambiguous.
           From the CIF: _refine_ls_abs_structure_Flack    0.490
           From the CIF: _refine_ls_abs_structure_Flack_su    0.008
PLAT220_ALERT_2_C NonSolvent   Resd 2  C   Ueq(max)/Ueq(min) Range        3.5 Ratio
```

Alert level G

```
PLAT002_ALERT_2_G Number of Distance or Angle Restraints on AtSite        28 Note
PLAT142_ALERT_4_G s.u. on b - Axis Small or Missing .............    0.00003 Ang.
PLAT143_ALERT_4_G s.u. on c - Axis Small or Missing .............    0.00010 Ang.
PLAT145_ALERT_4_G s.u. on beta     Small or Missing .............     0.0003 Degree
PLAT171_ALERT_4_G The CIF-Embedded .res File Contains EADP Records         4 Report
PLAT176_ALERT_4_G The CIF-Embedded .res File Contains SADI Records        42 Report
PLAT191_ALERT_3_G A Non-default SADI Restraint Value has been used     0.0400 Report
PLAT191_ALERT_3_G A Non-default SADI Restraint Value has been used     0.0400 Report
PLAT191_ALERT_3_G A Non-default SADI Restraint Value has been used     0.0400 Report
PLAT191_ALERT_3_G A Non-default SADI Restraint Value has been used     0.0400 Report
PLAT191_ALERT_3_G A Non-default SADI Restraint Value has been used     0.0400 Report
PLAT191_ALERT_3_G A Non-default SADI Restraint Value has been used     0.0400 Report
PLAT191_ALERT_3_G A Non-default SADI Restraint Value has been used     0.0400 Report
PLAT191_ALERT_3_G A Non-default SADI Restraint Value has been used     0.0400 Report
PLAT191_ALERT_3_G A Non-default SADI Restraint Value has been used     0.0400 Report
PLAT191_ALERT_3_G A Non-default SADI Restraint Value has been used     0.0400 Report
PLAT191_ALERT_3_G A Non-default SADI Restraint Value has been used     0.0400 Report
PLAT191_ALERT_3_G A Non-default SADI Restraint Value has been used     0.0400 Report
PLAT191_ALERT_3_G A Non-default SADI Restraint Value has been used     0.0400 Report
PLAT191_ALERT_3_G A Non-default SADI Restraint Value has been used     0.0400 Report
PLAT191_ALERT_3_G A Non-default SADI Restraint Value has been used     0.0400 Report
PLAT191_ALERT_3_G A Non-default SADI Restraint Value has been used     0.0400 Report
PLAT191_ALERT_3_G A Non-default SADI Restraint Value has been used     0.0400 Report
PLAT191_ALERT_3_G A Non-default SADI Restraint Value has been used     0.0400 Report
PLAT191_ALERT_3_G A Non-default SADI Restraint Value has been used     0.0400 Report
PLAT191_ALERT_3_G A Non-default SADI Restraint Value has been used     0.0400 Report
PLAT191_ALERT_3_G A Non-default SADI Restraint Value has been used     0.0400 Report
PLAT191_ALERT_3_G A Non-default SADI Restraint Value has been used     0.0400 Report
PLAT191_ALERT_3_G A Non-default SADI Restraint Value has been used     0.0400 Report
PLAT191_ALERT_3_G A Non-default SADI Restraint Value has been used     0.0400 Report
PLAT191_ALERT_3_G A Non-default SADI Restraint Value has been used     0.0400 Report
PLAT191_ALERT_3_G A Non-default SADI Restraint Value has been used     0.0400 Report
PLAT191_ALERT_3_G A Non-default SADI Restraint Value has been used     0.0400 Report
PLAT191_ALERT_3_G A Non-default SADI Restraint Value has been used     0.0400 Report
PLAT191_ALERT_3_G A Non-default SADI Restraint Value has been used     0.0400 Report
```

```
PLAT191_ALERT_3_G A Non-default SADI Restraint Value has been used     0.0400 Report
PLAT231_ALERT_4_G Hirshfeld Test (Solvent)   P1A      --F1A      .       16.2 s.u.
PLAT231_ALERT_4_G Hirshfeld Test (Solvent)   P1A      --F2A      .       11.3 s.u.
PLAT231_ALERT_4_G Hirshfeld Test (Solvent)   P1A      --F3A      .       16.3 s.u.
PLAT231_ALERT_4_G Hirshfeld Test (Solvent)   P1A      --F4A      .       11.6 s.u.
PLAT231_ALERT_4_G Hirshfeld Test (Solvent)   P1A      --F5A      .       14.9 s.u.
PLAT231_ALERT_4_G Hirshfeld Test (Solvent)   P1A      --F6A      .       15.0 s.u.
PLAT231_ALERT_4_G Hirshfeld Test (Solvent)   P2A      --F7A      .        6.2 s.u.
PLAT231_ALERT_4_G Hirshfeld Test (Solvent)   P2A      --F8A      .       11.9 s.u.
PLAT231_ALERT_4_G Hirshfeld Test (Solvent)   P2A      --F9A      .        6.3 s.u.
PLAT231_ALERT_4_G Hirshfeld Test (Solvent)   P2A      --F10A     .       11.6 s.u.
PLAT231_ALERT_4_G Hirshfeld Test (Solvent)   P2A      --F11A     .        5.5 s.u.
PLAT231_ALERT_4_G Hirshfeld Test (Solvent)   P2A      --F12A     .        5.6 s.u.
PLAT302_ALERT_4_G Anion/Solvent/Minor-Residue Disorder (Resd    3)       100% Note
PLAT302_ALERT_4_G Anion/Solvent/Minor-Residue Disorder (Resd    4)       100% Note
PLAT302_ALERT_4_G Anion/Solvent/Minor-Residue Disorder (Resd    5)       100% Note
PLAT302_ALERT_4_G Anion/Solvent/Minor-Residue Disorder (Resd    6)       100% Note
PLAT304_ALERT_4_G Non-Integer Number of Atoms in ..... (Resd    3)       5.54 Check
PLAT304_ALERT_4_G Non-Integer Number of Atoms in ..... (Resd    4)       3.54 Check
PLAT304_ALERT_4_G Non-Integer Number of Atoms in ..... (Resd    5)       1.46 Check
PLAT304_ALERT_4_G Non-Integer Number of Atoms in ..... (Resd    6)       3.46 Check
PLAT432_ALERT_2_G Short Inter X...Y Contact  F3B      ..C20      .       2.92 Ang.
                                               x,-1+y,z  =      1_545 Check
PLAT860_ALERT_3_G Number of Least-Squares Restraints ............          44 Note
PLAT883_ALERT_1_G Absent Datum for _atom_sites_solution_primary ..     Please Do !
PLAT912_ALERT_4_G Missing # of FCF Reflections Above STh/L=  0.600          9 Note
PLAT933_ALERT_2_G Number of HKL-OMIT Records in Embedded .res File          1 Note
                1 10  5,
PLAT961_ALERT_5_G Dataset Contains no Negative Intensities ......     Please Check
PLAT969_ALERT_5_G The 'Henn et al.' R-Factor-gap value ..........      3.213 Note
              Predicted wR2: Based on SigI**2  2.47 or SHELX Weight  7.68
PLAT978_ALERT_2_G Number C-C Bonds with Positive Residual Density.          0 Info
```

---

  0 **ALERT level A** = Most likely a serious problem - resolve or explain
  0 **ALERT level B** = A potentially serious problem, consider carefully
  2 **ALERT level C** = Check. Ensure it is not caused by an omission or oversight
 64 **ALERT level G** = General information/check it is not something unexpected

  1 ALERT type 1 CIF construction/syntax error, inconsistent or missing data
  5 ALERT type 2 Indicator that the structure model may be wrong or deficient
 31 ALERT type 3 Indicator that the structure quality may be low
 27 ALERT type 4 Improvement, methodology, query or suggestion
  2 ALERT type 5 Informative message, check

It is advisable to attempt to resolve as many as possible of the alerts in all categories. Often the minor alerts point to easily fixed oversights, errors and omissions in your CIF or refinement strategy, so attention to these fine details can be worthwhile. It is up to the individual to critically assess their own results and, if necessary, seek expert advice.

PLATON version of 26/09/2025; check.def file version of 20/09/2025

duplicate check

No duplication found

Datablock 2h - ellipsoid plot

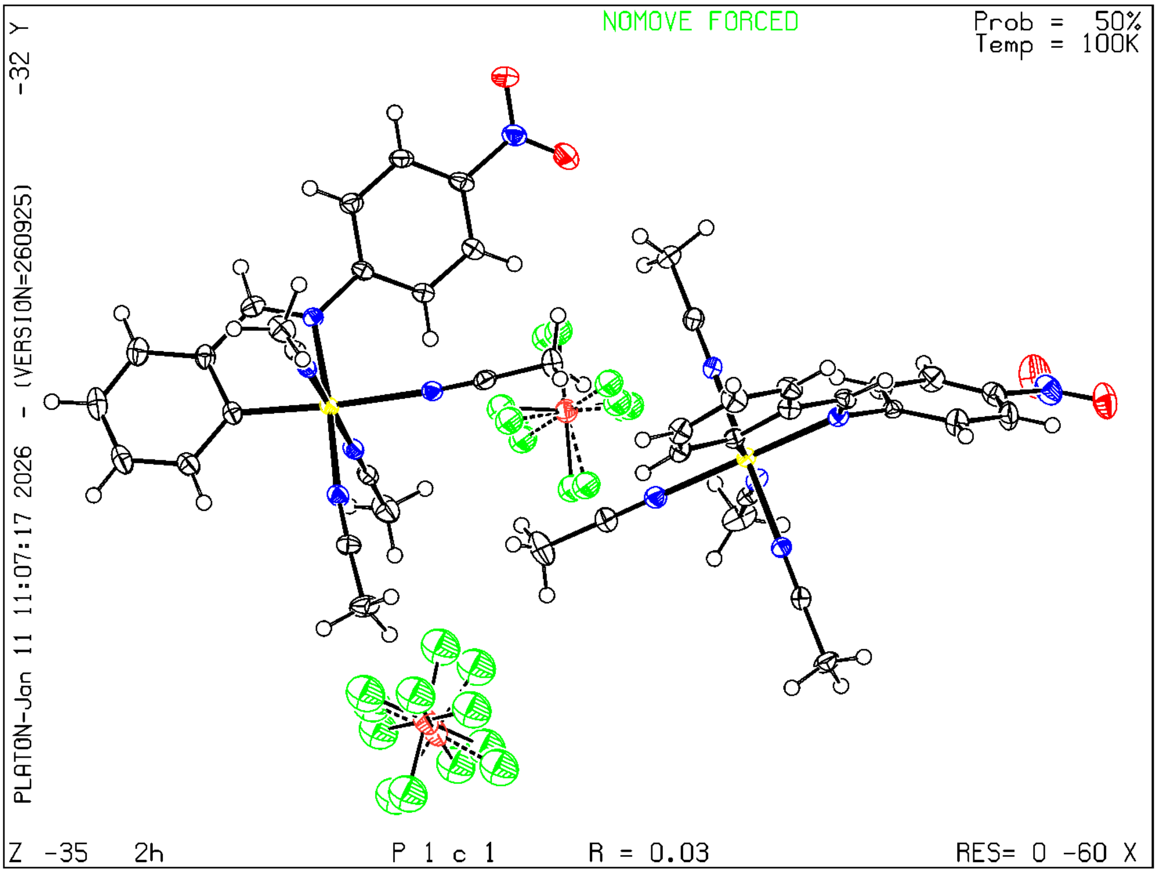

checkCIF/PLATON report

Structure factors have been supplied for datablock(s) 2i

THIS REPORT IS FOR GUIDANCE ONLY. IF USED AS PART OF A REVIEW PROCEDURE FOR PUBLICATION, IT SHOULD NOT REPLACE THE EXPERTISE OF AN EXPERIENCED CRYSTALLOGRAPHIC REFEREE.

No syntax errors found. CIF dictionary Interpreting this report

Datablock: 2i

Bond precision:	C-C = 0.0039 A		Wavelength=1.54184
Cell:	a=10.9455(1)	b=21.5916(1)	c=12.1260(1)
	alpha=90	beta=114.815(1)	gamma=90
Temperature:	100 K		

	Calculated	Reported
Volume	2601.15(4)	2601.15(4)
Space group	P 21/c	P 1 21/c 1
Hall group	-P 2ybc	-P 2ybc
Moiety formula	C22 H21 F3 N5 Ru, F6 P	C22 H21 F3 N5 Ru, F6 P
Sum formula	C22 H21 F9 N5 P Ru	C22 H21 F9 N5 P Ru
Mr	658.48	658.48
Dx,g cm-3	1.681	1.681
Z	4	4
Mu (mm-1)	6.262	6.262
F000	1312.0	1312.0
F000'	1318.13	
h,k,lmax	13,27,15	13,27,15
Nref	5672	5662
Tmin,Tmax	0.216,0.367	0.393,0.998
Tmin'	0.139	

Correction method= # Reported T Limits: Tmin=0.393 Tmax=0.998
AbsCorr = GAUSSIAN

Data completeness= 0.998 Theta(max)= 80.049

R(reflections)= 0.0338(5633) wR2(reflections)= 0.0797(5662)

S = 1.053 Npar= 338

The following ALERTS were generated. Each ALERT has the format
test-name_ALERT_alert-type_alert-level.
Click on the hyperlinks for more details of the test.

Alert level G

```
PLAT002_ALERT_2_G Number of Distance or Angle Restraints on AtSite         21 Note
PLAT083_ALERT_2_G SHELXL Second Parameter in WGHT  Unusually Large       6.59 Why ?
PLAT142_ALERT_4_G s.u. on b - Axis Small or Missing .............     0.00010 Ang.
PLAT143_ALERT_4_G s.u. on c - Axis Small or Missing .............     0.00010 Ang.
PLAT153_ALERT_1_G The s.u.'s on the Cell Axes   are Equal ..(Note)     0.0001 Ang.
PLAT171_ALERT_4_G The CIF-Embedded .res File Contains EADP Records          4 Report
PLAT176_ALERT_4_G The CIF-Embedded .res File Contains SADI Records         24 Report
PLAT191_ALERT_3_G A Non-default SADI Restraint Value has been used     0.0400 Report
PLAT191_ALERT_3_G A Non-default SADI Restraint Value has been used     0.0400 Report
PLAT191_ALERT_3_G A Non-default SADI Restraint Value has been used     0.0400 Report
PLAT191_ALERT_3_G A Non-default SADI Restraint Value has been used     0.0400 Report
PLAT191_ALERT_3_G A Non-default SADI Restraint Value has been used     0.0400 Report
PLAT191_ALERT_3_G A Non-default SADI Restraint Value has been used     0.0400 Report
PLAT191_ALERT_3_G A Non-default SADI Restraint Value has been used     0.0400 Report
PLAT191_ALERT_3_G A Non-default SADI Restraint Value has been used     0.0400 Report
PLAT191_ALERT_3_G A Non-default SADI Restraint Value has been used     0.0400 Report
PLAT191_ALERT_3_G A Non-default SADI Restraint Value has been used     0.0400 Report
PLAT191_ALERT_3_G A Non-default SADI Restraint Value has been used     0.0400 Report
PLAT191_ALERT_3_G A Non-default SADI Restraint Value has been used     0.0400 Report
PLAT191_ALERT_3_G A Non-default SADI Restraint Value has been used     0.0400 Report
PLAT191_ALERT_3_G A Non-default SADI Restraint Value has been used     0.0400 Report
PLAT231_ALERT_4_G Hirshfeld Test (Solvent)   P0       --F4B      .       21.7 s.u.
PLAT231_ALERT_4_G Hirshfeld Test (Solvent)   P0       --F5B      .       13.0 s.u.
PLAT231_ALERT_4_G Hirshfeld Test (Solvent)   P0       --F6B      .       21.3 s.u.
PLAT231_ALERT_4_G Hirshfeld Test (Solvent)   P0       --F7B      .       12.8 s.u.
PLAT231_ALERT_4_G Hirshfeld Test (Solvent)   P0       --F8B      .       15.2 s.u.
PLAT231_ALERT_4_G Hirshfeld Test (Solvent)   P0       --F9B      .       15.4 s.u.
PLAT232_ALERT_2_G Hirshfeld Test Diff (M-X)  Ru1      --N3       .        5.9 s.u.
PLAT232_ALERT_2_G Hirshfeld Test Diff (M-X)  Ru1      --N5       .        5.2 s.u.
PLAT301_ALERT_3_G Main Residue  Disorder .............(Resd    1)         10% Note
PLAT302_ALERT_4_G Anion/Solvent/Minor-Residue Disorder (Resd    2)       100% Note
PLAT302_ALERT_4_G Anion/Solvent/Minor-Residue Disorder (Resd    3)       100% Note
PLAT304_ALERT_4_G Non-Integer Number of Atoms in ..... (Resd    2)       3.89 Check
PLAT304_ALERT_4_G Non-Integer Number of Atoms in ..... (Resd    3)       3.11 Check
PLAT432_ALERT_2_G Short Inter X...Y Contact  C15      ..C17      .       3.07 Ang.
                                               1-x,1-y,1-z  =     3_666 Check
PLAT432_ALERT_2_G Short Inter X...Y Contact  C17      ..C19      .       3.16 Ang.
                                               1-x,1-y,1-z  =     3_666 Check
PLAT720_ALERT_4_G Number of Unusual/Non-Standard Labels .........          1 Note
              P0
PLAT811_ALERT_5_G No ADDSYM Analysis: Too Many Excluded Atoms ....          ! Info
```

```
PLAT860_ALERT_3_G Number of Least-Squares Restraints .............         24 Note
PLAT912_ALERT_4_G Missing # of FCF Reflections Above STh/L=  0.600          9 Note
PLAT961_ALERT_5_G Dataset Contains no Negative Intensities .......     Please Check
PLAT969_ALERT_5_G The 'Henn et al.' R-Factor-gap value ...........      3.458 Note
              Predicted wR2: Based on SigI**2  2.30 or SHELX Weight  7.57
PLAT978_ALERT_2_G Number C-C Bonds with Positive Residual Density.          0 Info
```

```
   0 ALERT level A = Most likely a serious problem - resolve or explain
   0 ALERT level B = A potentially serious problem, consider carefully
   0 ALERT level C = Check. Ensure it is not caused by an omission or oversight
  44 ALERT level G = General information/check it is not something unexpected

   1 ALERT type 1 CIF construction/syntax error, inconsistent or missing data
   7 ALERT type 2 Indicator that the structure model may be wrong or deficient
  17 ALERT type 3 Indicator that the structure quality may be low
  16 ALERT type 4 Improvement, methodology, query or suggestion
   3 ALERT type 5 Informative message, check
```

It is advisable to attempt to resolve as many as possible of the alerts in all categories. Often the minor alerts point to easily fixed oversights, errors and omissions in your CIF or refinement strategy, so attention to these fine details can be worthwhile. It is up to the individual to critically assess their own results and, if necessary, seek expert advice.

PLATON version of 26/09/2025; check.def file version of 20/09/2025

---

duplicate check

No duplication found

Datablock 2i - ellipsoid plot

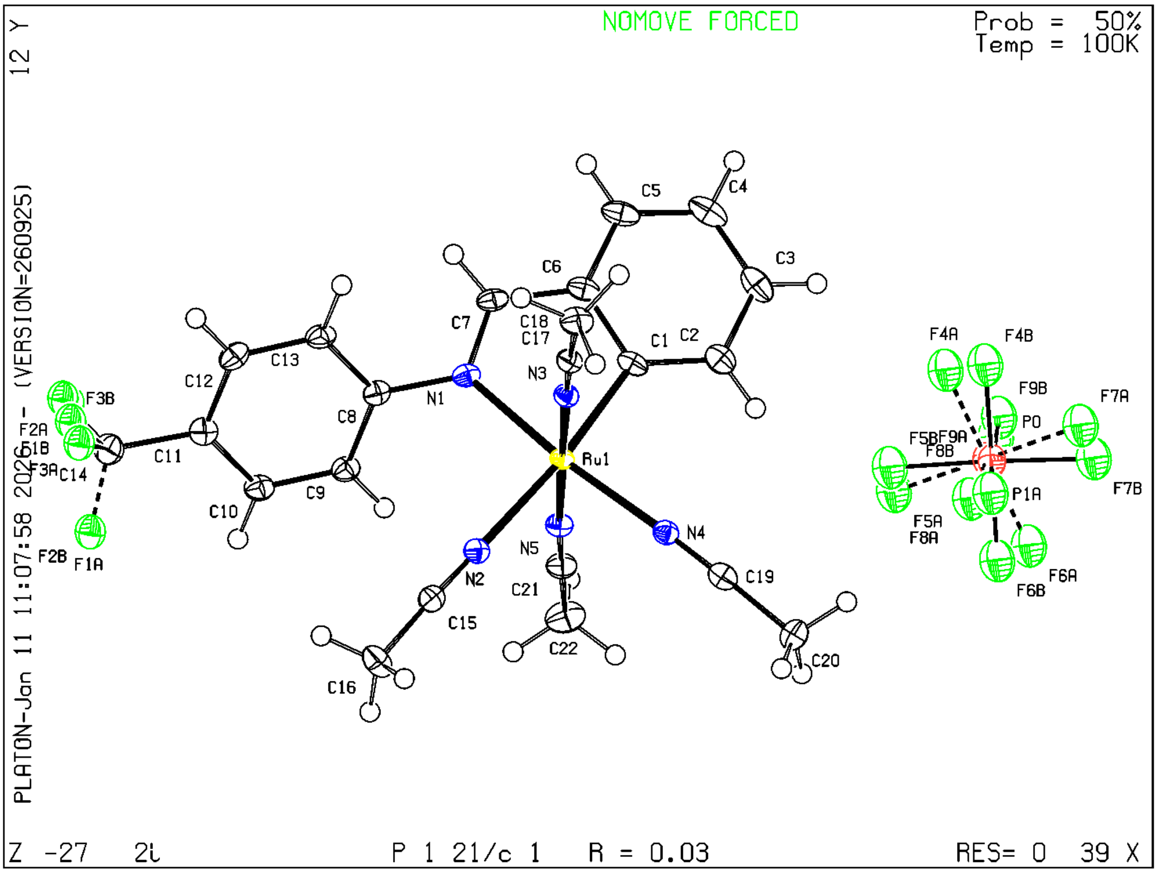

checkCIF/PLATON report

Structure factors have been supplied for datablock(s) 2j

THIS REPORT IS FOR GUIDANCE ONLY. IF USED AS PART OF A REVIEW PROCEDURE FOR PUBLICATION, IT SHOULD NOT REPLACE THE EXPERTISE OF AN EXPERIENCED CRYSTALLOGRAPHIC REFEREE.

No syntax errors found. CIF dictionary Interpreting this report

Datablock: 2j

```
Bond precision:   C-C = 0.0035 A          Wavelength=1.54184

Cell:            a=8.4438(1)        b=11.2103(1)       c=13.8196(2)
                 alpha=103.994(1)   beta=107.503(1)    gamma=95.779(1)
Temperature:     100 K

                  Calculated                  Reported
Volume            1189.09(3)                  1189.09(3)
Space group       P -1                        P -1
Hall group        -P 1                        -P 1
Moiety formula    C19 H19 F N5 Ru S, F6 P     C19 H19 F N5 Ru S, F6 P
Sum formula       C19 H19 F7 N5 P Ru S        C19 H19 F7 N5 P Ru S
Mr                614.49                      614.49
Dx,g cm-3         1.716                       1.716
Z                 2                           2
Mu (mm-1)         7.472                       7.472
F000              612.0                       612.0
F000'             615.36
h,k,lmax          10,14,17                    10,14,17
Nref              5194                        5148
Tmin,Tmax         0.281,0.344                 0.252,1.000
Tmin'             0.038

Correction method= # Reported T Limits: Tmin=0.252 Tmax=1.000
AbsCorr = GAUSSIAN
```

Data completeness= 0.991 Theta(max)= 80.096

R(reflections)= 0.0274(5098) wR2(reflections)= 0.0716(5148)

S = 1.081 Npar= 357

The following ALERTS were generated. Each ALERT has the format
test-name_ALERT_alert-type_alert-level.
Click on the hyperlinks for more details of the test.

Alert level C

```
PLAT911_ALERT_3_C Missing FCF Refl Between Thmin & STh/L=    0.600          5 Report
                3 -3 13,   3 -2 13,   3 -1 13,   2 -2 14,   2 -1 14,
```

Alert level G

```
PLAT002_ALERT_2_G Number of Distance or Angle Restraints on AtSite         21 Note
PLAT154_ALERT_1_G The s.u.'s on the Cell Angles are Equal ..(Note)      0.001 Degree
PLAT171_ALERT_4_G The CIF-Embedded .res File Contains EADP Records          7 Report
PLAT176_ALERT_4_G The CIF-Embedded .res File Contains SADI Records         18 Report
PLAT191_ALERT_3_G A Non-default SADI Restraint Value has been used     0.0400 Report
PLAT191_ALERT_3_G A Non-default SADI Restraint Value has been used     0.0400 Report
PLAT191_ALERT_3_G A Non-default SADI Restraint Value has been used     0.0400 Report
PLAT191_ALERT_3_G A Non-default SADI Restraint Value has been used     0.0400 Report
PLAT191_ALERT_3_G A Non-default SADI Restraint Value has been used     0.0400 Report
PLAT191_ALERT_3_G A Non-default SADI Restraint Value has been used     0.0400 Report
PLAT191_ALERT_3_G A Non-default SADI Restraint Value has been used     0.0400 Report
PLAT191_ALERT_3_G A Non-default SADI Restraint Value has been used     0.0400 Report
PLAT191_ALERT_3_G A Non-default SADI Restraint Value has been used     0.0400 Report
PLAT191_ALERT_3_G A Non-default SADI Restraint Value has been used     0.0400 Report
PLAT191_ALERT_3_G A Non-default SADI Restraint Value has been used     0.0400 Report
PLAT191_ALERT_3_G A Non-default SADI Restraint Value has been used     0.0400 Report
PLAT232_ALERT_2_G Hirshfeld Test Diff (M-X)  Ru1      --N3       .        6.7 s.u.
PLAT232_ALERT_2_G Hirshfeld Test Diff (M-X)  Ru1      --N4       .        7.3 s.u.
PLAT232_ALERT_2_G Hirshfeld Test Diff (M-X)  Ru1      --N5       .        6.0 s.u.
PLAT302_ALERT_4_G Anion/Solvent/Minor-Residue Disorder (Resd    2)       100% Note
PLAT302_ALERT_4_G Anion/Solvent/Minor-Residue Disorder (Resd    3)       100% Note
PLAT302_ALERT_4_G Anion/Solvent/Minor-Residue Disorder (Resd    4)       100% Note
PLAT304_ALERT_4_G Non-Integer Number of Atoms in ..... (Resd    2)       5.27 Check
PLAT304_ALERT_4_G Non-Integer Number of Atoms in ..... (Resd    3)       1.36 Check
PLAT304_ALERT_4_G Non-Integer Number of Atoms in ..... (Resd    4)       0.37 Check
PLAT432_ALERT_2_G Short Inter X...Y Contact  F4C      ..C13      .       2.84 Ang.
                                                     x,y,z  =     1_555 Check
PLAT432_ALERT_2_G Short Inter X...Y Contact  F4C      ..C14      .       2.95 Ang.
                                                     x,y,z  =     1_555 Check
PLAT432_ALERT_2_G Short Inter X...Y Contact  F6C      ..C20      .       2.78 Ang.
                                                1-x,-y,1-z  =     2_656 Check
PLAT811_ALERT_5_G No ADDSYM Analysis: Too Many Excluded Atoms ....          ! Info
PLAT860_ALERT_3_G Number of Least-Squares Restraints ............          55 Note
PLAT912_ALERT_4_G Missing # of FCF Reflections Above STh/L=  0.600         41 Note
PLAT969_ALERT_5_G The 'Henn et al.' R-Factor-gap value ..........       2.730 Note
              Predicted wR2: Based on SigI**2  2.62 or SHELX Weight  6.62
PLAT978_ALERT_2_G Number C-C Bonds with Positive Residual Density.          4 Info
```

```
  0 ALERT level A = Most likely a serious problem - resolve or explain
  0 ALERT level B = A potentially serious problem, consider carefully
  1 ALERT level C = Check. Ensure it is not caused by an omission or oversight
 33 ALERT level G = General information/check it is not something unexpected

  1 ALERT type 1 CIF construction/syntax error, inconsistent or missing data
  8 ALERT type 2 Indicator that the structure model may be wrong or deficient
 14 ALERT type 3 Indicator that the structure quality may be low
  9 ALERT type 4 Improvement, methodology, query or suggestion
  2 ALERT type 5 Informative message, check
```

It is advisable to attempt to resolve as many as possible of the alerts in all categories. Often the minor alerts point to easily fixed oversights, errors and omissions in your CIF or refinement strategy, so attention to these fine details can be worthwhile. It is up to the individual to critically assess their own results and, if necessary, seek expert advice.

PLATON version of 26/09/2025; check.def file version of 20/09/2025

duplicate check

No duplication found

Datablock 2j - ellipsoid plot

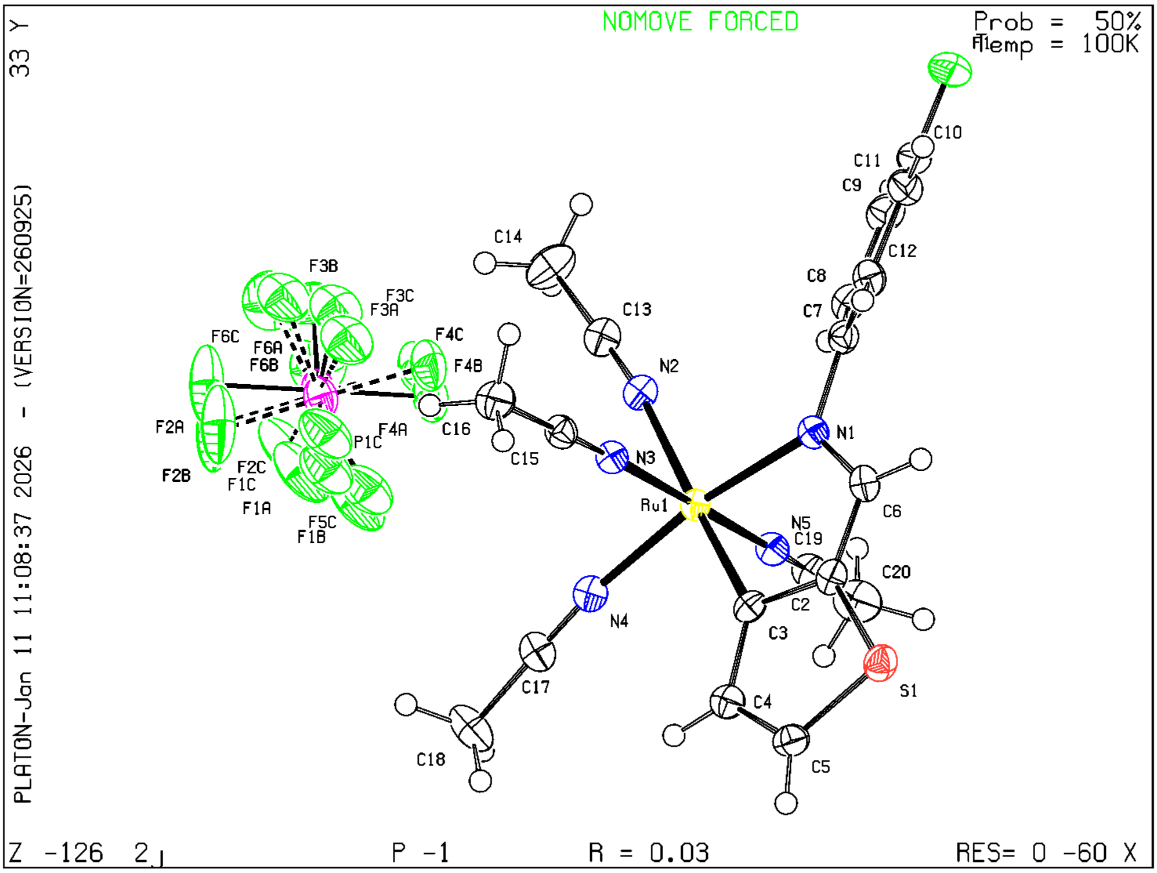

checkCIF/PLATON report

Structure factors have been supplied for datablock(s) 2k

THIS REPORT IS FOR GUIDANCE ONLY. IF USED AS PART OF A REVIEW PROCEDURE FOR PUBLICATION, IT SHOULD NOT REPLACE THE EXPERTISE OF AN EXPERIENCED CRYSTALLOGRAPHIC REFEREE.

No syntax errors found. CIF dictionary Interpreting this report

Datablock: 2k

```
Bond precision:   C-C = 0.0045 A          Wavelength=1.54184

Cell:           a=8.4940(1)        b=11.8298(1)       c=13.8596(1)
                alpha=107.103(1)   beta=107.578(1)    gamma=97.561(1)
Temperature:    100 K

                 Calculated                    Reported
Volume           1231.23(2)                    1231.22(2)
Space group      P -1                          P -1
Hall group       -P 1                          -P 1
Moiety formula   C20 H22 N5 Ru S, F6 P         C20 H22 N5 Ru S, F6 P
Sum formula      C20 H22 F6 N5 P Ru S          C20 H22 F6 N5 P Ru S
Mr               610.53                        610.52
Dx,g cm-3        1.647                         1.647
Z                2                             2
Mu (mm-1)        7.150                         7.150
F000             612.0                         612.0
F000'            615.24
h,k,lmax         10,15,17                      10,15,17
Nref             5383                          5330
Tmin,Tmax        0.390,0.824                   0.242,1.000
Tmin'            0.091

Correction method= # Reported T Limits: Tmin=0.242 Tmax=1.000
AbsCorr = GAUSSIAN
```

Data completeness= 0.990   Theta(max)= 80.030

R(reflections)= 0.0318(5222)   wR2(reflections)= 0.0809(5330)

S = 1.078   Npar= 350

The following ALERTS were generated. Each ALERT has the format
test-name_ALERT_alert-type_alert-level.
Click on the hyperlinks for more details of the test.

Alert level C

```
PLAT911_ALERT_3_C Missing FCF Refl Between Thmin & STh/L=    0.600          9 Report
            -10  2  2, -10  3  2, -10  2  3, -10  3  3, -10  4  3, -10  2  4,
            -10  3  4, -10  4  4, -10  3  5,
```

Alert level G

```
PLAT002_ALERT_2_G Number of Distance or Angle Restraints on AtSite         28 Note
PLAT153_ALERT_1_G The s.u.'s on the Cell Axes   are Equal ..(Note)     0.0001 Ang.
PLAT154_ALERT_1_G The s.u.'s on the Cell Angles are Equal ..(Note)      0.001 Degree
PLAT171_ALERT_4_G The CIF-Embedded .res File Contains EADP Records          2 Report
PLAT176_ALERT_4_G The CIF-Embedded .res File Contains SADI Records         63 Report
PLAT191_ALERT_3_G A Non-default SADI Restraint Value has been used     0.0400 Report
PLAT191_ALERT_3_G A Non-default SADI Restraint Value has been used     0.0400 Report
PLAT191_ALERT_3_G A Non-default SADI Restraint Value has been used     0.0400 Report
PLAT191_ALERT_3_G A Non-default SADI Restraint Value has been used     0.0400 Report
PLAT191_ALERT_3_G A Non-default SADI Restraint Value has been used     0.0400 Report
PLAT191_ALERT_3_G A Non-default SADI Restraint Value has been used     0.0400 Report
PLAT191_ALERT_3_G A Non-default SADI Restraint Value has been used     0.0400 Report
PLAT191_ALERT_3_G A Non-default SADI Restraint Value has been used     0.0400 Report
PLAT191_ALERT_3_G A Non-default SADI Restraint Value has been used     0.0400 Report
PLAT191_ALERT_3_G A Non-default SADI Restraint Value has been used     0.0400 Report
PLAT191_ALERT_3_G A Non-default SADI Restraint Value has been used     0.0400 Report
PLAT191_ALERT_3_G A Non-default SADI Restraint Value has been used     0.0400 Report
PLAT191_ALERT_3_G A Non-default SADI Restraint Value has been used     0.0400 Report
PLAT191_ALERT_3_G A Non-default SADI Restraint Value has been used     0.0400 Report
PLAT191_ALERT_3_G A Non-default SADI Restraint Value has been used     0.0400 Report
PLAT191_ALERT_3_G A Non-default SADI Restraint Value has been used     0.0400 Report
PLAT191_ALERT_3_G A Non-default SADI Restraint Value has been used     0.0400 Report
PLAT191_ALERT_3_G A Non-default SADI Restraint Value has been used     0.0400 Report
PLAT191_ALERT_3_G A Non-default SADI Restraint Value has been used     0.0400 Report
PLAT191_ALERT_3_G A Non-default SADI Restraint Value has been used     0.0400 Report
PLAT191_ALERT_3_G A Non-default SADI Restraint Value has been used     0.0400 Report
PLAT191_ALERT_3_G A Non-default SADI Restraint Value has been used     0.0400 Report
PLAT191_ALERT_3_G A Non-default SADI Restraint Value has been used     0.0400 Report
PLAT191_ALERT_3_G A Non-default SADI Restraint Value has been used     0.0400 Report
PLAT191_ALERT_3_G A Non-default SADI Restraint Value has been used     0.0400 Report
PLAT191_ALERT_3_G A Non-default SADI Restraint Value has been used     0.0400 Report
PLAT191_ALERT_3_G A Non-default SADI Restraint Value has been used     0.0400 Report
PLAT191_ALERT_3_G A Non-default SADI Restraint Value has been used     0.0400 Report
PLAT191_ALERT_3_G A Non-default SADI Restraint Value has been used     0.0400 Report
PLAT191_ALERT_3_G A Non-default SADI Restraint Value has been used     0.0400 Report
PLAT191_ALERT_3_G A Non-default SADI Restraint Value has been used     0.0400 Report
```

```
PLAT191_ALERT_3_G A Non-default SADI Restraint Value has been used     0.0400 Report
PLAT191_ALERT_3_G A Non-default SADI Restraint Value has been used     0.0400 Report
PLAT191_ALERT_3_G A Non-default SADI Restraint Value has been used     0.0400 Report
PLAT191_ALERT_3_G A Non-default SADI Restraint Value has been used     0.0400 Report
PLAT191_ALERT_3_G A Non-default SADI Restraint Value has been used     0.0400 Report
PLAT191_ALERT_3_G A Non-default SADI Restraint Value has been used     0.0400 Report
PLAT191_ALERT_3_G A Non-default SADI Restraint Value has been used     0.0400 Report
PLAT191_ALERT_3_G A Non-default SADI Restraint Value has been used     0.0400 Report
PLAT191_ALERT_3_G A Non-default SADI Restraint Value has been used     0.0400 Report
PLAT191_ALERT_3_G A Non-default SADI Restraint Value has been used     0.0400 Report
PLAT191_ALERT_3_G A Non-default SADI Restraint Value has been used     0.0400 Report
PLAT191_ALERT_3_G A Non-default SADI Restraint Value has been used     0.0400 Report
PLAT191_ALERT_3_G A Non-default SADI Restraint Value has been used     0.0400 Report
PLAT191_ALERT_3_G A Non-default SADI Restraint Value has been used     0.0400 Report
PLAT302_ALERT_4_G Anion/Solvent/Minor-Residue Disorder (Resd    2)       100% Note
PLAT302_ALERT_4_G Anion/Solvent/Minor-Residue Disorder (Resd    3)       100% Note
PLAT302_ALERT_4_G Anion/Solvent/Minor-Residue Disorder (Resd    4)       100% Note
PLAT302_ALERT_4_G Anion/Solvent/Minor-Residue Disorder (Resd    5)       100% Note
PLAT304_ALERT_4_G Non-Integer Number of Atoms in ..... (Resd    2)       2.60 Check
PLAT304_ALERT_4_G Non-Integer Number of Atoms in ..... (Resd    3)       2.05 Check
PLAT304_ALERT_4_G Non-Integer Number of Atoms in ..... (Resd    4)       1.13 Check
PLAT304_ALERT_4_G Non-Integer Number of Atoms in ..... (Resd    5)       1.22 Check
PLAT432_ALERT_2_G Short Inter X...Y Contact  F2B      ..C17      .       2.96 Ang.
                                                      x,y,z  =      1_555 Check
PLAT432_ALERT_2_G Short Inter X...Y Contact  F7D      ..C21      .       2.96 Ang.
                                                    1+x,y,z  =      1_655 Check
PLAT811_ALERT_5_G No ADDSYM Analysis: Too Many Excluded Atoms ....          ! Info
PLAT860_ALERT_3_G Number of Least-Squares Restraints ............         127 Note
PLAT912_ALERT_4_G Missing # of FCF Reflections Above STh/L=  0.600         44 Note
PLAT969_ALERT_5_G The 'Henn et al.' R-Factor-gap value ..........       3.293 Note
             Predicted wR2: Based on SigI**2  2.46 or SHELX Weight  7.50
PLAT978_ALERT_2_G Number C-C Bonds with Positive Residual Density.          2 Info
```

---

0 **ALERT level A** = Most likely a serious problem - resolve or explain
0 **ALERT level B** = A potentially serious problem, consider carefully
1 **ALERT level C** = Check. Ensure it is not caused by an omission or oversight
65 **ALERT level G** = General information/check it is not something unexpected

2 ALERT type 1 CIF construction/syntax error, inconsistent or missing data
4 ALERT type 2 Indicator that the structure model may be wrong or deficient
47 ALERT type 3 Indicator that the structure quality may be low
11 ALERT type 4 Improvement, methodology, query or suggestion
2 ALERT type 5 Informative message, check

It is advisable to attempt to resolve as many as possible of the alerts in all categories. Often the minor alerts point to easily fixed oversights, errors and omissions in your CIF or refinement strategy, so attention to these fine details can be worthwhile. It is up to the individual to critically assess their own results and, if necessary, seek expert advice.

PLATON version of 26/09/2025; check.def file version of 20/09/2025

duplicate check

No duplication found

Datablock 2k - ellipsoid plot

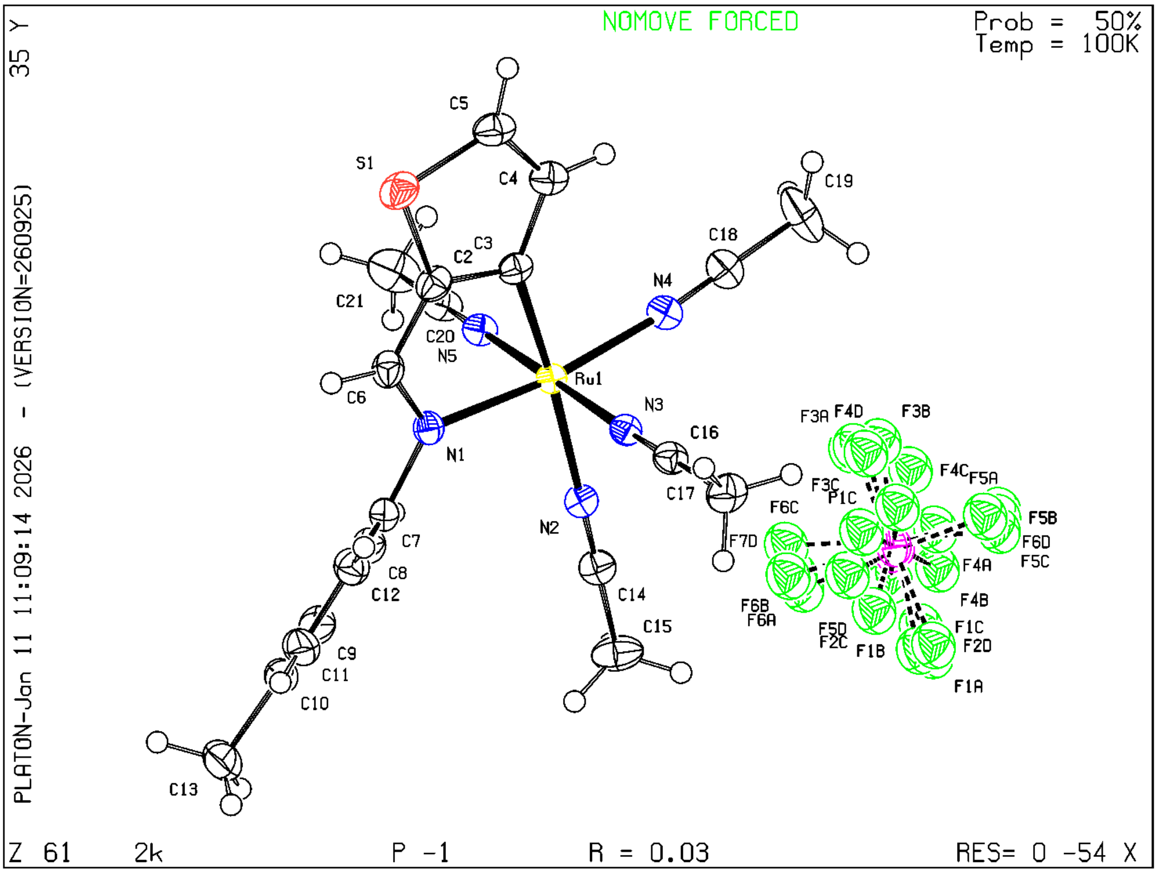

checkCIF/PLATON report

Structure factors have been supplied for datablock(s) 2l

THIS REPORT IS FOR GUIDANCE ONLY. IF USED AS PART OF A REVIEW PROCEDURE FOR PUBLICATION, IT SHOULD NOT REPLACE THE EXPERTISE OF AN EXPERIENCED CRYSTALLOGRAPHIC REFEREE.

No syntax errors found.   CIF dictionary   Interpreting this report

Datablock: 2l

```
Bond precision:   C-C = 0.0045 A           Wavelength=1.54184

Cell:            a=10.6030(2)      b=10.8966(2)      c=24.7104(3)
                 alpha=81.331(1)   beta=88.641(1)    gamma=63.214(2)
Temperature:     100 K
```

	Calculated	Reported
Volume	2516.33(9)	2516.33(8)
Space group	P -1	P -1
Hall group	-P 1	-P 1
Moiety formula	C20 H22 N5 O Ru S, F6 P	C20 H22 N5 O Ru S, F6 P
Sum formula	C20 H22 F6 N5 O P Ru S	C20 H22 F6 N5 O P Ru S
Mr	626.53	626.52
Dx,g cm-3	1.654	1.654
Z	4	4
Mu (mm-1)	7.045	7.045
F000	1256.0	1256.0
F000'	1262.68	
h,k,lmax	13,13,31	13,13,31
Nref	10995	10879
Tmin,Tmax	0.040,0.076	0.014,0.669
Tmin'	0.002	

```
Correction method= # Reported T Limits: Tmin=0.014 Tmax=0.669
AbsCorr = GAUSSIAN
```

```
Data completeness= 0.989            Theta(max)= 80.098

R(reflections)= 0.0432( 10569)                          wR2(reflections)=
                                                        0.1150( 10879)
S = 1.133                 Npar= 642
```

The following ALERTS were generated. Each ALERT has the format
test-name_ALERT_alert-type_alert-level.
Click on the hyperlinks for more details of the test.

Alert level C

```
PLAT244_ALERT_4_C Low    'Solvent' Ueq as Compared to Neighbors of         P2 Check
PLAT906_ALERT_3_C Large K Value in the Analysis of Variance ......      2.799 Check
PLAT911_ALERT_3_C Missing FCF Refl Between Thmin & STh/L=    0.600          8 Report
               11  8 12,  11  9 12,  11  8 13,  11  9 13,  11  8 14,  11  7 15,
               10  8 16,  10  8 17,
```

Alert level G

```
PLAT063_ALERT_4_G Crystal Size Possibly too Large for Beam Size ..       0.77 mm
PLAT232_ALERT_2_G Hirshfeld Test Diff (M-X)  Ru2      --N7       .        7.1 s.u.
PLAT232_ALERT_2_G Hirshfeld Test Diff (M-X)  Ru2      --N9       .        6.5 s.u.
PLAT432_ALERT_2_G Short Inter X...Y Contact  C14      ..C20      .       3.14 Ang.
                                              1-x,1-y,1-z  =     2_666 Check
PLAT432_ALERT_2_G Short Inter X...Y Contact  C14      ..C18      .       3.15 Ang.
                                              1-x,1-y,1-z  =     2_666 Check
PLAT432_ALERT_2_G Short Inter X...Y Contact  C18      ..C20      .       3.12 Ang.
                                              1-x,1-y,1-z  =     2_666 Check
PLAT432_ALERT_2_G Short Inter X...Y Contact  C34      ..C38      .       3.05 Ang.
                                                 -x,-y,-z  =     2_555 Check
PLAT432_ALERT_2_G Short Inter X...Y Contact  C34      ..C40      .       3.17 Ang.
                                                 -x,-y,-z  =     2_555 Check
PLAT910_ALERT_3_G Missing FCF Reflection(s) Below Theta(Min)[Deg]=       3.62 Note
                0  0  1,
PLAT912_ALERT_4_G Missing # of FCF Reflections Above STh/L=  0.600        107 Note
PLAT969_ALERT_5_G The 'Henn et al.' R-Factor-gap value ..........      3.140 Note
              Predicted wR2: Based on SigI**2  3.66 or SHELX Weight 10.15
PLAT978_ALERT_2_G Number C-C Bonds with Positive Residual Density.          0 Info
```

---

   0 **ALERT level A** = Most likely a serious problem - resolve or explain
   0 **ALERT level B** = A potentially serious problem, consider carefully
   3 **ALERT level C** = Check. Ensure it is not caused by an omission or oversight
  12 **ALERT level G** = General information/check it is not something unexpected

```
   0 ALERT type 1 CIF construction/syntax error, inconsistent or missing data
   8 ALERT type 2 Indicator that the structure model may be wrong or deficient
   3 ALERT type 3 Indicator that the structure quality may be low
   3 ALERT type 4 Improvement, methodology, query or suggestion
   1 ALERT type 5 Informative message, check
```

It is advisable to attempt to resolve as many as possible of the alerts in all categories. Often the minor alerts point to easily fixed oversights, errors and omissions in your CIF or refinement strategy, so attention to these fine details can be worthwhile. It is up to the individual to critically assess their own results and, if necessary, seek expert advice.

PLATON version of 26/09/2025; check.def file version of 20/09/2025

---

duplicate check

No duplication found

Datablock 2l - ellipsoid plot

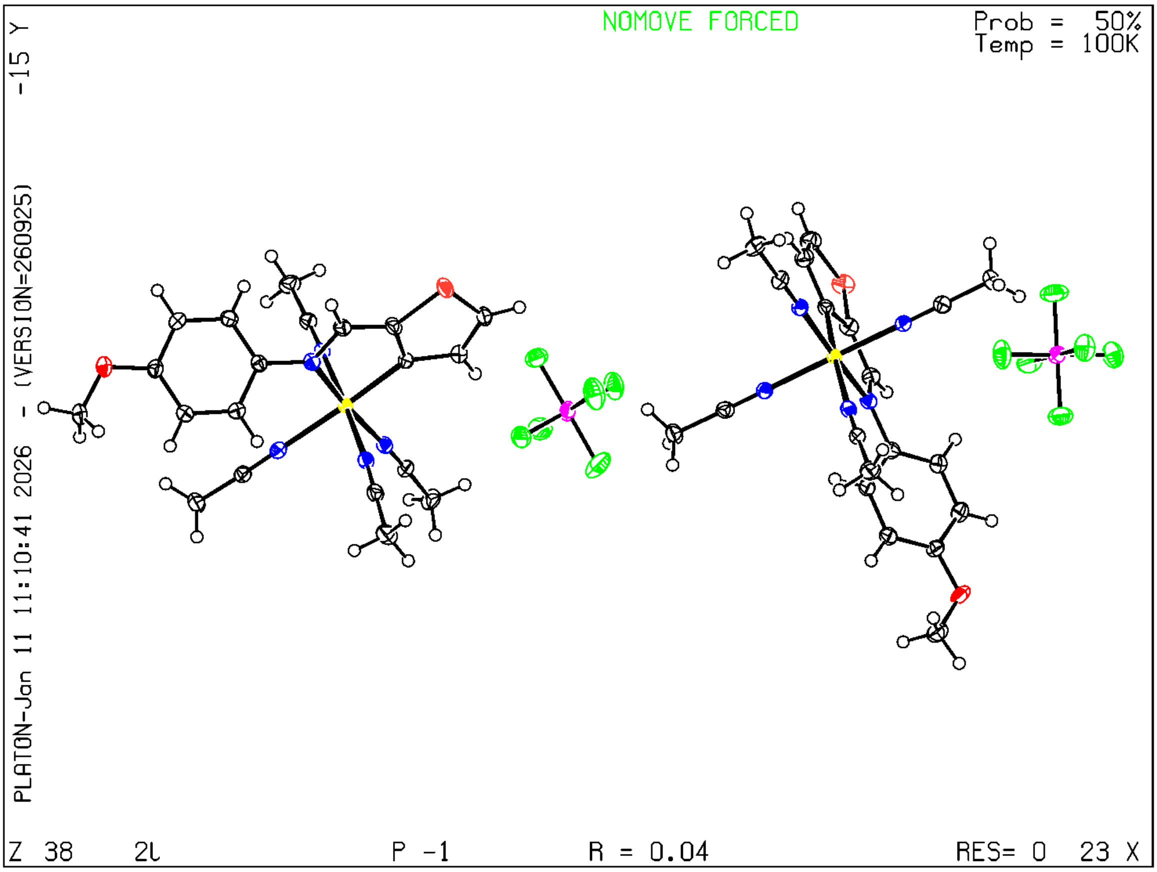

checkCIF/PLATON report

Structure factors have been supplied for datablock(s) 2n

THIS REPORT IS FOR GUIDANCE ONLY. IF USED AS PART OF A REVIEW PROCEDURE FOR PUBLICATION, IT SHOULD NOT REPLACE THE EXPERTISE OF AN EXPERIENCED CRYSTALLOGRAPHIC REFEREE.

No syntax errors found. CIF dictionary Interpreting this report

Datablock: 2n

```
Bond precision:   C-C = 0.0047 A          Wavelength=1.54184

Cell:           a=8.4253(3)        b=12.0245(4)       c=13.6424(7)
                alpha=107.573(4)   beta=106.241(4)    gamma=97.271(3)
Temperature:    100 K

                 Calculated                   Reported
Volume           1230.96(10)                  1230.96(9)
Space group      P -1                         P -1
Hall group       -P 1                         -P 1
Moiety formula   C19 H19 N6 O2 Ru S, F6 P     C19 H19 N6 O2 Ru S, F6 P
Sum formula      C19 H19 F6 N6 O2 P Ru S      C19 H19 F6 N6 O2 P Ru S
Mr               641.50                       641.50
Dx,g cm-3        1.731                        1.731
Z                2                            2
Mu (mm-1)        7.263                        7.263
F000             640.0                        640.0
F000'            643.46
h,k,lmax         10,15,17                     10,15,17
Nref             5387                         5325
Tmin,Tmax        0.246,0.748                  0.211,1.000
Tmin'            0.157

Correction method= # Reported T Limits: Tmin=0.211 Tmax=1.000
AbsCorr = GAUSSIAN
```

```
Data completeness= 0.988           Theta(max)= 79.970

                                                       wR2(reflections)=
R(reflections)= 0.0347( 5231)                          0.0917( 5325)

S = 1.080                   Npar= 373
```

```
The following ALERTS were generated. Each ALERT has the format
       test-name_ALERT_alert-type_alert-level.
Click on the hyperlinks for more details of the test.
```

Alert level C

```
PLAT911_ALERT_3_C Missing FCF Refl Between Thmin & STh/L=    0.600          5 Report
                5  8  3,   4  8  4,   4  7  6,   3  8  6,  -5  2  8,
```

Alert level G

```
PLAT002_ALERT_2_G Number of Distance or Angle Restraints on AtSite         28 Note
PLAT171_ALERT_4_G The CIF-Embedded .res File Contains EADP Records          2 Report
PLAT176_ALERT_4_G The CIF-Embedded .res File Contains SADI Records          6 Report
PLAT191_ALERT_3_G A Non-default SADI Restraint Value has been used     0.0400 Report
PLAT191_ALERT_3_G A Non-default SADI Restraint Value has been used     0.0400 Report
PLAT191_ALERT_3_G A Non-default SADI Restraint Value has been used     0.0400 Report
PLAT191_ALERT_3_G A Non-default SADI Restraint Value has been used     0.0400 Report
PLAT191_ALERT_3_G A Non-default SADI Restraint Value has been used     0.0400 Report
PLAT230_ALERT_2_G Hirshfeld Test Diff for    N2       --C13      .        6.0 s.u.
PLAT232_ALERT_2_G Hirshfeld Test Diff (M-X)  Ru1      --N4       .        5.2 s.u.
PLAT232_ALERT_2_G Hirshfeld Test Diff (M-X)  Ru1      --N5       .        5.5 s.u.
PLAT302_ALERT_4_G Anion/Solvent/Minor-Residue Disorder (Resd    2)       100% Note
PLAT302_ALERT_4_G Anion/Solvent/Minor-Residue Disorder (Resd    3)       100% Note
PLAT302_ALERT_4_G Anion/Solvent/Minor-Residue Disorder (Resd    4)       100% Note
PLAT302_ALERT_4_G Anion/Solvent/Minor-Residue Disorder (Resd    5)       100% Note
PLAT304_ALERT_4_G Non-Integer Number of Atoms in ..... (Resd    2)       2.41 Check
PLAT304_ALERT_4_G Non-Integer Number of Atoms in ..... (Resd    3)       1.51 Check
PLAT304_ALERT_4_G Non-Integer Number of Atoms in ..... (Resd    4)       1.18 Check
PLAT304_ALERT_4_G Non-Integer Number of Atoms in ..... (Resd    5)       1.90 Check
PLAT432_ALERT_2_G Short Inter X...Y Contact  F6C      ..C18      .       2.91 Ang.
                                               1-x,-y,1-z  =      2_656 Check
PLAT811_ALERT_5_G No ADDSYM Analysis: Too Many Excluded Atoms ....          ! Info
PLAT860_ALERT_3_G Number of Least-Squares Restraints ............         557 Note
PLAT912_ALERT_4_G Missing # of FCF Reflections Above STh/L=  0.600         57 Note
PLAT969_ALERT_5_G The 'Henn et al.' R-Factor-gap value ..........       3.956 Note
              Predicted wR2: Based on SigI**2  2.32 or SHELX Weight  8.48
PLAT978_ALERT_2_G Number C-C Bonds with Positive Residual Density.          0 Info
```

---

   0 **ALERT level A** = Most likely a serious problem - resolve or explain
   0 **ALERT level B** = A potentially serious problem, consider carefully
   1 **ALERT level C** = Check. Ensure it is not caused by an omission or oversight
  25 **ALERT level G** = General information/check it is not something unexpected

   0 ALERT type 1 CIF construction/syntax error, inconsistent or missing data

```
 6 ALERT type 2 Indicator that the structure model may be wrong or deficient
 7 ALERT type 3 Indicator that the structure quality may be low
11 ALERT type 4 Improvement, methodology, query or suggestion
 2 ALERT type 5 Informative message, check
```

It is advisable to attempt to resolve as many as possible of the alerts in all categories. Often the minor alerts point to easily fixed oversights, errors and omissions in your CIF or refinement strategy, so attention to these fine details can be worthwhile. It is up to the individual to critically assess their own results and, if necessary, seek expert advice.

PLATON version of 26/09/2025; check.def file version of 20/09/2025

---

duplicate check

No duplication found

Datablock 2n - ellipsoid plot

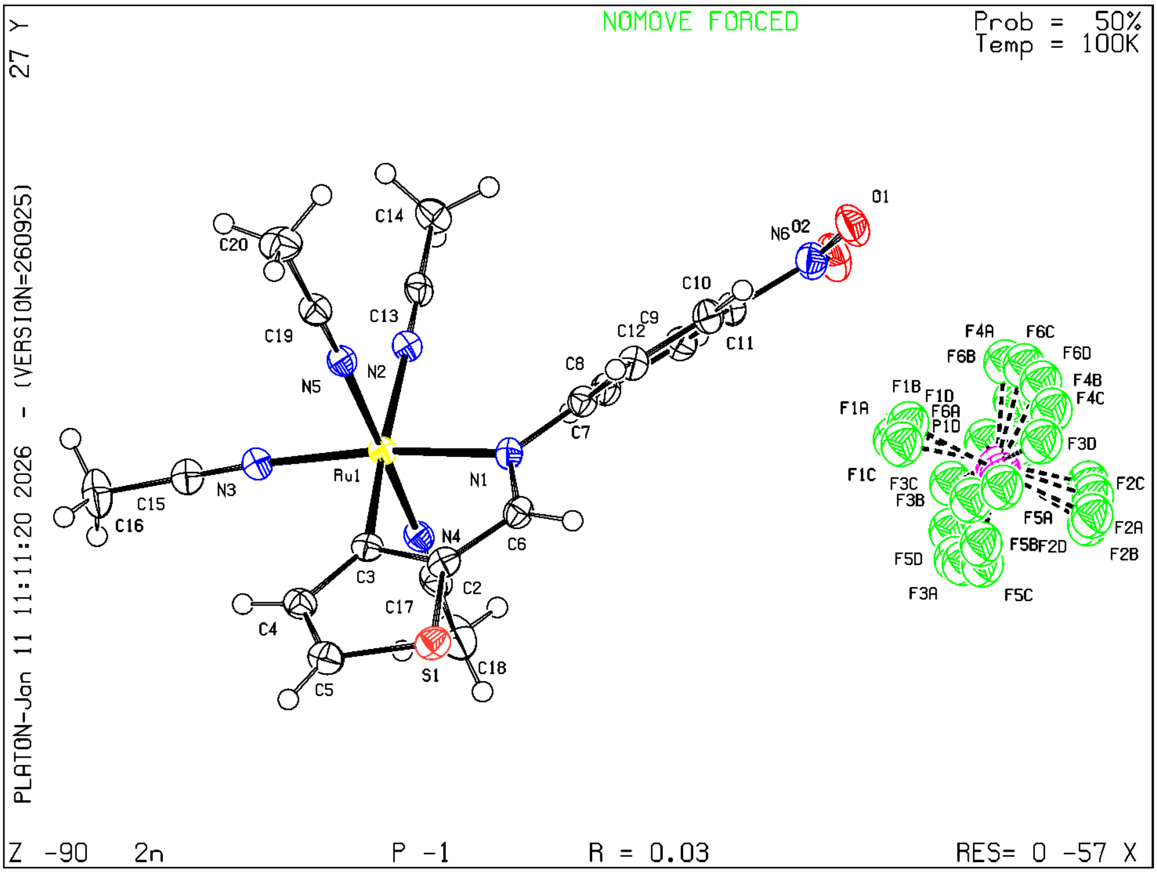

checkCIF/PLATON report

Structure factors have been supplied for datablock(s) 3a

THIS REPORT IS FOR GUIDANCE ONLY. IF USED AS PART OF A REVIEW PROCEDURE FOR PUBLICATION, IT SHOULD NOT REPLACE THE EXPERTISE OF AN EXPERIENCED CRYSTALLOGRAPHIC REFEREE.

No syntax errors found. CIF dictionary Interpreting this report

Datablock: 3a

Bond precision:	C-C = 0.0035 A		Wavelength=1.54184
Cell:	a=11.9888(1)	b=13.0843(1)	c=20.8901(1)
	alpha=90	beta=92.995(1)	gamma=90
Temperature:	100 K		

	Calculated	Reported
Volume	3272.45(4)	3272.45(4)
Space group	P 21/n	P 1 21/n 1
Hall group	-P 2yn	-P 2yn
Moiety formula	C33 H26 N5 Ru, F6 P, C2 H3 N	C33 H26 N5 Ru, F6 P, C2 H3 N
Sum formula	C35 H29 F6 N6 P Ru	C35 H29 F6 N6 P Ru
Mr	779.68	779.68
Dx,g cm-3	1.583	1.583
Z	4	4
Mu (mm-1)	4.959	4.959
F000	1576.0	1576.0
F000'	1582.24	
h,k,lmax	15,16,26	15,16,26
Nref	7159	7134
Tmin,Tmax	0.272,0.485	0.136,1.000
Tmin'	0.035	

Correction method= # Reported T Limits: Tmin=0.136 Tmax=1.000
AbsCorr = GAUSSIAN

Data completeness= 0.997 Theta(max)= 80.170

R(reflections)= 0.0333(7008) wR2(reflections)= 0.0935(7134)

S = 1.117 Npar= 455

The following ALERTS were generated. Each ALERT has the format
test-name_ALERT_alert-type_alert-level.
Click on the hyperlinks for more details of the test.

Alert level C

```
PLAT244_ALERT_4_C Low    'Solvent' Ueq as Compared to Neighbors of         P1 Check
```

Alert level G

```
PLAT002_ALERT_2_G Number of Distance or Angle Restraints on AtSite          6 Note
PLAT063_ALERT_4_G Crystal Size Possibly too Large for Beam Size ..       0.63 mm
PLAT142_ALERT_4_G s.u. on b - Axis Small or Missing .............    0.00010 Ang.
PLAT143_ALERT_4_G s.u. on c - Axis Small or Missing .............    0.00010 Ang.
PLAT153_ALERT_1_G The s.u.'s on the Cell Axes   are Equal ..(Note)     0.0001 Ang.
PLAT171_ALERT_4_G The CIF-Embedded .res File Contains EADP Records          3 Report
PLAT176_ALERT_4_G The CIF-Embedded .res File Contains SADI Records          3 Report
PLAT191_ALERT_3_G A Non-default SADI Restraint Value has been used     0.0400 Report
PLAT302_ALERT_4_G Anion/Solvent/Minor-Residue Disorder (Resd    3)       100% Note
PLAT302_ALERT_4_G Anion/Solvent/Minor-Residue Disorder (Resd    4)       100% Note
PLAT304_ALERT_4_G Non-Integer Number of Atoms in ..... (Resd    3)       3.20 Check
PLAT304_ALERT_4_G Non-Integer Number of Atoms in ..... (Resd    4)       2.80 Check
PLAT860_ALERT_3_G Number of Least-Squares Restraints ............           3 Note
PLAT912_ALERT_4_G Missing # of FCF Reflections Above STh/L=  0.600         25 Note
PLAT933_ALERT_2_G Number of HKL-OMIT Records in Embedded .res File          1 Note
               -4 16  1,
PLAT969_ALERT_5_G The 'Henn et al.' R-Factor-gap value ..........       3.744 Note
              Predicted wR2: Based on SigI**2  2.50 or SHELX Weight  8.37
PLAT978_ALERT_2_G Number C-C Bonds with Positive Residual Density.          6 Info
```

---

0 **ALERT level A** = Most likely a serious problem - resolve or explain
0 **ALERT level B** = A potentially serious problem, consider carefully
1 **ALERT level C** = Check. Ensure it is not caused by an omission or oversight
17 **ALERT level G** = General information/check it is not something unexpected

1 ALERT type 1 CIF construction/syntax error, inconsistent or missing data
3 ALERT type 2 Indicator that the structure model may be wrong or deficient
2 ALERT type 3 Indicator that the structure quality may be low
11 ALERT type 4 Improvement, methodology, query or suggestion
1 ALERT type 5 Informative message, check

It is advisable to attempt to resolve as many as possible of the alerts in all categories. Often the minor alerts point to easily fixed oversights, errors and omissions in your CIF or refinement strategy, so attention to these fine details can be worthwhile. It is up to the individual to critically assess their own results and, if necessary, seek expert advice.

PLATON version of 26/09/2025; check.def file version of 20/09/2025

---

duplicate check

No duplication found

Datablock 3a - ellipsoid plot

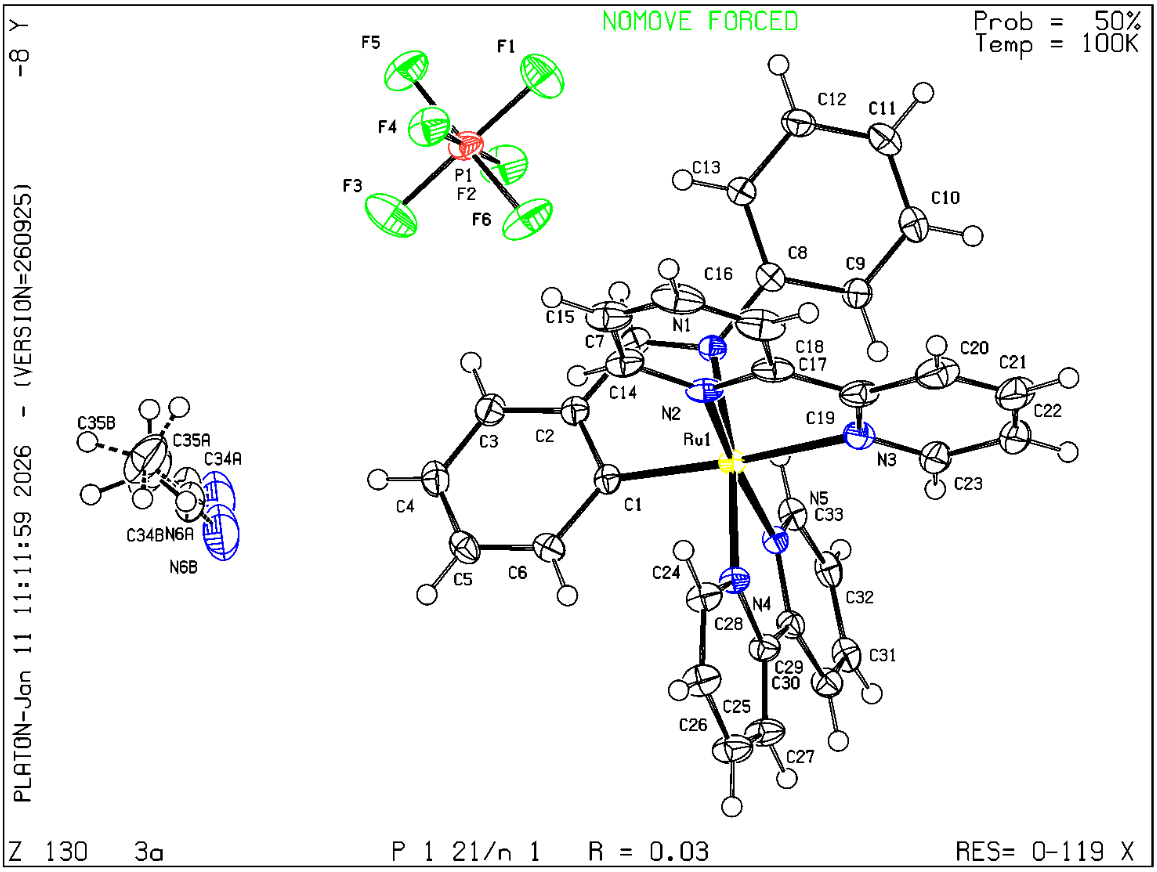

checkCIF/PLATON report

Structure factors have been supplied for datablock(s) 3b

THIS REPORT IS FOR GUIDANCE ONLY. IF USED AS PART OF A REVIEW PROCEDURE FOR PUBLICATION, IT SHOULD NOT REPLACE THE EXPERTISE OF AN EXPERIENCED CRYSTALLOGRAPHIC REFEREE.

No syntax errors found.   CIF dictionary   Interpreting this report

Datablock: 3b

Bond precision:	C-C = 0.0051 A		Wavelength=1.54184
Cell:	a=12.4931(2)	b=13.2824(2)	c=13.5109(2)
	alpha=100.066(1)	beta=116.292(2)	gamma=111.075(2)
Temperature:	100 K		

	Calculated	Reported
Volume	1719.56(8)	1719.56(6)
Space group	P -1	P -1
Hall group	-P 1	-P 1
Moiety formula	C33 H25 F N5 Ru, F6 P, C4 H10 O	C33 H25 F N5 Ru, F6 P, C4 H10 O
Sum formula	C37 H35 F7 N5 O P Ru	C37 H35 F7 N5 O P Ru
Mr	830.74	830.74
Dx,g cm-3	1.605	1.604
Z	2	2
Mu (mm-1)	4.813	4.813
F000	844.0	844.0
F000'	847.38	
h,k,lmax	15,16,17	15,16,17
Nref	7493	7422
Tmin,Tmax	0.186,0.401	0.220,1.000
Tmin'	0.039	

Correction method= # Reported T Limits: Tmin=0.220 Tmax=1.000
AbsCorr = GAUSSIAN

Data completeness= 0.991 Theta(max)= 80.034

R(reflections)= 0.0404(7353) wR2(reflections)= 0.1060(7422)

S = 1.048 Npar= 463

The following ALERTS were generated. Each ALERT has the format
test-name_ALERT_alert-type_alert-level.
Click on the hyperlinks for more details of the test.

Alert level C

```
PLAT911_ALERT_3_C Missing FCF Refl Between Thmin & STh/L=    0.600          7 Report
                4-15  6,   5-15  6,   3-15  7,   4-15  7,   5-15  7,   2-15  8,
                3-15  8,
```

Alert level G

```
PLAT002_ALERT_2_G Number of Distance or Angle Restraints on AtSite         14 Note
PLAT063_ALERT_4_G Crystal Size Possibly too Large for Beam Size ..       0.63 mm
PLAT152_ALERT_1_G The Supplied and Calc. Volume s.u. Differ by ...          2 Units
PLAT171_ALERT_4_G The CIF-Embedded .res File Contains EADP Records          2 Report
PLAT176_ALERT_4_G The CIF-Embedded .res File Contains SADI Records         21 Report
PLAT191_ALERT_3_G A Non-default SADI Restraint Value has been used     0.0400 Report
PLAT191_ALERT_3_G A Non-default SADI Restraint Value has been used     0.0400 Report
PLAT191_ALERT_3_G A Non-default SADI Restraint Value has been used     0.0400 Report
PLAT191_ALERT_3_G A Non-default SADI Restraint Value has been used     0.0400 Report
PLAT191_ALERT_3_G A Non-default SADI Restraint Value has been used     0.0400 Report
PLAT191_ALERT_3_G A Non-default SADI Restraint Value has been used     0.0400 Report
PLAT191_ALERT_3_G A Non-default SADI Restraint Value has been used     0.0400 Report
PLAT191_ALERT_3_G A Non-default SADI Restraint Value has been used     0.0400 Report
PLAT191_ALERT_3_G A Non-default SADI Restraint Value has been used     0.0400 Report
PLAT191_ALERT_3_G A Non-default SADI Restraint Value has been used     0.0400 Report
PLAT191_ALERT_3_G A Non-default SADI Restraint Value has been used     0.0400 Report
PLAT191_ALERT_3_G A Non-default SADI Restraint Value has been used     0.0400 Report
PLAT191_ALERT_3_G A Non-default SADI Restraint Value has been used     0.0400 Report
PLAT191_ALERT_3_G A Non-default SADI Restraint Value has been used     0.0400 Report
PLAT191_ALERT_3_G A Non-default SADI Restraint Value has been used     0.0400 Report
PLAT231_ALERT_4_G Hirshfeld Test (Solvent)   P1A      --F2A      .       13.1 s.u.
PLAT231_ALERT_4_G Hirshfeld Test (Solvent)   P1A      --F3A      .        9.4 s.u.
PLAT231_ALERT_4_G Hirshfeld Test (Solvent)   P1A      --F4A      .       13.4 s.u.
PLAT231_ALERT_4_G Hirshfeld Test (Solvent)   P1A      --F5A      .        9.4 s.u.
PLAT231_ALERT_4_G Hirshfeld Test (Solvent)   P1A      --F6A      .       12.0 s.u.
PLAT231_ALERT_4_G Hirshfeld Test (Solvent)   P1A      --F7A      .       11.8 s.u.
PLAT302_ALERT_4_G Anion/Solvent/Minor-Residue Disorder (Resd    2)       100% Note
PLAT302_ALERT_4_G Anion/Solvent/Minor-Residue Disorder (Resd    3)       100% Note
PLAT432_ALERT_2_G Short Inter X...Y Contact  C17      ..C21      .       3.19 Ang.
                                              1-x,1-y,-z  =      2_665 Check
PLAT860_ALERT_3_G Number of Least-Squares Restraints ............         21 Note
PLAT912_ALERT_4_G Missing # of FCF Reflections Above STh/L=  0.600         64 Note
PLAT961_ALERT_5_G Dataset Contains no Negative Intensities .......     Please Check
PLAT969_ALERT_5_G The 'Henn et al.' R-Factor-gap value ..........       3.611 Note
              Predicted wR2: Based on SigI**2  2.94 or SHELX Weight 10.12
PLAT978_ALERT_2_G Number C-C Bonds with Positive Residual Density.          3 Info
```

```
 0 ALERT level A = Most likely a serious problem - resolve or explain
 0 ALERT level B = A potentially serious problem, consider carefully
 1 ALERT level C = Check. Ensure it is not caused by an omission or oversight
34 ALERT level G = General information/check it is not something unexpected

 1 ALERT type 1 CIF construction/syntax error, inconsistent or missing data
 3 ALERT type 2 Indicator that the structure model may be wrong or deficient
17 ALERT type 3 Indicator that the structure quality may be low
12 ALERT type 4 Improvement, methodology, query or suggestion
 2 ALERT type 5 Informative message, check
```

It is advisable to attempt to resolve as many as possible of the alerts in all categories. Often the minor alerts point to easily fixed oversights, errors and omissions in your CIF or refinement strategy, so attention to these fine details can be worthwhile. It is up to the individual to critically assess their own results and, if necessary, seek expert advice.

PLATON version of 26/09/2025; check.def file version of 20/09/2025

---

duplicate check

No duplication found

Datablock 3b - ellipsoid plot

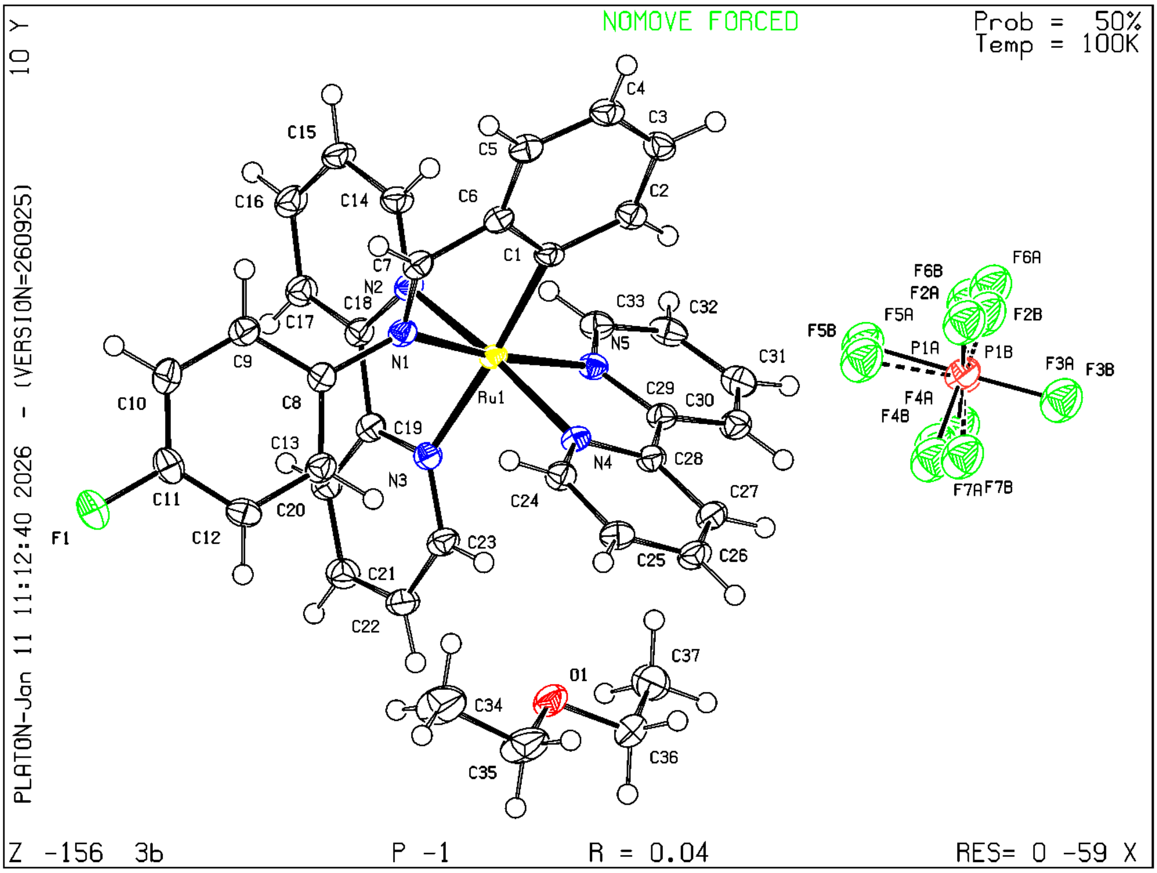

checkCIF/PLATON report

Structure factors have been supplied for datablock(s) 3c

THIS REPORT IS FOR GUIDANCE ONLY. IF USED AS PART OF A REVIEW PROCEDURE FOR PUBLICATION, IT SHOULD NOT REPLACE THE EXPERTISE OF AN EXPERIENCED CRYSTALLOGRAPHIC REFEREE.

No syntax errors found. CIF dictionary Interpreting this report

Datablock: 3c

Bond precision:	C-C = 0.0020 A		Wavelength=0.71073
Cell:	a=11.1184(2)	b=12.9449(2)	c=13.0295(2)
	alpha=83.285(1)	beta=75.762(1)	gamma=71.920(1)
Temperature:	100 K		

	Calculated	Reported
Volume	1726.26(5)	1726.26(5)
Space group	P -1	P -1
Hall group	-P 1	-P 1
Moiety formula	C34 H28 N5 Ru, F6 P [+ solvent]	C34 H28 N5 Ru, 1(F6 P), 0.75[C4H10O]
Sum formula	C34 H28 F6 N5 P Ru [+ solvent]	C37 H35.50 F6 N5 O0.75 P Ru
Mr	752.65	808.24
Dx,g cm-3	1.448	1.555
Z	2	2
Mu (mm-1)	0.564	0.571
F000	760.0	823.0
F000’	758.05	
h,k,lmax	18,21,21	17,20,21
Nref	15635	14589
Tmin,Tmax	0.825,0.944	0.353,1.000
Tmin’	0.706	

Correction method= # Reported T Limits: Tmin=0.353 Tmax=1.000
AbsCorr = GAUSSIAN

Data completeness= 0.933 Theta(max)= 35.385

R(reflections)= 0.0321(13128) wR2(reflections)= 0.0847(14589)

S = 1.044 Npar= 440

The following ALERTS were generated. Each ALERT has the format
test-name_ALERT_alert-type_alert-level.
Click on the hyperlinks for more details of the test.

Alert level C

```
PLAT094_ALERT_2_C Ratio of Maximum / Minimum Residual Density ....       2.17 Report
PLAT977_ALERT_2_C Check Negative Difference Density on H34A      .      -0.35 eA-3
```

Alert level G

```
FORMU01_ALERT_2_G  There is a discrepancy between the atom counts in the
            _chemical_formula_sum and the formula from the _atom_site* data.
            Atom count from _chemical_formula_sum:C37 H35.5 F6 N5 O0.75 P1 Ru1
            Atom count from the _atom_site data:  C34 H28 F6 N5 P1 Ru1
CELLZ01_ALERT_1_G Difference between formula and atom_site contents detected.
CELLZ01_ALERT_1_G ALERT: Large difference may be due to a
            symmetry error - see SYMMG tests
           From the CIF: _cell_formula_units_Z    2
           From the CIF: _chemical_formula_sum  C37 H35.50 F6 N5 O0.75 P Ru
           TEST: Compare cell contents of formula and atom_site data

           atom    Z*formula  cif sites diff
           C         74.00     68.00    6.00
           H         71.00     56.00   15.00
           F         12.00     12.00    0.00
           N         10.00     10.00    0.00
           O          1.50      0.00    1.50
           P          2.00      2.00    0.00
           Ru         2.00      2.00    0.00
PLAT002_ALERT_2_G Number of Distance or Angle Restraints on AtSite         21 Note
PLAT041_ALERT_1_G Calc. and Reported SumFormula    Strings  Differ     Please Check
             Calc: C34 H28 F6 N5 P Ru
             Rep.: C37 H35.50 F6 N5 O0.75 P Ru
PLAT042_ALERT_1_G Calc. and Reported MoietyFormula Strings  Differ     Please Check
             Calc: C34 H28 N5 Ru, F6 P
             Rep.: C34 H28 N5 Ru, 1(F6 P), 0.75[C4H10O]
PLAT063_ALERT_4_G Crystal Size Possibly too Large for Beam Size ..       0.61 mm
PLAT154_ALERT_1_G The s.u.'s on the Cell Angles are Equal ..(Note)      0.001 Degree
PLAT171_ALERT_4_G The CIF-Embedded .res File Contains EADP Records          2 Report
PLAT176_ALERT_4_G The CIF-Embedded .res File Contains SADI Records         42 Report
PLAT191_ALERT_3_G A Non-default SADI Restraint Value has been used     0.0400 Report
PLAT191_ALERT_3_G A Non-default SADI Restraint Value has been used     0.0400 Report
PLAT191_ALERT_3_G A Non-default SADI Restraint Value has been used     0.0400 Report
```

```
PLAT191_ALERT_3_G A Non-default SADI Restraint Value has been used     0.0400 Report
PLAT191_ALERT_3_G A Non-default SADI Restraint Value has been used     0.0400 Report
PLAT191_ALERT_3_G A Non-default SADI Restraint Value has been used     0.0400 Report
PLAT191_ALERT_3_G A Non-default SADI Restraint Value has been used     0.0400 Report
PLAT191_ALERT_3_G A Non-default SADI Restraint Value has been used     0.0400 Report
PLAT191_ALERT_3_G A Non-default SADI Restraint Value has been used     0.0400 Report
PLAT191_ALERT_3_G A Non-default SADI Restraint Value has been used     0.0400 Report
PLAT191_ALERT_3_G A Non-default SADI Restraint Value has been used     0.0400 Report
PLAT191_ALERT_3_G A Non-default SADI Restraint Value has been used     0.0400 Report
PLAT191_ALERT_3_G A Non-default SADI Restraint Value has been used     0.0400 Report
PLAT191_ALERT_3_G A Non-default SADI Restraint Value has been used     0.0400 Report
PLAT191_ALERT_3_G A Non-default SADI Restraint Value has been used     0.0400 Report
PLAT191_ALERT_3_G A Non-default SADI Restraint Value has been used     0.0400 Report
PLAT191_ALERT_3_G A Non-default SADI Restraint Value has been used     0.0400 Report
PLAT191_ALERT_3_G A Non-default SADI Restraint Value has been used     0.0400 Report
PLAT191_ALERT_3_G A Non-default SADI Restraint Value has been used     0.0400 Report
PLAT191_ALERT_3_G A Non-default SADI Restraint Value has been used     0.0400 Report
PLAT191_ALERT_3_G A Non-default SADI Restraint Value has been used     0.0400 Report
PLAT191_ALERT_3_G A Non-default SADI Restraint Value has been used     0.0400 Report
PLAT191_ALERT_3_G A Non-default SADI Restraint Value has been used     0.0400 Report
PLAT191_ALERT_3_G A Non-default SADI Restraint Value has been used     0.0400 Report
PLAT191_ALERT_3_G A Non-default SADI Restraint Value has been used     0.0400 Report
PLAT191_ALERT_3_G A Non-default SADI Restraint Value has been used     0.0400 Report
PLAT191_ALERT_3_G A Non-default SADI Restraint Value has been used     0.0400 Report
PLAT191_ALERT_3_G A Non-default SADI Restraint Value has been used     0.0400 Report
PLAT191_ALERT_3_G A Non-default SADI Restraint Value has been used     0.0400 Report
PLAT191_ALERT_3_G A Non-default SADI Restraint Value has been used     0.0400 Report
PLAT232_ALERT_2_G Hirshfeld Test Diff (M-X)  Ru1      --N2       .        5.2 s.u.
PLAT232_ALERT_2_G Hirshfeld Test Diff (M-X)  Ru1      --N4       .        5.8 s.u.
PLAT232_ALERT_2_G Hirshfeld Test Diff (M-X)  Ru1      --C2       .        6.2 s.u.
PLAT302_ALERT_4_G Anion/Solvent/Minor-Residue Disorder (Resd    2)       100% Note
PLAT302_ALERT_4_G Anion/Solvent/Minor-Residue Disorder (Resd    3)       100% Note
PLAT302_ALERT_4_G Anion/Solvent/Minor-Residue Disorder (Resd    4)       100% Note
PLAT304_ALERT_4_G Non-Integer Number of Atoms in ..... (Resd    2)       3.50 Check
PLAT304_ALERT_4_G Non-Integer Number of Atoms in ..... (Resd    4)       1.49 Check
PLAT605_ALERT_4_G Largest Solvent Accessible VOID in the Structure        252 A**3
PLAT860_ALERT_3_G Number of Least-Squares Restraints ............          64 Note
PLAT868_ALERT_4_G ALERTS Due to the Use of _smtbx_masks Suppressed          ! Info
PLAT910_ALERT_3_G Missing FCF Reflection(s) Below Theta(Min)[Deg]=       2.18 Note
                1  0  0,   0  1  0,   0  0  1,
PLAT912_ALERT_4_G Missing # of FCF Reflections Above STh/L=  0.600       1015 Note
PLAT933_ALERT_2_G Number of HKL-OMIT Records in Embedded .res File          1 Note
                1  0  0,
PLAT969_ALERT_5_G The 'Henn et al.' R-Factor-gap value ..........       2.747 Note
              Predicted wR2: Based on SigI**2  3.08 or SHELX Weight  8.11
PLAT978_ALERT_2_G Number C-C Bonds with Positive Residual Density.          9 Info
```

---

0 **ALERT level A** = Most likely a serious problem - resolve or explain
0 **ALERT level B** = A potentially serious problem, consider carefully
2 **ALERT level C** = Check. Ensure it is not caused by an omission or oversight
56 **ALERT level G** = General information/check it is not something unexpected

5 ALERT type 1 CIF construction/syntax error, inconsistent or missing data

```
 9 ALERT type 2 Indicator that the structure model may be wrong or deficient
32 ALERT type 3 Indicator that the structure quality may be low
11 ALERT type 4 Improvement, methodology, query or suggestion
 1 ALERT type 5 Informative message, check
```

It is advisable to attempt to resolve as many as possible of the alerts in all categories. Often the minor alerts point to easily fixed oversights, errors and omissions in your CIF or refinement strategy, so attention to these fine details can be worthwhile. It is up to the individual to critically assess their own results and, if necessary, seek expert advice.

PLATON version of 26/09/2025; check.def file version of 20/09/2025

duplicate check

No duplication found

Datablock 3c - ellipsoid plot

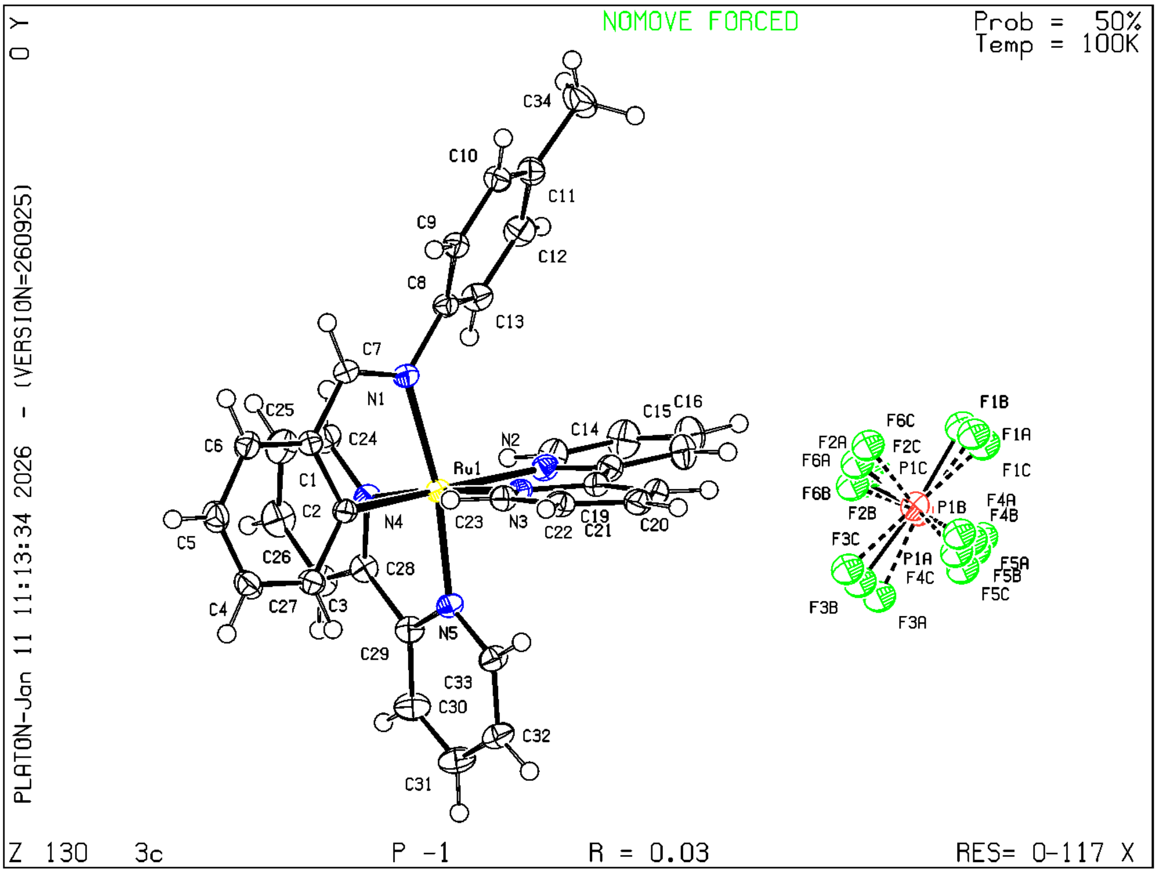

checkCIF/PLATON report

Structure factors have been supplied for datablock(s) 3e

THIS REPORT IS FOR GUIDANCE ONLY. IF USED AS PART OF A REVIEW PROCEDURE FOR PUBLICATION, IT SHOULD NOT REPLACE THE EXPERTISE OF AN EXPERIENCED CRYSTALLOGRAPHIC REFEREE.

No syntax errors found. CIF dictionary Interpreting this report

Datablock: 3e

Bond precision: C-C = 0.0016 A Wavelength=0.71073

Cell:	a=11.7141(2)	b=12.5133(2)	c=12.9723(2)
	alpha=83.745(1)	beta=89.781(1)	gamma=71.786(1)
Temperature:	100 K		

	Calculated	Reported
Volume	1794.56(5)	1794.56(5)
Space group	P -1	P-1
Hall group	-P 1	-P 1
Moiety formula	C37 H34 N5 Ru, F6 P, C2 H3 N	C37 H34 N5 Ru, F6 P, C2 H3 N
Sum formula	C39 H37 F6 N6 P Ru	C39 H37 F6 N6 P Ru
Mr	835.79	835.78
Dx,g cm-3	1.547	1.547
Z	2	2
Mu (mm-1)	0.551	0.551
F000	852.0	852.0
F000'	850.07	
h,k,lmax	18,19,19	18,19,19
Nref	13719	13707
Tmin,Tmax	0.810,0.889	0.526,0.566
Tmin'	0.792	

Correction method= # Reported T Limits: Tmin=0.526 Tmax=0.566
AbsCorr = MULTI-SCAN

Data completeness= 0.999 Theta(max)= 33.197

R(reflections)= 0.0239(12549) wR2(reflections)= 0.0590(13707)

S = 1.042 Npar= 482

The following ALERTS were generated. Each ALERT has the format
test-name_ALERT_alert-type_alert-level.
Click on the hyperlinks for more details of the test.

Alert level C

```
PLAT244_ALERT_4_C Low    'Solvent' Ueq as Compared to Neighbors of        C38 Check
PLAT911_ALERT_3_C Missing FCF Refl Between Thmin & STh/L=    0.600          2 Report
                0 -1  1,   1  1  1,
```

Alert level G

```
PLAT154_ALERT_1_G The s.u.'s on the Cell Angles are Equal ..(Note)      0.001 Degree
PLAT232_ALERT_2_G Hirshfeld Test Diff (M-X)  Ru1      --N2       .        6.5 s.u.
PLAT232_ALERT_2_G Hirshfeld Test Diff (M-X)  Ru1      --N3       .        5.8 s.u.
PLAT232_ALERT_2_G Hirshfeld Test Diff (M-X)  Ru1      --N5       .        6.5 s.u.
PLAT232_ALERT_2_G Hirshfeld Test Diff (M-X)  Ru1      --C2       .        5.5 s.u.
PLAT910_ALERT_3_G Missing FCF Reflection(s) Below Theta(Min)[Deg]=       2.08 Note
                1  0  0,   0  1  0,   0  0  1,
PLAT912_ALERT_4_G Missing # of FCF Reflections Above STh/L=  0.600          7 Note
PLAT933_ALERT_2_G Number of HKL-OMIT Records in Embedded .res File          1 Note
                1  1  1,
PLAT969_ALERT_5_G The 'Henn et al.' R-Factor-gap value ..........      3.248 Note
              Predicted wR2: Based on SigI**2  1.82 or SHELX Weight  5.66
PLAT978_ALERT_2_G Number C-C Bonds with Positive Residual Density.         23 Info
```

---

0 **ALERT level A** = Most likely a serious problem - resolve or explain
0 **ALERT level B** = A potentially serious problem, consider carefully
2 **ALERT level C** = Check. Ensure it is not caused by an omission or oversight
10 **ALERT level G** = General information/check it is not something unexpected

1 ALERT type 1 CIF construction/syntax error, inconsistent or missing data
6 ALERT type 2 Indicator that the structure model may be wrong or deficient
2 ALERT type 3 Indicator that the structure quality may be low
2 ALERT type 4 Improvement, methodology, query or suggestion
1 ALERT type 5 Informative message, check

It is advisable to attempt to resolve as many as possible of the alerts in all categories. Often the minor alerts point to easily fixed oversights, errors and omissions in your CIF or refinement strategy, so attention to these fine details can be worthwhile. It is up to the individual to critically assess their own results and, if necessary, seek expert advice.

PLATON version of 26/09/2025; check.def file version of 20/09/2025

duplicate check

No duplication found

Datablock 3e - ellipsoid plot

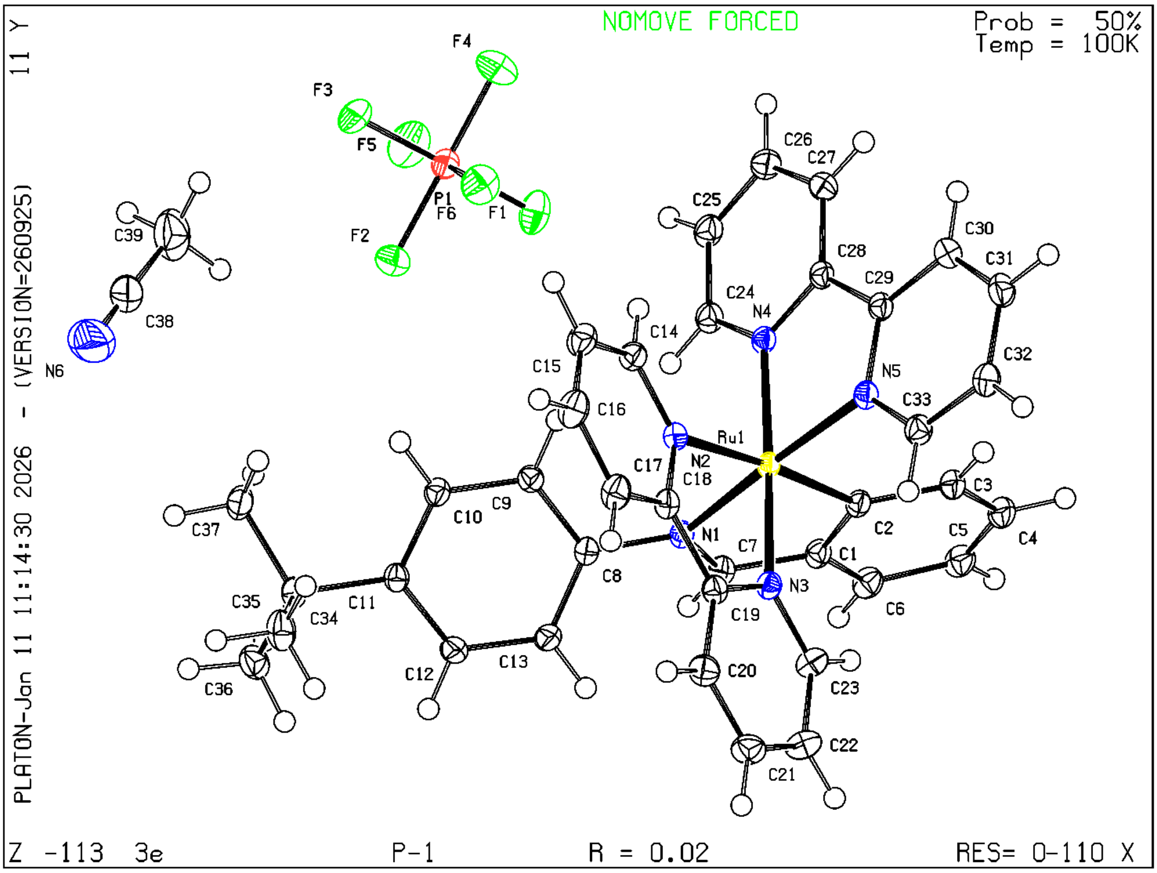

checkCIF/PLATON report

Structure factors have been supplied for datablock(s) 3f

THIS REPORT IS FOR GUIDANCE ONLY. IF USED AS PART OF A REVIEW PROCEDURE FOR PUBLICATION, IT SHOULD NOT REPLACE THE EXPERTISE OF AN EXPERIENCED CRYSTALLOGRAPHIC REFEREE.

No syntax errors found. CIF dictionary Interpreting this report

Datablock: 3f

```
Bond precision:   C-C = 0.0030 A          Wavelength=1.54184

Cell:             a=13.11770(6)     b=11.20731(5)       c=23.18196(9)
                  alpha=90          beta=97.3765(4)     gamma=90
Temperature:      100 K
```

	Calculated	Reported
Volume	3379.87(3)	3379.87(2)
Space group	P 21/n	P 1 21/n 1
Hall group	-P 2yn	-P 2yn
Moiety formula	C34 H28 N5 O Ru, F6 P, C2 H3 N	C34 H28 N5 O Ru, F6 P, C2 H3 N
Sum formula	C36 H31 F6 N6 O P Ru	C36 H31 F6 N6 O P Ru
Mr	809.71	809.71
Dx,g cm-3	1.591	1.591
Z	4	4
Mu (mm-1)	4.848	4.848
F000	1640.0	1640.0
F000'	1646.50	
h,k,lmax	16,14,29	16,14,29
Nref	7370	7341
Tmin,Tmax	0.316,0.688	0.376,1.000
Tmin'	0.230	

```
Correction method= # Reported T Limits: Tmin=0.376 Tmax=1.000
AbsCorr = GAUSSIAN
```

Data completeness= 0.996 Theta(max)= 80.004

R(reflections)= 0.0290(7125) wR2(reflections)= 0.0722(7341)

S = 1.019 Npar= 477

The following ALERTS were generated. Each ALERT has the format
test-name_ALERT_alert-type_alert-level.
Click on the hyperlinks for more details of the test.

Alert level G

```
PLAT002_ALERT_2_G Number of Distance or Angle Restraints on AtSite         21 Note
PLAT142_ALERT_4_G s.u. on b - Axis Small or Missing .............     0.00005 Ang.
PLAT143_ALERT_4_G s.u. on c - Axis Small or Missing .............     0.00009 Ang.
PLAT145_ALERT_4_G s.u. on beta     Small or Missing .............      0.0004 Degree
PLAT171_ALERT_4_G The CIF-Embedded .res File Contains EADP Records          2 Report
PLAT176_ALERT_4_G The CIF-Embedded .res File Contains SADI Records          7 Report
PLAT191_ALERT_3_G A Non-default SADI Restraint Value has been used     0.0400 Report
PLAT302_ALERT_4_G Anion/Solvent/Minor-Residue Disorder (Resd    2)       100% Note
PLAT302_ALERT_4_G Anion/Solvent/Minor-Residue Disorder (Resd    3)       100% Note
PLAT302_ALERT_4_G Anion/Solvent/Minor-Residue Disorder (Resd    4)       100% Note
PLAT304_ALERT_4_G Non-Integer Number of Atoms in ..... (Resd    2)       3.22 Check
PLAT304_ALERT_4_G Non-Integer Number of Atoms in ..... (Resd    3)       2.33 Check
PLAT304_ALERT_4_G Non-Integer Number of Atoms in ..... (Resd    4)       1.45 Check
PLAT432_ALERT_2_G Short Inter X...Y Contact  F2A      ..C7       .       2.89 Ang.
                                                  x,y,z  =      1_555 Check
PLAT432_ALERT_2_G Short Inter X...Y Contact  F2C      ..C7       .       2.79 Ang.
                                                  x,y,z  =      1_555 Check
PLAT432_ALERT_2_G Short Inter X...Y Contact  F3B      ..C16      .       2.96 Ang.
                                      3/2-x,1/2+y,1/2-z  =      2_655 Check
PLAT860_ALERT_3_G Number of Least-Squares Restraints ............        649 Note
PLAT912_ALERT_4_G Missing # of FCF Reflections Above STh/L=  0.600        28 Note
PLAT969_ALERT_5_G The 'Henn et al.' R-Factor-gap value ..........      2.935 Note
              Predicted wR2: Based on SigI**2  2.46 or SHELX Weight  7.08
PLAT978_ALERT_2_G Number C-C Bonds with Positive Residual Density.          4 Info
```

---

0 **ALERT level A** = Most likely a serious problem - resolve or explain
0 **ALERT level B** = A potentially serious problem, consider carefully
0 **ALERT level C** = Check. Ensure it is not caused by an omission or oversight
20 **ALERT level G** = General information/check it is not something unexpected

0 ALERT type 1 CIF construction/syntax error, inconsistent or missing data
5 ALERT type 2 Indicator that the structure model may be wrong or deficient
2 ALERT type 3 Indicator that the structure quality may be low
12 ALERT type 4 Improvement, methodology, query or suggestion
1 ALERT type 5 Informative message, check

It is advisable to attempt to resolve as many as possible of the alerts in all categories. Often the minor alerts point to easily fixed oversights, errors and omissions in your CIF or refinement strategy, so attention to these fine details can be worthwhile. It is up to the individual to critically assess their own results and, if necessary, seek expert advice.

PLATON version of 26/09/2025; check.def file version of 20/09/2025

---

duplicate check

No duplication found

Datablock 3f - ellipsoid plot

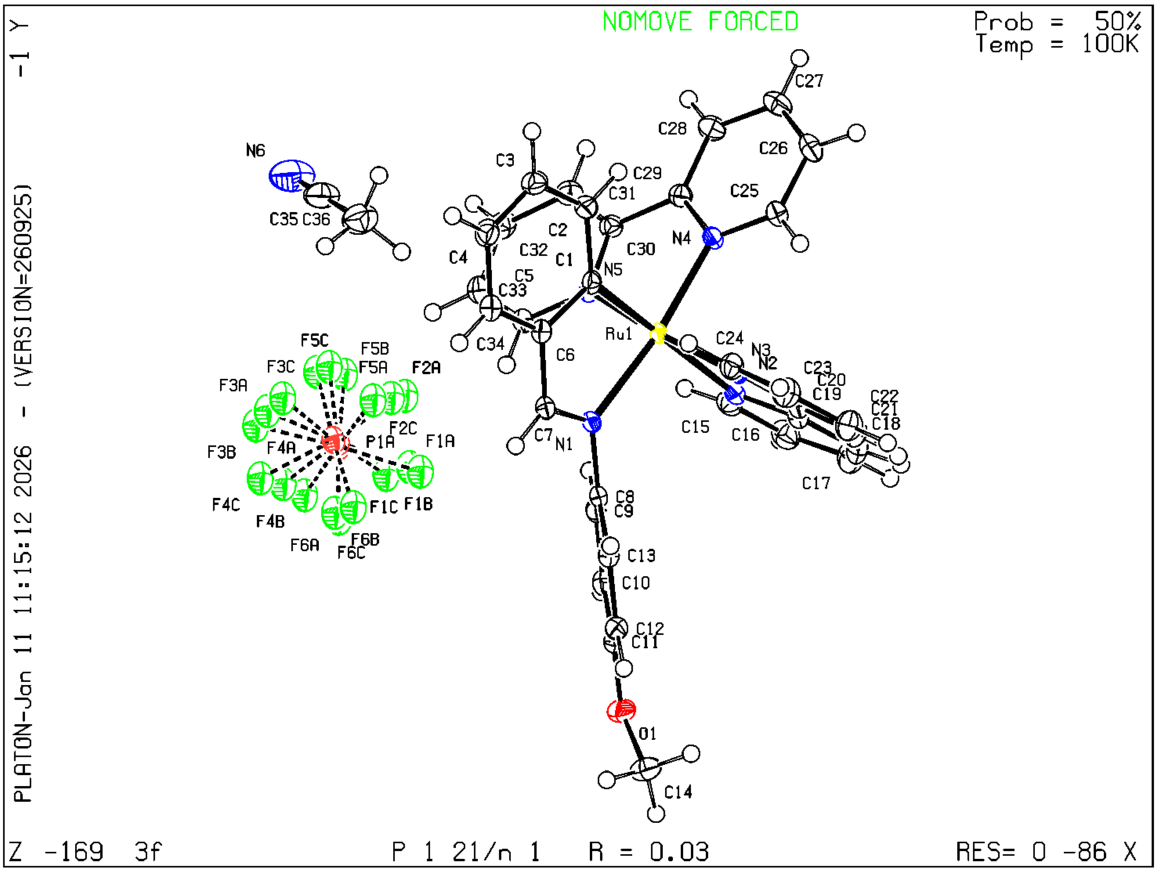

checkCIF/PLATON report

Structure factors have been supplied for datablock(s) 3h

THIS REPORT IS FOR GUIDANCE ONLY. IF USED AS PART OF A REVIEW PROCEDURE FOR PUBLICATION, IT SHOULD NOT REPLACE THE EXPERTISE OF AN EXPERIENCED CRYSTALLOGRAPHIC REFEREE.

No syntax errors found.   CIF dictionary   Interpreting this report

Datablock: 3h

Bond precision:	C-C = 0.0033 A		Wavelength=1.54184
Cell:	a=13.0457(1)	b=11.0904(1)	c=23.6918(1)
	alpha=90	beta=99.687(1)	gamma=90
Temperature:	100 K		

	Calculated	Reported
Volume	3378.90(4)	3378.90(4)
Space group	P 21/n	P 1 21/n 1
Hall group	-P 2yn	-P 2yn
Moiety formula	C33 H25 N6 O2 Ru, F6 P, C2 H3 N	C33 H25 N6 O2 Ru, F6 P, C2 H3 N
Sum formula	C35 H28 F6 N7 O2 P Ru	C35 H28 F6 N7 O2 P Ru
Mr	824.68	824.68
Dx,g cm-3	1.621	1.621
Z	4	4
Mu (mm-1)	4.895	4.895
F000	1664.0	1664.0
F000'	1670.73	
h,k,lmax	16,14,30	16,14,30
Nref	7373	7360
Tmin,Tmax	0.241,0.584	0.328,1.000
Tmin'	0.069	

Correction method= # Reported T Limits: Tmin=0.328 Tmax=1.000
AbsCorr = GAUSSIAN

Data completeness= 0.998 Theta(max)= 80.007

R(reflections)= 0.0328(7230) wR2(reflections)= 0.0835(7360)

S = 1.058 Npar= 486

The following ALERTS were generated. Each ALERT has the format
test-name_ALERT_alert-type_alert-level.
Click on the hyperlinks for more details of the test.

Alert level C

```
PLAT911_ALERT_3_C Missing FCF Refl Between Thmin & STh/L=    0.600          2 Report
                 2  5  0,   7  6  1,
```

Alert level G

```
PLAT002_ALERT_2_G Number of Distance or Angle Restraints on AtSite         21 Note
PLAT142_ALERT_4_G s.u. on b - Axis Small or Missing .............     0.00010 Ang.
PLAT143_ALERT_4_G s.u. on c - Axis Small or Missing .............     0.00010 Ang.
PLAT153_ALERT_1_G The s.u.'s on the Cell Axes   are Equal ..(Note)     0.0001 Ang.
PLAT171_ALERT_4_G The CIF-Embedded .res File Contains EADP Records          2 Report
PLAT176_ALERT_4_G The CIF-Embedded .res File Contains SADI Records          2 Report
PLAT191_ALERT_3_G A Non-default SADI Restraint Value has been used     0.0400 Report
PLAT302_ALERT_4_G Anion/Solvent/Minor-Residue Disorder (Resd    2)       100% Note
PLAT302_ALERT_4_G Anion/Solvent/Minor-Residue Disorder (Resd    3)       100% Note
PLAT302_ALERT_4_G Anion/Solvent/Minor-Residue Disorder (Resd    4)       100% Note
PLAT304_ALERT_4_G Non-Integer Number of Atoms in ..... (Resd    2)       2.74 Check
PLAT304_ALERT_4_G Non-Integer Number of Atoms in ..... (Resd    3)       2.32 Check
PLAT304_ALERT_4_G Non-Integer Number of Atoms in ..... (Resd    4)       1.93 Check
PLAT432_ALERT_2_G Short Inter X...Y Contact  F3B      ..C7       .       2.87 Ang.
                                                     x,y,z  =      1_555 Check
PLAT432_ALERT_2_G Short Inter X...Y Contact  F4A      ..C22      .       2.96 Ang.
                                         3/2-x,1/2+y,1/2-z  =      2_655 Check
PLAT432_ALERT_2_G Short Inter X...Y Contact  F5C      ..C7       .       2.83 Ang.
                                                     x,y,z  =      1_555 Check
PLAT860_ALERT_3_G Number of Least-Squares Restraints ............         784 Note
PLAT912_ALERT_4_G Missing # of FCF Reflections Above STh/L=  0.600         11 Note
PLAT969_ALERT_5_G The 'Henn et al.' R-Factor-gap value ..........       3.679 Note
              Predicted wR2: Based on SigI**2  2.27 or SHELX Weight  7.89
PLAT978_ALERT_2_G Number C-C Bonds with Positive Residual Density.          2 Info
```

---

0 **ALERT level A** = Most likely a serious problem - resolve or explain
0 **ALERT level B** = A potentially serious problem, consider carefully
1 **ALERT level C** = Check. Ensure it is not caused by an omission or oversight
20 **ALERT level G** = General information/check it is not something unexpected

1 ALERT type 1 CIF construction/syntax error, inconsistent or missing data
5 ALERT type 2 Indicator that the structure model may be wrong or deficient
3 ALERT type 3 Indicator that the structure quality may be low
11 ALERT type 4 Improvement, methodology, query or suggestion
1 ALERT type 5 Informative message, check

It is advisable to attempt to resolve as many as possible of the alerts in all categories. Often the minor alerts point to easily fixed oversights, errors and omissions in your CIF or refinement strategy, so attention to these fine details can be worthwhile. It is up to the individual to critically assess their own results and, if necessary, seek expert advice.

PLATON version of 26/09/2025; check.def file version of 20/09/2025

duplicate check

No duplication found

Datablock 3h - ellipsoid plot

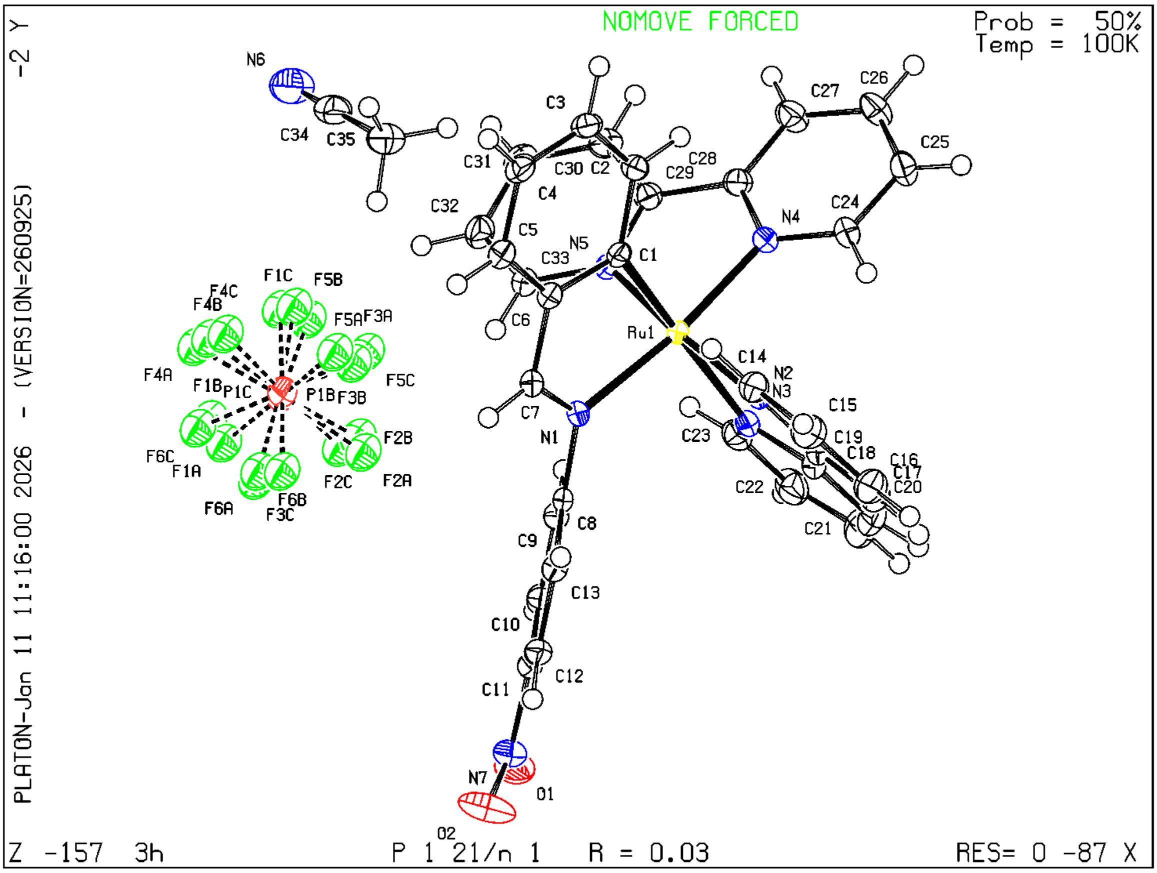

checkCIF/PLATON report

Structure factors have been supplied for datablock(s) 3j

THIS REPORT IS FOR GUIDANCE ONLY. IF USED AS PART OF A REVIEW PROCEDURE FOR PUBLICATION, IT SHOULD NOT REPLACE THE EXPERTISE OF AN EXPERIENCED CRYSTALLOGRAPHIC REFEREE.

No syntax errors found. CIF dictionary Interpreting this report

Datablock: 3j

Bond precision:	C-C = 0.0034 A		Wavelength=1.54184
Cell:	a=12.1502(1)	b=13.1069(1)	c=20.4987(1)
	alpha=90	beta=94.989(1)	gamma=90
Temperature:	100 K		

	Calculated	Reported
Volume	3252.08(4)	3252.08(4)
Space group	P 21/n	P 1 21/n 1
Hall group	-P 2yn	-P 2yn
Moiety formula	C31 H23 F N5 Ru S, F6 P, C2 H3 N	C31 H23 F N5 Ru S, F6 P, C2 H3 N
Sum formula	C33 H26 F7 N6 P Ru S	C33 H26 F7 N6 P Ru S
Mr	803.70	803.70
Dx,g cm-3	1.641	1.641
Z	4	4
Mu (mm-1)	5.640	5.640
F000	1616.0	1616.0
F000'	1623.75	
h,k,lmax	15,16,26	15,16,26
Nref	7105	7076
Tmin,Tmax	0.122,0.165	0.080,0.912
Tmin'	0.022	

Correction method= # Reported T Limits: Tmin=0.080 Tmax=0.912
AbsCorr = GAUSSIAN

Data completeness= 0.996 Theta(max)= 80.000

R(reflections)= 0.0333(7005) wR2(reflections)= 0.0810(7076)

S = 1.118 Npar= 477

The following ALERTS were generated. Each ALERT has the format
test-name_ALERT_alert-type_alert-level.
Click on the hyperlinks for more details of the test.

Alert level C

```
PLAT906_ALERT_3_C Large K Value in the Analysis of Variance ......      2.132 Check
```

Alert level G

```
PLAT002_ALERT_2_G Number of Distance or Angle Restraints on AtSite         20 Note
PLAT003_ALERT_2_G Number of Uiso or U(i,j)  Restrained non-H-Atoms          4 Report
PLAT142_ALERT_4_G s.u. on b - Axis Small or Missing .............    0.00010 Ang.
PLAT143_ALERT_4_G s.u. on c - Axis Small or Missing .............    0.00010 Ang.
PLAT153_ALERT_1_G The s.u.'s on the Cell Axes   are Equal ..(Note)     0.0001 Ang.
PLAT171_ALERT_4_G The CIF-Embedded .res File Contains EADP Records         10 Report
PLAT176_ALERT_4_G The CIF-Embedded .res File Contains SADI Records         22 Report
PLAT186_ALERT_4_G The CIF-Embedded .res File Contains ISOR Records          2 Report
PLAT191_ALERT_3_G A Non-default SADI Restraint Value has been used     0.0400 Report
PLAT191_ALERT_3_G A Non-default SADI Restraint Value has been used     0.0400 Report
PLAT191_ALERT_3_G A Non-default SADI Restraint Value has been used     0.0400 Report
PLAT191_ALERT_3_G A Non-default SADI Restraint Value has been used     0.0400 Report
PLAT191_ALERT_3_G A Non-default SADI Restraint Value has been used     0.0400 Report
PLAT191_ALERT_3_G A Non-default SADI Restraint Value has been used     0.0400 Report
PLAT191_ALERT_3_G A Non-default SADI Restraint Value has been used     0.0400 Report
PLAT191_ALERT_3_G A Non-default SADI Restraint Value has been used     0.0400 Report
PLAT191_ALERT_3_G A Non-default SADI Restraint Value has been used     0.0400 Report
PLAT191_ALERT_3_G A Non-default SADI Restraint Value has been used     0.0400 Report
PLAT191_ALERT_3_G A Non-default SADI Restraint Value has been used     0.0400 Report
PLAT191_ALERT_3_G A Non-default SADI Restraint Value has been used     0.0400 Report
PLAT191_ALERT_3_G A Non-default SADI Restraint Value has been used     0.0400 Report
PLAT302_ALERT_4_G Anion/Solvent/Minor-Residue Disorder (Resd    2)       100% Note
PLAT302_ALERT_4_G Anion/Solvent/Minor-Residue Disorder (Resd    3)       100% Note
PLAT302_ALERT_4_G Anion/Solvent/Minor-Residue Disorder (Resd    4)       100% Note
PLAT302_ALERT_4_G Anion/Solvent/Minor-Residue Disorder (Resd    5)       100% Note
PLAT304_ALERT_4_G Non-Integer Number of Atoms in ..... (Resd    2)       4.76 Check
PLAT304_ALERT_4_G Non-Integer Number of Atoms in ..... (Resd    3)       2.24 Check
PLAT304_ALERT_4_G Non-Integer Number of Atoms in ..... (Resd    4)       3.98 Check
PLAT304_ALERT_4_G Non-Integer Number of Atoms in ..... (Resd    5)       2.02 Check
PLAT860_ALERT_3_G Number of Least-Squares Restraints ............          45 Note
PLAT912_ALERT_4_G Missing # of FCF Reflections Above STh/L=  0.600         28 Note
PLAT933_ALERT_2_G Number of HKL-OMIT Records in Embedded .res File          1 Note
               10  1  5,
PLAT969_ALERT_5_G The 'Henn et al.' R-Factor-gap value ..........       2.984 Note
              Predicted wR2: Based on SigI**2  2.71 or SHELX Weight  7.24
PLAT978_ALERT_2_G Number C-C Bonds with Positive Residual Density.          7 Info
```

```
 0 ALERT level A = Most likely a serious problem - resolve or explain
 0 ALERT level B = A potentially serious problem, consider carefully
 1 ALERT level C = Check. Ensure it is not caused by an omission or oversight
34 ALERT level G = General information/check it is not something unexpected

 1 ALERT type 1 CIF construction/syntax error, inconsistent or missing data
 4 ALERT type 2 Indicator that the structure model may be wrong or deficient
15 ALERT type 3 Indicator that the structure quality may be low
14 ALERT type 4 Improvement, methodology, query or suggestion
 1 ALERT type 5 Informative message, check
```

It is advisable to attempt to resolve as many as possible of the alerts in all categories. Often the minor alerts point to easily fixed oversights, errors and omissions in your CIF or refinement strategy, so attention to these fine details can be worthwhile. It is up to the individual to critically assess their own results and, if necessary, seek expert advice.

PLATON version of 26/09/2025; check.def file version of 20/09/2025

---

duplicate check

No duplication found

Datablock 3j - ellipsoid plot

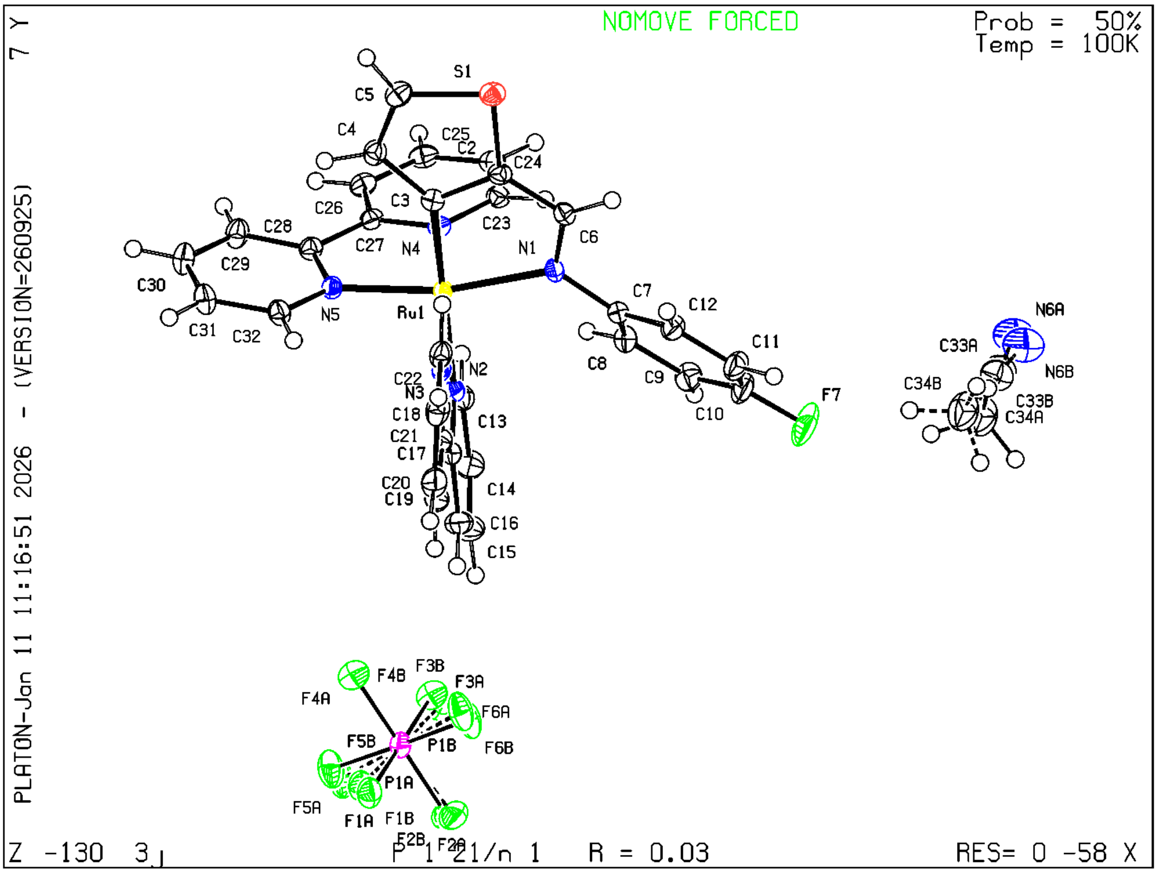

checkCIF/PLATON report

Structure factors have been supplied for datablock(s) 3l

THIS REPORT IS FOR GUIDANCE ONLY. IF USED AS PART OF A REVIEW PROCEDURE FOR PUBLICATION, IT SHOULD NOT REPLACE THE EXPERTISE OF AN EXPERIENCED CRYSTALLOGRAPHIC REFEREE.

No syntax errors found. CIF dictionary Interpreting this report

Datablock: 3l

Bond precision: C-C = 0.0019 A Wavelength=0.71073

Cell:	a=11.1999(2)	b=11.8657(2)	c=13.0164(2)
	alpha=89.344(1)	beta=75.814(1)	gamma=75.793(1)
Temperature:	100 K		

	Calculated	Reported
Volume	1623.58(5)	1623.58(5)
Space group	P -1	P-1
Hall group	-P 1	-P 1
Moiety formula	C32 H26 N5 O Ru S, F6 P, C2 H3 N	C32 H26 N5 O Ru S, F6 P, C2 H3 N
Sum formula	C34 H29 F6 N6 O P Ru S	C34 H29 F6 N6 O P Ru S
Mr	815.88	815.73
Dx,g cm-3	1.669	1.669
Z	2	2
Mu (mm-1)	0.671	0.671
F000	824.1	824.0
F000'	822.46	
h,k,lmax	17,18,20	17,18,20
Nref	12463	12446
Tmin,Tmax	0.777,0.891	0.503,0.566
Tmin'	0.740	

Correction method= # Reported T Limits: Tmin=0.503 Tmax=0.566
AbsCorr = MULTI-SCAN

Data completeness= 0.999   Theta(max)= 33.227

R(reflections)= 0.0258(11436)   wR2(reflections)= 0.0630(12446)

S = 1.033   Npar= 468

The following ALERTS were generated. Each ALERT has the format
test-name_ALERT_alert-type_alert-level.
Click on the hyperlinks for more details of the test.

Alert level C

```
PLAT911_ALERT_3_C Missing FCF Refl Between Thmin & STh/L=    0.600          4 Report
               -1  1  0,   1  1  0,  -2  1  1,   2  2  3,
```

Alert level G

```
PLAT002_ALERT_2_G Number of Distance or Angle Restraints on AtSite         21 Note
PLAT068_ALERT_1_G Reported F000 Differs from Calcd (or Missing)...     Please Check
PLAT154_ALERT_1_G The s.u.'s on the Cell Angles are Equal ..(Note)      0.001 Degree
PLAT171_ALERT_4_G The CIF-Embedded .res File Contains EADP Records          2 Report
PLAT176_ALERT_4_G The CIF-Embedded .res File Contains SADI Records         23 Report
PLAT191_ALERT_3_G A Non-default SADI Restraint Value has been used     0.0400 Report
PLAT191_ALERT_3_G A Non-default SADI Restraint Value has been used     0.0400 Report
PLAT191_ALERT_3_G A Non-default SADI Restraint Value has been used     0.0400 Report
PLAT191_ALERT_3_G A Non-default SADI Restraint Value has been used     0.0400 Report
PLAT191_ALERT_3_G A Non-default SADI Restraint Value has been used     0.0400 Report
PLAT191_ALERT_3_G A Non-default SADI Restraint Value has been used     0.0400 Report
PLAT191_ALERT_3_G A Non-default SADI Restraint Value has been used     0.0400 Report
PLAT191_ALERT_3_G A Non-default SADI Restraint Value has been used     0.0400 Report
PLAT191_ALERT_3_G A Non-default SADI Restraint Value has been used     0.0400 Report
PLAT191_ALERT_3_G A Non-default SADI Restraint Value has been used     0.0400 Report
PLAT191_ALERT_3_G A Non-default SADI Restraint Value has been used     0.0400 Report
PLAT191_ALERT_3_G A Non-default SADI Restraint Value has been used     0.0400 Report
PLAT191_ALERT_3_G A Non-default SADI Restraint Value has been used     0.0400 Report
PLAT191_ALERT_3_G A Non-default SADI Restraint Value has been used     0.0400 Report
PLAT191_ALERT_3_G A Non-default SADI Restraint Value has been used     0.0400 Report
PLAT191_ALERT_3_G A Non-default SADI Restraint Value has been used     0.0400 Report
PLAT232_ALERT_2_G Hirshfeld Test Diff (M-X)  Ru1      --N2       .        6.0 s.u.
PLAT232_ALERT_2_G Hirshfeld Test Diff (M-X)  Ru1      --N3       .        5.8 s.u.
PLAT232_ALERT_2_G Hirshfeld Test Diff (M-X)  Ru1      --N4       .        6.5 s.u.
PLAT232_ALERT_2_G Hirshfeld Test Diff (M-X)  Ru1      --N5       .        5.0 s.u.
PLAT302_ALERT_4_G Anion/Solvent/Minor-Residue Disorder (Resd    2)       100% Note
PLAT302_ALERT_4_G Anion/Solvent/Minor-Residue Disorder (Resd    3)       100% Note
PLAT302_ALERT_4_G Anion/Solvent/Minor-Residue Disorder (Resd    4)       100% Note
PLAT304_ALERT_4_G Non-Integer Number of Atoms in ..... (Resd    2)       2.42 Check
PLAT304_ALERT_4_G Non-Integer Number of Atoms in ..... (Resd    3)       2.64 Check
PLAT304_ALERT_4_G Non-Integer Number of Atoms in ..... (Resd    4)       1.95 Check
PLAT860_ALERT_3_G Number of Least-Squares Restraints ............         623 Note
PLAT910_ALERT_3_G Missing FCF Reflection(s) Below Theta(Min)[Deg]=       1.94 Note
                0  1  0,   0  0  1,
PLAT912_ALERT_4_G Missing # of FCF Reflections Above STh/L=  0.600         11 Note
PLAT933_ALERT_2_G Number of HKL-OMIT Records in Embedded .res File          3 Note
               -2  1  1,   1  1  0,   2  2  3,
```

```
PLAT969_ALERT_5_G The 'Henn et al.' R-Factor-gap value ...........      3.423 Note
              Predicted wR2: Based on SigI**2  1.84 or SHELX Weight  6.10
PLAT978_ALERT_2_G Number C-C Bonds with Positive Residual Density.        14 Info
```

```
   0 ALERT level A = Most likely a serious problem - resolve or explain
   0 ALERT level B = A potentially serious problem, consider carefully
   1 ALERT level C = Check. Ensure it is not caused by an omission or oversight
  37 ALERT level G = General information/check it is not something unexpected

   2 ALERT type 1 CIF construction/syntax error, inconsistent or missing data
   7 ALERT type 2 Indicator that the structure model may be wrong or deficient
  19 ALERT type 3 Indicator that the structure quality may be low
   9 ALERT type 4 Improvement, methodology, query or suggestion
   1 ALERT type 5 Informative message, check
```

It is advisable to attempt to resolve as many as possible of the alerts in all categories. Often the minor alerts point to easily fixed oversights, errors and omissions in your CIF or refinement strategy, so attention to these fine details can be worthwhile. It is up to the individual to critically assess their own results and, if necessary, seek expert advice.

PLATON version of 26/09/2025; check.def file version of 20/09/2025

---

duplicate check

No duplication found

Datablock 3l - ellipsoid plot

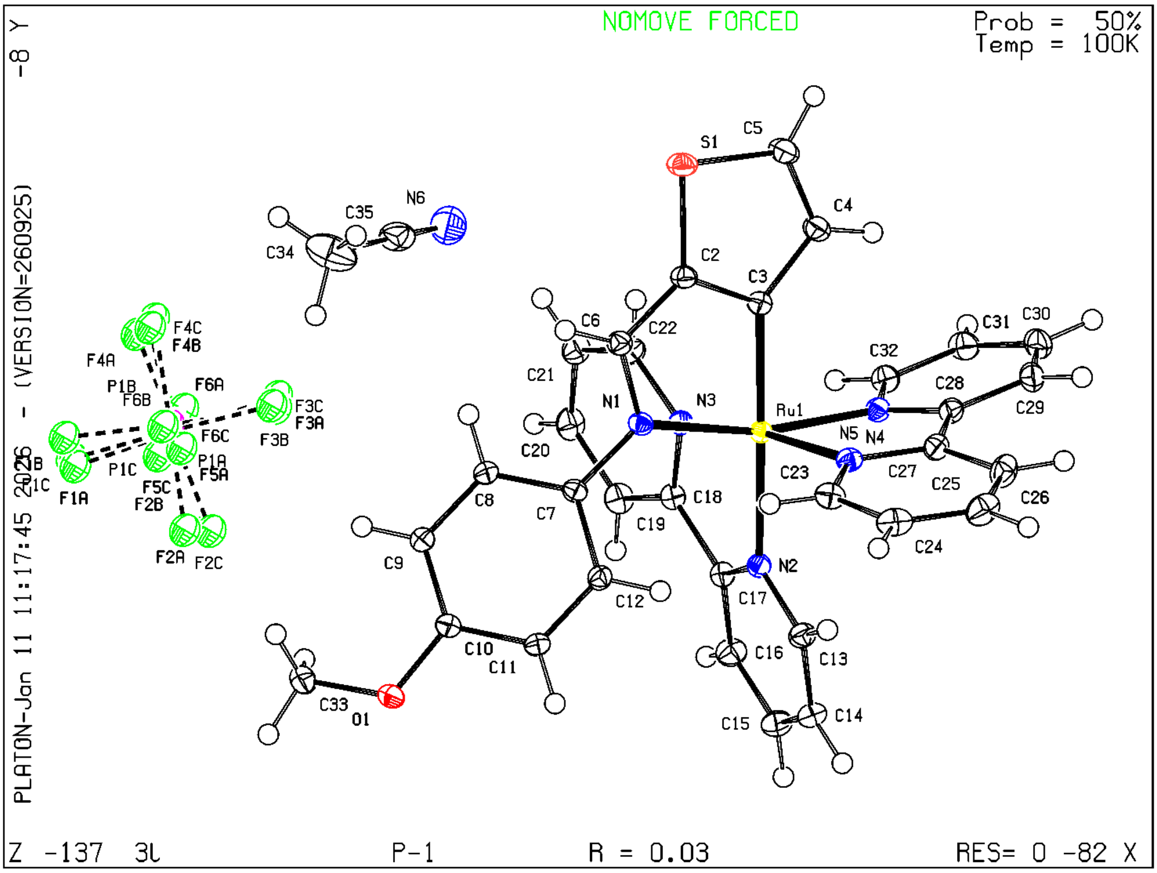

checkCIF/PLATON report

Structure factors have been supplied for datablock(s) 3m

THIS REPORT IS FOR GUIDANCE ONLY. IF USED AS PART OF A REVIEW PROCEDURE FOR PUBLICATION, IT SHOULD NOT REPLACE THE EXPERTISE OF AN EXPERIENCED CRYSTALLOGRAPHIC REFEREE.

No syntax errors found. CIF dictionary Interpreting this report

Datablock: 3m

Bond precision:	C-C = 0.0027 A		Wavelength=0.71073
Cell:	a=12.8277(5)	b=13.2553(5)	c=13.3198(5)
	alpha=118.386(1)	beta=114.061(1)	gamma=91.943(1)
Temperature:	100 K		

	Calculated	Reported
Volume	1744.75(12)	1744.75(12)
Space group	P -1	P-1
Hall group	-P 1	-P 1
Moiety formula	C34 H28 N5 O2 Ru S, F6 P [+ solvent]	C34 H28 N5 O2 Ru S, 1(F6 P1), 0.625[CH3CN]
Sum formula	C34 H28 F6 N5 O2 P Ru S [+ solvent]	C35.24 H29.88 F6 N5.62 O2 P Ru S
Mr	816.63	842.19
Dx,g cm-3	1.554	1.603
Z	2	2
Mu (mm-1)	0.626	0.629
F000	823.9	851.0
F000’	822.26	
h,k,lmax	19,20,20	19,20,20
Nref	13361	13338
Tmin,Tmax	0.853,0.927	0.475,0.566
Tmin’	0.839	

Correction method= # Reported T Limits: Tmin=0.475 Tmax=0.566
AbsCorr = MULTI-SCAN

Data completeness= 0.998 Theta(max)= 33.194

R(reflections)= 0.0321(12367) wR2(reflections)= 0.0793(13338)

S = 1.042 Npar= 524

The following ALERTS were generated. Each ALERT has the format
test-name_ALERT_alert-type_alert-level.
Click on the hyperlinks for more details of the test.

Alert level C

```
PLAT220_ALERT_2_C NonSolvent   Resd 1  C   Ueq(max)/Ueq(min) Range        3.3 Ratio
PLAT910_ALERT_3_C Missing FCF Reflection(s) Below Theta(Min)[Deg]=       2.18 Note
                1  0  0,   0  1  0,   0 -1  1,  -1  0  1,   0  0  1,
PLAT911_ALERT_3_C Missing FCF Refl Between Thmin & STh/L=    0.600          3 Report
               -1 -1  1,   2 -1  1,  -2  0  1,
```

Alert level G

```
FORMU01_ALERT_2_G  There is a discrepancy between the atom counts in the
            _chemical_formula_sum and the formula from the _atom_site* data.
            Atom count from _chemical_formula_sum:C35.24 H29.88 F6 N5.62 O2 P1 Ru1
            Atom count from the _atom_site data:  C34 H28 F6.001401 N5 O2 P1.00439
CELLZ01_ALERT_1_G Difference between formula and atom_site contents detected.
CELLZ01_ALERT_1_G ALERT: Large difference may be due to a
            symmetry error - see SYMMG tests
           From the CIF: _cell_formula_units_Z    2
           From the CIF: _chemical_formula_sum  C35.24 H29.88 F6 N5.62 O2 P Ru S
           TEST: Compare cell contents of formula and atom_site data

           atom    Z*formula  cif sites diff
           C         70.48     68.00    2.48
           H         59.76     56.00    3.76
           F         12.00     11.99    0.01
           N         11.24     10.00    1.24
           O          4.00      4.00    0.00
           P          2.00      2.00    0.00
           Ru         2.00      2.00    0.00
           S          2.00      2.00    0.00
PLAT002_ALERT_2_G Number of Distance or Angle Restraints on AtSite         51 Note
PLAT041_ALERT_1_G Calc. and Reported SumFormula    Strings  Differ     Please Check
              Calc: C34 H28 F6 N5 O2 P Ru S
              Rep.: C35.24 H29.88 F6 N5.62 O2 P Ru S
PLAT042_ALERT_1_G Calc. and Reported MoietyFormula Strings  Differ     Please Check
              Calc: C34 H28 N5 O2 Ru S, F6 P
              Rep.: C34 H28 N5 O2 Ru S, 1(F6 P1), 0.625[CH3CN]
PLAT154_ALERT_1_G The s.u.'s on the Cell Angles are Equal ..(Note)      0.001 Degree
PLAT171_ALERT_4_G The CIF-Embedded .res File Contains EADP Records         13 Report
PLAT176_ALERT_4_G The CIF-Embedded .res File Contains SADI Records         29 Report
```

```
PLAT191_ALERT_3_G A Non-default SADI Restraint Value has been used     0.0400 Report
PLAT191_ALERT_3_G A Non-default SADI Restraint Value has been used     0.0400 Report
PLAT191_ALERT_3_G A Non-default SADI Restraint Value has been used     0.0400 Report
PLAT191_ALERT_3_G A Non-default SADI Restraint Value has been used     0.0400 Report
PLAT191_ALERT_3_G A Non-default SADI Restraint Value has been used     0.0400 Report
PLAT191_ALERT_3_G A Non-default SADI Restraint Value has been used     0.0400 Report
PLAT191_ALERT_3_G A Non-default SADI Restraint Value has been used     0.0400 Report
PLAT191_ALERT_3_G A Non-default SADI Restraint Value has been used     0.0400 Report
PLAT191_ALERT_3_G A Non-default SADI Restraint Value has been used     0.0400 Report
PLAT191_ALERT_3_G A Non-default SADI Restraint Value has been used     0.0400 Report
PLAT191_ALERT_3_G A Non-default SADI Restraint Value has been used     0.0400 Report
PLAT191_ALERT_3_G A Non-default SADI Restraint Value has been used     0.0400 Report
PLAT191_ALERT_3_G A Non-default SADI Restraint Value has been used     0.0400 Report
PLAT191_ALERT_3_G A Non-default SADI Restraint Value has been used     0.0400 Report
PLAT191_ALERT_3_G A Non-default SADI Restraint Value has been used     0.0400 Report
PLAT191_ALERT_3_G A Non-default SADI Restraint Value has been used     0.0400 Report
PLAT301_ALERT_3_G Main Residue  Disorder .............(Resd    1)        26% Note
PLAT302_ALERT_4_G Anion/Solvent/Minor-Residue Disorder (Resd    2)       100% Note
PLAT302_ALERT_4_G Anion/Solvent/Minor-Residue Disorder (Resd    3)       100% Note
PLAT302_ALERT_4_G Anion/Solvent/Minor-Residue Disorder (Resd    4)       100% Note
PLAT302_ALERT_4_G Anion/Solvent/Minor-Residue Disorder (Resd    5)       100% Note
PLAT304_ALERT_4_G Non-Integer Number of Atoms in ..... (Resd    2)       2.60 Check
PLAT304_ALERT_4_G Non-Integer Number of Atoms in ..... (Resd    3)       1.38 Check
PLAT304_ALERT_4_G Non-Integer Number of Atoms in ..... (Resd    4)       2.16 Check
PLAT304_ALERT_4_G Non-Integer Number of Atoms in ..... (Resd    5)       0.86 Check
PLAT432_ALERT_2_G Short Inter X...Y Contact  S1       ..C33B     .       3.15 Ang.
                                                1-x,1-y,1-z  =      2_666 Check
PLAT605_ALERT_4_G Largest Solvent Accessible VOID in the Structure        122 A**3
PLAT811_ALERT_5_G No ADDSYM Analysis: Too Many Excluded Atoms ....          ! Info
PLAT860_ALERT_3_G Number of Least-Squares Restraints ............        1346 Note
PLAT868_ALERT_4_G ALERTS Due to the Use of _smtbx_masks Suppressed          ! Info
PLAT912_ALERT_4_G Missing # of FCF Reflections Above STh/L=  0.600         15 Note
PLAT969_ALERT_5_G The 'Henn et al.' R-Factor-gap value ..........       3.914 Note
             Predicted wR2: Based on SigI**2  2.03 or SHELX Weight  7.61
PLAT978_ALERT_2_G Number C-C Bonds with Positive Residual Density.          8 Info
```

```
   0 ALERT level A = Most likely a serious problem - resolve or explain
   0 ALERT level B = A potentially serious problem, consider carefully
   3 ALERT level C = Check. Ensure it is not caused by an omission or oversight
  42 ALERT level G = General information/check it is not something unexpected

   5 ALERT type 1 CIF construction/syntax error, inconsistent or missing data
   5 ALERT type 2 Indicator that the structure model may be wrong or deficient
  20 ALERT type 3 Indicator that the structure quality may be low
  13 ALERT type 4 Improvement, methodology, query or suggestion
   2 ALERT type 5 Informative message, check
```

It is advisable to attempt to resolve as many as possible of the alerts in all categories. Often the minor alerts point to easily fixed oversights, errors and omissions in your CIF or refinement strategy, so attention to these fine details can be worthwhile. It is up to the individual to critically assess their own results and, if necessary, seek expert advice.

PLATON version of 26/09/2025; check.def file version of 20/09/2025

duplicate check

No duplication found

Datablock 3m - ellipsoid plot

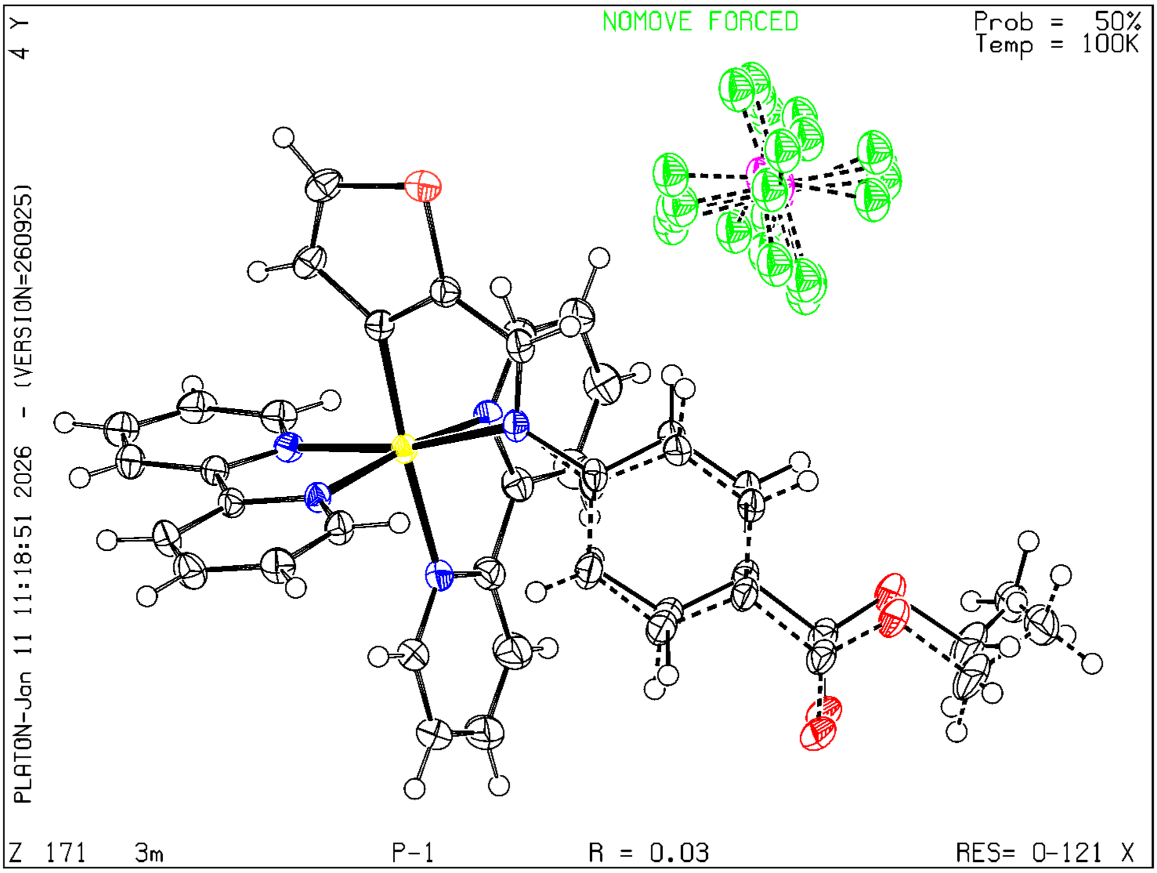

checkCIF/PLATON report

Structure factors have been supplied for datablock(s) 3o

THIS REPORT IS FOR GUIDANCE ONLY. IF USED AS PART OF A REVIEW PROCEDURE FOR PUBLICATION, IT SHOULD NOT REPLACE THE EXPERTISE OF AN EXPERIENCED CRYSTALLOGRAPHIC REFEREE.

No syntax errors found. CIF dictionary Interpreting this report

Datablock: 3o

Bond precision: C-C = 0.0031 A Wavelength=0.71073

Cell:	a=9.9059(4)	b=12.5785(5)	c=13.7546(6)
	alpha=80.180(2)	beta=77.243(2)	gamma=70.897(2)
Temperature:	100 K		

	Calculated	Reported
Volume	1570.48(11)	1570.48(11)
Space group	P -1	P-1
Hall group	-P 1	-P 1
Moiety formula	C32 H23 F3 N5 Ru S, F6 P	C32 H23 F3 N5 Ru S, F6 P
Sum formula	C32 H23 F9 N5 P Ru S	C32 H23 F9 N5 P Ru S
Mr	812.65	812.65
Dx,g cm-3	1.719	1.719
Z	2	2
Mu (mm-1)	0.704	0.704
F000	812.0	812.0
F000'	810.39	
h,k,lmax	15,19,21	15,19,21
Nref	12172	12017
Tmin,Tmax	0.820,0.877	0.476,0.566
Tmin'	0.670	

Correction method= # Reported T Limits: Tmin=0.476 Tmax=0.566
AbsCorr = MULTI-SCAN

```
Data completeness= 0.987          Theta(max)= 33.324

R(reflections)= 0.0403( 10140)                     wR2(reflections)=
                                                    0.0982( 12017)
S = 1.039                  Npar= 442
```

The following ALERTS were generated. Each ALERT has the format
test-name_ALERT_alert-type_alert-level.
Click on the hyperlinks for more details of the test.

Alert level C

```
PLAT244_ALERT_4_C Low    'Solvent' Ueq as Compared to Neighbors of         P1 Check
PLAT911_ALERT_3_C Missing FCF Refl Between Thmin & STh/L=    0.600          2 Report
                1  1  0,   0 -1  1,
PLAT975_ALERT_2_C Check Calcd Resid. Dens.  1.07Ang From N3      .       0.42 eA-3
```

Alert level G

```
PLAT154_ALERT_1_G The s.u.'s on the Cell Angles are Equal ..(Note)      0.002 Degree
PLAT242_ALERT_2_G Low    'MainMol' Ueq as Compared to Neighbors of        C33 Check
PLAT910_ALERT_3_G Missing FCF Reflection(s) Below Theta(Min)[Deg]=       1.72 Note
                0  0  1,
PLAT912_ALERT_4_G Missing # of FCF Reflections Above STh/L=  0.600        151 Note
PLAT969_ALERT_5_G The 'Henn et al.' R-Factor-gap value ..........      3.264 Note
              Predicted wR2: Based on SigI**2  3.01 or SHELX Weight  9.45
PLAT978_ALERT_2_G Number C-C Bonds with Positive Residual Density.          5 Info
```

---

0 **ALERT level A** = Most likely a serious problem - resolve or explain
0 **ALERT level B** = A potentially serious problem, consider carefully
3 **ALERT level C** = Check. Ensure it is not caused by an omission or oversight
6 **ALERT level G** = General information/check it is not something unexpected

1 ALERT type 1 CIF construction/syntax error, inconsistent or missing data
3 ALERT type 2 Indicator that the structure model may be wrong or deficient
2 ALERT type 3 Indicator that the structure quality may be low
2 ALERT type 4 Improvement, methodology, query or suggestion
1 ALERT type 5 Informative message, check

It is advisable to attempt to resolve as many as possible of the alerts in all categories. Often the minor alerts point to easily fixed oversights, errors and omissions in your CIF or refinement strategy, so attention to these fine details can be worthwhile. It is up to the individual to critically assess their own results and, if necessary, seek expert advice.

PLATON version of 26/09/2025; check.def file version of 20/09/2025

---

duplicate check

No duplication found

Datablock 3o - ellipsoid plot

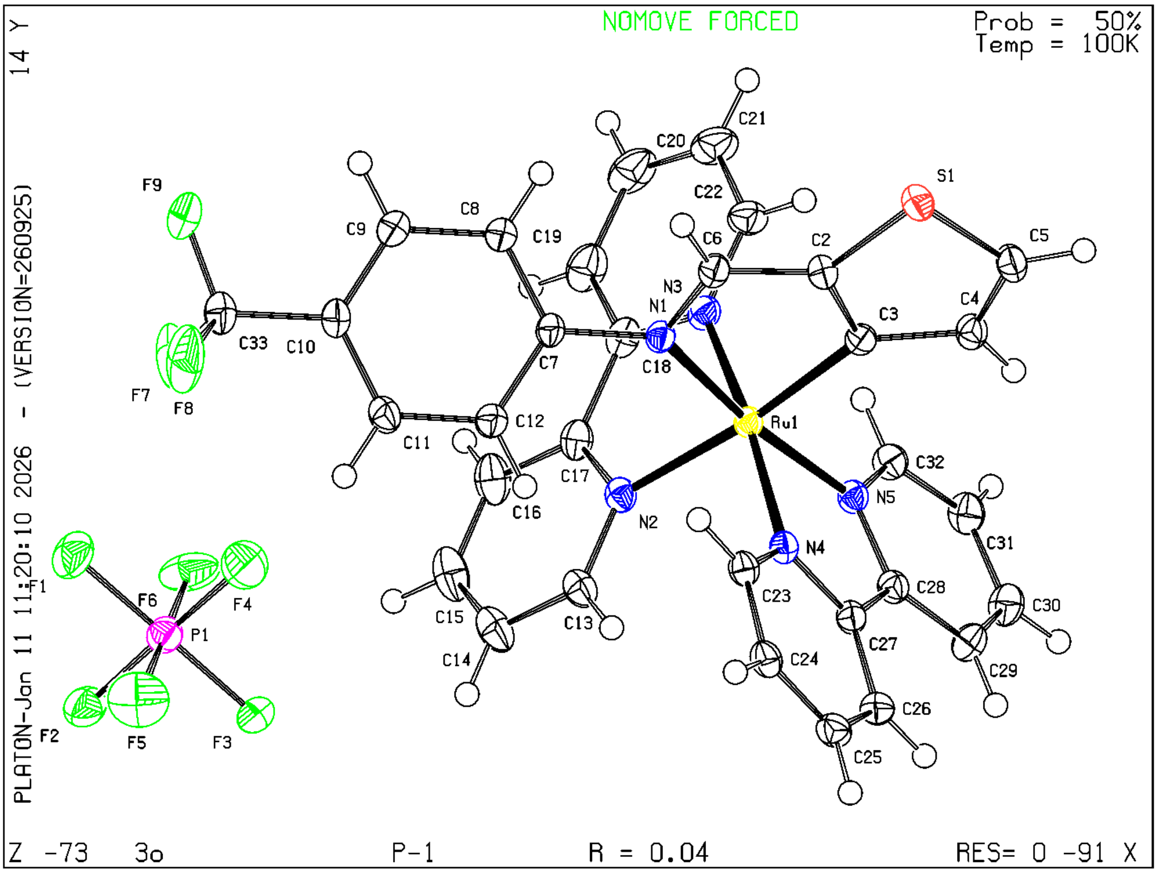

Supplement: Supplementary file 1 [file molecules-31-00315-s001.zip › checkcif_all.pdf]
