# Supplementary material for: Cycloruthenated Imines: A Step into the Nanomolar Region
Source: Molecules. 2026 Jan 16;31(2):315. doi: 10.3390/molecules31020315 (PMC12844335; doi:10.3390/molecules31020315)
Supplement: Supplementary file 1 [file molecules-31-00315-s001.zip › Vasiliev_SI_Rev3.pdf]

Supporting Information for

Cycloruthenated Imines: A Step into the Nanomolar Region

Arsenii A. Vasil'ev [1,2], Ivan I. Troshin [1,3], Pavel G. Shangin [1], Ksenia M. Voroshilkina [2], Ilya A. Shutkov [2], Alexey A. Nazarov [2] and Aleksei V. Medved'ko [1,*]

*lexeym@gmail.com

1 N.D. Zelinsky Institute of Organic Chemistry RAS, 47 Leninsky Prospect, 119991 Moscow, Russia

2 Chemistry Department, M.V. Lomonosov Moscow State University, Leninskie Gory 1-3, 119234 Moscow, Russia

3 Department of Chemistry and Technology of Biologically Active Compounds, Medicinal and Organic Chemistry, Institute of Fine Chemical Technologies, MIREA-Russian Technological University, 86 Vernadsky Avenue, 119571 Moscow, Russia

Table of Contents:

	**Experimental details**	**2-4**
	**NMR spectra**	**5-34**
	**Crystallography**	**35-52**
	**CV curves**	**53-67**
	**Stability in PBS**	**68-70**

Experimental details

Single crystal X-ray crystallographic data and refinement details.

X-ray diffraction data for **2a**, **3e**, **3m** and **3o** were collected at 100K on a Bruker Quest D8 diffractometer equipped with a Photon-III area-detector (graphite monochromator, shutterless φ- and ω-scan technique), using Mo K -radiation. The intensity data were integrated by the SAINT program [1] and were corrected for absorption and decay using SADABS [2]. The structure was solved by direct methods using SHELXT [3] and refined on F^2 using SHELXL-2018 [4] in the OLEX2 program [5]. All non-hydrogen atoms were refined with individual anisotropic displacement parameters. All hydrogen atoms were placed in ideal calculated positions and refined as riding atoms with relative isotropic displacement parameters. The SHELXTL program suite [1] was used for molecular graphics.

X-ray diffraction data were collected at 100K on a four-circle Rigaku Synergy S diffractometer equipped with a HyPix600HE area-detector (kappa geometry, shutterless ω-scan technique), using monochromatized Cu K -radiation (for compounds **1n**, **2b**, **2d**, **2f-2l**, **2n**, **3a**, **3b**, **3f**, **3h**, **3j**) and using monochromatized Mo K -radiation (for compounds **2e**, **3c**). The intensity data were integrated and corrected for absorption and decay by the CrysAlisPro program [6]. The structure was solved by direct methods using SHELXT [3] and refined on F^2 using SHELXL-2018[4] in the OLEX2 program.[5] All non-hydrogen atoms were refined with individual anisotropic displacement parameters. All hydrogen atoms were placed in ideal calculated positions and refined as riding atoms with relative isotropic displacement parameters. The SHELXTL program suite [1] was used for molecular graphics.

The structures of **2a**, **2b**, **3c** and **3m** contained unresolved/highly disordered acetonitrile and ether molecules in the crystal channels, which were removed by the SQUEEZE method [7] implemented in the OLEX2 program [5].

Cyclic voltammetry

The electrochemical oxidation and reduction behavior of the compounds under discussion was investigated by cyclic voltammetry using an IPC-Pro-MF potentiostat from Econix. The preparation of solutions and all measurements were performed in an argon-filled glovebox at a water and oxygen level of no more than 0.1 ppm. Before use, acetonitrile (HPLC grade, Acros) with an initial water content of no more than 100 ppm was stored over molecular sieves (4 Å) pre-dried under oil pump vacuum at 200-250 °C for 4 h. Bu_4NPF_6 (Sigma Aldrich) was dried under oil pump vacuum at 80 °C for 4 h. The water content in the 0.1 M Bu_4NPF_6/acetonitrile system used did not exceed 20 ppm after that, which was monitored by Karl Fischer titration using a Mettler-Toledo C10SD titrator. The compounds dissolved in 5 ml of the background electrolyte were electrochemically analyzed in a standard conical three-electrode glass cell. The working electrode was a 1.7 mm diameter glassy carbon disk electrode placed in PTFE. Before use, it was polished with sandpaper and GOI paste until a mirror shine was achieved. The auxiliary electrode was a platinum wire pre-calcined in a gas burner flame. The potentials of the processes under study were measured relative to a silver wire coated with AgCl (achieved by galvanostatic anodizing in a 5% hydrochloric acid solution) separated from the main solution by an electrochemical bridge filled with an auxiliary electrolyte. Separately, under similar conditions, the oxidation curves of ferrocene were recorded, and the given values were corrected according to the oxidation potential of the latter.

Cells and MTT assay.

The human A2780 ovarian adenocarcinoma, A2780cis cisplatin resistant ovarian adenocarcinoma, HEK293 human embryonic kidney cell lines were obtained from the European collection of authenticated cell cultures (ECACC; Salisbury, UK). Cells were grown in a RPMI 1640 (Gibco™, Ireland) cell medium (A2780, A2780cis), or DMEM (Gibco™, Ireland) cell medium (HEK293) supplemented with 10% fetal bovine serum (Gibco™, Brazil). The cells were cultured in an incubator at 37 °C in a humidified 5% CO2 atmosphere and were subcultured two times a week. The antiproliferative activity was studied by MTT assays as published previously [8]

1. Bruker. APEX-III. *Bruker AXS Inc.*, Madison, Wisconsin, USA, **2019**.
2. Krause, L.; Herbst-Irmer, R.; Sheldrick, G. M.; Stalke, D. Comparison of silver and molybdenum microfocus X-ray sources for single-crystal structure determination. *J. Appl. Cryst.* **2015**, 48, 3–10. http://doi.org/10.1107/S1600576714022985
3. Sheldrick, G. M. SHELXT - Integrated space-group and crystal-structure determination. *Acta Cryst.* **2015**, A71, 3-8. http://doi.org/10.1107/S2053273314026370
4. Sheldrick, G. M. Crystal structure refinement with SHELXL. *Acta Cryst.* **2015**, C71, 3-8. http://doi.org/10.1107/S2053229614024218
5. Dolomanov O.V.; Bourhis L.J.; Gildea R.J.; Howard J.A.K.; Puschmann H. OLEX2: a complete structure solution, refinement and analysis program. *J. Appl. Cryst.* **2009**, 42(2), 229-341. http://doi.org/10.1107/S0021889808042726
6. CrysAlisPro. Version 1.171.41. *Rigaku Oxford Diffraction*, **2021**.
7. Spek A. L., *Acta Cryst.*, 2015, **C71**, 9-18, http://dx.doi.org/10.1107/S2053229614024929

8. Nosova, Y.N.; Foteeva, L.S.; Zenin, I. V.; Fetisov, T.I.; Kirsanov, K.I.; Yakubovskaya, M.G.; Antonenko, T.A.; Tafeenko, V.A.; Aslanov, L.A.; Lobas, A.A.; et al. Enhancing the Cytotoxic Activity of Anticancer Pt IV Complexes by Introduction of Lonidamine as an Axial Ligand. *Eur. J. Inorg. Chem.* **2017**, *2017*, 1785–1791, http://dx.doi.org/10.1002/ejic.201600857

Preparation of starting materials

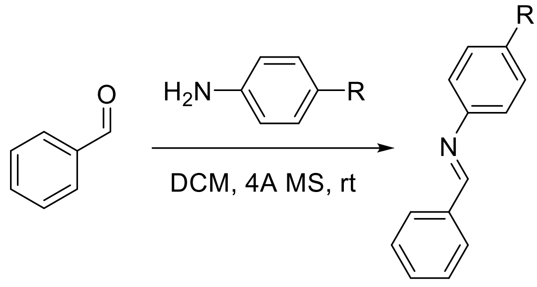

1a (R = H): 92%
1b (R = F): 94%
1c (R = Me): 99%
1d (R = Et): 96%
1e (R = tBu): 96%
1f (R = OMe): 95%
1g (R = COOEt): 91%
1h (R = NO_2): 90%
1i (R = CF_3): 90%

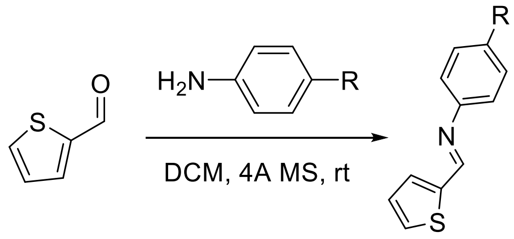

1j (R = F): 94%
1k (R = Me): 99%
1l (R = OMe): 96%
1m (R = COOEt): 94%
1n (R = NO_2): 85%
1o (R = CF_3): 77%

Scheme S1. Preparation of starting imines.

*General procedure for imines **1a**-**1o**.*

Equimolar amounts of corresponding aldehyde (2.5 mmol) and aniline (2.5 mmol) were stirred in dry DCM (7.8 ml) with 2.5 g of 4 Å molecular sieves (1 g/1 mmol) for 72 h in argon atmosphere, reaction mixture was filtered and evaporated. Product used without further purification.

NMR spectra

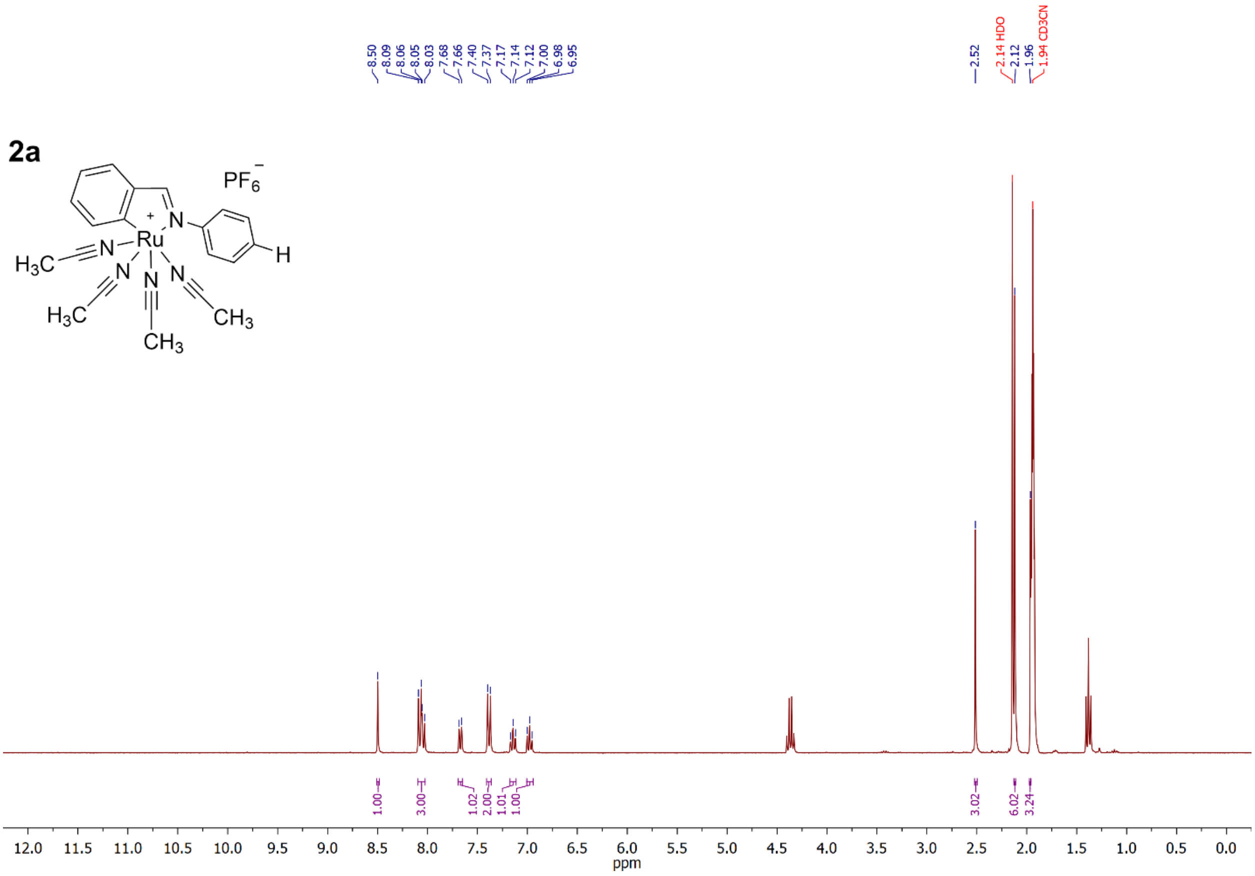

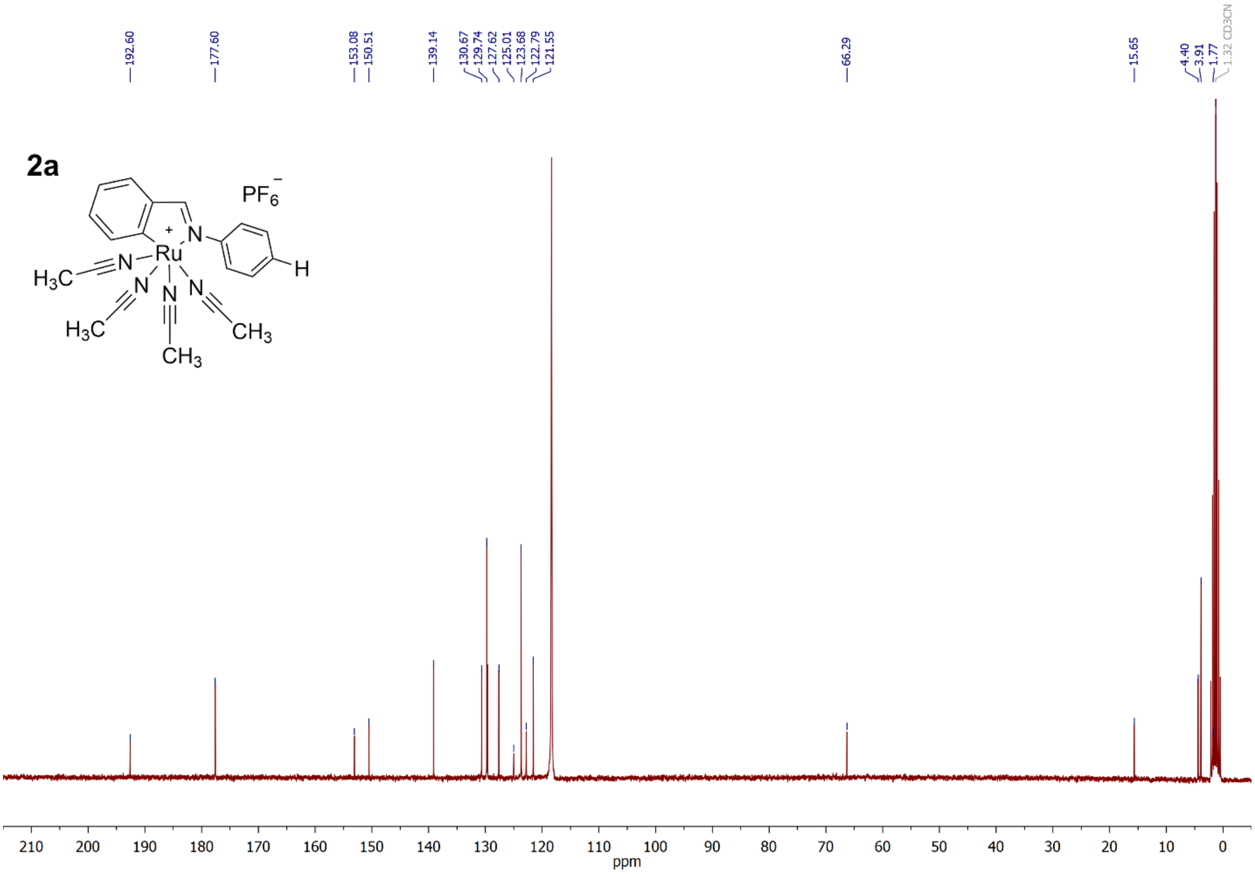

Figure S1. NMR spectra of **2a**.

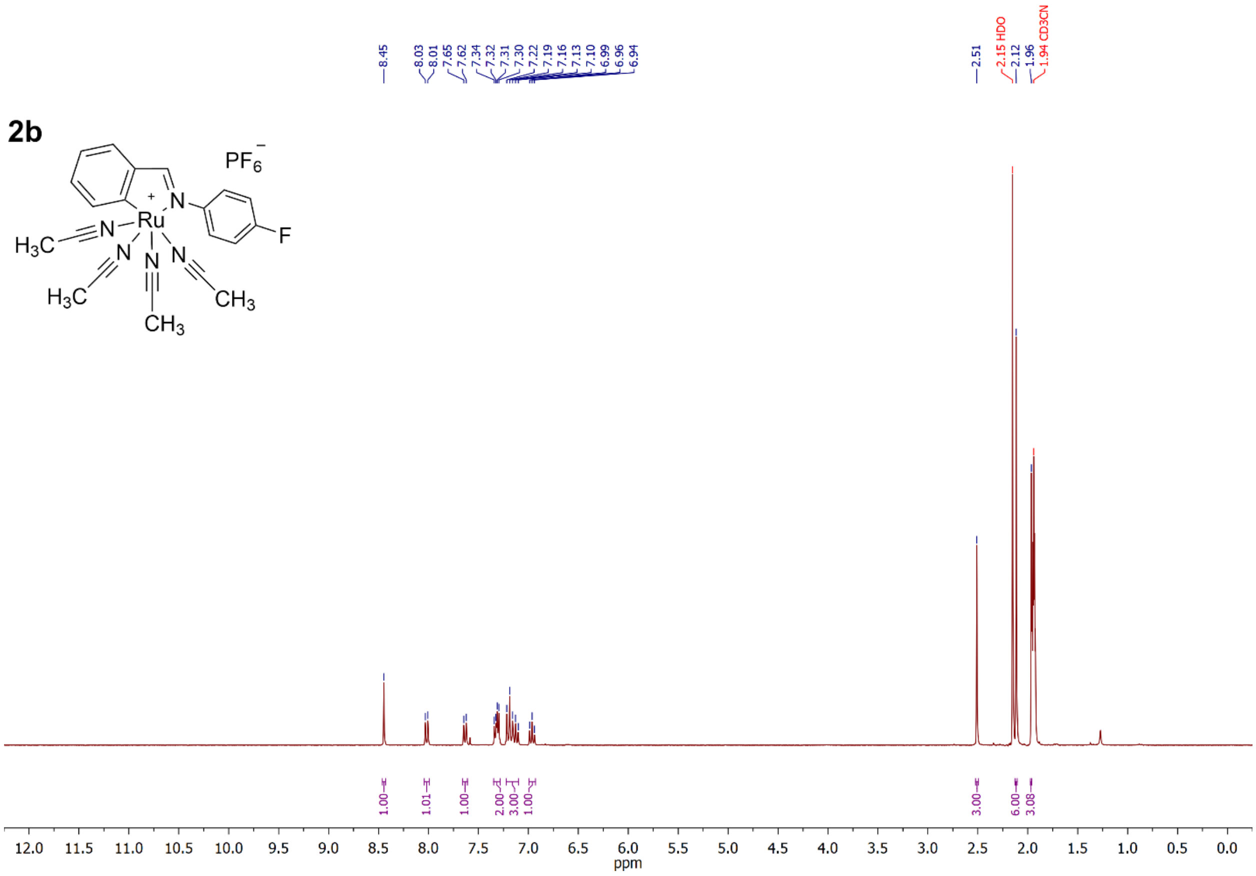

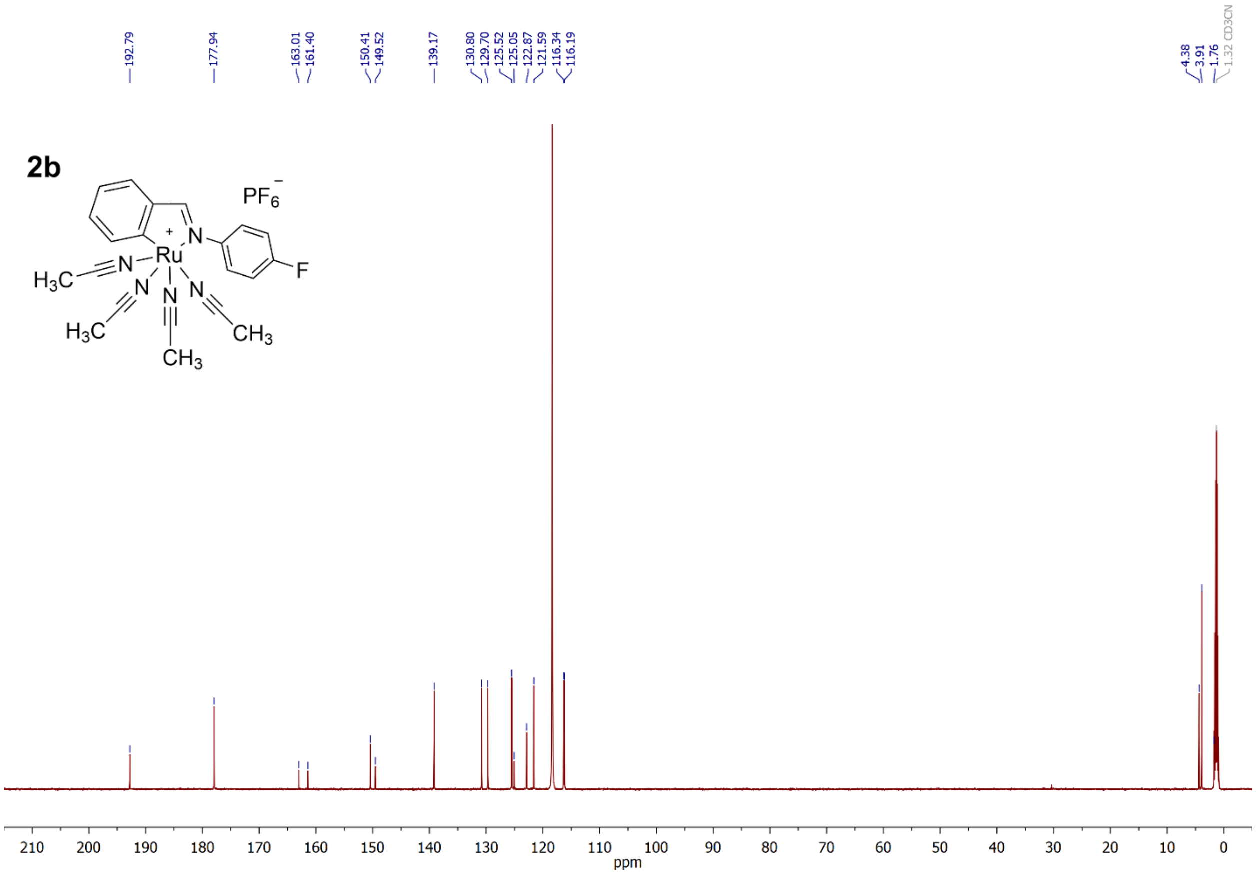

Figure S2. NMR spectra of **2b**.

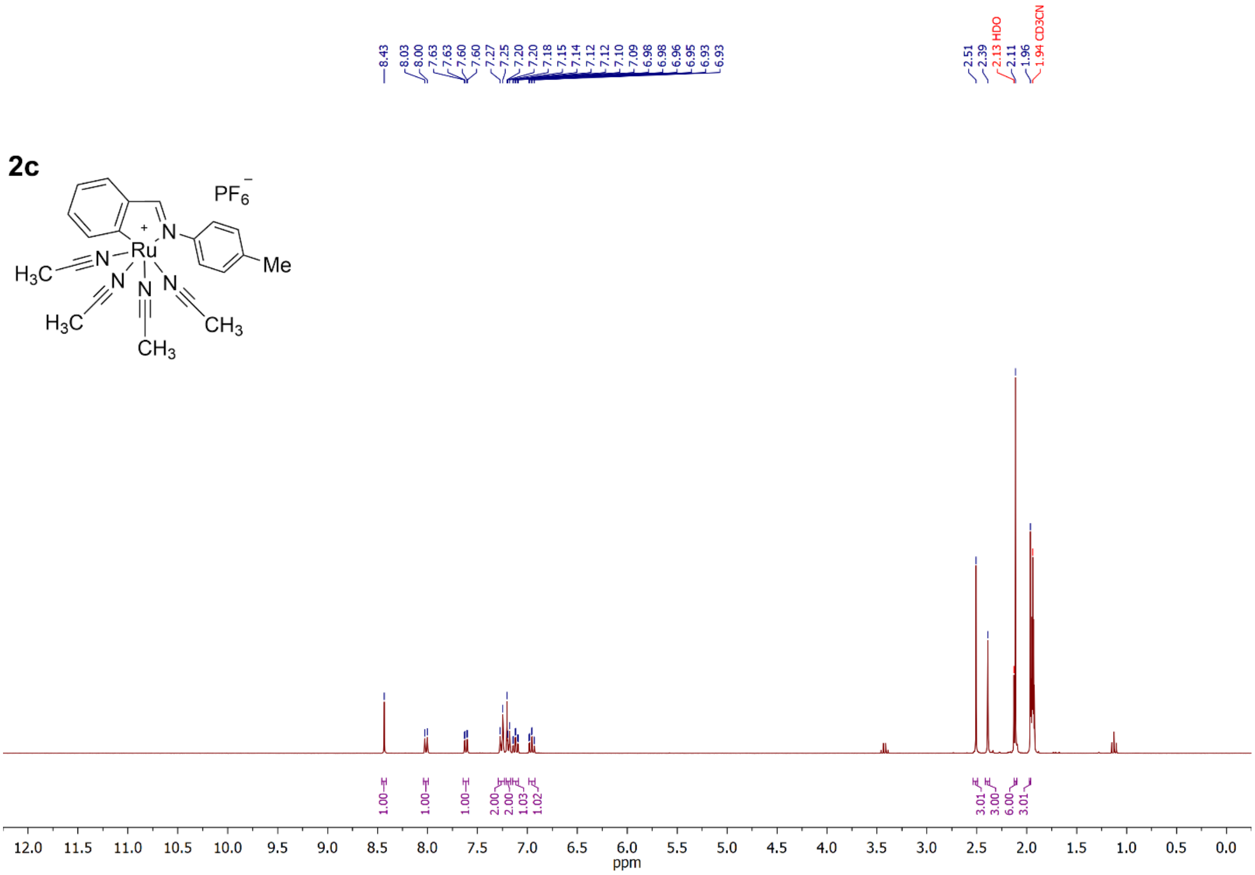

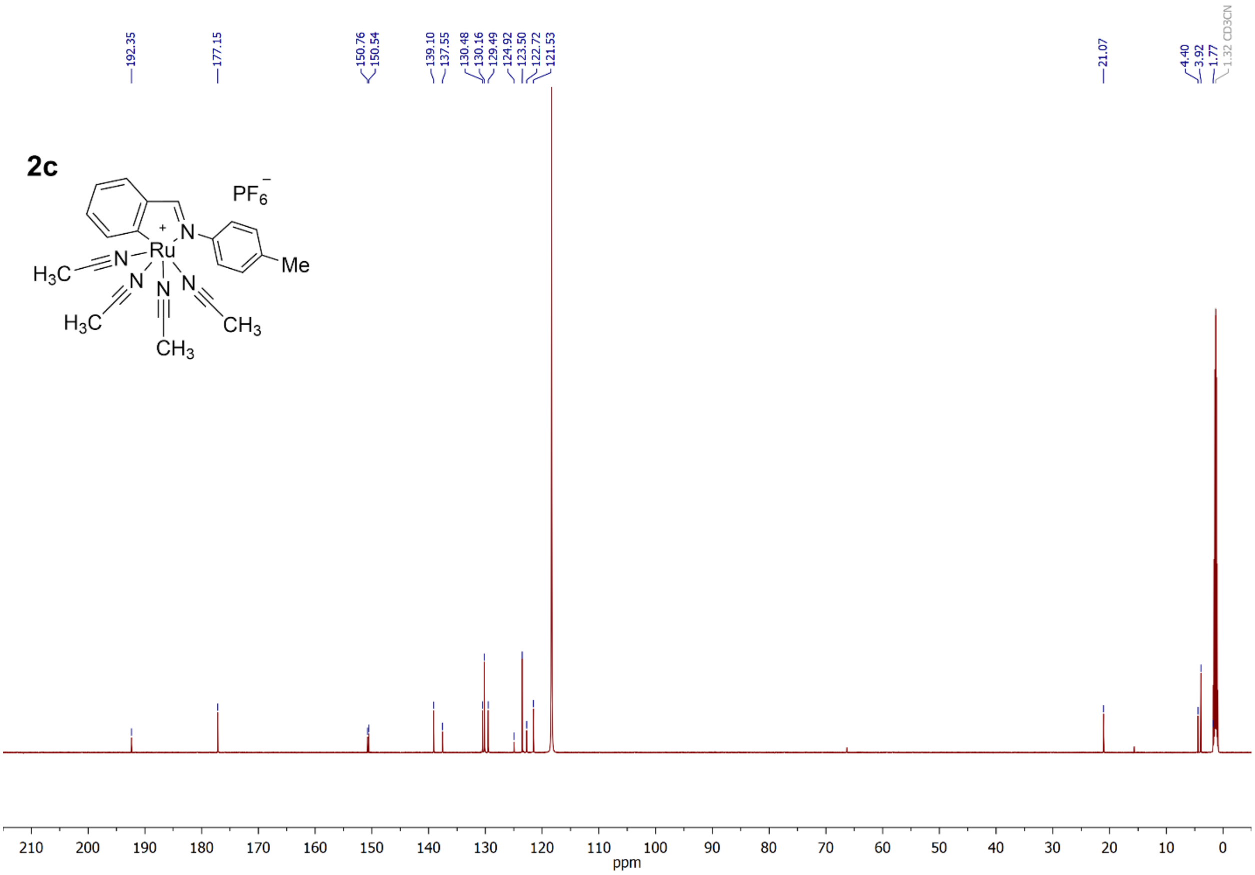

Figure S3. NMR spectra of **2c**.

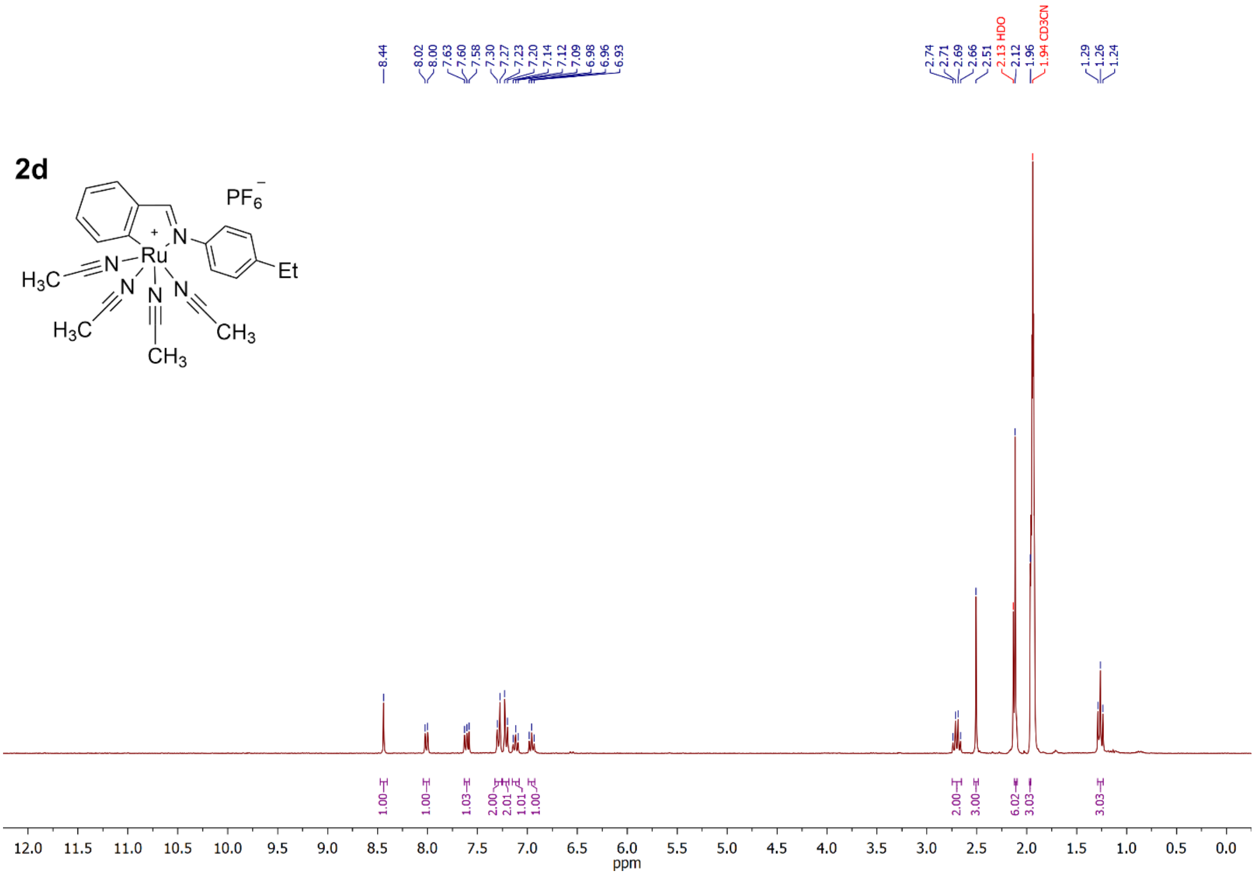

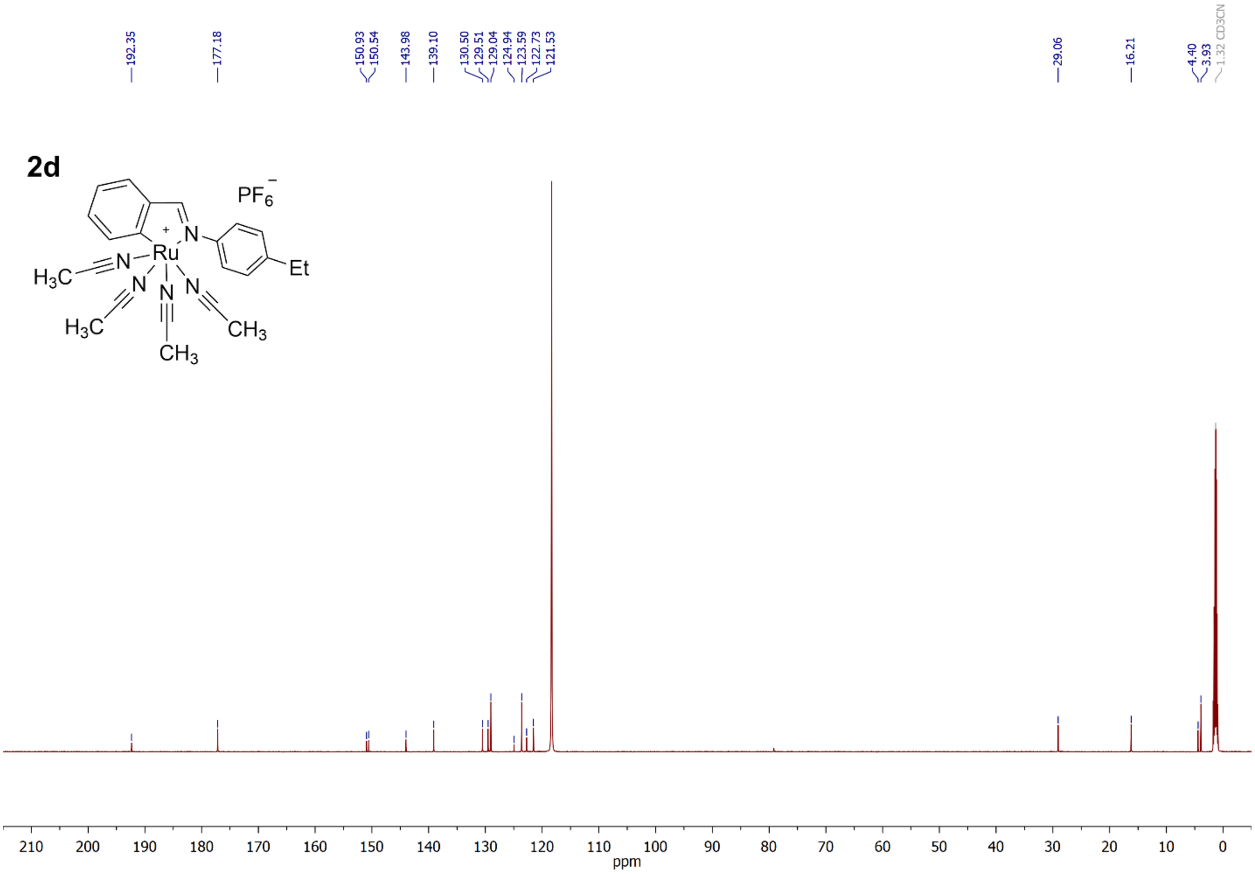

Figure S4. NMR spectra of **2d**.

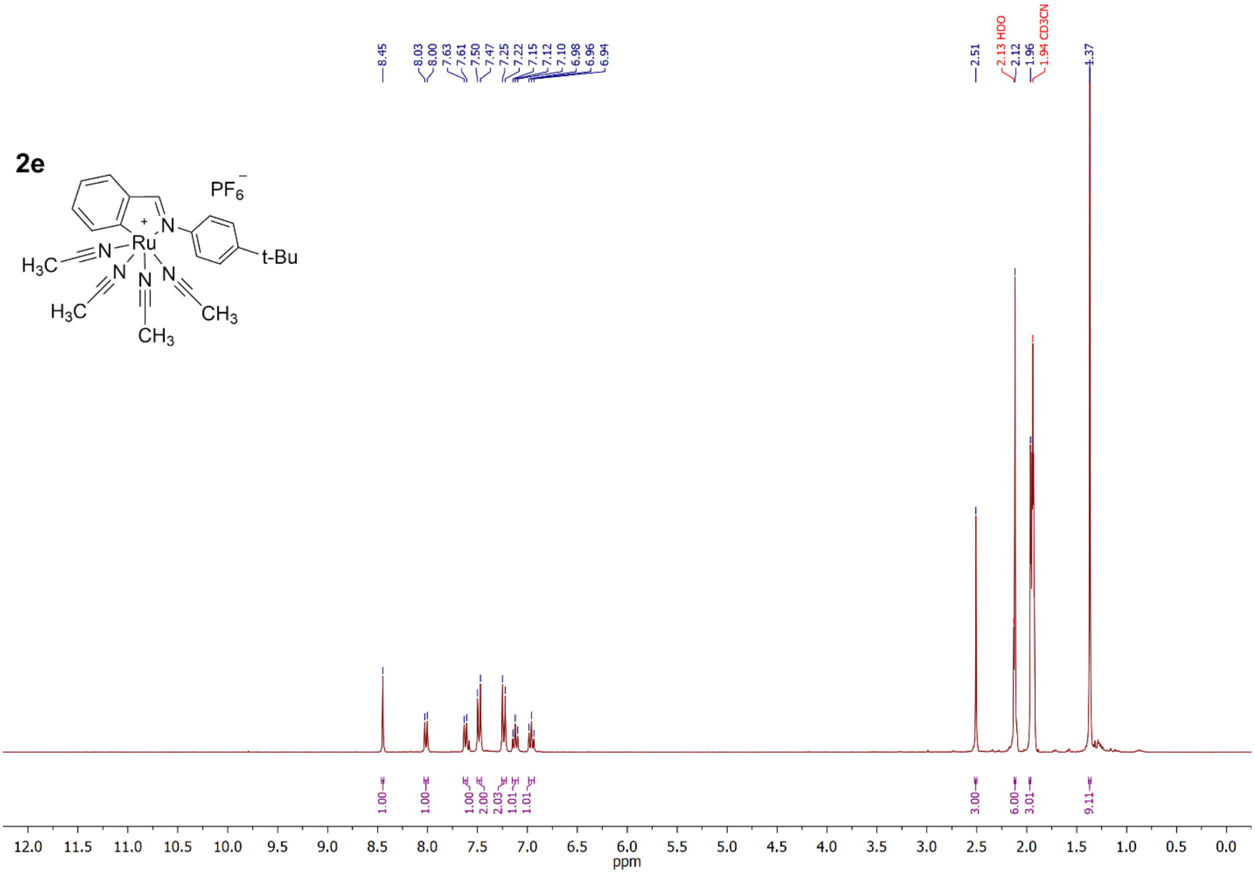

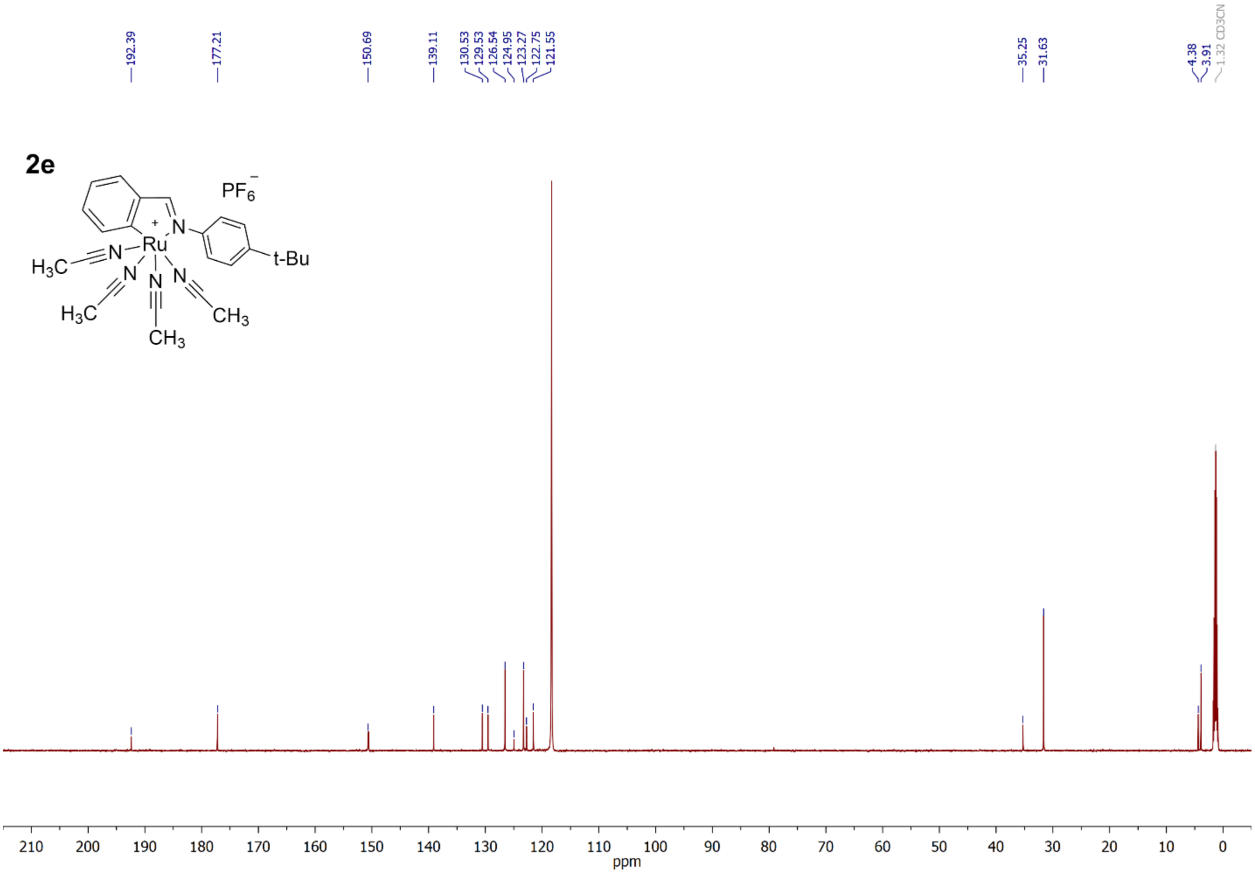

Figure S5. NMR spectra of **2e**.

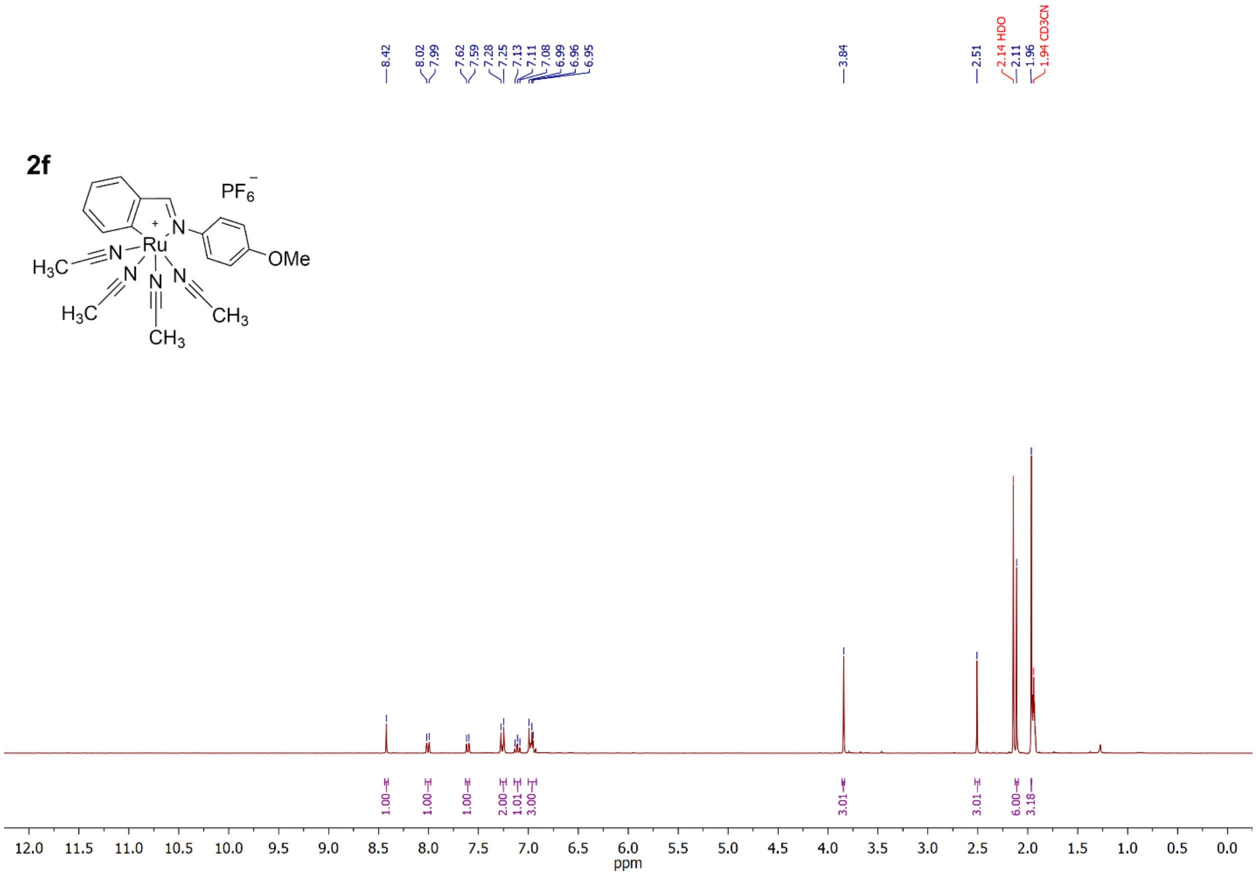

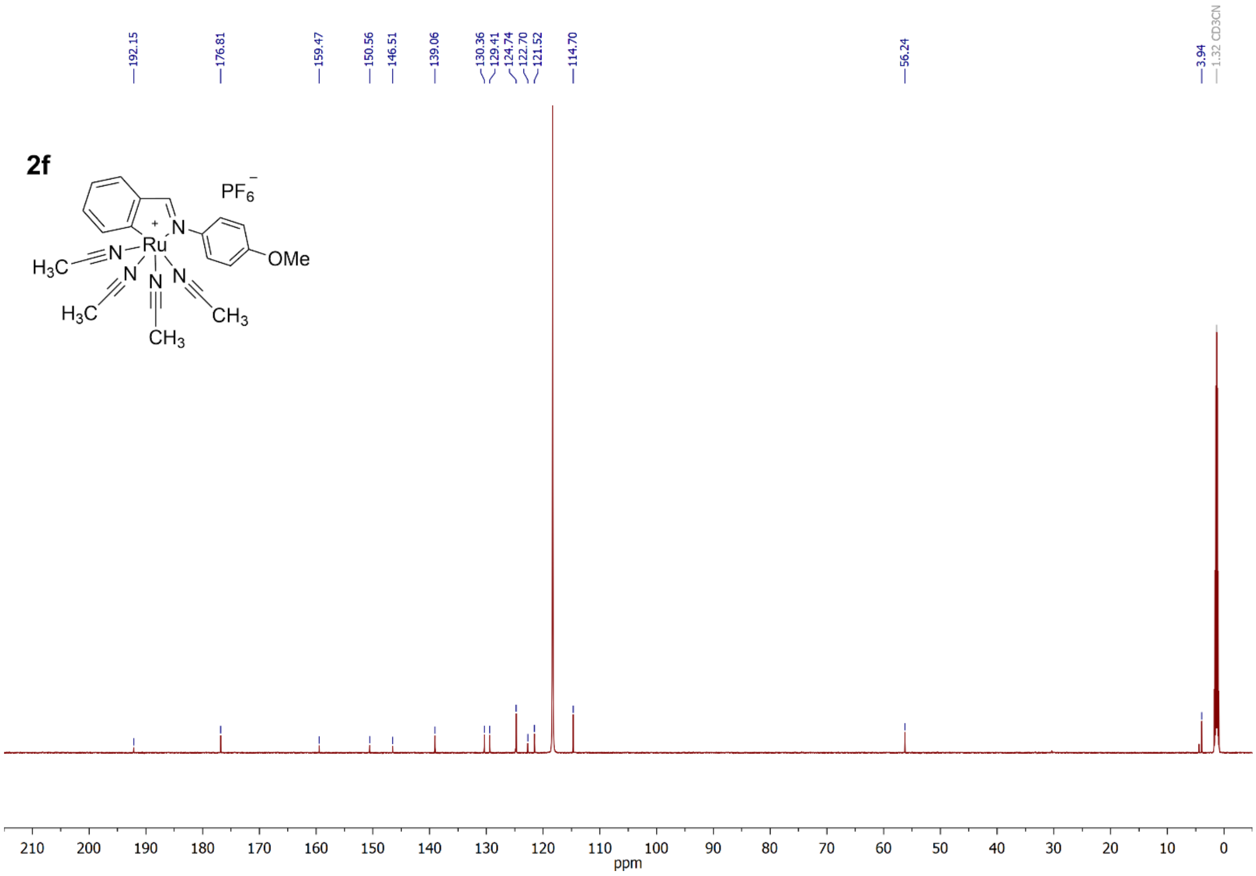

Figure S6. NMR spectra of **2f**.

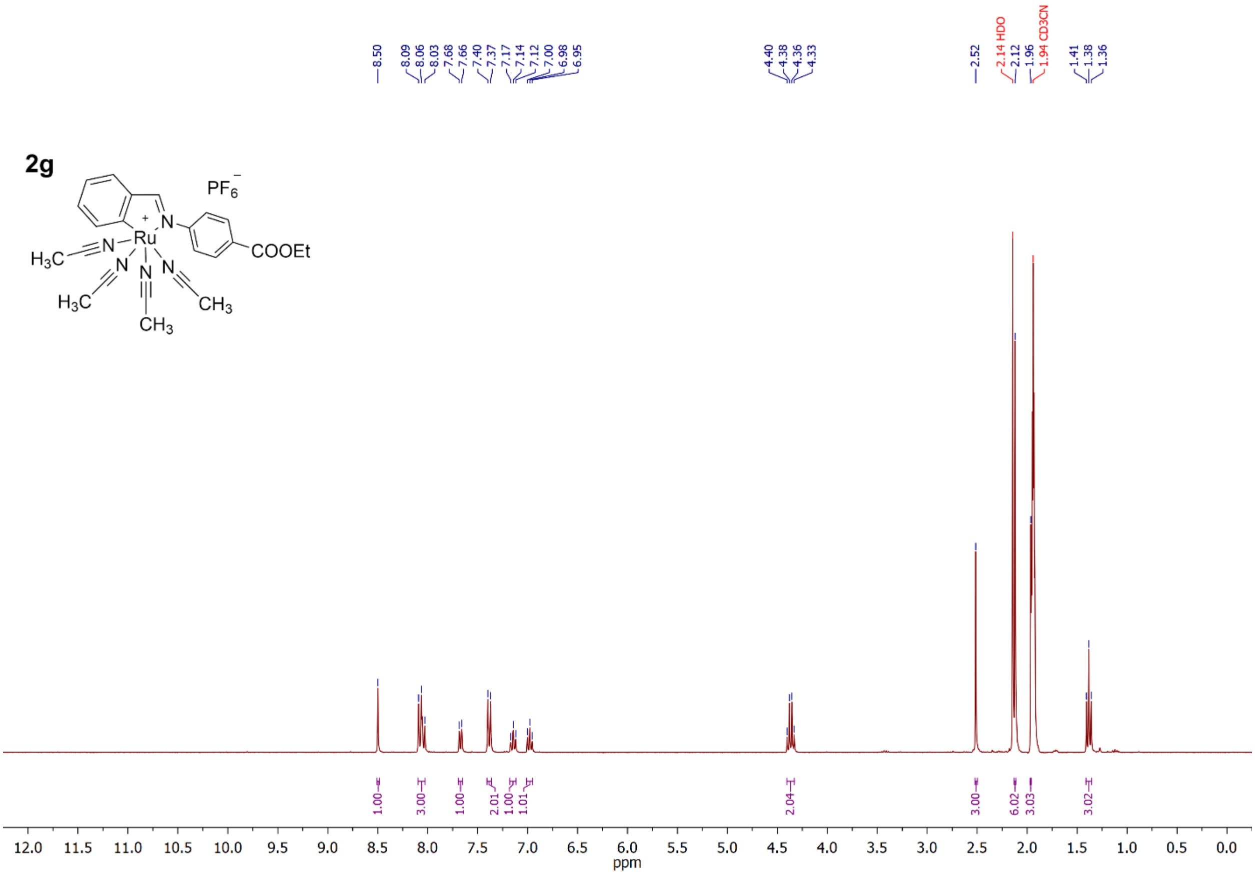

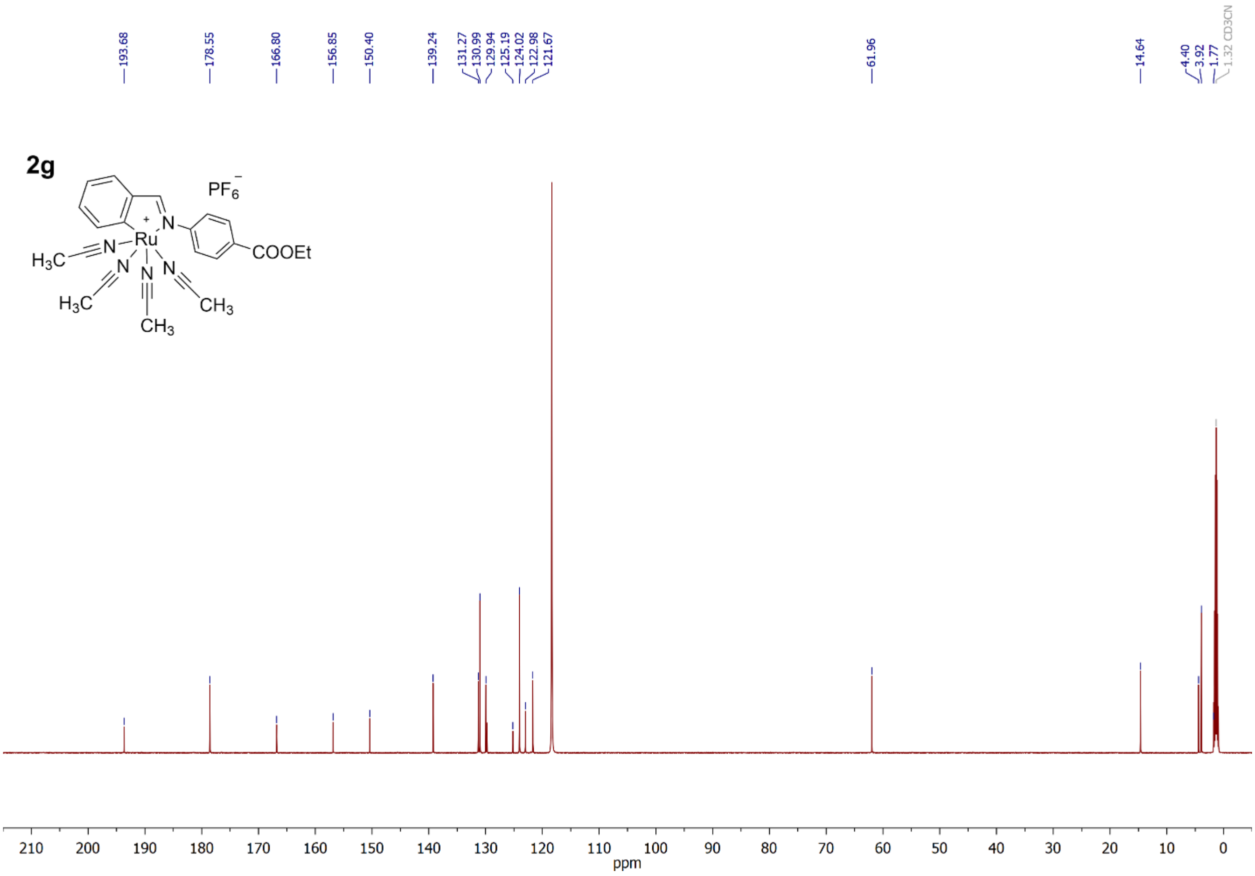

Figure S7. NMR spectra of **2g**.

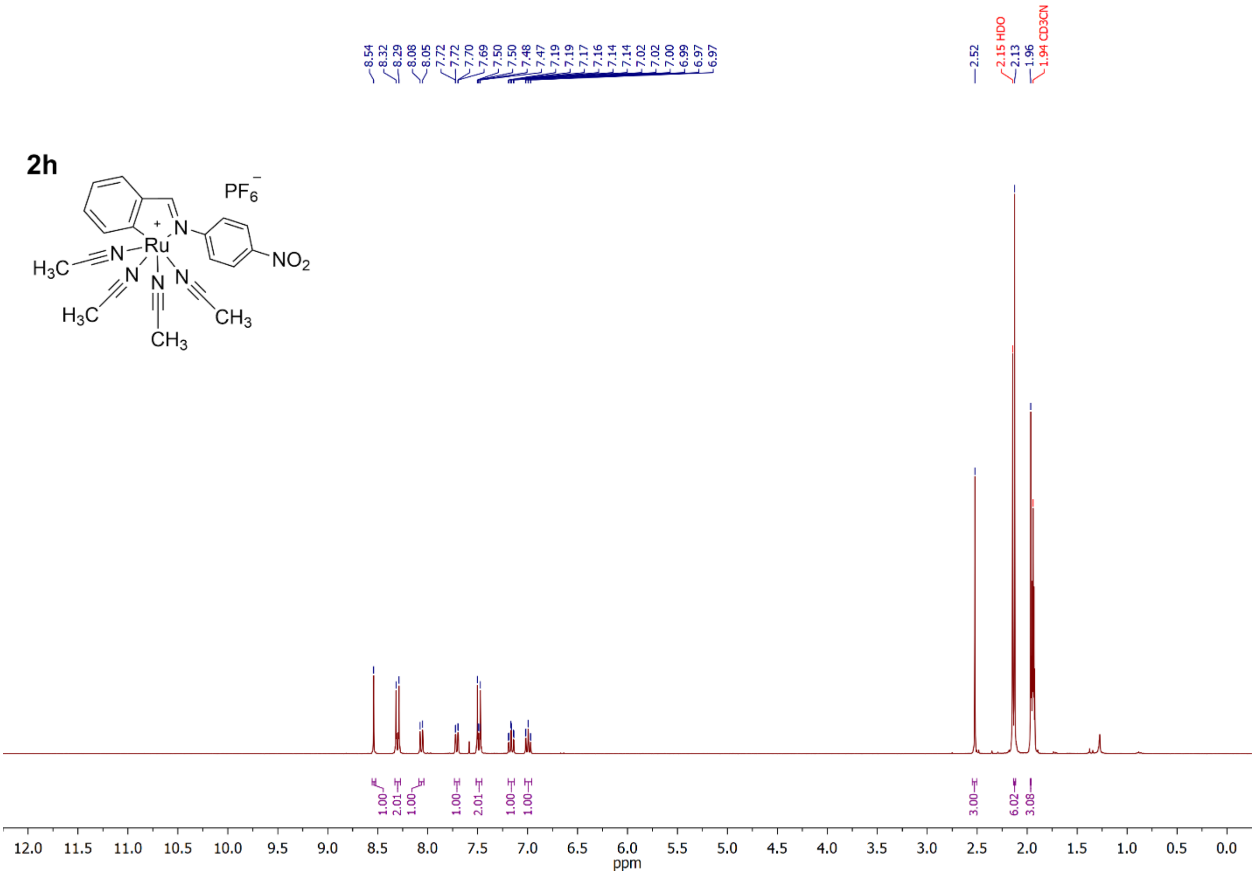

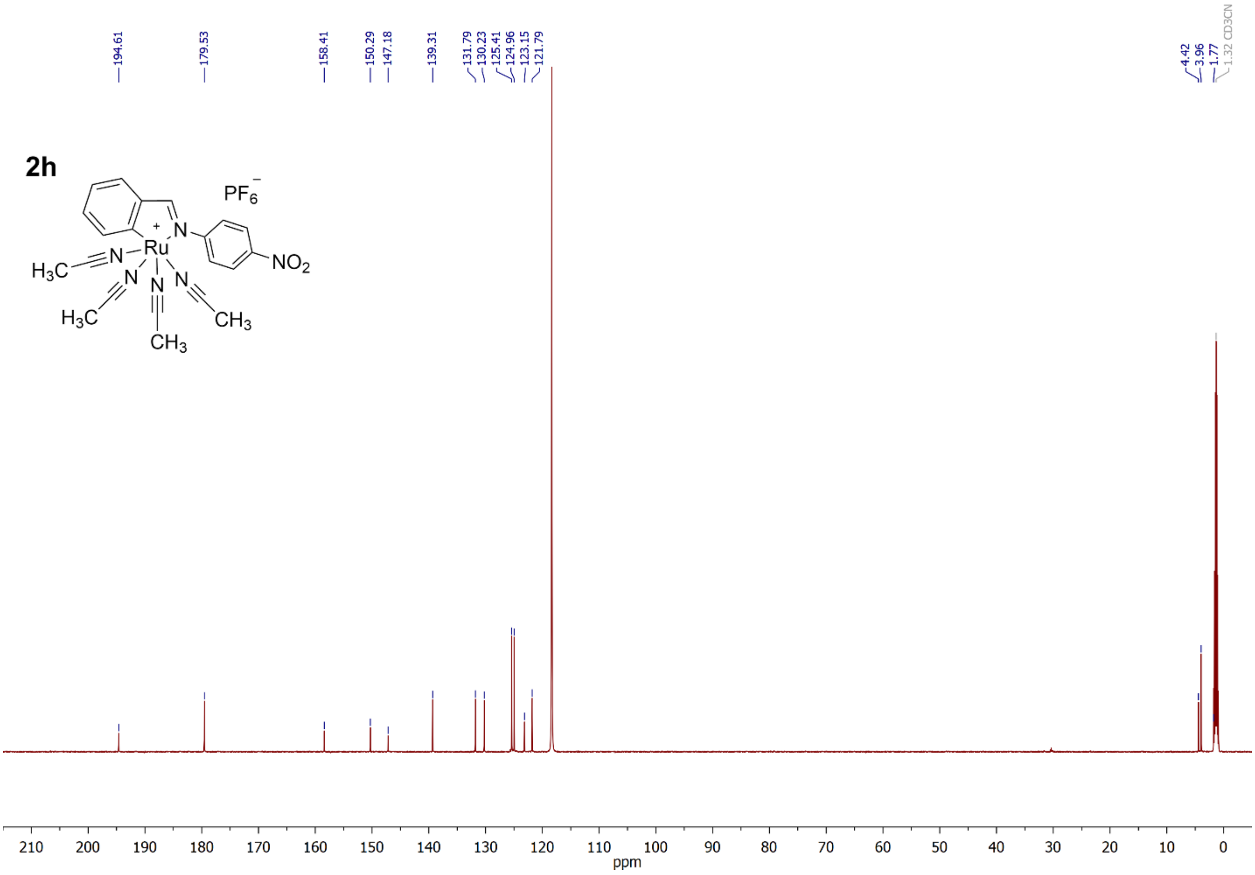

Figure S8. NMR spectra of **2h**.

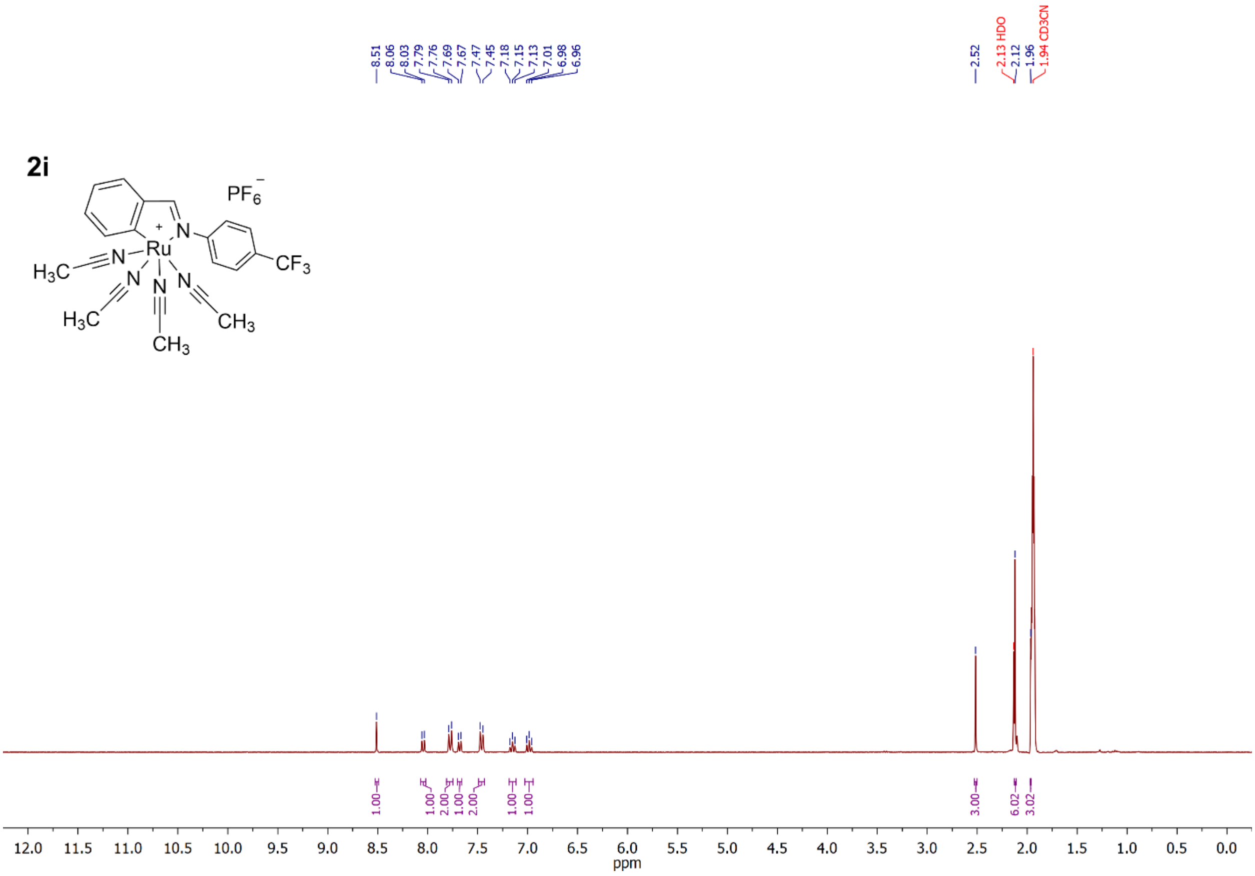

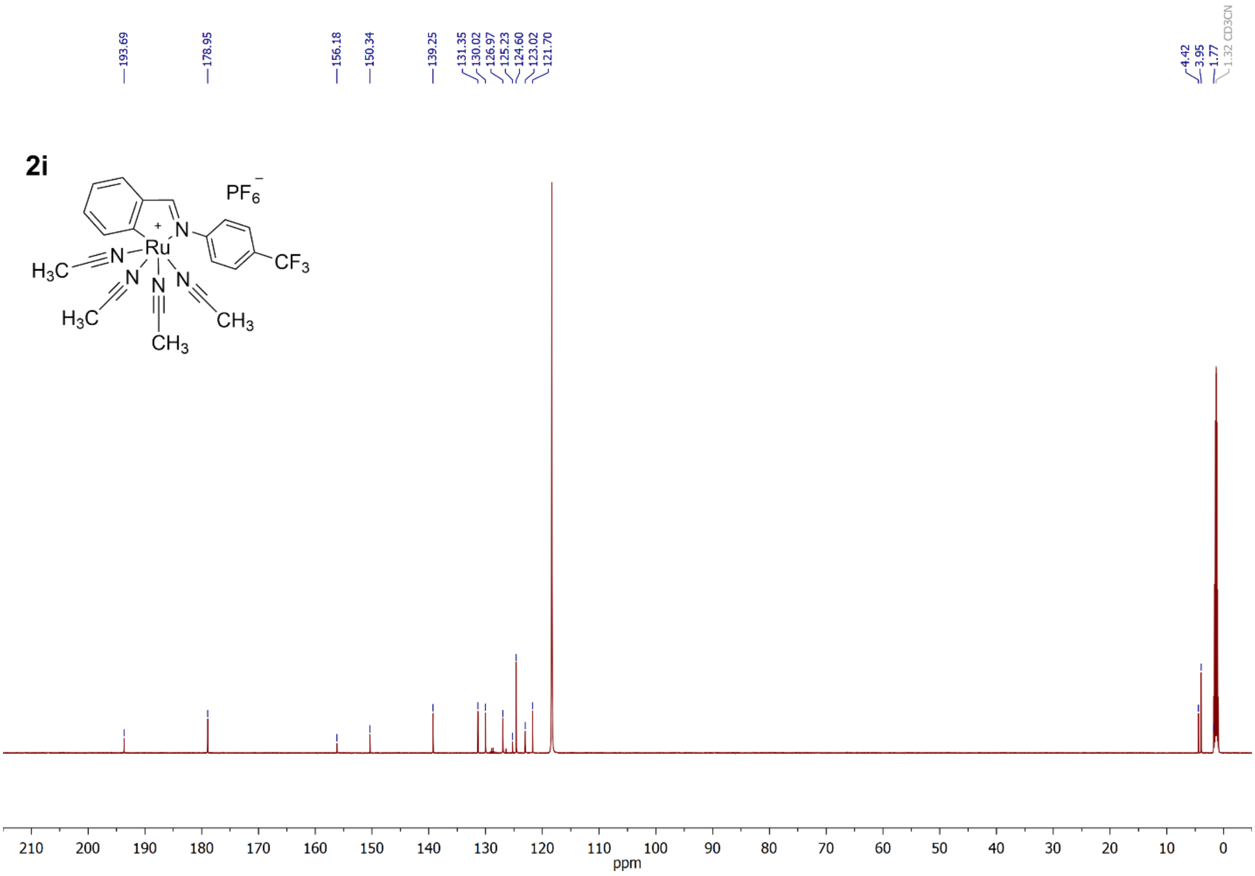

Figure S9. NMR spectra of **2i**.

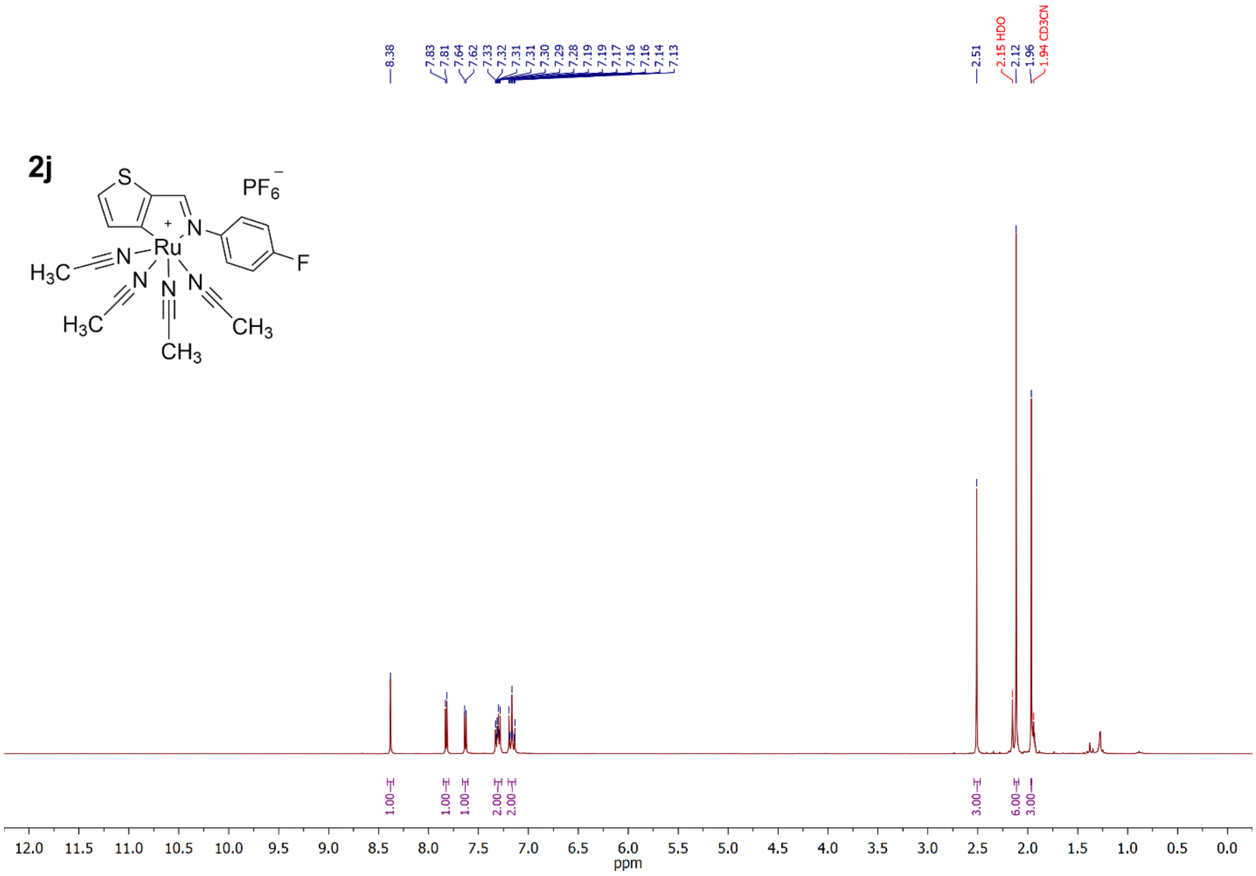

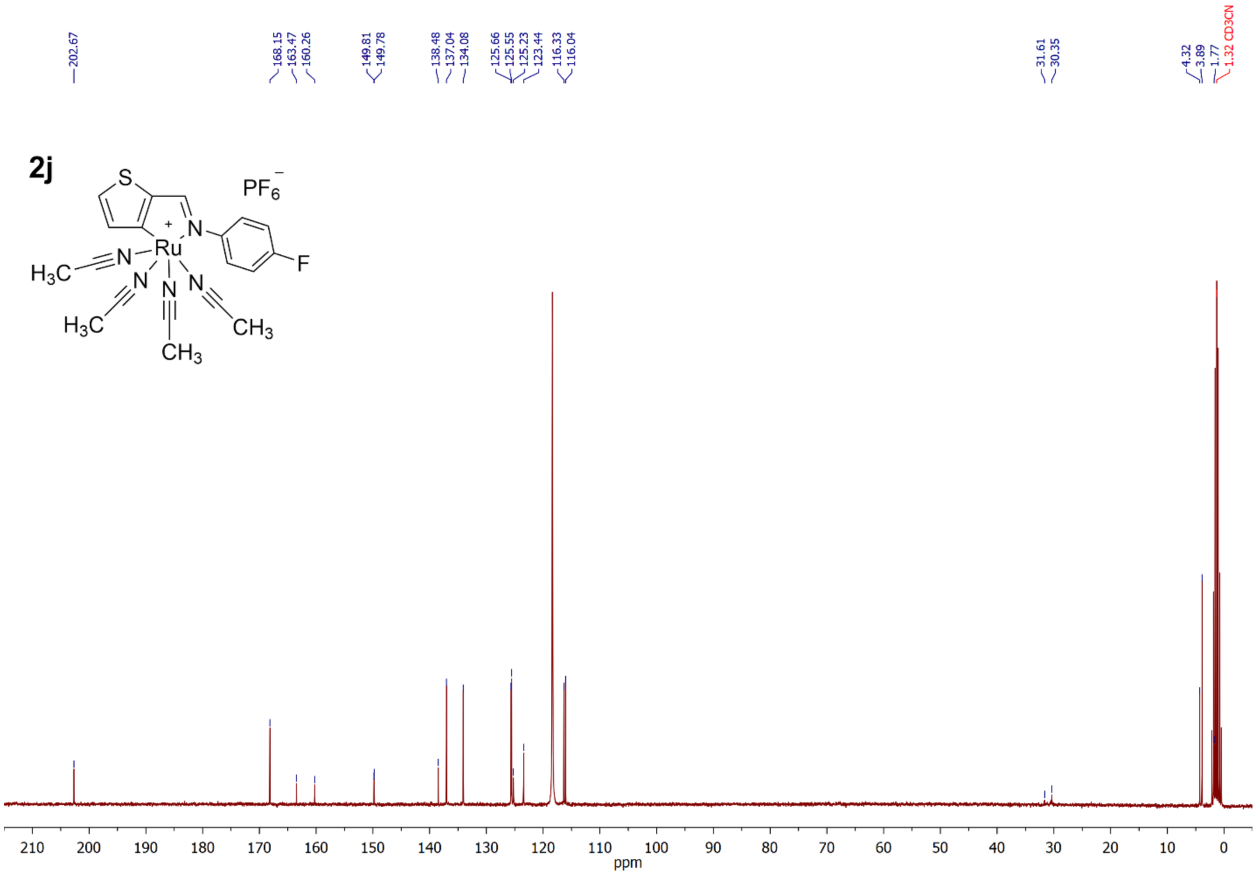

Figure S10. NMR spectra of **2j**.

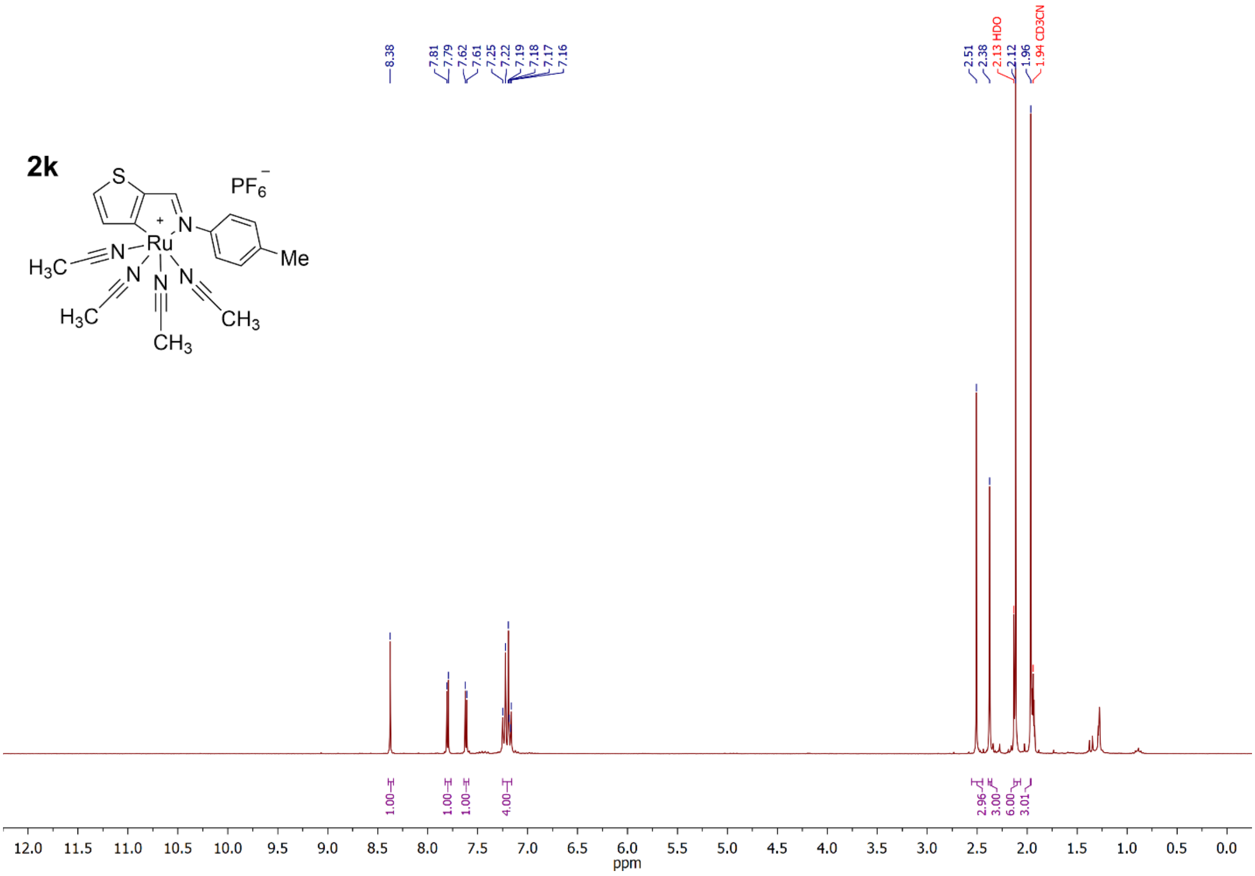

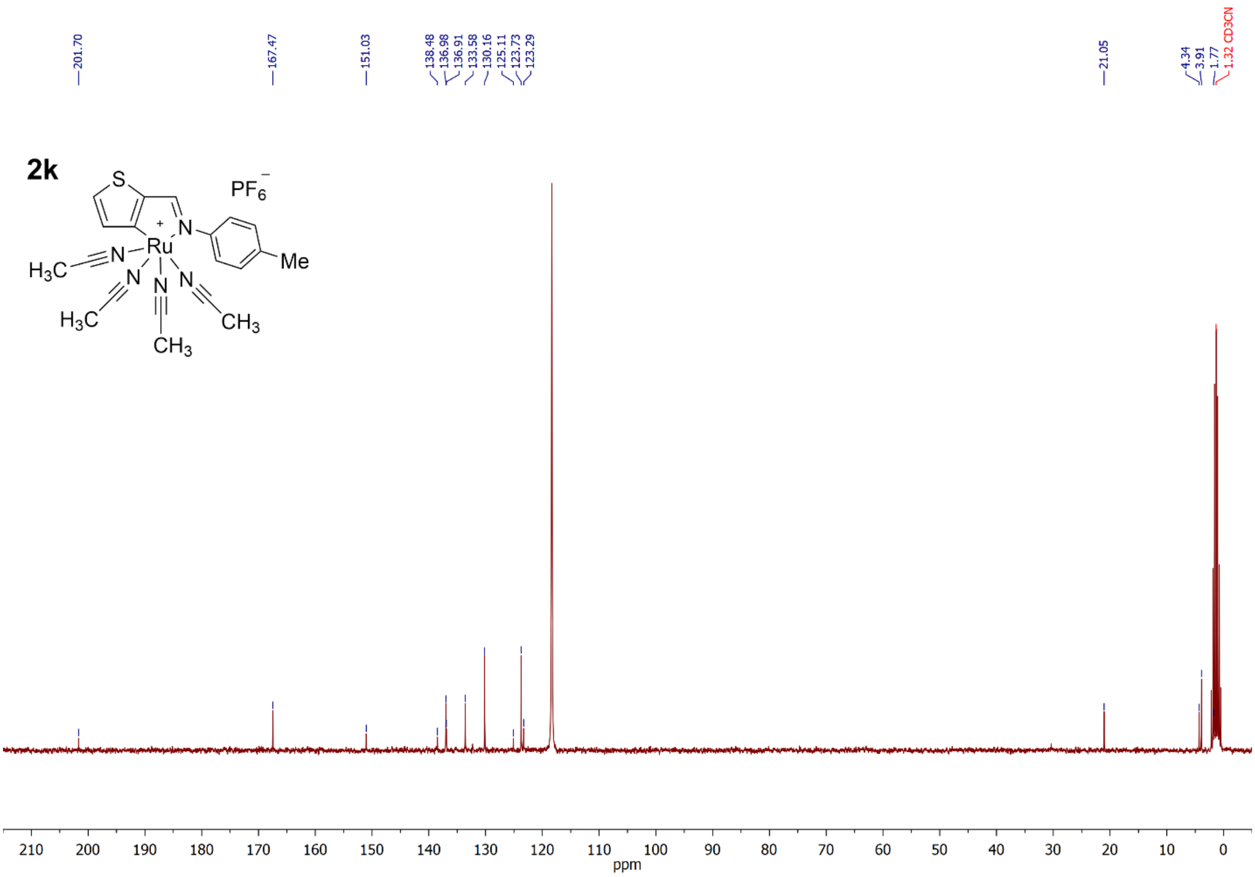

Figure S11. NMR spectra of **2k**.

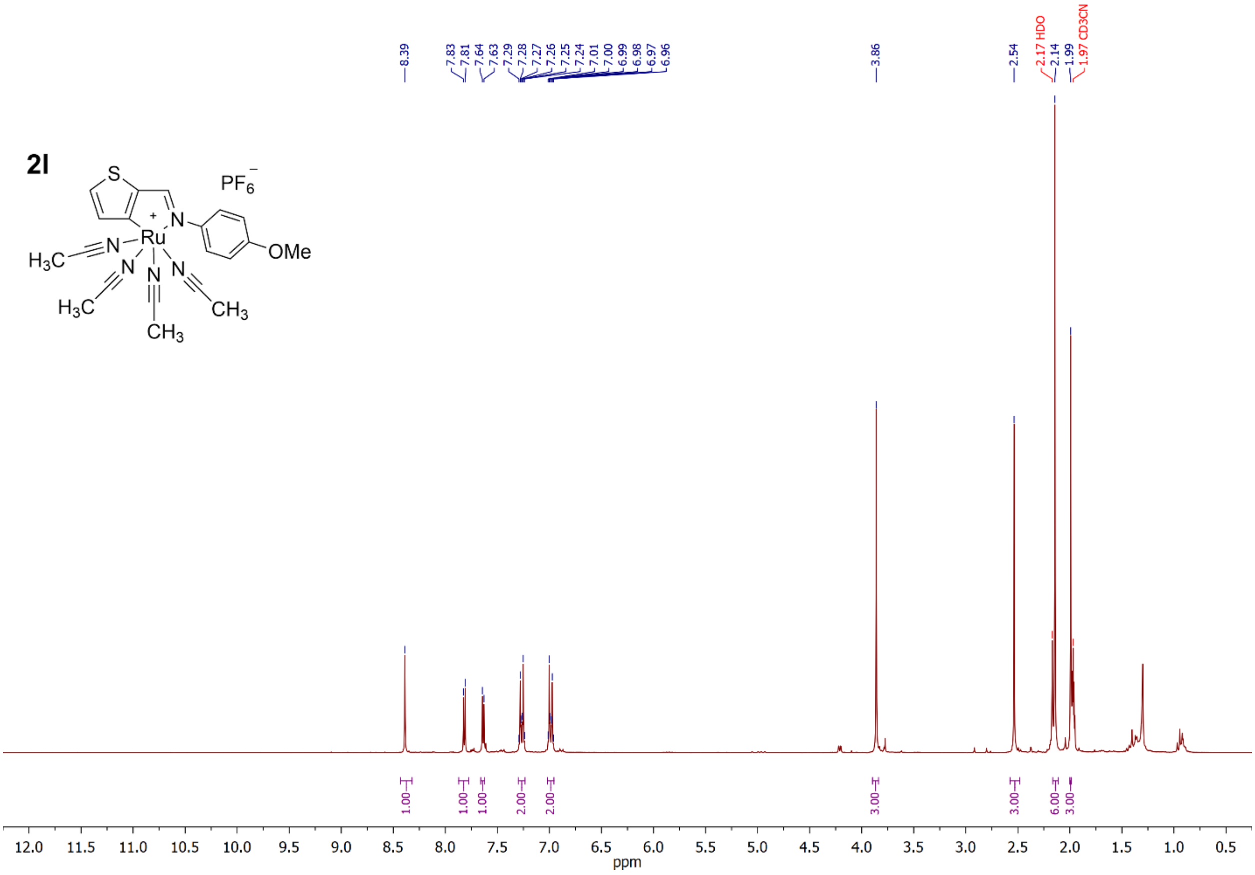

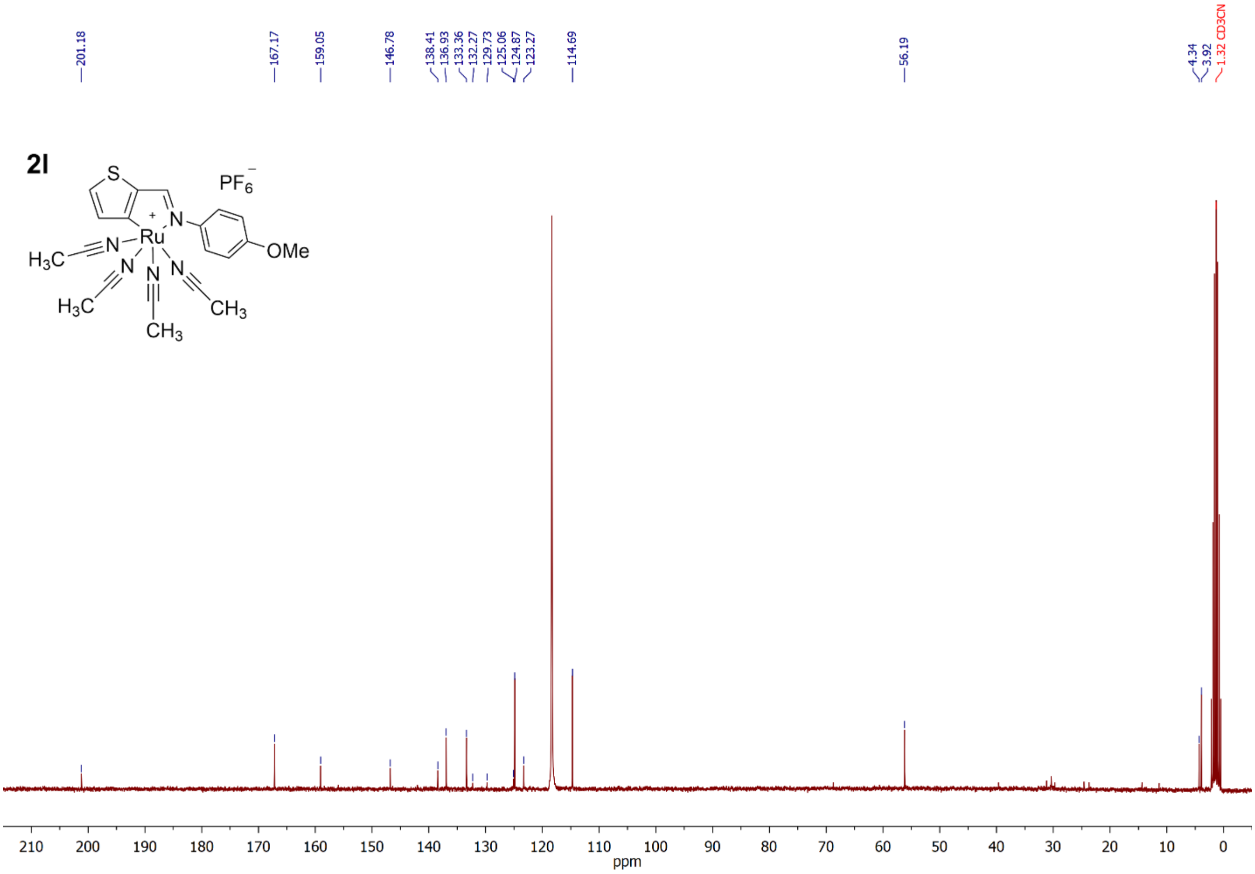

Figure S12. NMR spectra of **2l**.

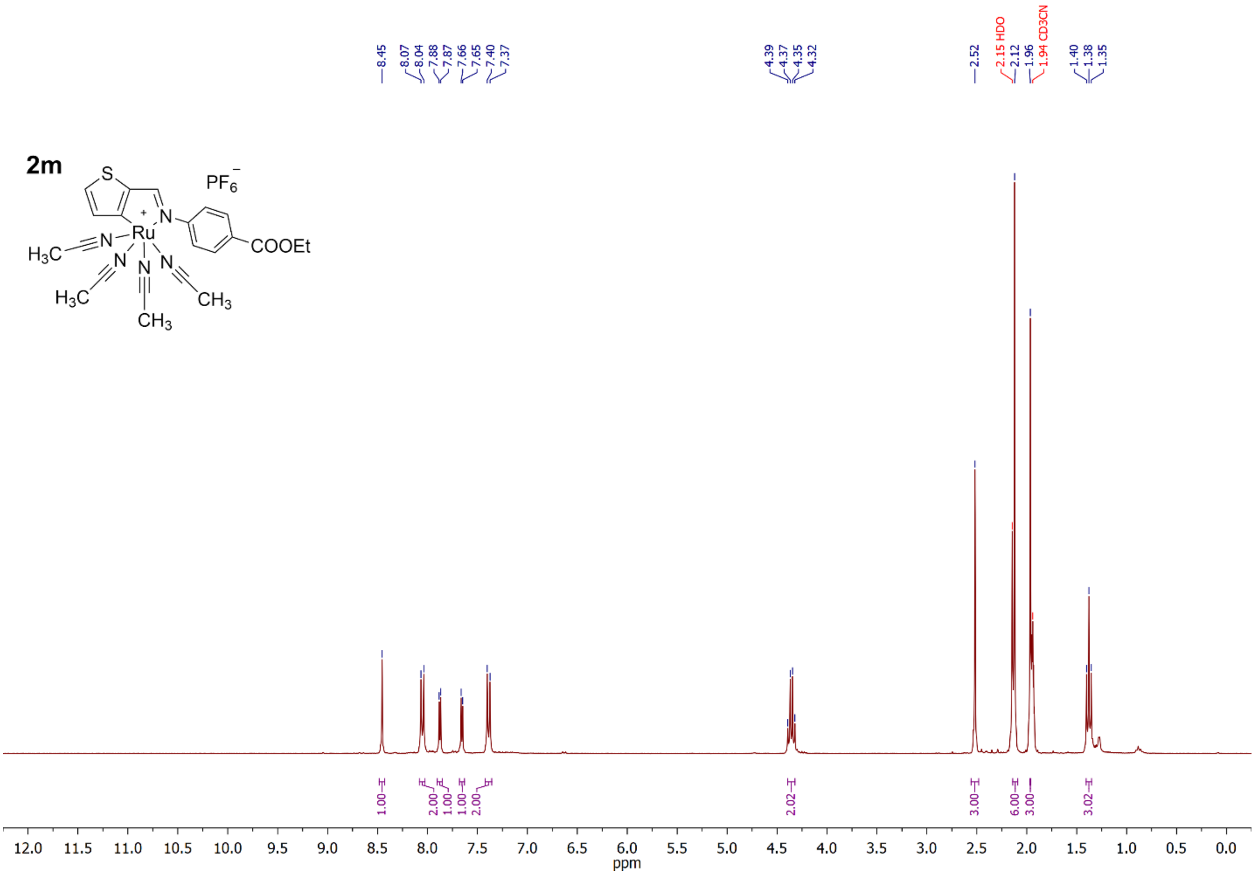

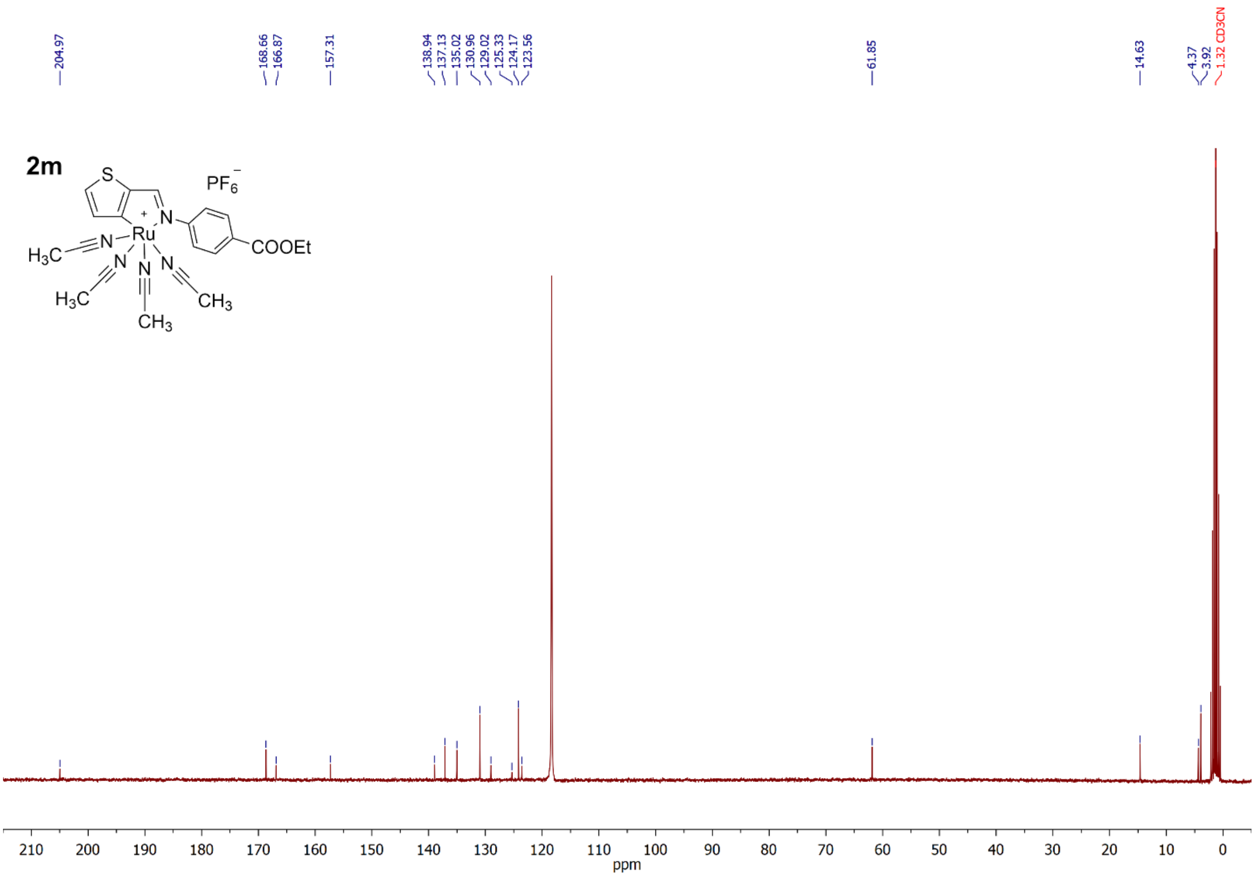

Figure S13. NMR spectra of **2m**.

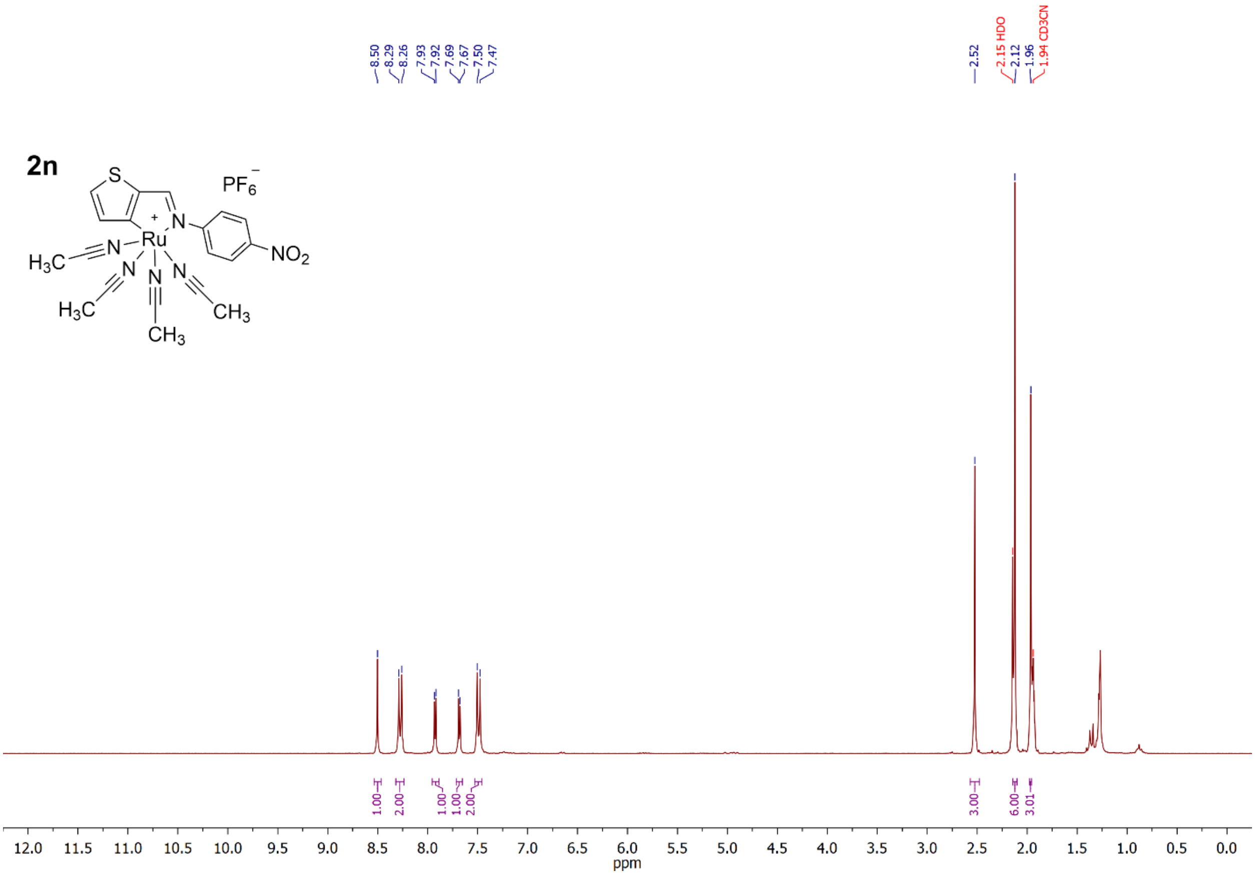

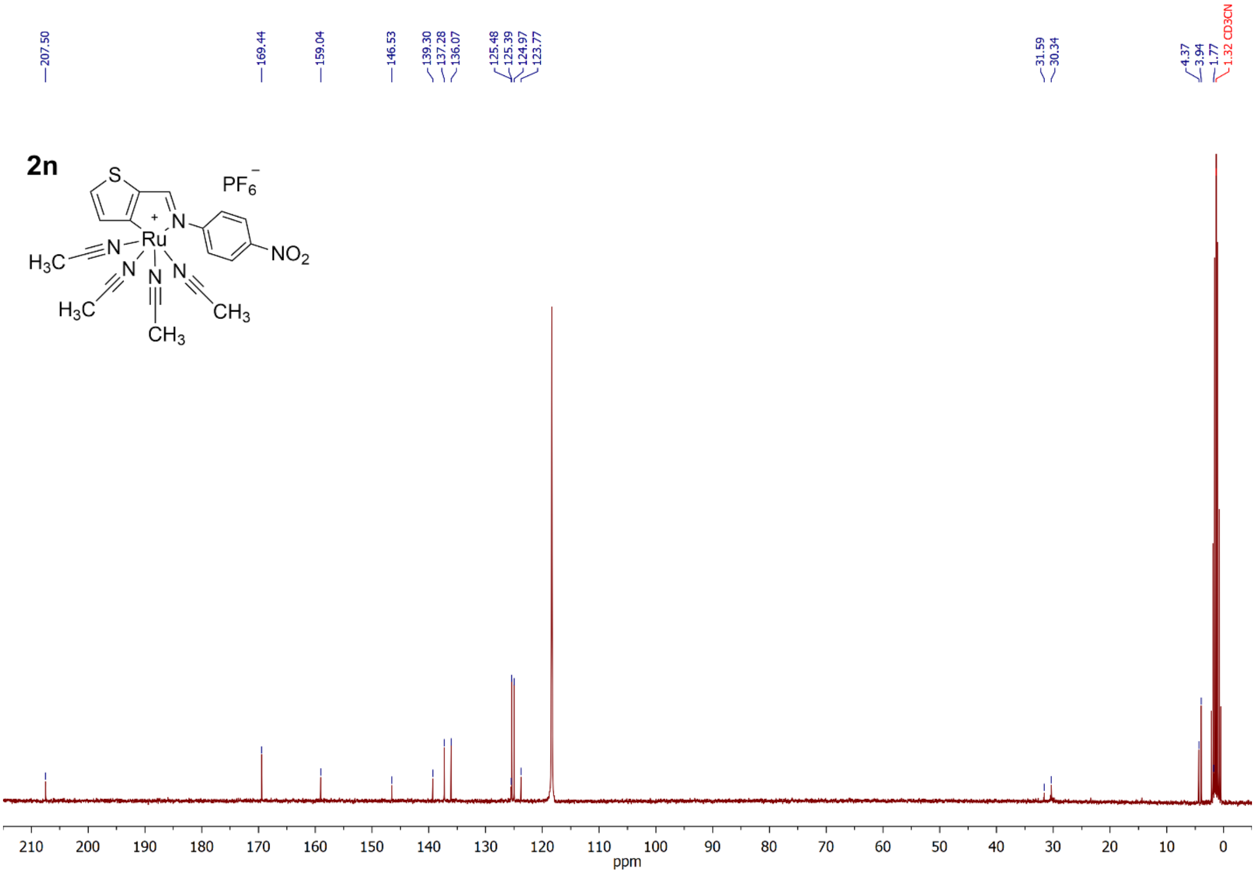

Figure S14. NMR spectra of **2n**.

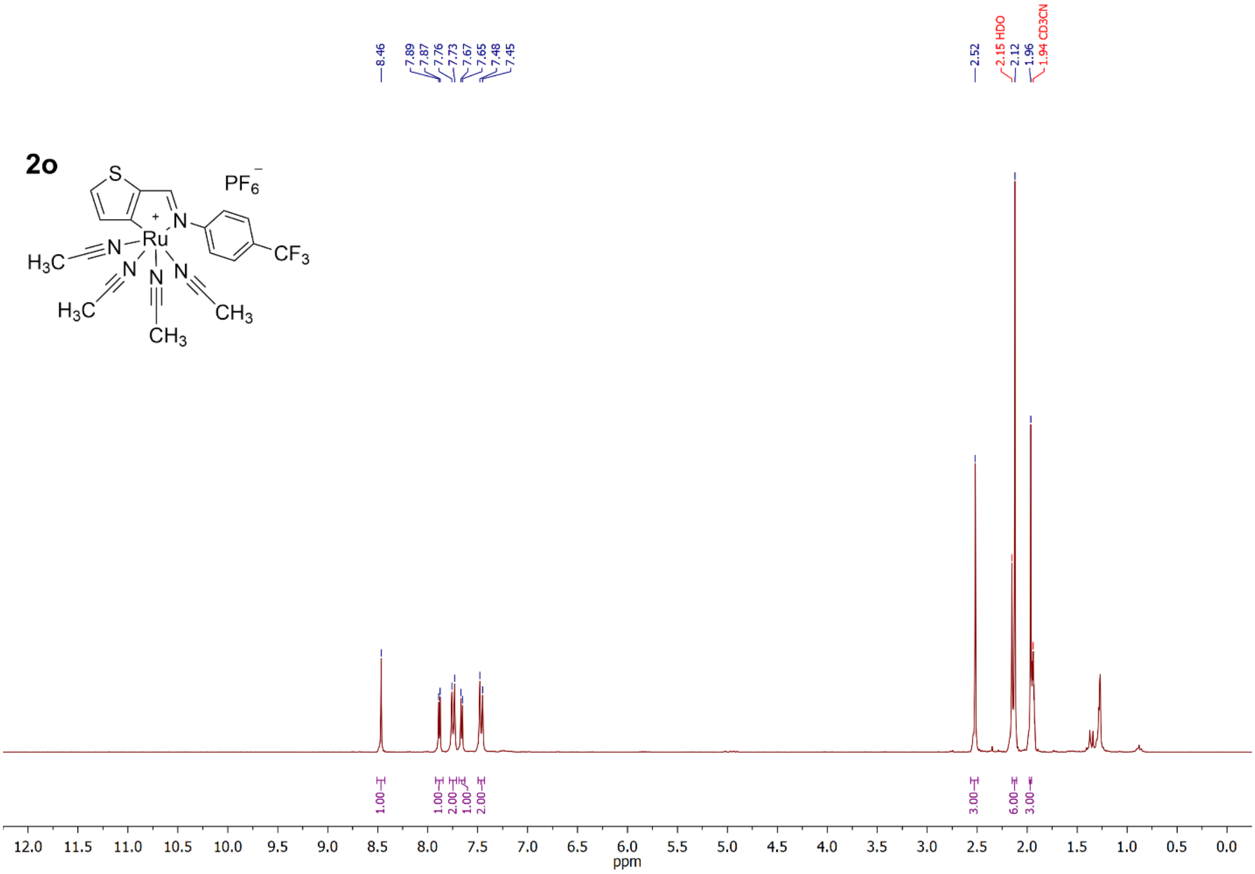

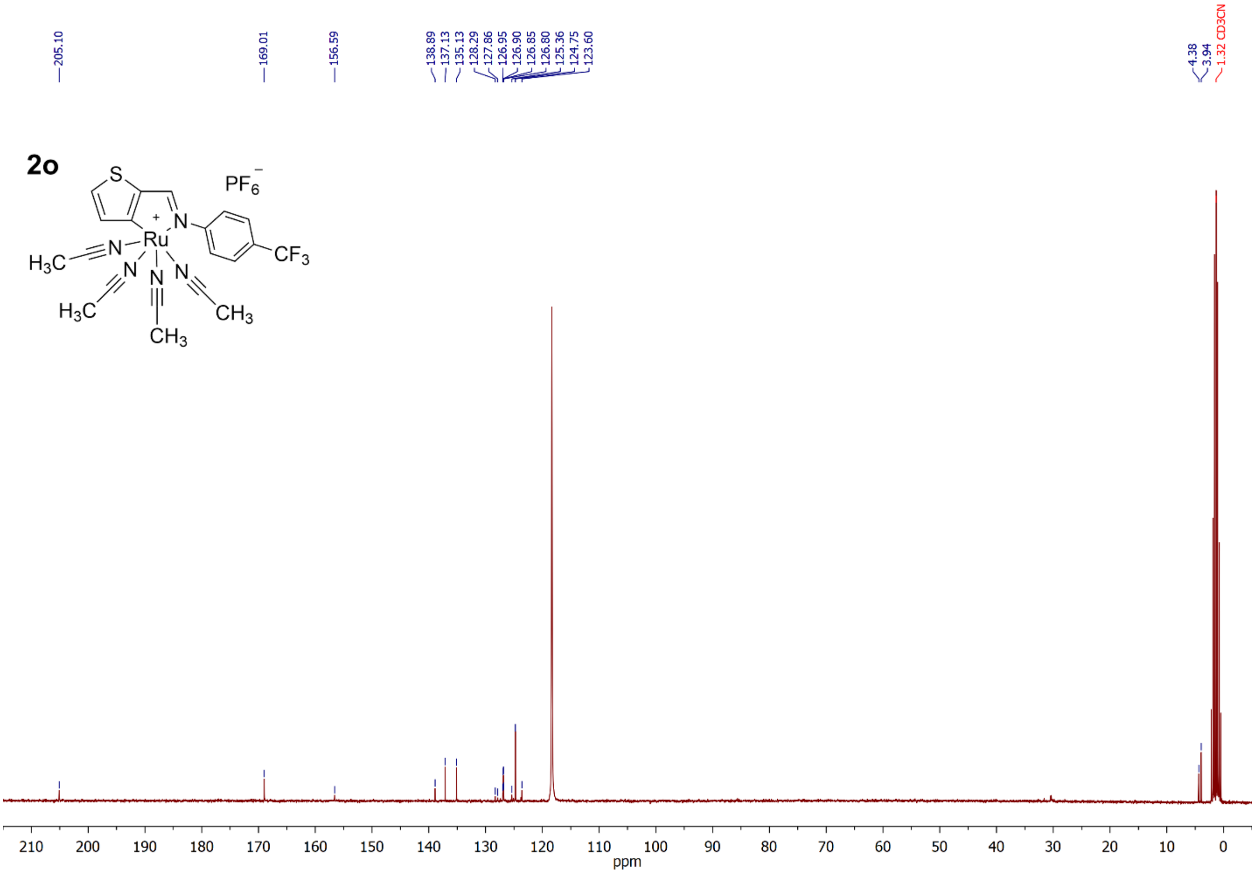

Figure S15. NMR spectra of **2o**.

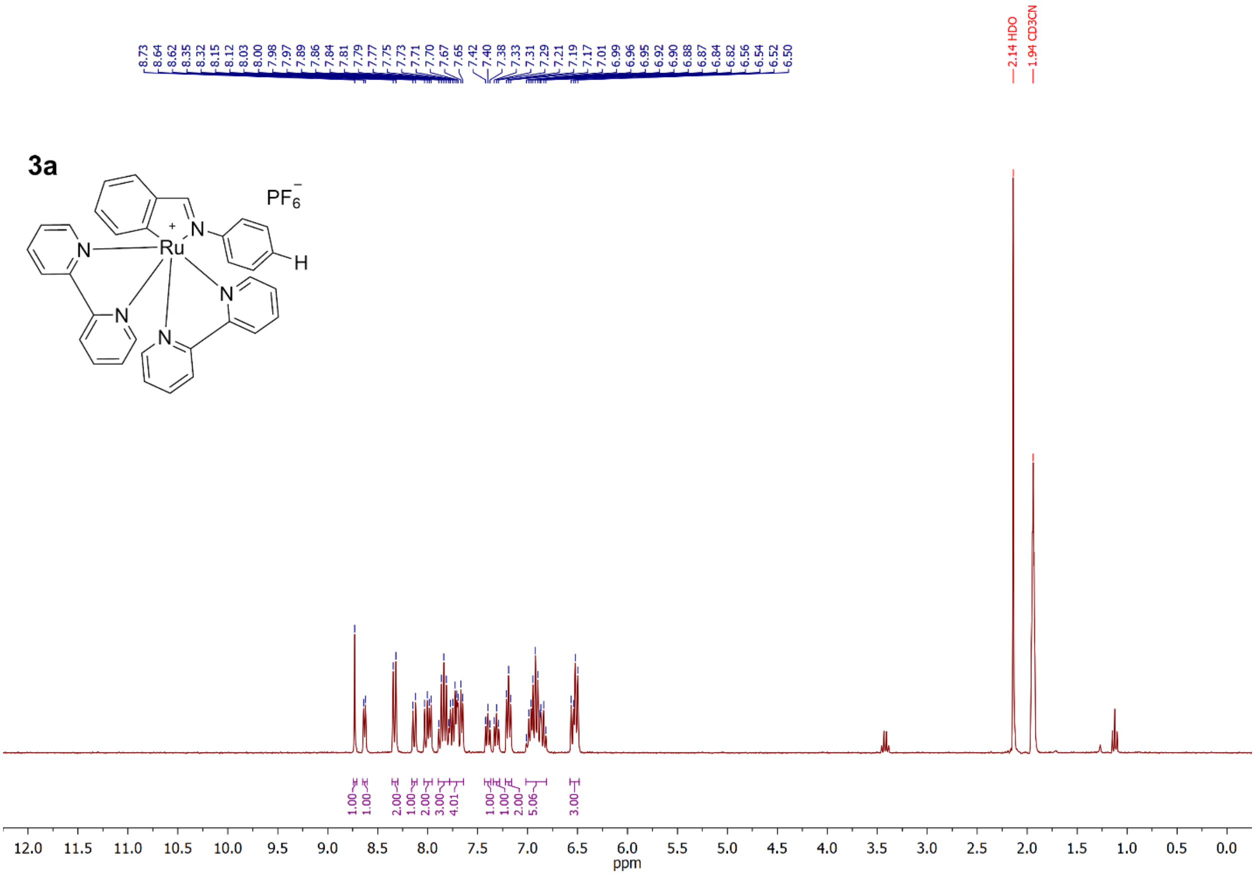

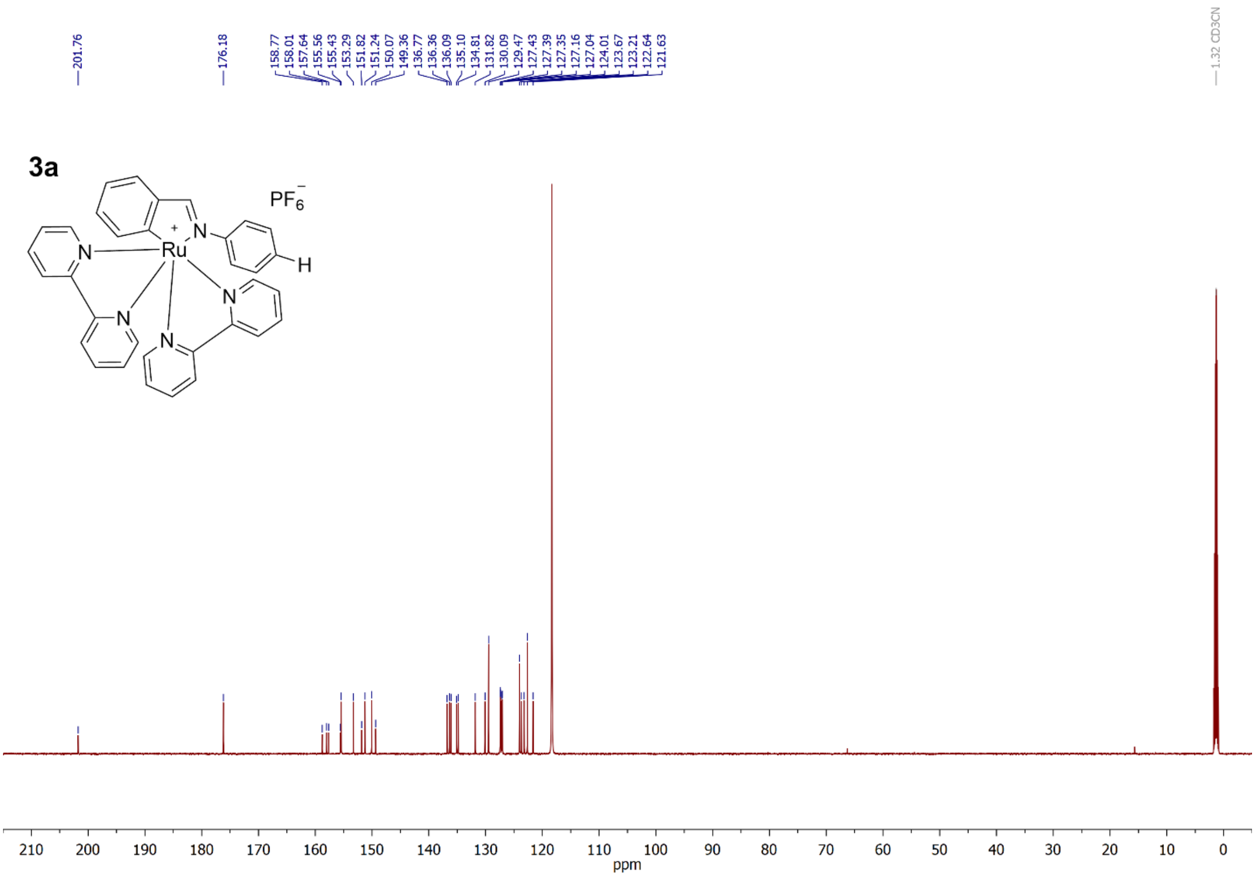

Figure S16. NMR spectra of **3a**.

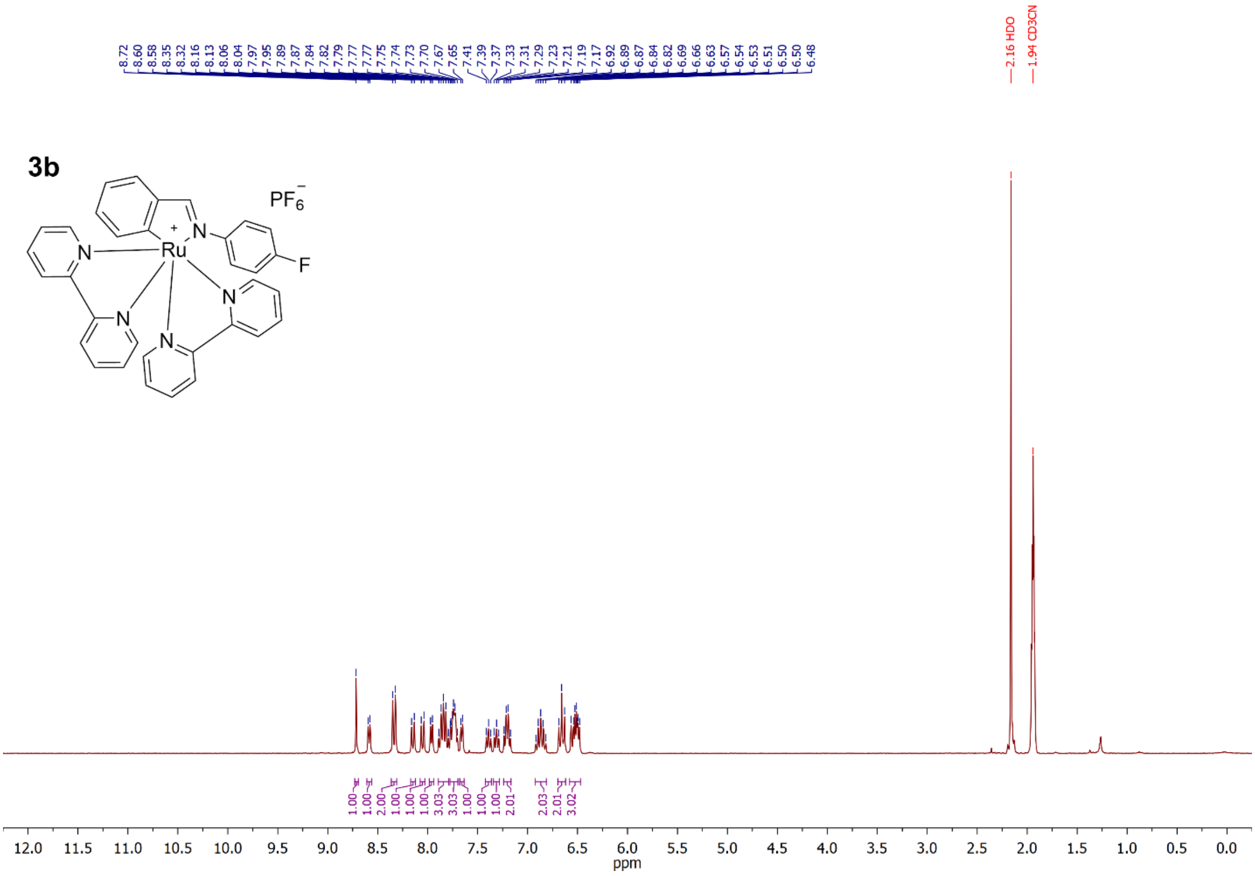

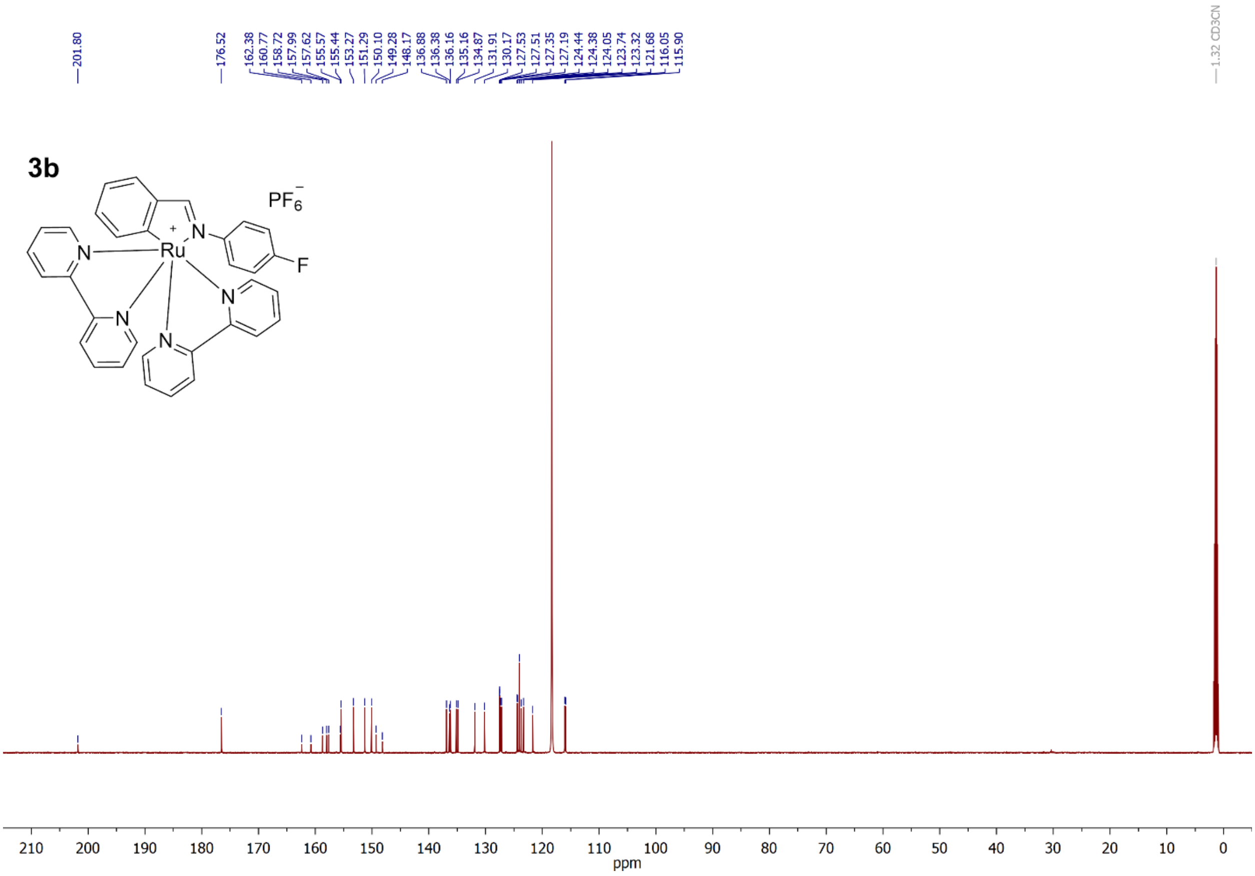

Figure S17. NMR spectra of **3b**.

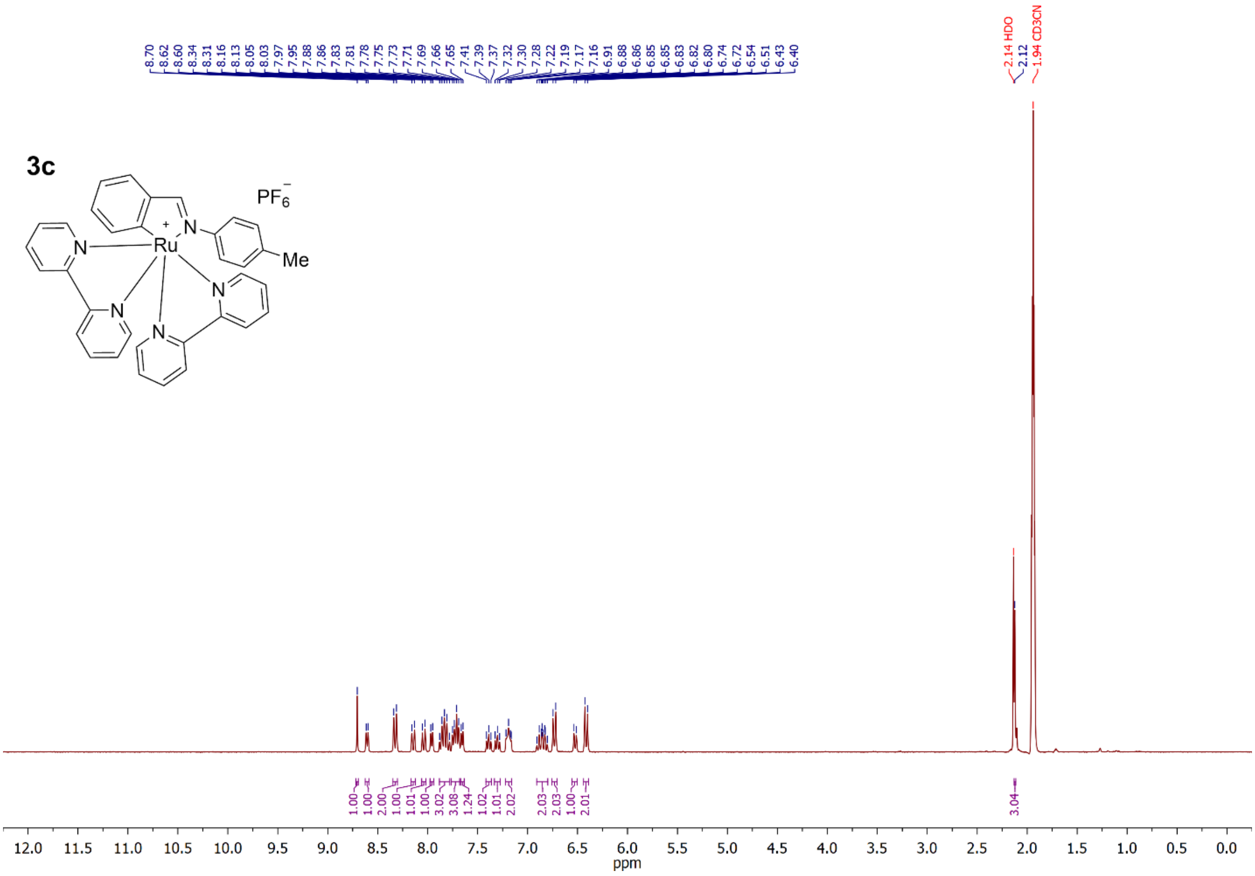

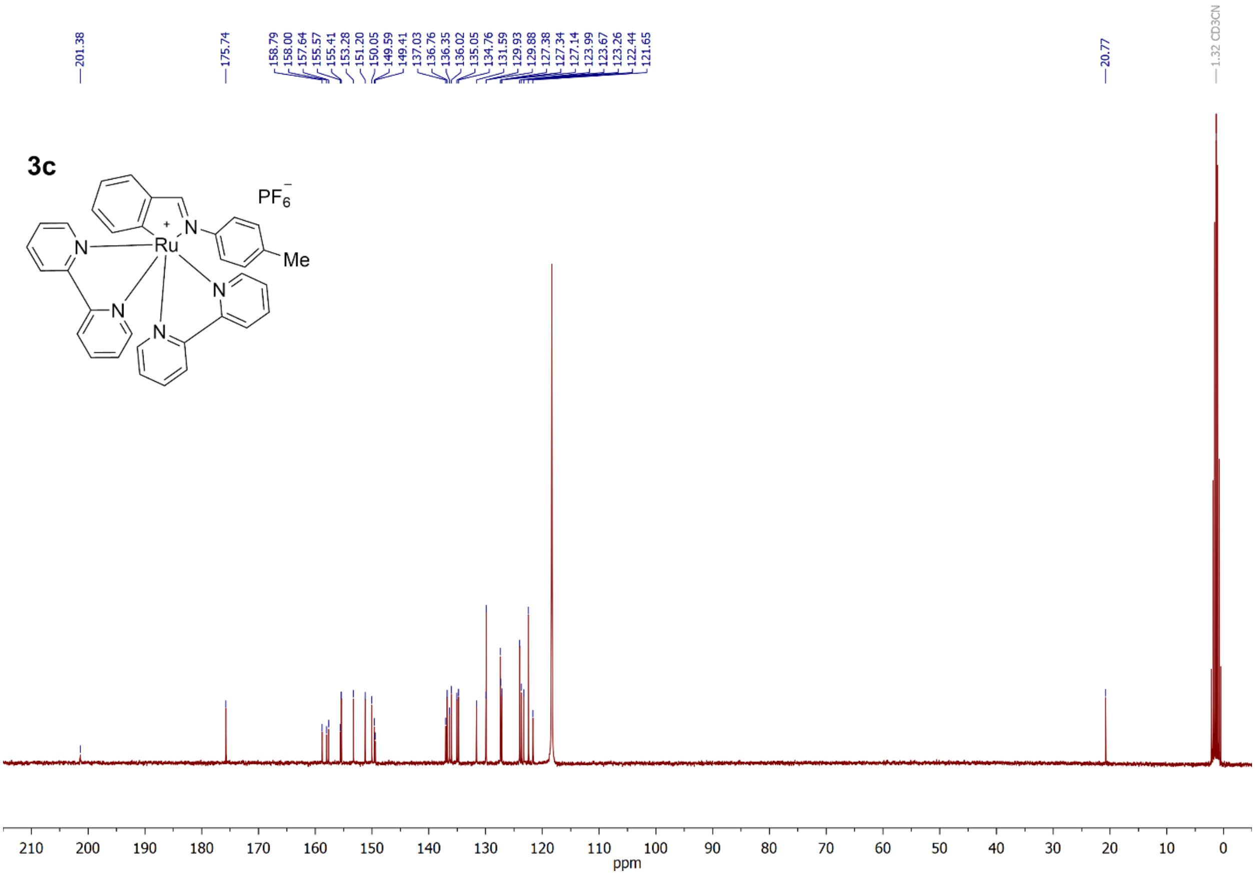

Figure S18. NMR spectra of **3c**.

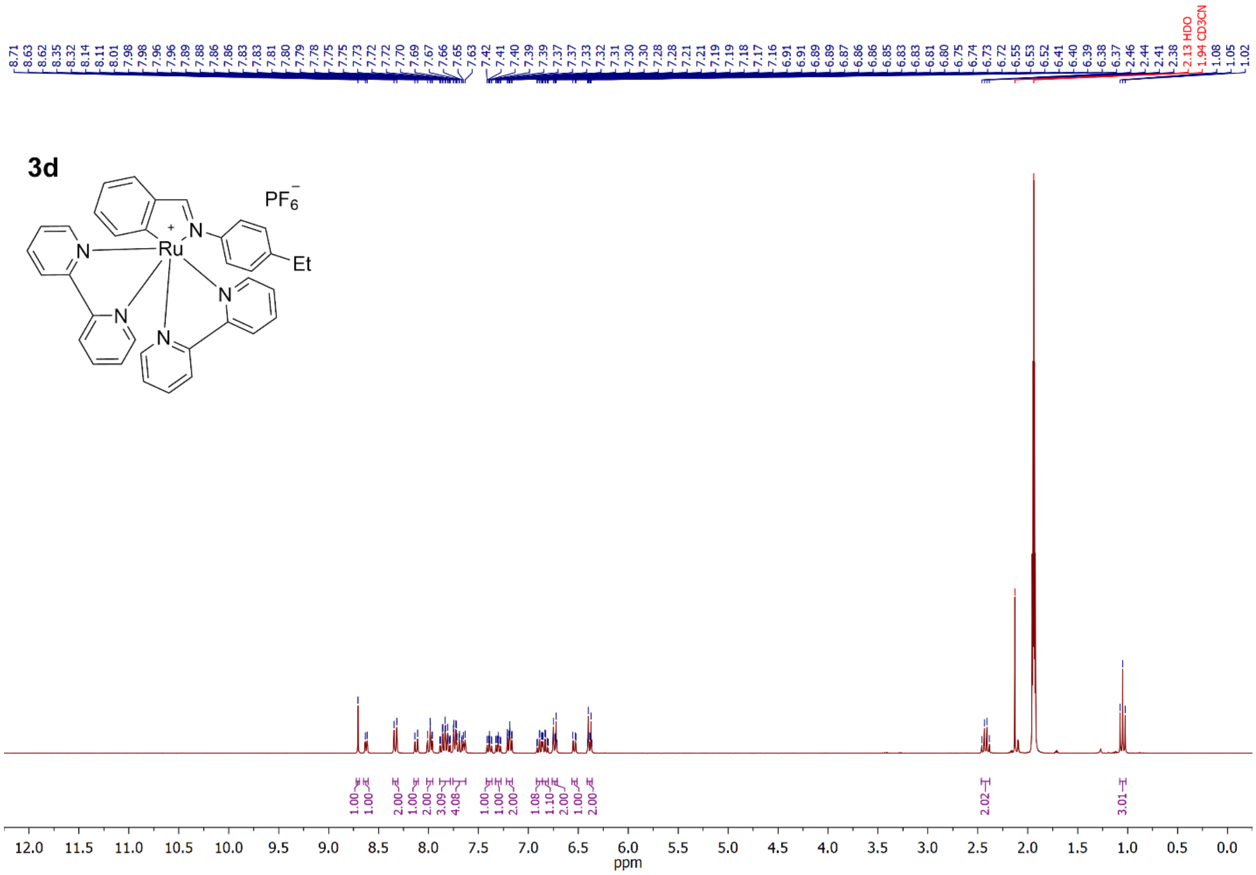

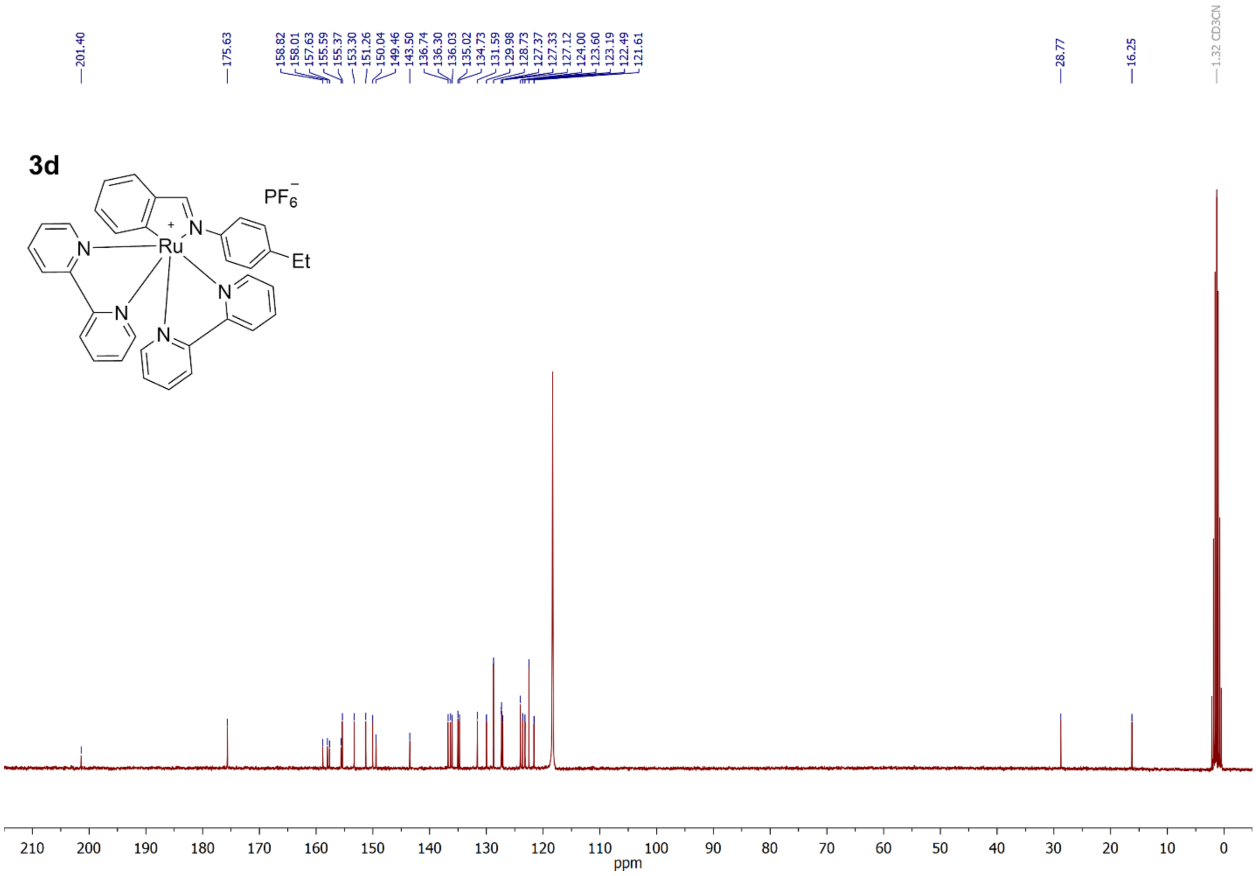

Figure S19. NMR spectra of **3d**.

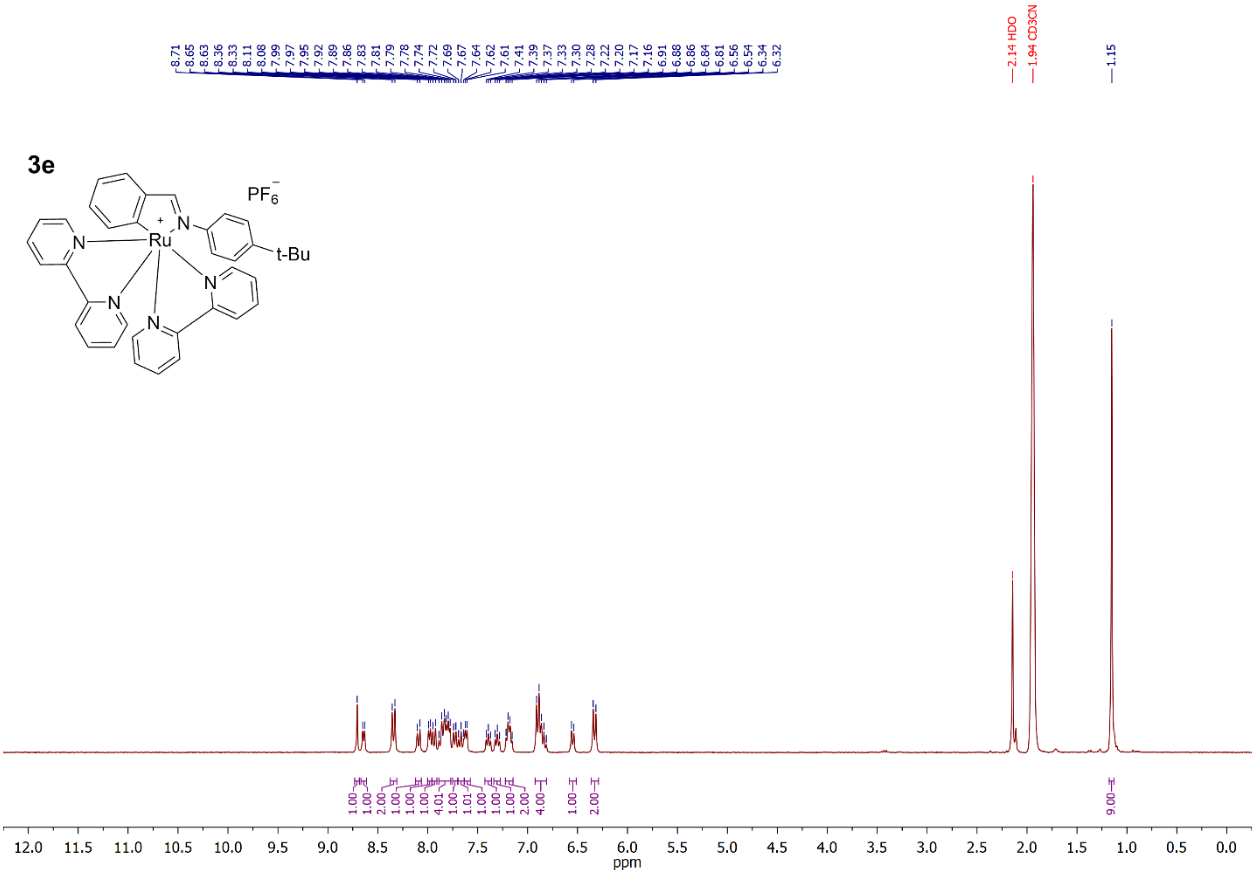

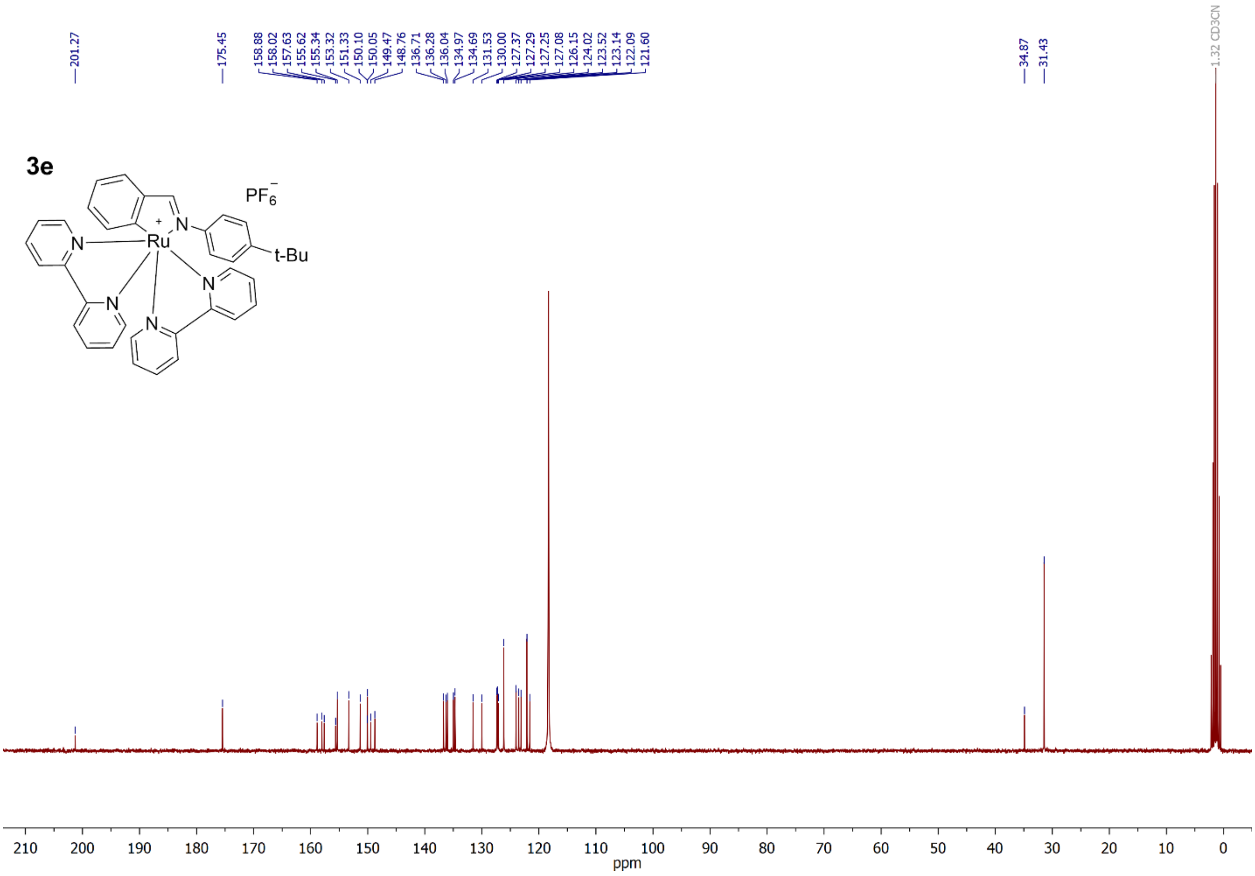

Figure S20. NMR spectra of **3e**.

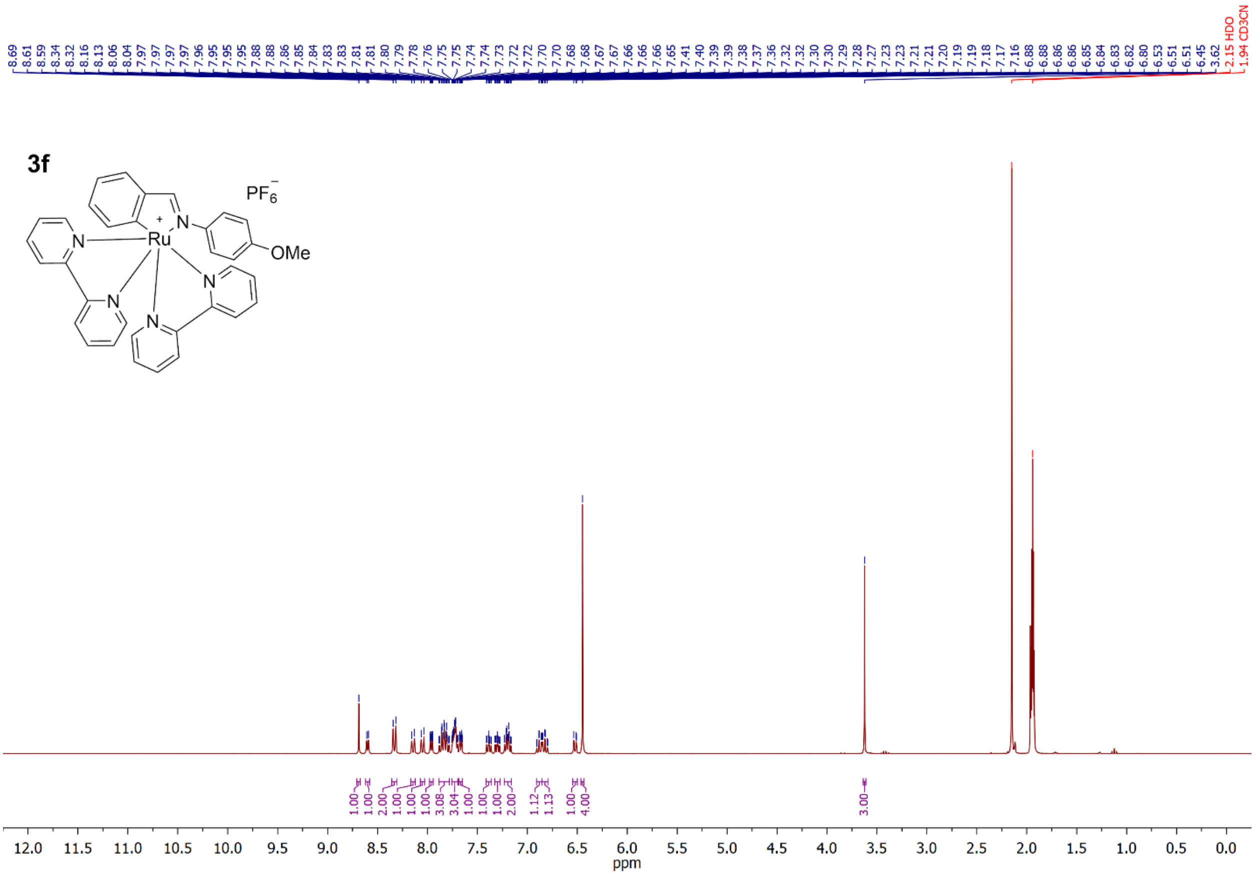

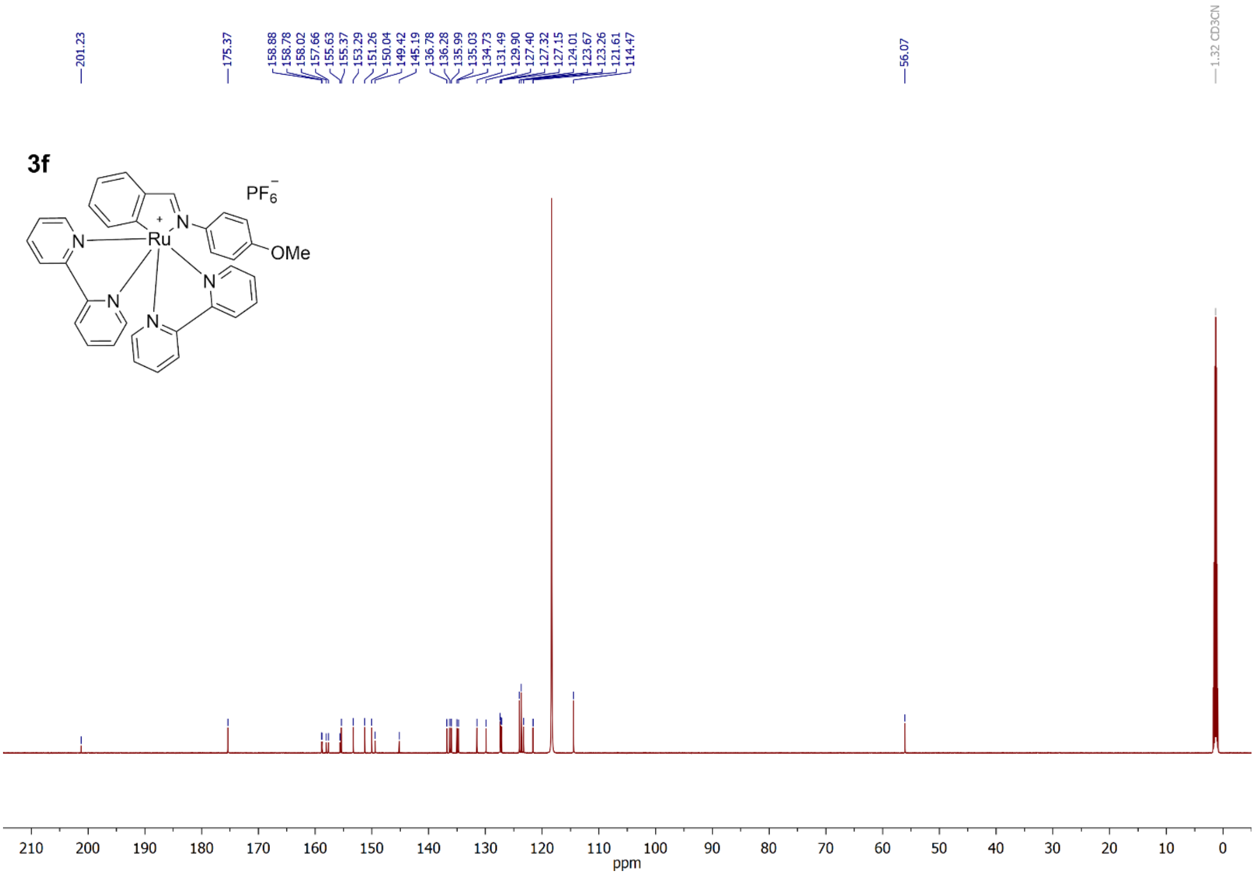

Figure S21. NMR spectra of **3f**.

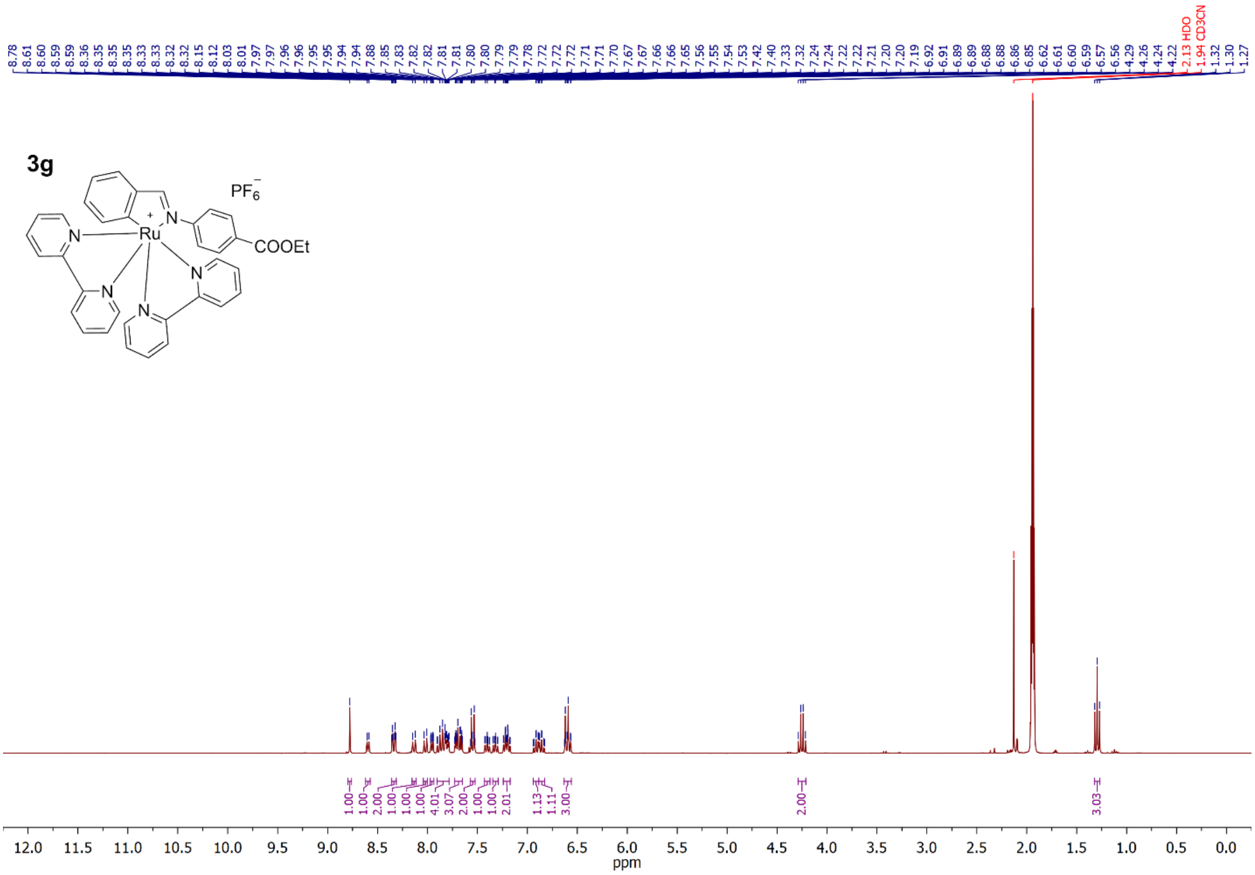

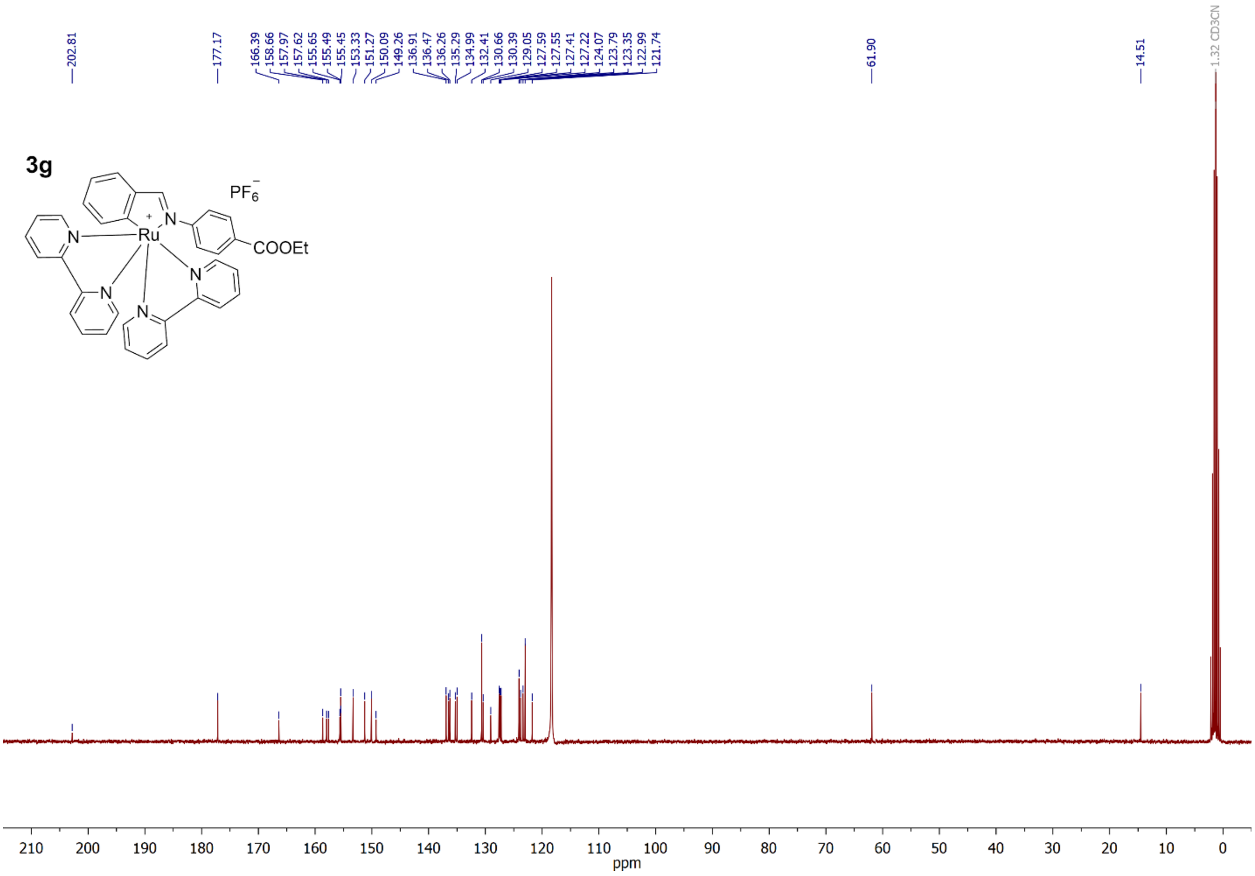

Figure S22. NMR spectra of **3g**.

3h

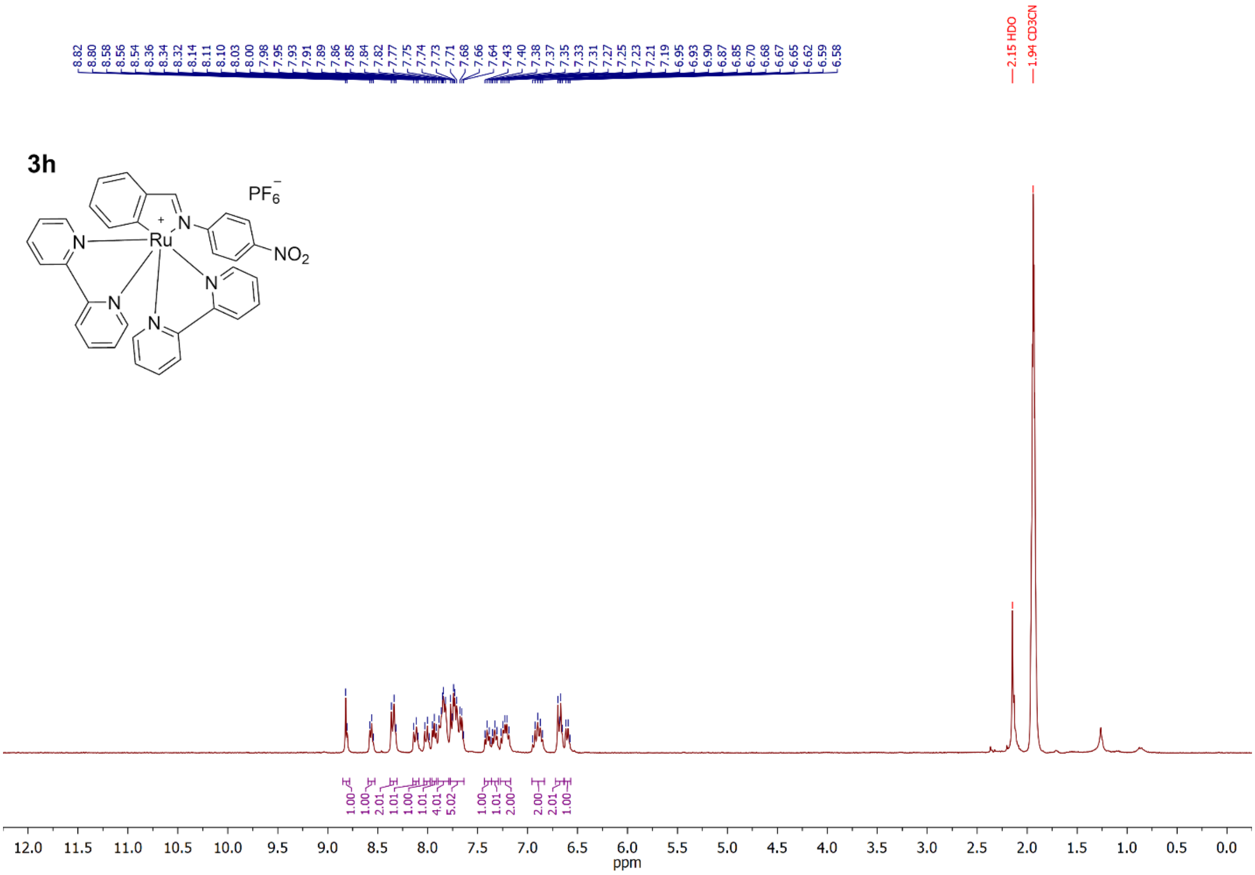

3h

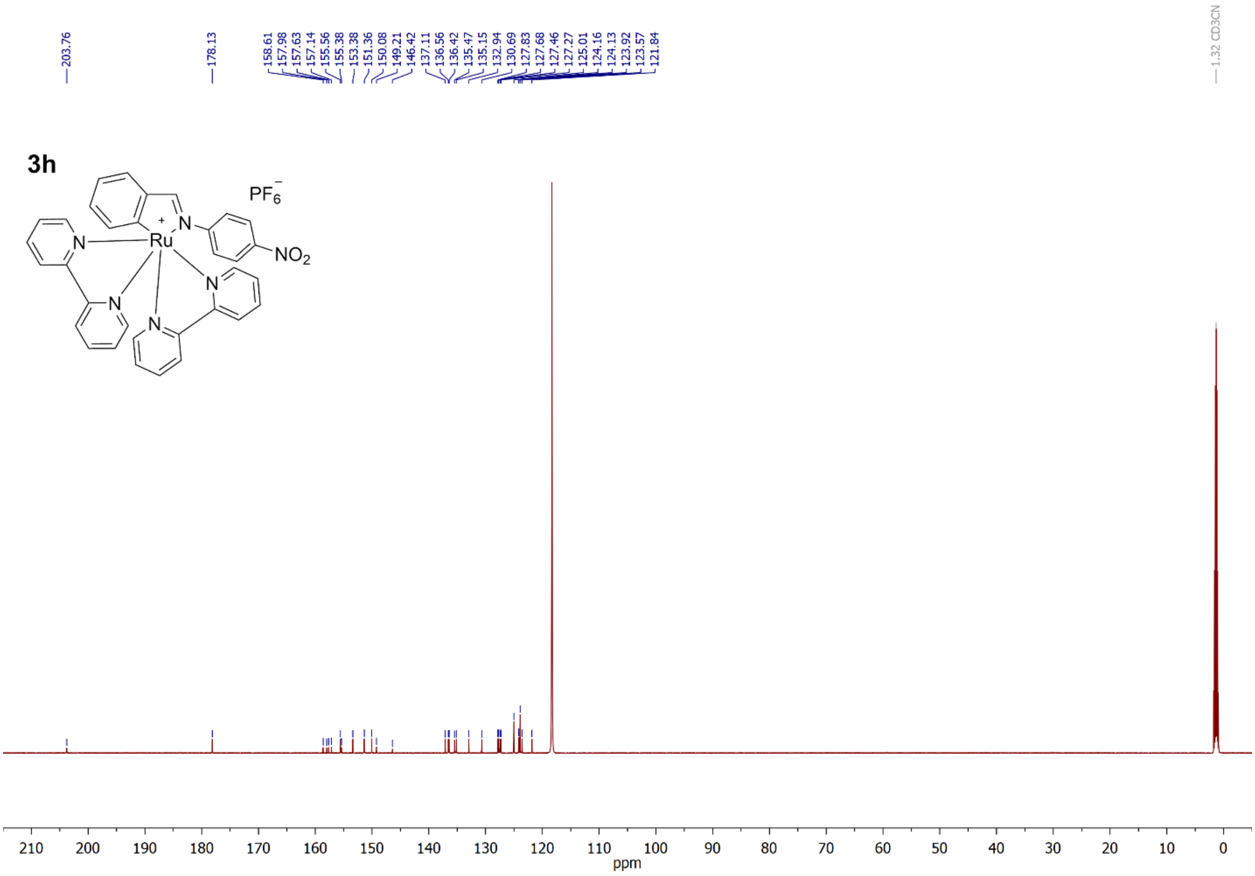

Figure S23. NMR spectra of **3h**.

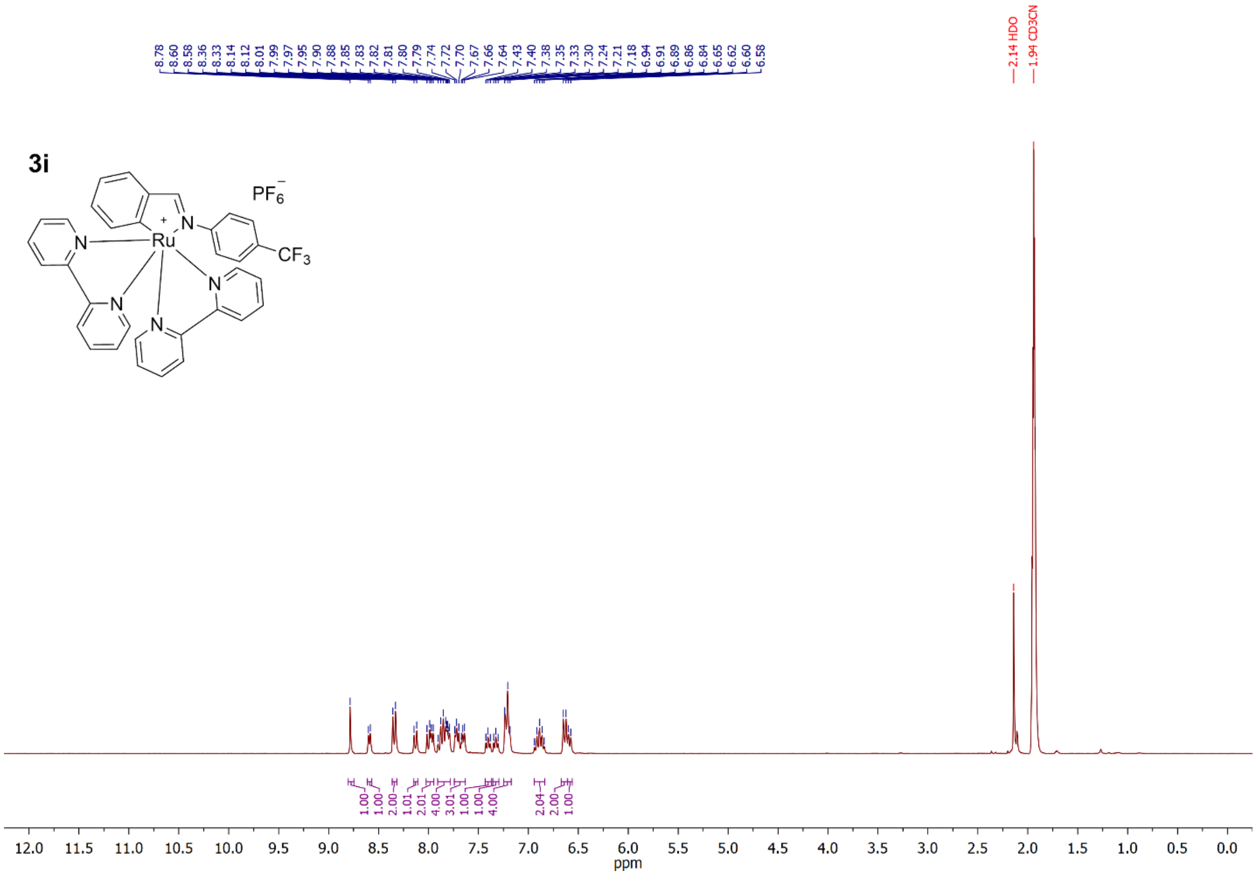

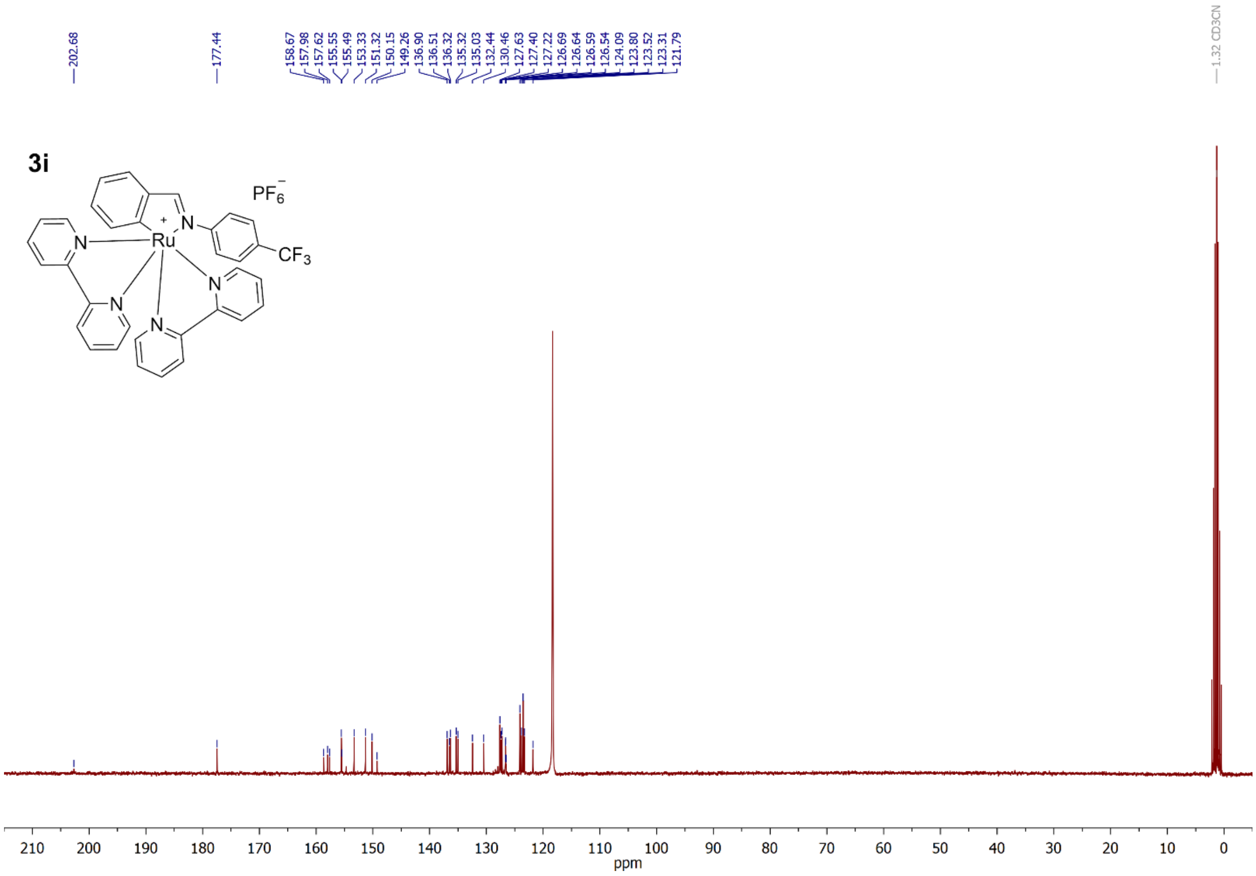

Figure S24. NMR spectra of **3i**.

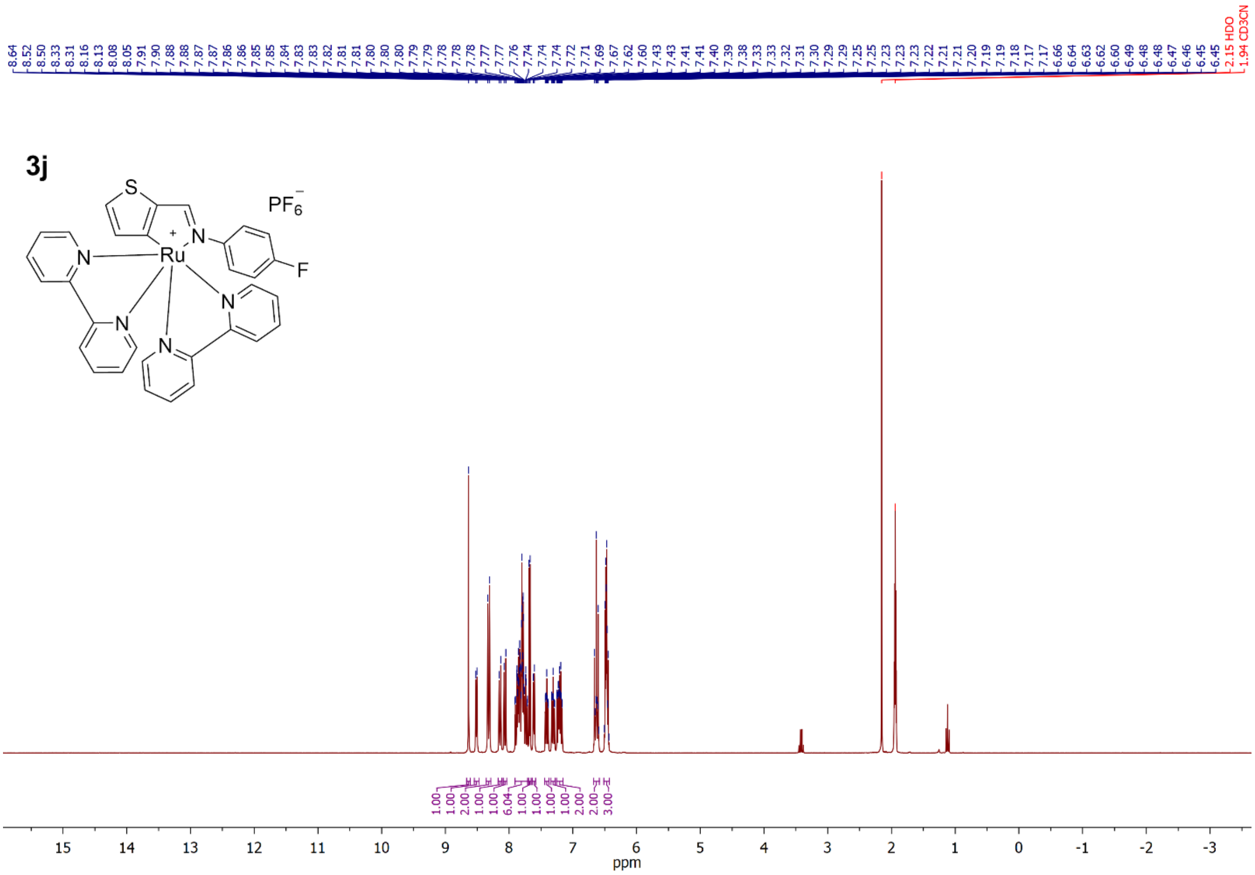

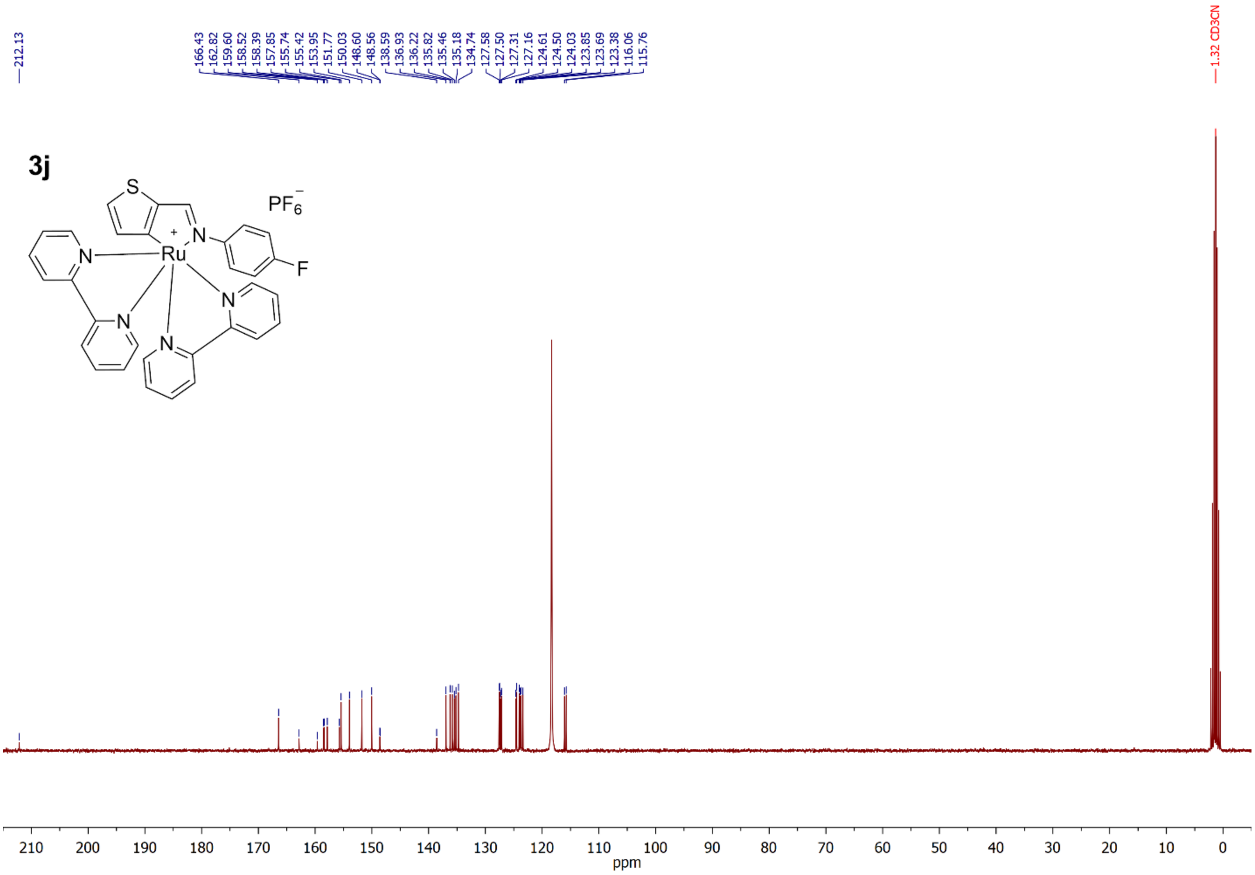

Figure S25. NMR spectra of **3j**.

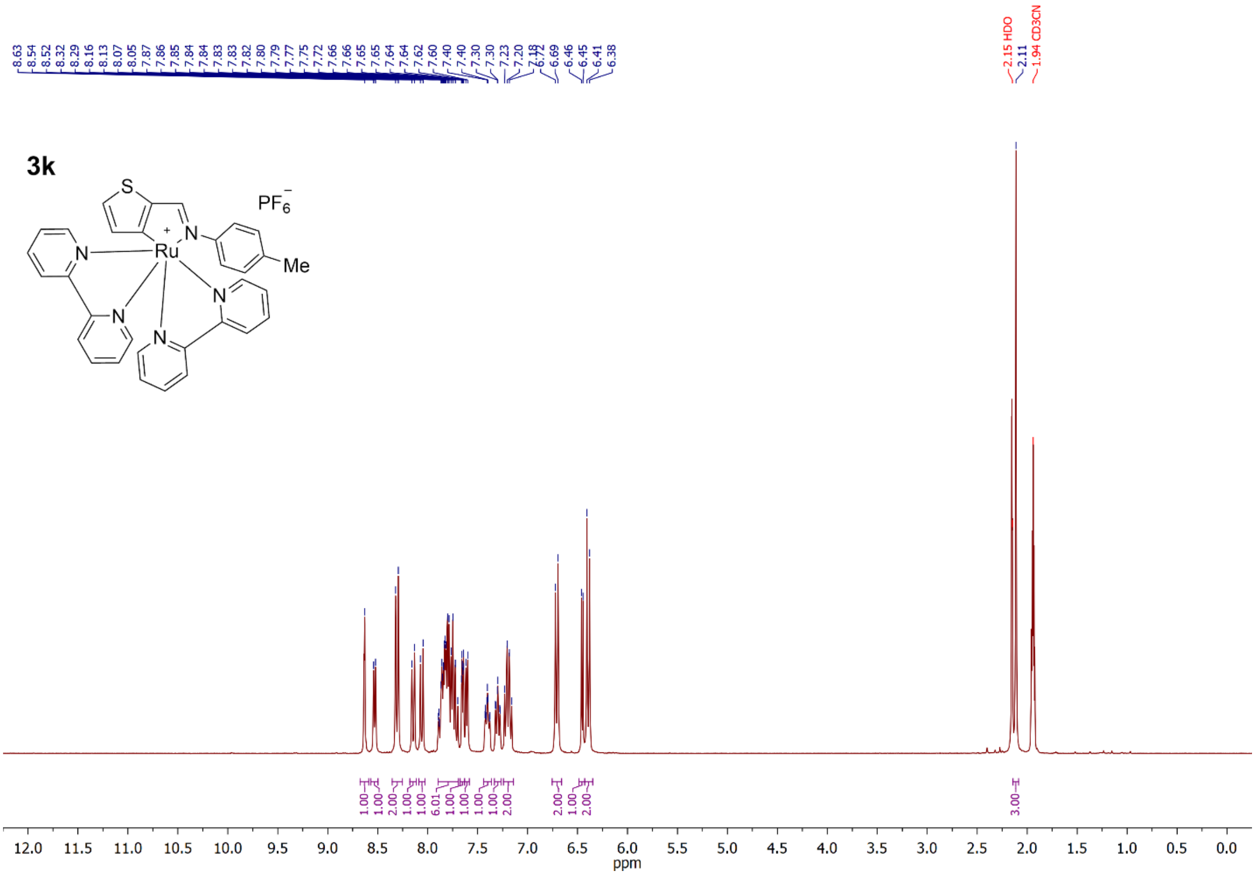

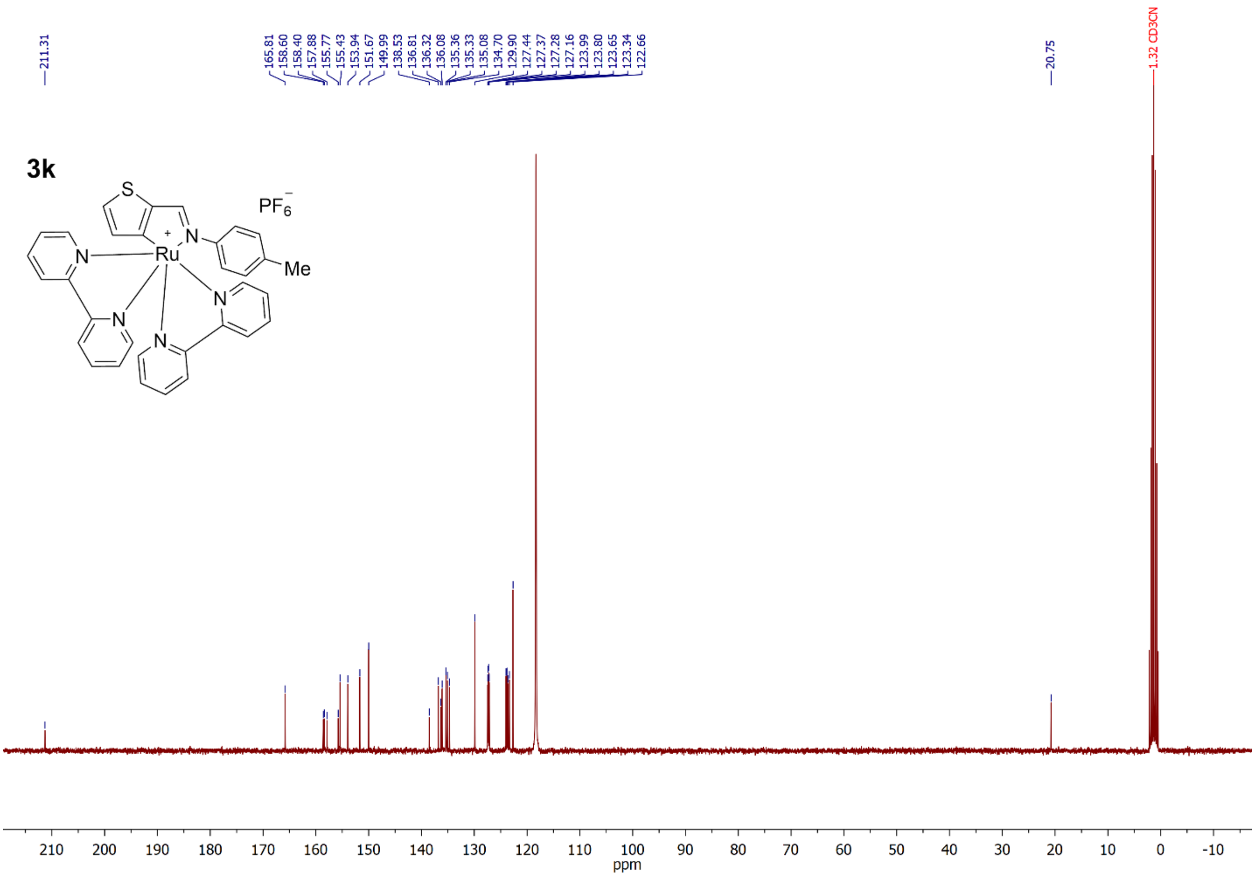

Figure S26. NMR spectra of **3k**.

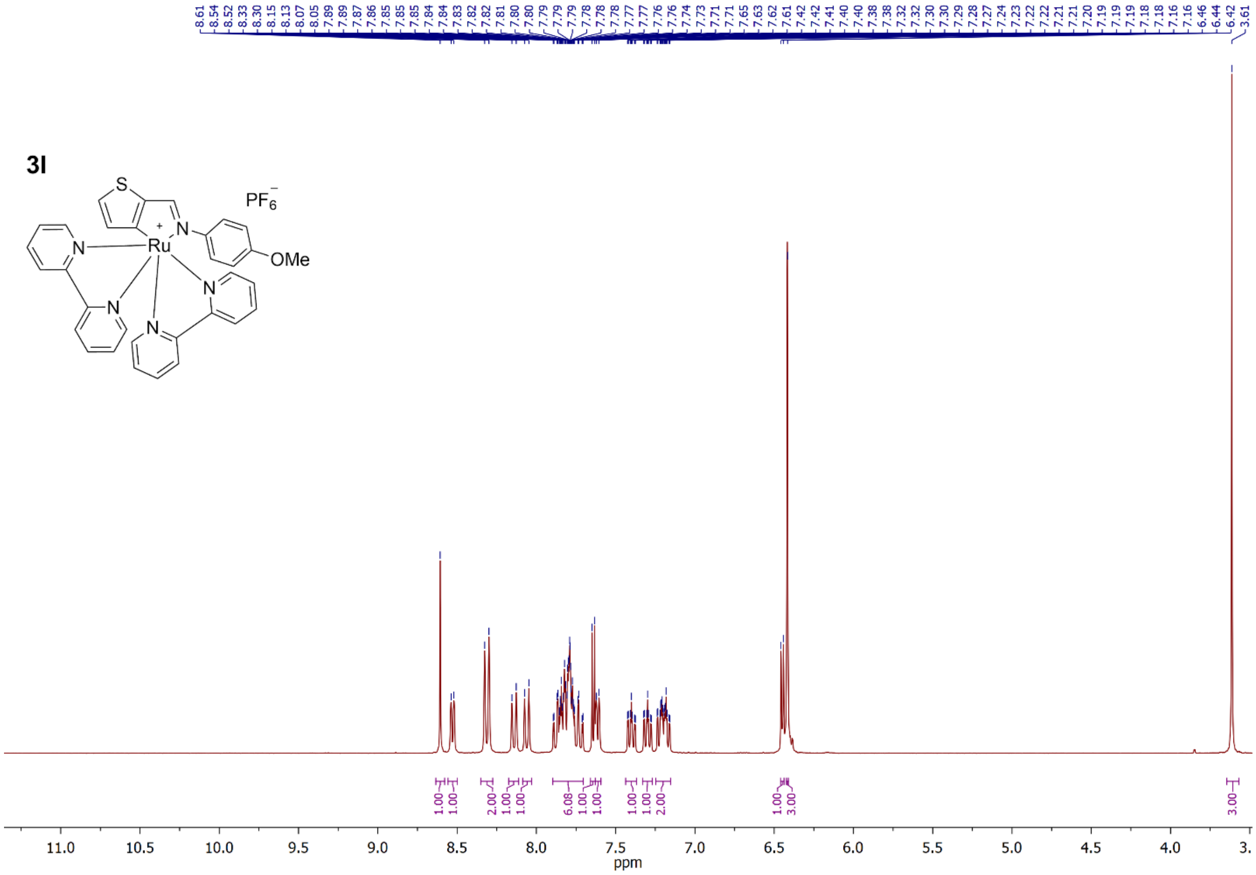

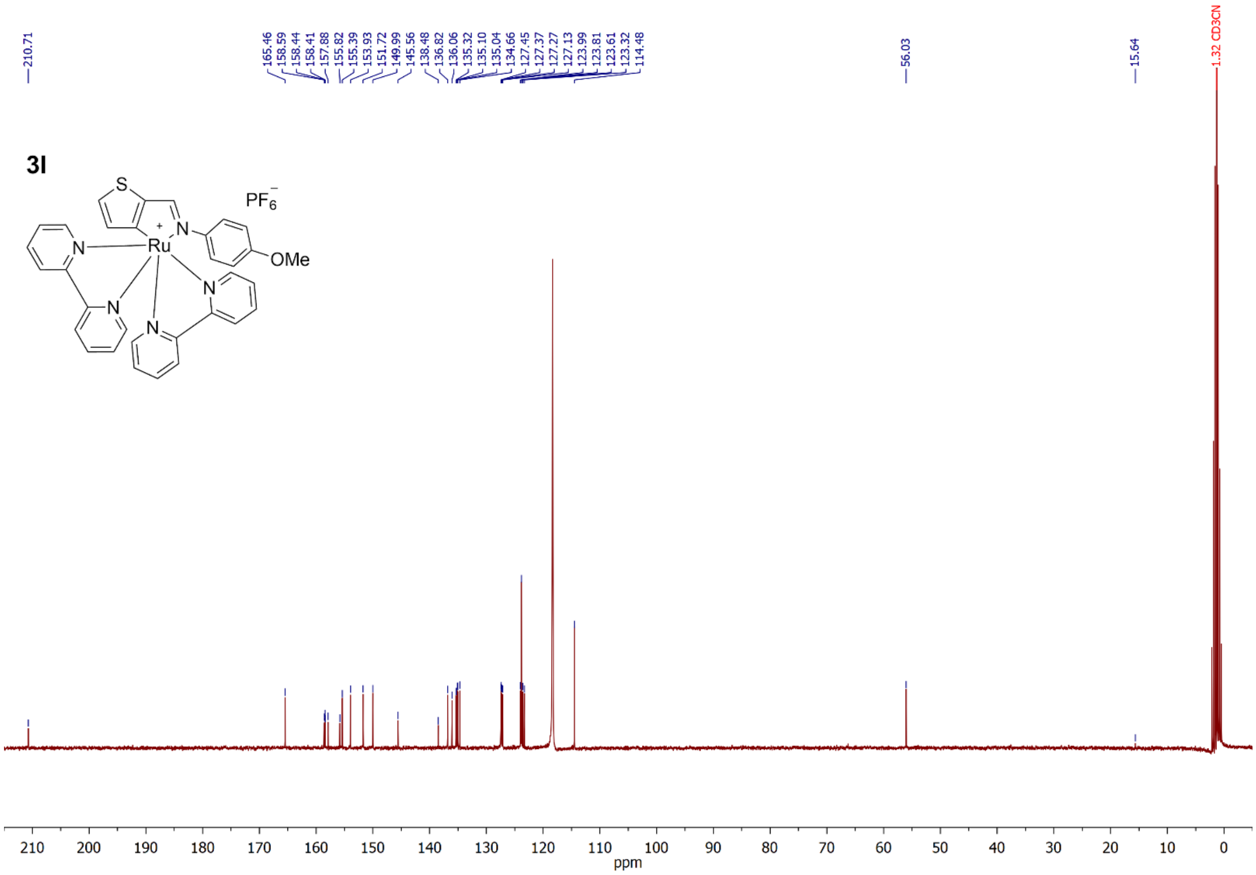

Figure S27. NMR spectra of **3l**.

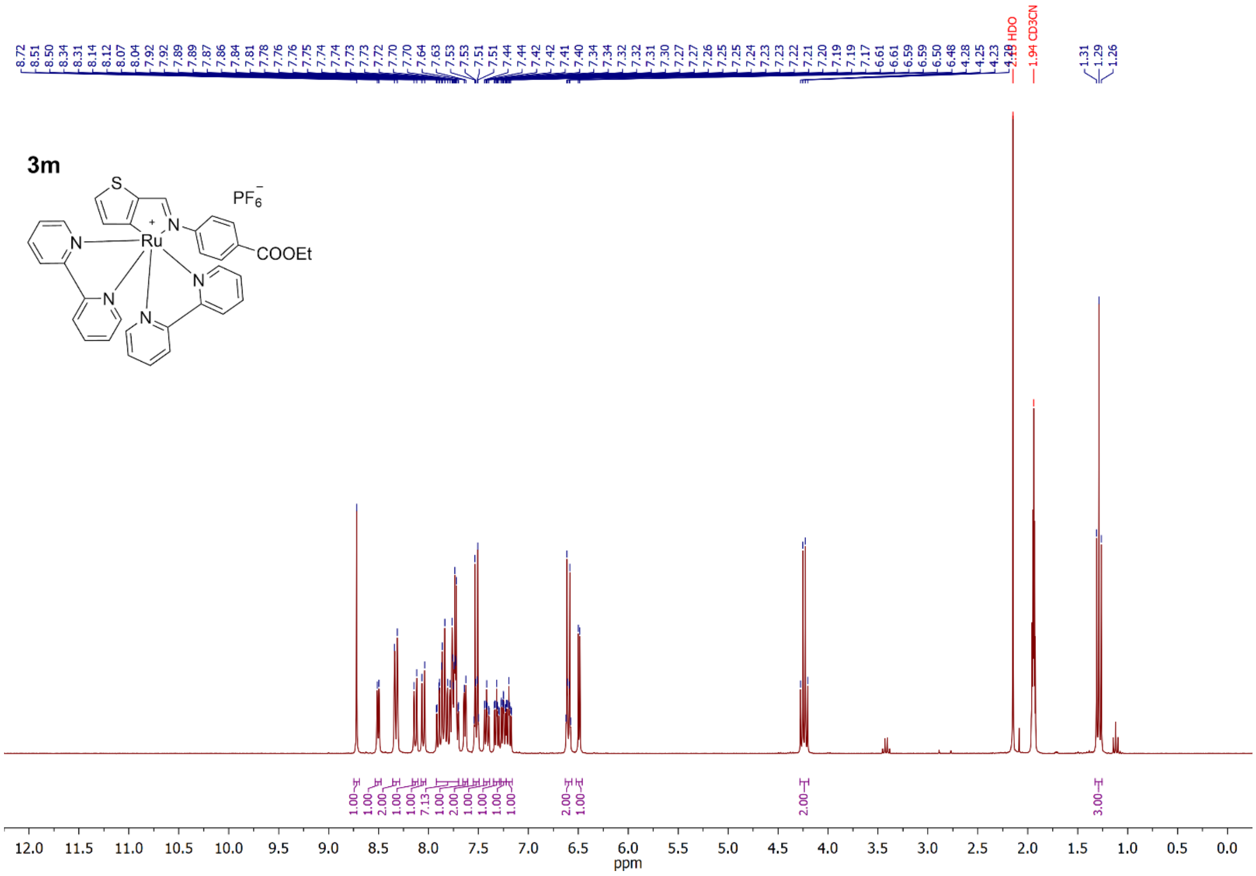

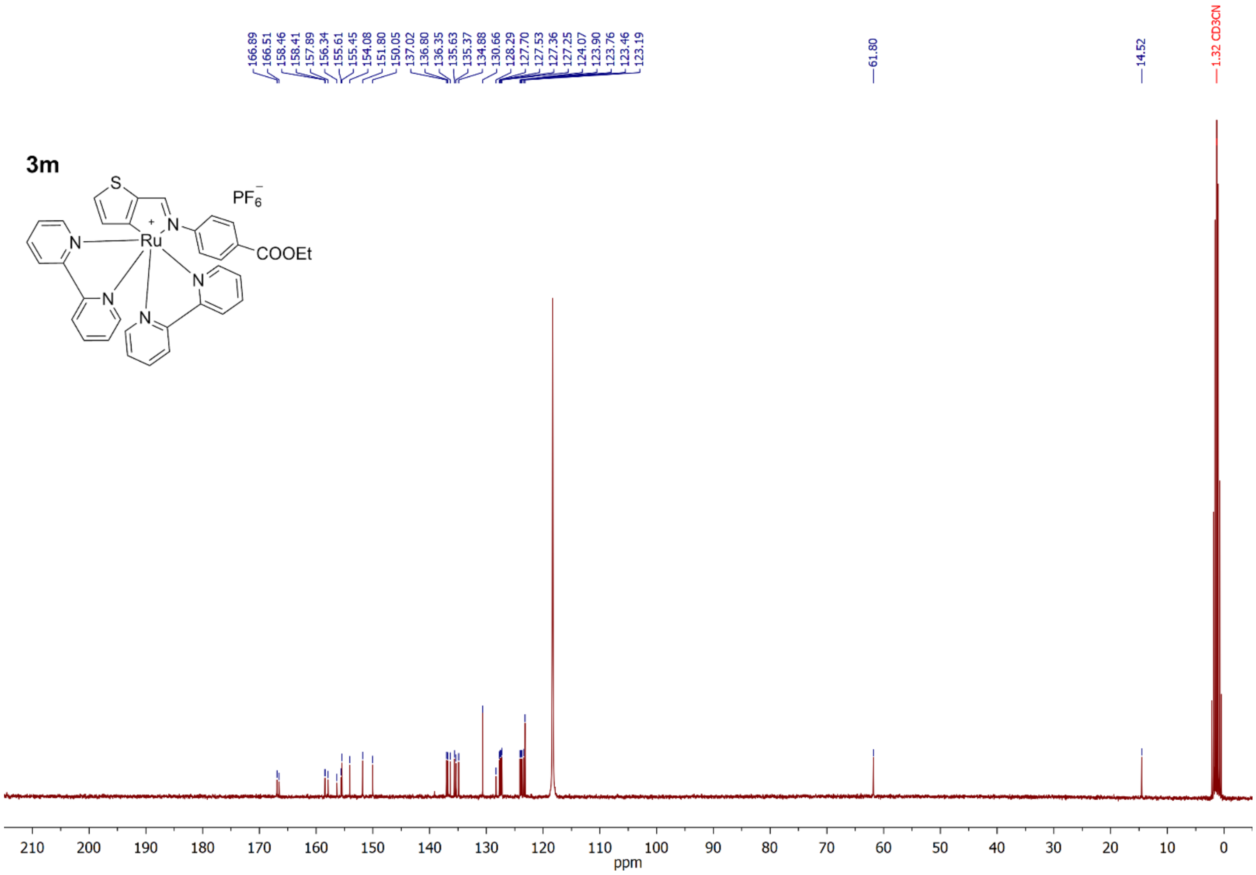

Figure S28. NMR spectra of **3m**.

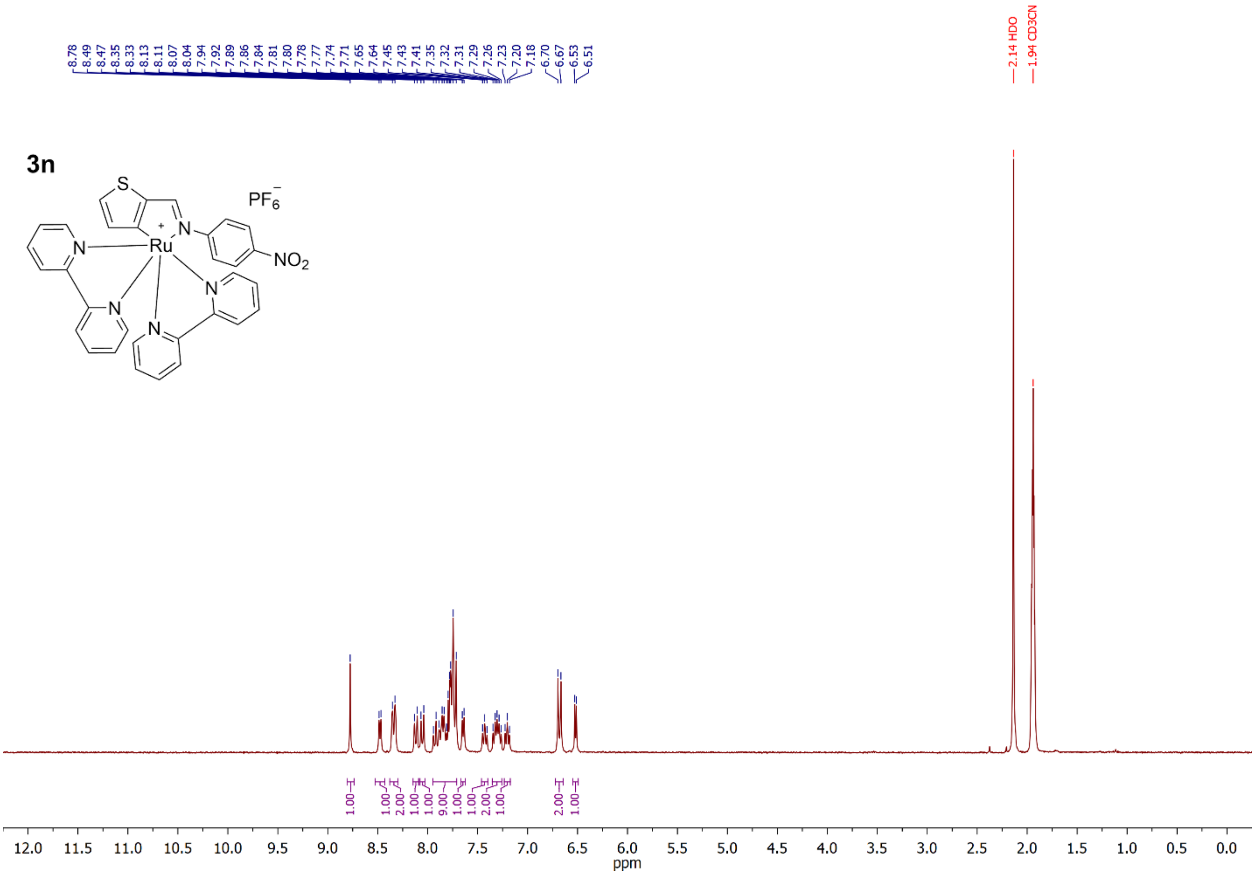

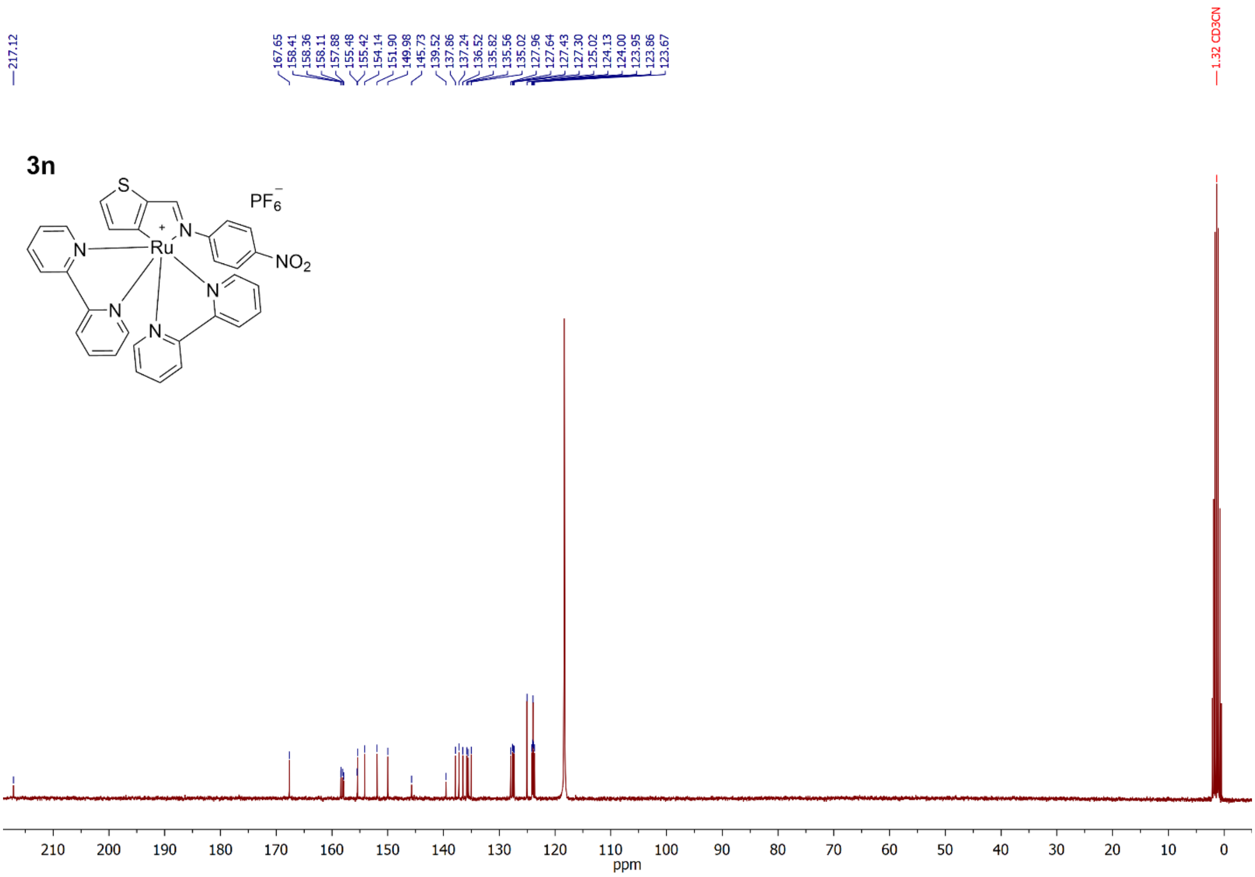

Figure S29. NMR spectra of **3n**.

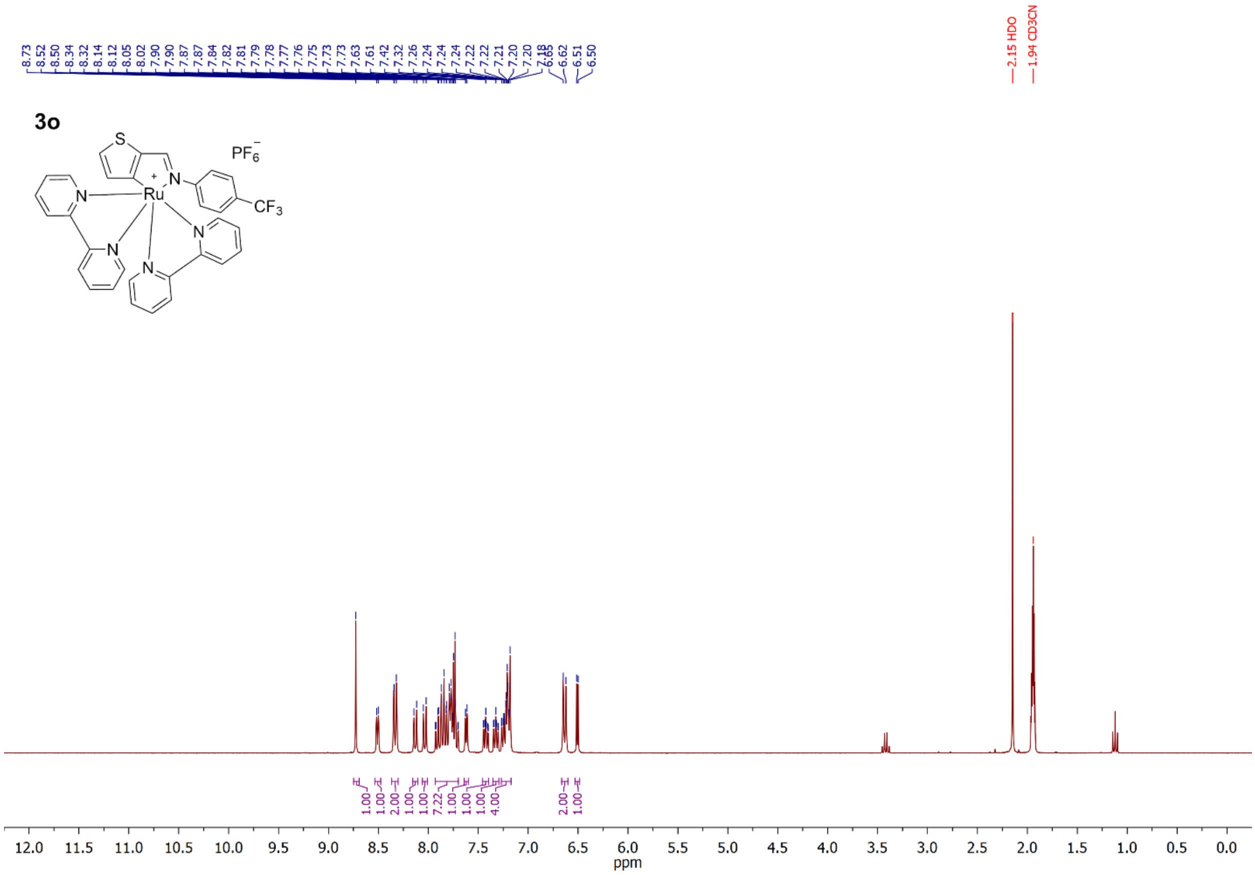

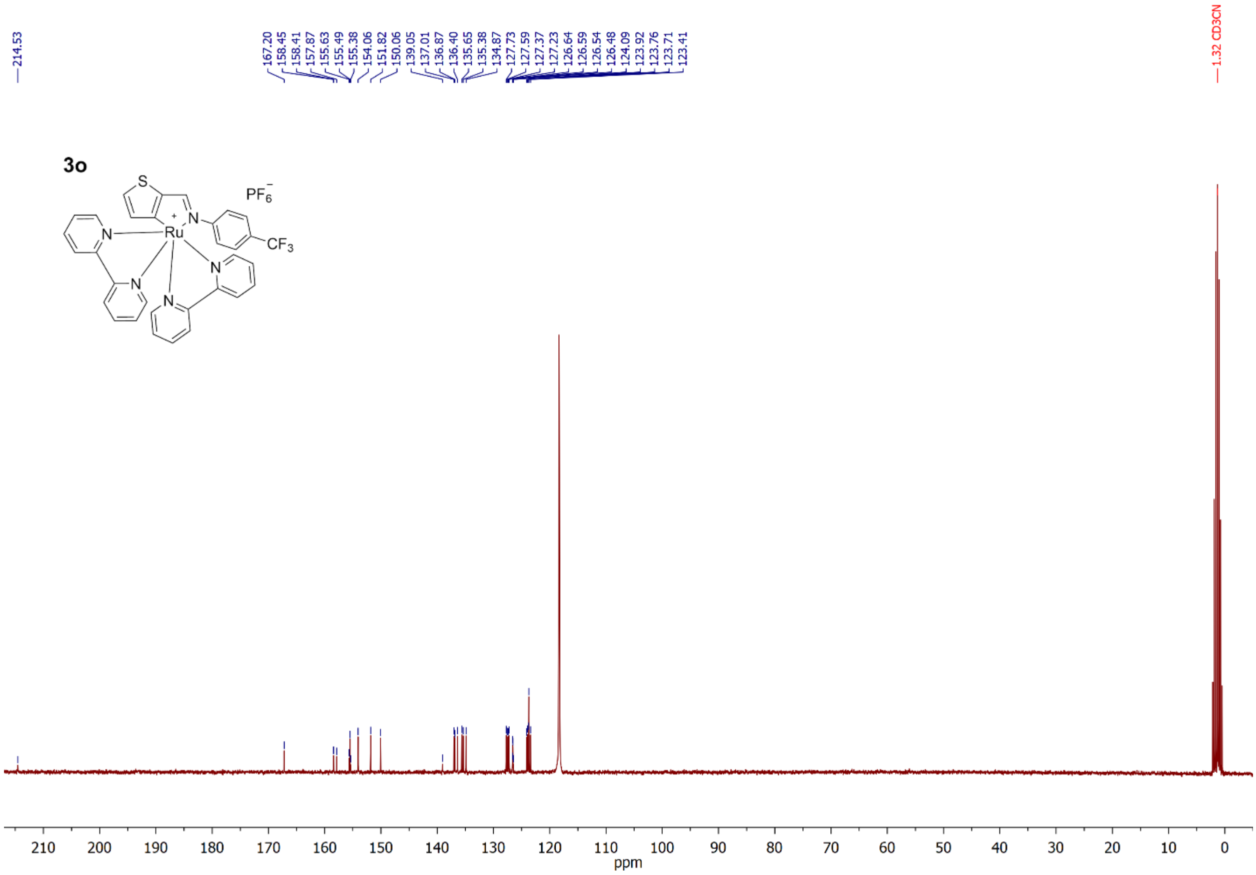

Figure S30. NMR spectra of **3o**.

Table S1. Structure parameters of ruthenium complexes

	**2a***0.8CH3CN	**2b***CH3CN	**2d**	**2e**
Empirical formula	$C_{21.80}H_{23.20}F_6N_{5.40}PRu$	$C_{22}H_{22.50}F_7N_{5.50}PRu$	$C_{23}H_{26}F_6N_5PRu$	$C_{25}H_{30}F_6N_5PRu$
Formula weight	606.90	628.99	618.53	646.58
Temperature, K	100.00	100.15		100.15
Wavelength, Å	0.71073	1.54184	1.54184	0.71073
Crystal system	Triclinic	Triclinic	Monoclinic	Triclinic
Space group	P-1	P-1	P 2_1/c	P-1
Unit cell dimensions				
a, Å	8.8365(3)	8.8149(2)	11.25950(10)	8.45880(10)
b, Å	11.6174(4)	12.0070(2)	21.85140(10)	12.80830(10)
c, Å	13.2729(5)	13.4216(2)	11.72570(10)	13.41760(10)
α, °	102.9410(10)	104.459(2)	90	92.1060(10)
β, °	102.1310(10)	102.197(2)	114.7490(10)	101.5280(10)
γ, °	96.3880(10)	99.947(2)	90	99.9540(10)
Volume, $Å^3$	1280.25(8)	1305.79(4)	2619.96(4)	1399.10(2)
Z	2	2	4	2
Density (calculated), g/cm^3	1.574	1.600	1.568	1.535
Absorption coefficient, mm^{-1}	0.739	6.097	6.003	0.681
F(000)	610	630	1248	656
Crystal size, mm^3	0.695 x 0.415 x 0.336	0.35 x 0.24 x 0.15	0.47 x 0.3 x 0.13	0.35 x 0.19 x 0.08
Theta range for data collection	2.543 to 33.175	3.529 to 80.124	4.046 to 80.037	2.159 to 35.304
Reflections collected	66386	34569	35764	66639
Independent reflections	9771	5649	5703	11863
R(int)	0.0261	0.0429	0.0405	0.0307
Observed reflections	9354	5539	5581	11024
Data / restraints / parameters	9771 / 784 / 326	5649 / 172 / 336	5703 / 131 / 368	11863 / 38 / 359
Goodness-of-fit on F^2	1.054	1.036	1.051	1.041
R_1 [I>2σ (I)]	0.0288	0.0365	0.0301	0.0272
wR_2 (all data)	0.0766	0.0905	0.0753	0.0696
Largest diff. peak and hole, e*$Å^3$	0.841 and -0.869	0.518 and -0.787	0.543 and -0.669	0.937 and -0.691
CCDC number	2502950	2502941	2502939	2502947

Table S1 (continued). Structure parameters of ruthenium complexes

	**2f**	**2g***0.5Et$_2$O*0.5CH$_3$CN	**2h**	**2i**
Empirical formula	$C_{22}H_{24}F_6N_5OPRu$	$C_{27}H_{32.50}F_6N_{5.50}O_{2.50}PRu$	$C_{21}H_{21}F_6N_6O_2PRu$	$C_{22}H_{21}F_9N_5PRu$
Formula weight	620.50	720.12	635.48	658.48
Temperature, K	99.99(10)	99.97(18)	100.15	99.98(13)
Wavelength, Å	1.54184	1.54184	1.54184	1.54184
Crystal system	Triclinic	Triclinic	Monoclinic	Monoclinic
Space group	P-1	P-1	P c	P 2_1/c
Unit cell dimensions				
a, Å	8.64530(10)	8.54460(10)	11.61256(4)	10.94550(10)
b, Å	11.32120(10)	17.28890(10)	8.33269(3)	21.59160(10)
c, Å	13.8756(2)	22.9894(2)	26.25801(10)	12.12600(10)
α, °	107.6330(10)	105.8030(10)	90	90
β, °	103.6220(10)	96.5870(10)	93.8689(3)	114.8150(10)
γ, °	91.2860(10)	101.5920(10)	90	90
Volume, Å^3	1251.40(3)	3148.99(5)	2535.036(16)	2601.15(4)
Z	2	4	4	
Density (calculated), g/cm^3	1.647	1.519	1.665	1.681
Absorption coefficient, mm^{-1}	6.318	5.148	6.298	6.262
F(000)	624	1464	1272	1312
Crystal size, mm^3	0.88 x 0.53 x 0.2	0.59 x 0.45 x 0.06	0.442 x 0.216 x 0.104	0.28 x 0.27 x 0.16
Theta range for data collection	3.456 to 80.408	2.738 to 80.128	3.374 to 80.131	4.095 to 80.049
Reflections collected	33371	84174	66221	35846
Independent reflections	5411	13614	10703	5662
R(int)	0.0516	0.0435	0.0402	0.0376
Observed reflections	5386	13295	10675	5633
Data / restraints / parameters	5411 / 70 / 323	13614 / 99 / 834	10703 / 44 / 661	5662 / 24 / 338
Goodness-of-fit on F^2	1.110	1.039	1.032	1.053
R_1 [I>2σ (I)]	0.0370	0.0344	0.0306	0.0338
wR_2 (all data)	0.0954	0.0888	0.0793	0.0797
Largest diff. peak and hole, e*Å^3	0.974 and -1.046	0.871 and -0.791	0.715 and -0.699	0.912 and -0.967
CCDC number	2502937	2502951	2502949	2502940

Table S1 (continued). Structure parameters of ruthenium complexes

	**2j**	**2k**	**2l**	**2n**
Empirical formula	$C_{19}H_{19}F_7N_5PRuS$	$C_{20}H_{22}F_6N_5PRuS$	$C_{20}H_{22}F_6N_5OPRuS$	$C_{19}H_{19}F_6N_6O_2PRuS$
Formula weight	614.49	610.52	626.52	641.50
Temperature, K	100.00(10)	100.00(10)	100.00(10)	100.00(10)
Wavelength, Å	1.54184	1.54184	1.54184	1.54184
Crystal system	Triclinic	Triclinic	Triclinic	Triclinic
Space group	P-1	P-1	P-1	P-1
Unit cell dimensions				
a, Å	8.44380(10)	8.49400(10)	10.6030(2)	8.4253(3)
b, Å	11.21030(10)	11.82980(10)	10.8966(2)	12.0245(4)
c, Å	13.8196(2)	13.85960(10)	24.7104(3)	13.6424(7)
α, °	103.9940(10)	107.1030(10)	81.3310(10)	107.573(4)
β, °	107.5030(10)	107.5780(10)	88.6410(10)	106.241(4)
γ, °	95.7790(10)	97.5610(10)	63.214(2)	97.271(3)
Volume, $Å^3$	1189.09(3)	1231.22(2)	2516.33(8)	1230.96(9)
Z	2	2	4	2
Density (calculated), g/cm^3	1.716	1.647	1.654	1.731
Absorption coefficient, mm^{-1}	7.472	7.150	7.045	7.263
F(000)	612	612	1256	640
Crystal size, mm^3	0.42 x 0.157 x 0.143	0.316 x 0.126 x 0.027	0.772 x 0.489 x 0.365	0.224 x 0.199 x 0.04
Theta range for data collection	3.500 to 80.096	3.576 to 80.030	3.623 to 80.098	3.608 to 79.970
Reflections collected	31531	32933	67133	31040
Independent reflections	5148	5330	10879	5325
R(int)	0.0399	0.0370	0.0684	0.0371
Observed reflections	5098	5222	10569	5231
Data / restraints / parameters	5148 / 55 / 357	5330 / 127 / 350	10879 / 0 / 642	5325 / 557 / 373
Goodness-of-fit on F^2	1.081	1.078	1.133	1.080
R_1 [$I>2\sigma(I)$]	0.0274	0.0318	0.0432	0.0347
wR_2 (all data)	0.0716	0.0809	0.1150	0.0917
Largest diff. peak and hole, e*$Å^3$	0.624 and -0.637	0.737 and -0.729	1.388 and -1.622	0.897 and -0.981
CCDC number	2502936	2502938	2502948	2502935

Table S1 (continued). Structure parameters of ruthenium complexes

	**3a***CH3CN	**3b***Et_2O	**3c***1.5Et_2O	**3e***CH_3CN
Empirical formula	$C_{35}H_{29}F_6N_6PRu$	$C_{37}H_{35}F_7N_5OPRu$	$C_{37}H_{35.50}F_6N_5O_{0.75}PRu$	$C_{39}H_{37}F_6N_6PRu$
Formula weight	779.68	830.74	808.24	835.78
Temperature, K	99.97(13)	99.97(15)	100.0(2)	100.00
Wavelength, Å	1.54184	1.54184	0.71073	0.71073
Crystal system	Monoclinic	Triclinic	Triclinic	Triclinic
Space group	P 2_1/n	P-1	P-1	P-1
Unit cell dimensions				
a, Å	11.98880(10)	12.4931(2)	11.1184(2)	11.7141(2)
b, Å	13.08430(10)	13.2824(2)	12.9449(2)	12.5133(2)
c, Å	20.89010(10)	13.5109(2)	13.0295(2)	12.9723(2)
α, °	90	100.0660(10)	83.2850(10)	83.7450(10)
β, °	92.9950(10)	116.292(2)	75.7620(10)	89.7810(10)
γ, °	90	111.075(2)	71.9200(10)	71.7860(10)
Volume, $Å^3$	3272.45(4)	1719.56(6)	1726.26(5)	1794.56(5)
Z	4	2	2	2
Density (calculated), g/cm^3	1.583	1.604	1.555	1.547
Absorption coefficient, mm^{-1}	4.959	4.813	0.571	0.551
F(000)	1576	844	823	852
Crystal size, mm^3	0.631 x 0.256 x 0.146	0.63 x 0.33 x 0.19	0.61 x 0.28 x 0.1	0.423 x 0.318 x 0.214
Theta range for data collection	3.988 to 80.170	3.890 to 80.034	2.176 to 35.385	2.084 to 33.197
Reflections collected	45013	45842	82597	111797
Independent reflections	7134	7422	14589	13707
R(int)	0.0407	0.0499	0.0430	0.0348
Observed reflections	7008	7353	13128	12549
Data / restraints / parameters	7134 / 3 / 455	7422 / 21 / 463	14589 / 64 / 440	13707 / 0 / 482
Goodness-of-fit on F^2	1.117	1.048	1.044	1.041
R_1 [I>2σ (I)]	0.0333	0.0404	0.0321	0.0239
wR_2 (all data)	0.0935	0.1060	0.0847	0.0590
Largest diff. peak and hole, e*$Å^3$	1.044 and -1.356	1.355 and -1.200	1.440 and -0.664	0.551 and -0.675
CCDC number	2502943	2502942	2502952	2502954

Table S1 (continued). Structure parameters of ruthenium complexes

	**3f***CH_3CN	**3h***CH_3CN	**3j***CH_3CN	**3l***CH_3CN
Empirical formula	$C_{36}H_{31}F_6N_6OPRu$	$C_{35}H_{28}F_6N_7O_2PRu$	$C_{33}H_{26}F_7N_6PRuS$	$C_{34}H_{29}F_6N_6OPRuS$
Formula weight	809.71	824.68	803.70	815.73
Temperature, K	99.98(13)	100.15	100.00(10)	100.00
Wavelength, Å	1.54184	1.54184	1.54184	0.71073
Crystal system	Monoclinic	Monoclinic	Monoclinic	Triclinic
Space group	P 2_1/n	P 2_1/n	P 2_1/n	P-1
Unit cell dimensions				
a, Å	13.11770(6)	13.04570(10)	12.15020(10)	11.1999(2)
b, Å	11.20731(5)	11.09040(10)	13.10690(10)	11.8657(2)
c, Å	23.18196(9)	23.69180(10)	20.49870(10)	13.0164(2)
α, °	90	90	90	89.3440(10)
β, °	97.3765(4)	99.6870(10)	94.9890(10)	75.8140(10)
γ, °	90	90	90	75.7930(10)
Volume, $Å^3$	3379.87(2)	3378.90(4)	3252.08(4)	1623.58(5)
Z	4	4	4	2
Density (calculated), g/cm^3	1.591	1.621	1.641	1.669
Absorption coefficient, mm^{-1}	4.848	4.895	5.640	0.671
F(000)	1640	1664	1616	824
Crystal size, mm^3	0.274 x 0.222 x 0.077	0.5 x 0.28 x 0.11	0.56 x 0.406 x 0.319	0.449 x 0.314 x 0.172
Theta range for data collection	3.683 to 80.004	3.634 to 80.007	4.008 to 80.000	1.937 to 33.227
Reflections collected	47084	47102	44852	109729
Independent reflections	7341	7360	7076	12446
R(int)	0.0376	0.0341	0.0457	0.0362
Observed reflections	7125	7230	7005	11436
Data / restraints / parameters	7341 / 649 / 477	7360 / 784 / 486	7076 / 45 / 477	12446 / 623 / 468
Goodness-of-fit on F^2	1.019	1.058	1.118	1.033
R_1 [I>2σ (I)]	0.0290	0.0328	0.0333	0.0258
wR_2 (all data)	0.0722	0.0835	0.0810	0.0630
Largest diff. peak and hole, e*$Å^3$	0.663 and -0.629	0.676 and -0.845	0.720 and -0.697	0.777 and -0.676
CCDC number	2502946	2502945	2502944	2502953

Table S1 (continued). Structure parameters of ruthenium complexes

	**3m***1.25CH_3CN	**3o**	**1n**
Empirical formula	$C_{35.24}H_{29.88}F_6N_{5.62}O_2PRuS$	$C_{32}H_{23}F_9N_5PRuS$	$C_{11}H_8N_2O_2S$
Formula weight	842.19	812.65	232.25
Temperature, K	100.00	100.00	100.00(10)
Wavelength, Å	0.71073	0.71073	1.54184
Crystal system	Triclinic	Triclinic	Monoclinic
Space group	P-1	P-1	C 2/c
Unit cell dimensions			
a, Å	12.8277(5)	9.9059(4)	9.27410(10)
b, Å	13.2553(5)	12.5785(5)	11.99550(10)
c, Å	13.3198(5)	13.7546(6)	18.4996(2)
α, °	118.3860(10)	80.180(2)	90
β, °	114.0610(10)	77.243(2)	92.6890(10)
γ, °	91.9430(10)	70.897(2)	90
Volume, Å^3	1744.75(12)	1570.48(11)	2055.77(4)
Z	2	2	8
Density (calculated), g/cm^3	1.603	1.719	1.501
Absorption coefficient, mm^{-1}	0.629	0.704	2.692
F(000)	851	812	960
Crystal size, mm^3	0.28 x 0.21 x 0.12	0.568 x 0.235 x 0.186	0.356 x 0.321 x 0.18
Theta range for data collection	2.175 to 33.194	1.723 to 33.324	4.786 to 80.024
Reflections collected	119201	112703	14187
Independent reflections	13338	12017	2247
R(int)	0.0349	0.0578	0.0356
Observed reflections	12367	10140	2184
Data / restraints / parameters	13338 / 1346 / 524	12017 / 0 / 442	2247 / 0 / 146
Goodness-of-fit on F^2	1.042	1.039	1.071
R_1 [I>2σ (I)]	0.0321	0.0403	0.0336
wR_2 (all data)	0.0793	0.0982	0.0922
Largest diff. peak and hole, e*Å^3	0.924 and -0.900	1.015 and -1.396	0.322 and -0.265
CCDC number	2502956	2502955	2502934

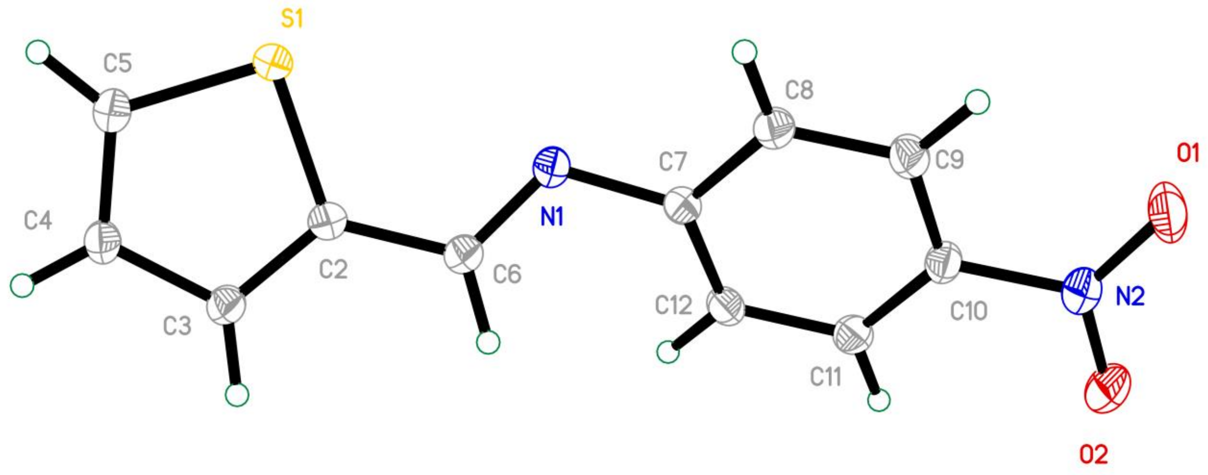

Figure S31. Crystal structure of **1n**.

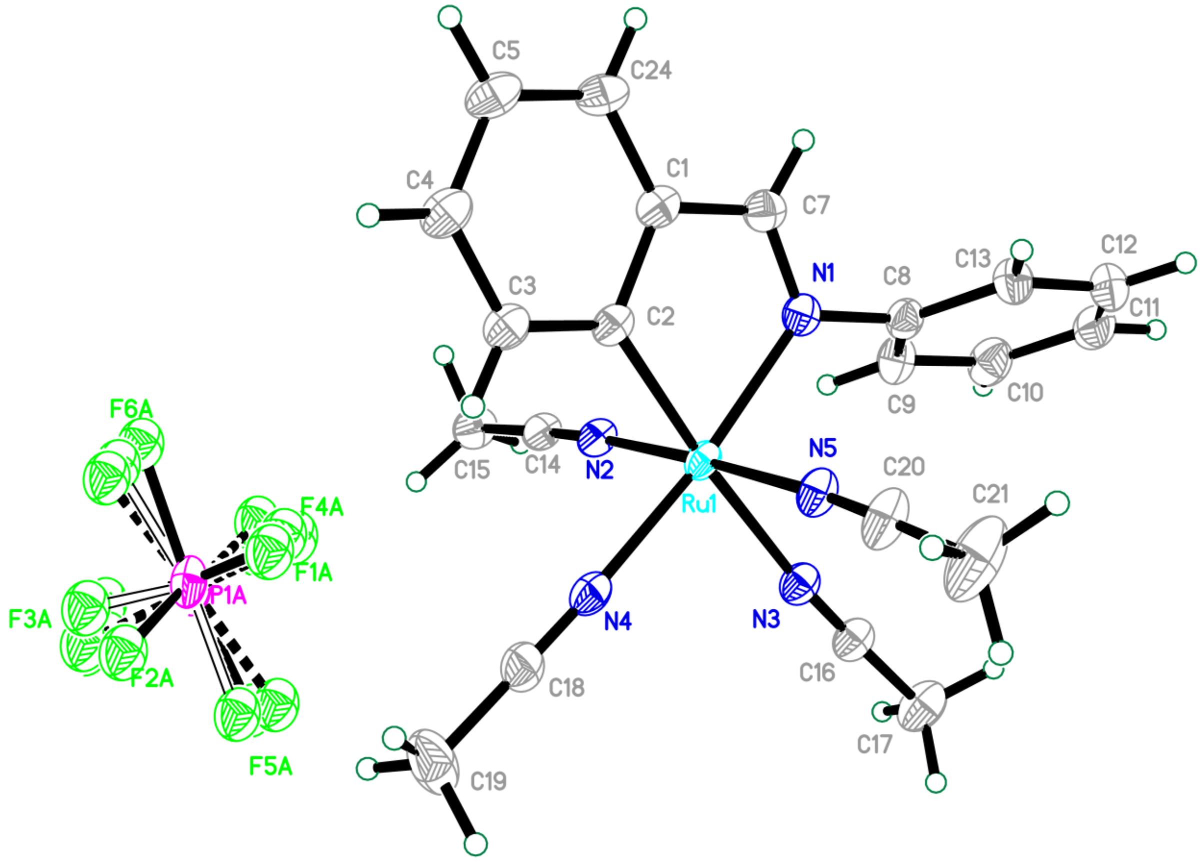

Figure S32. Crystal structure of **2a**.

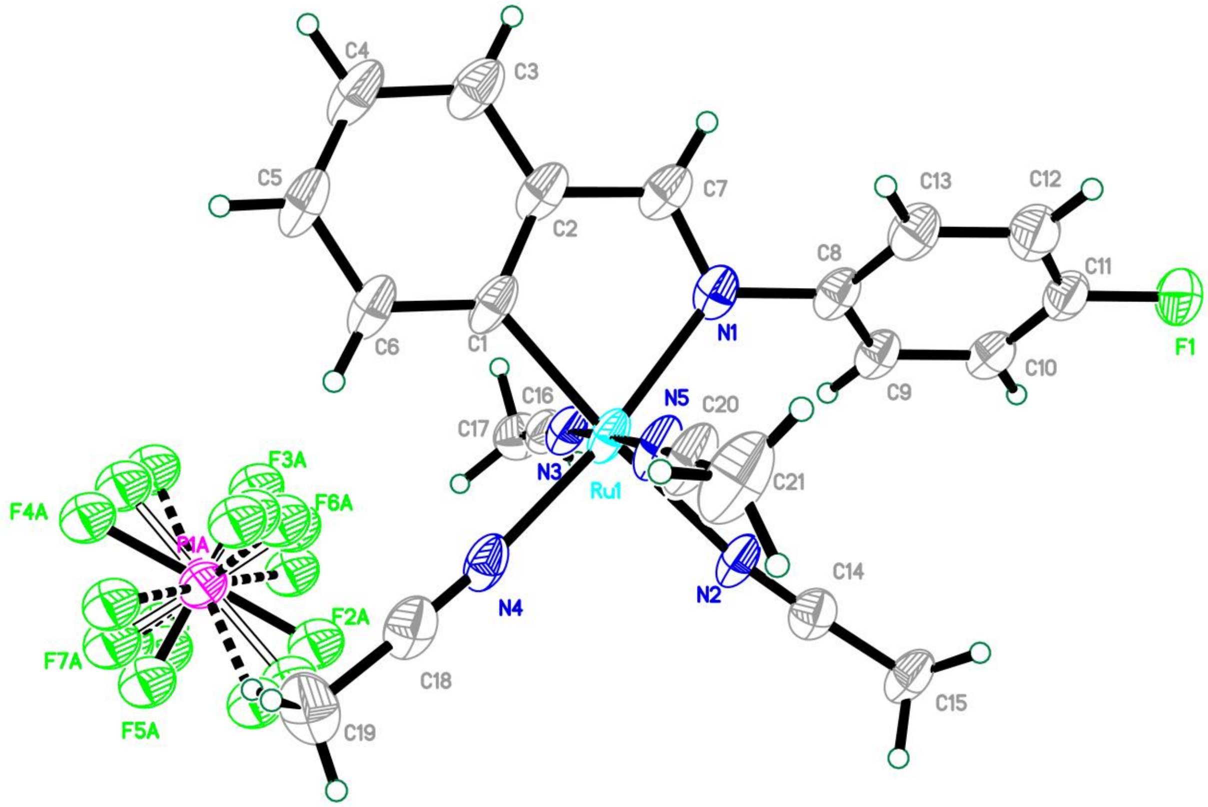

Figure S33. Crystal structure of **2b**.

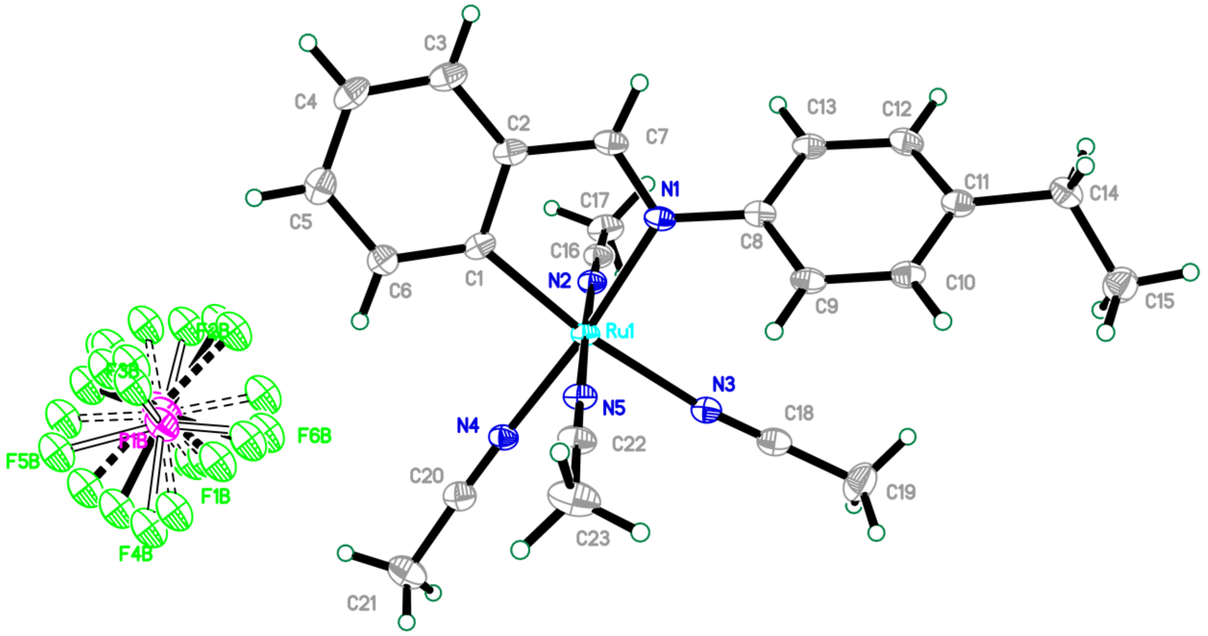

Figure S34. Crystal structure of **2d**.

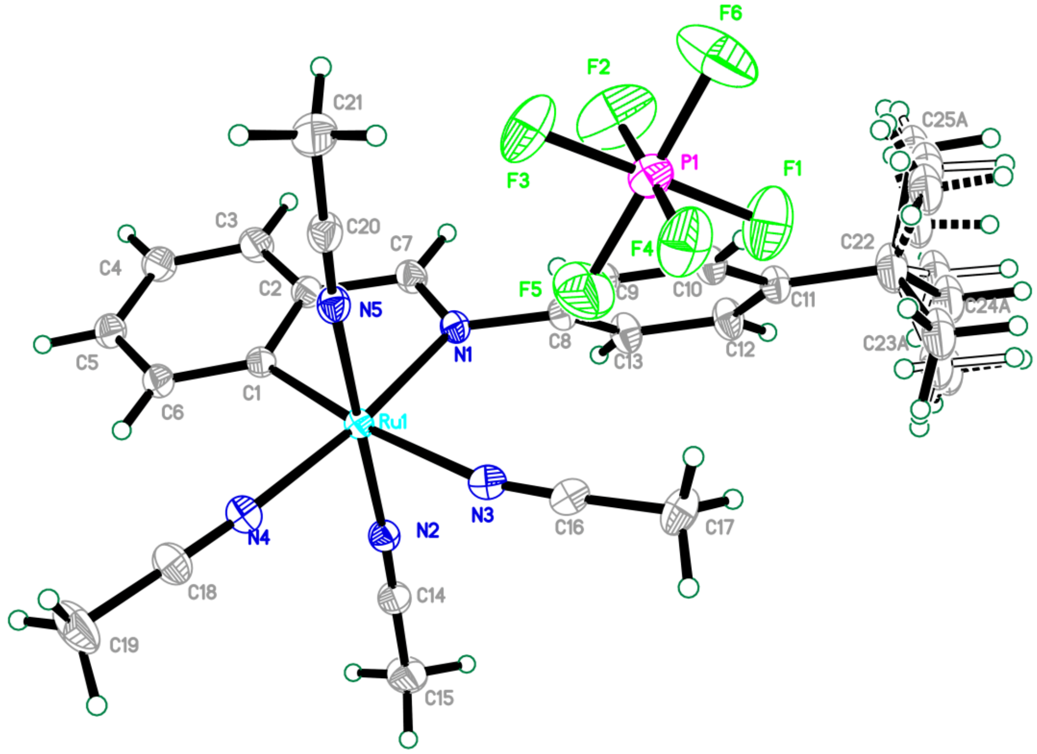

Figure S35. Crystal structure of **2e**.

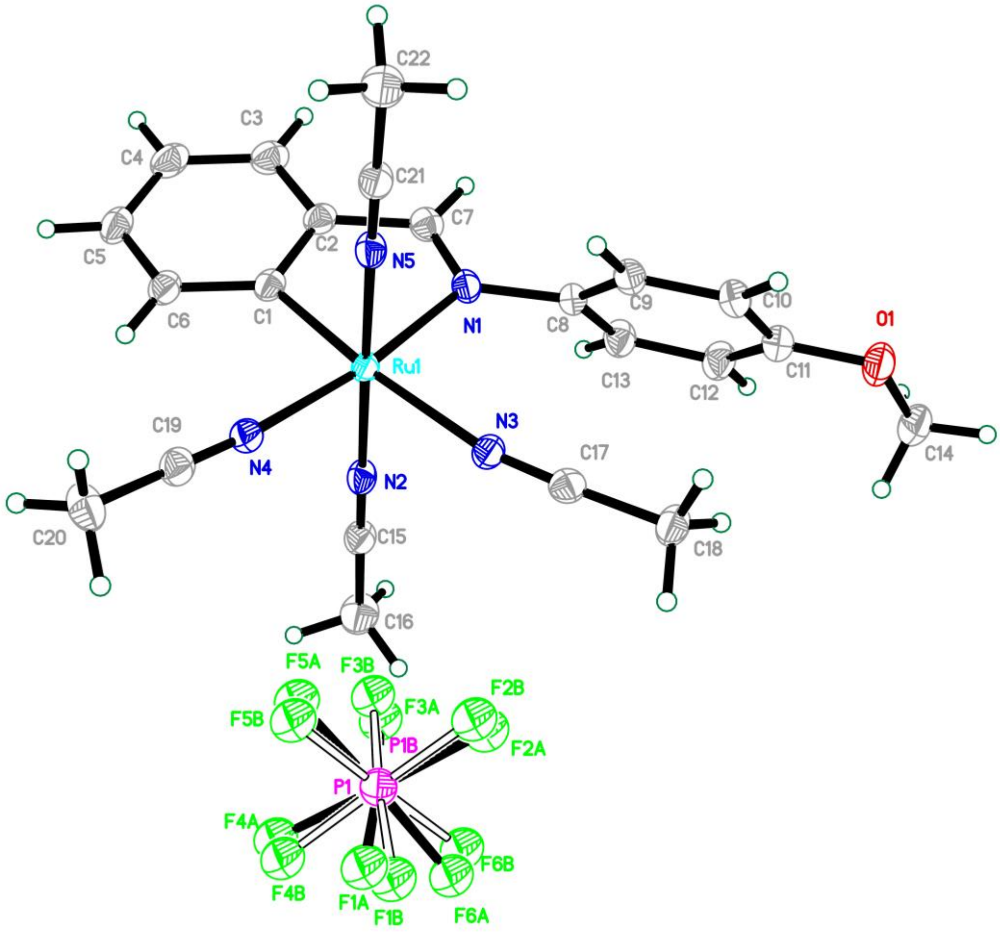

Figure S36. Crystal structure of **2f**.

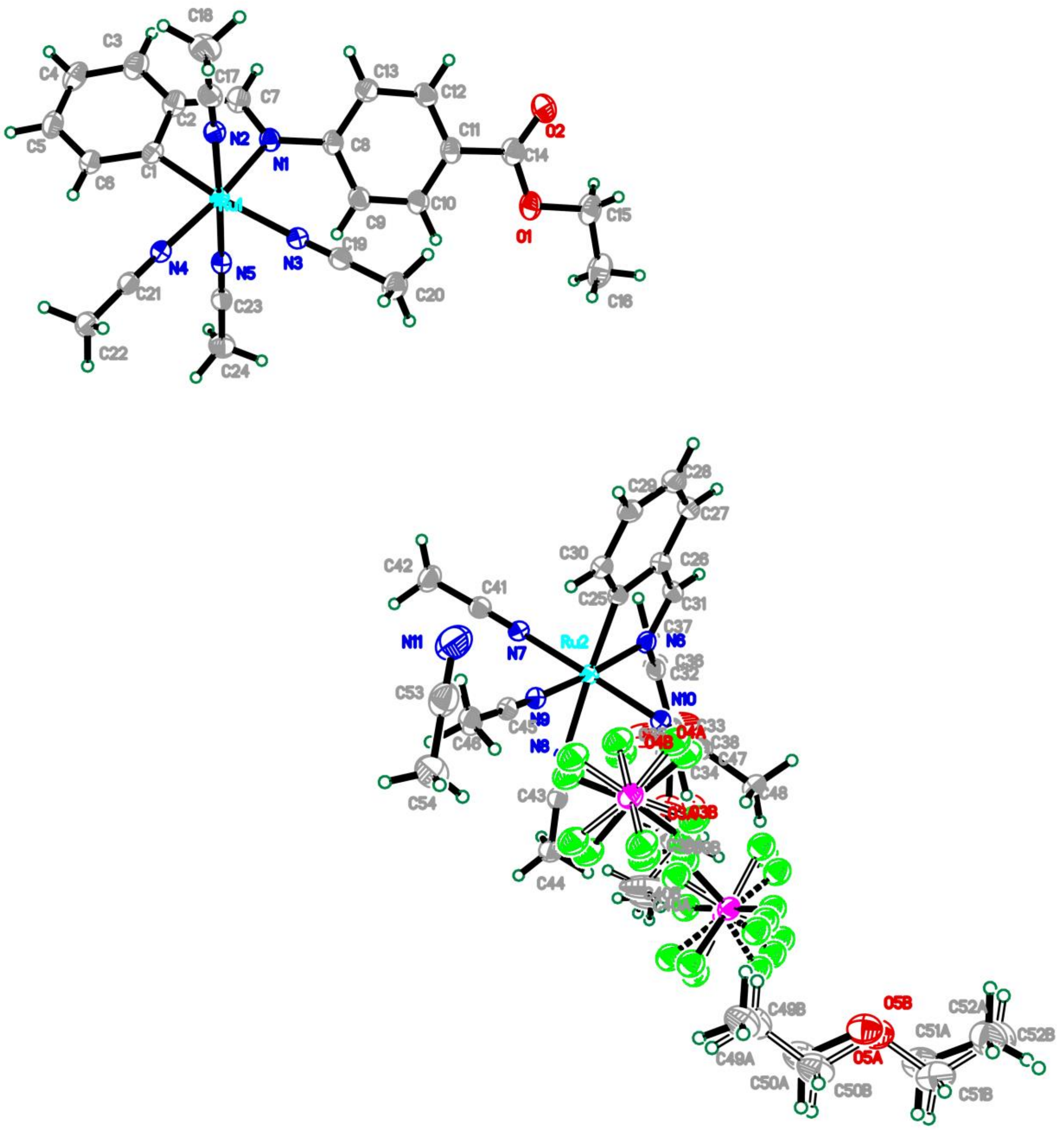

Figure S37. Crystal structure of **2g**.

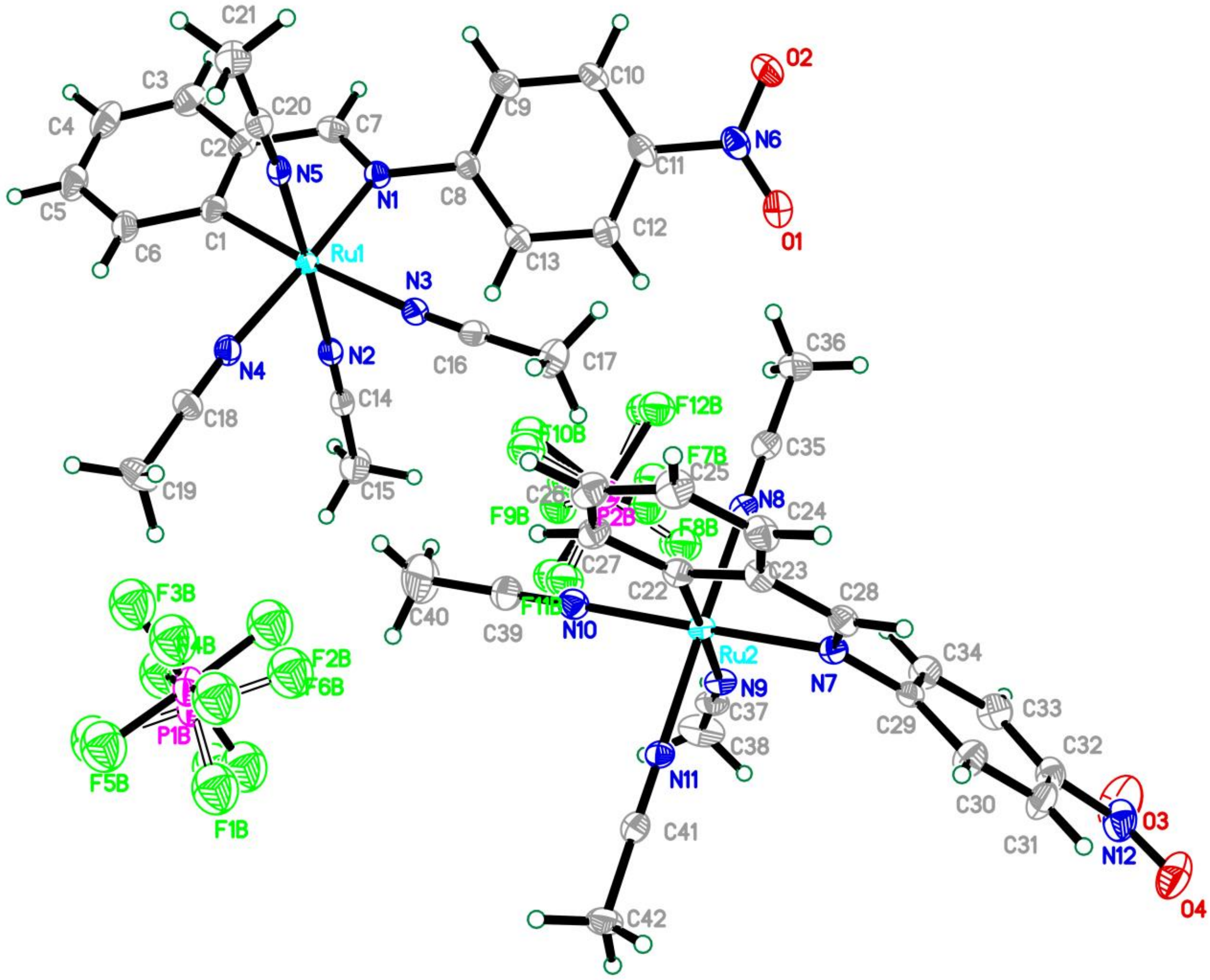

Figure S38. Crystal structure of **2h**.

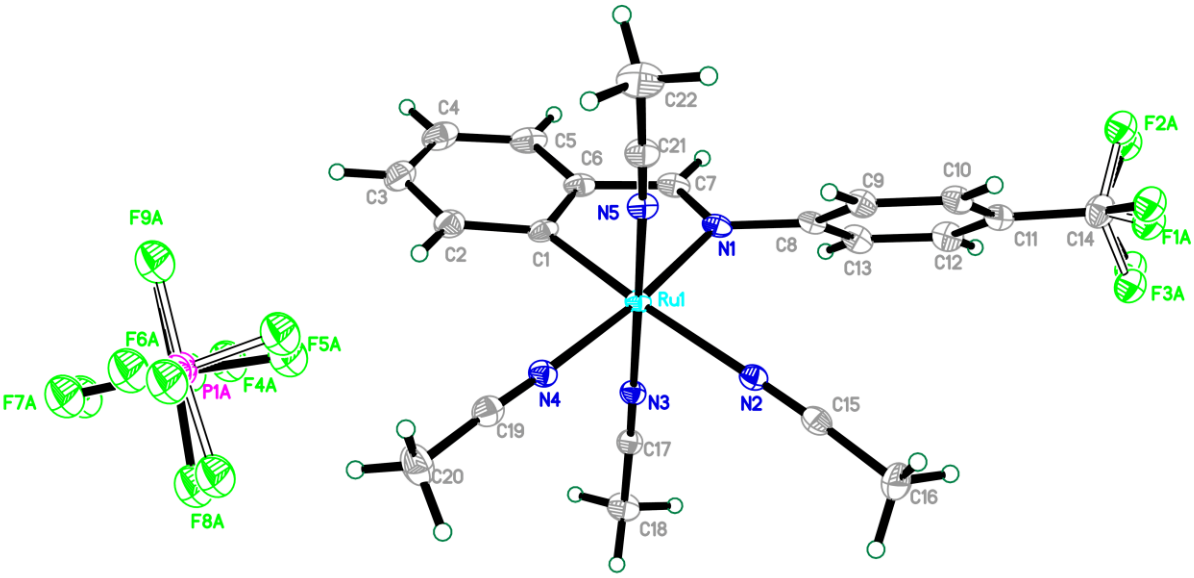

Figure S39. Crystal structure of **2i**.

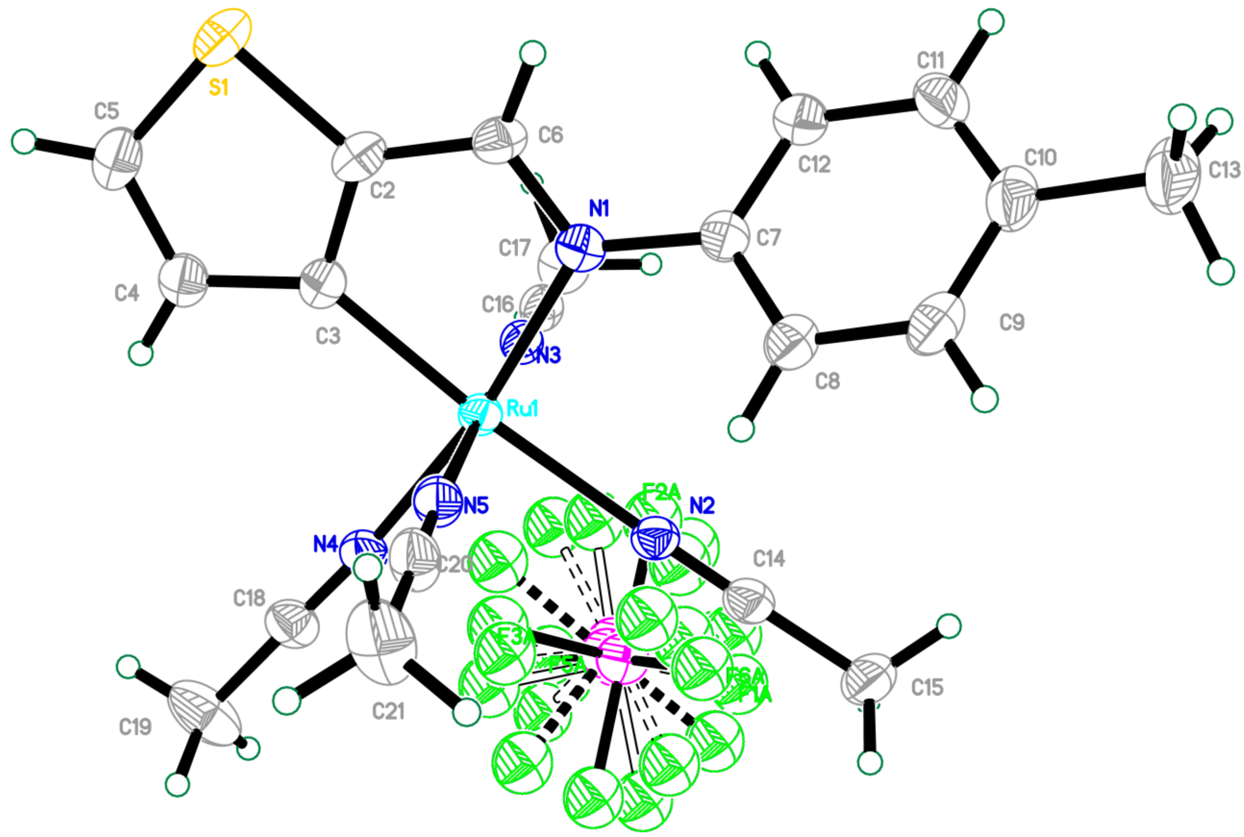

Figure S40. Crystal structure of **2k**.

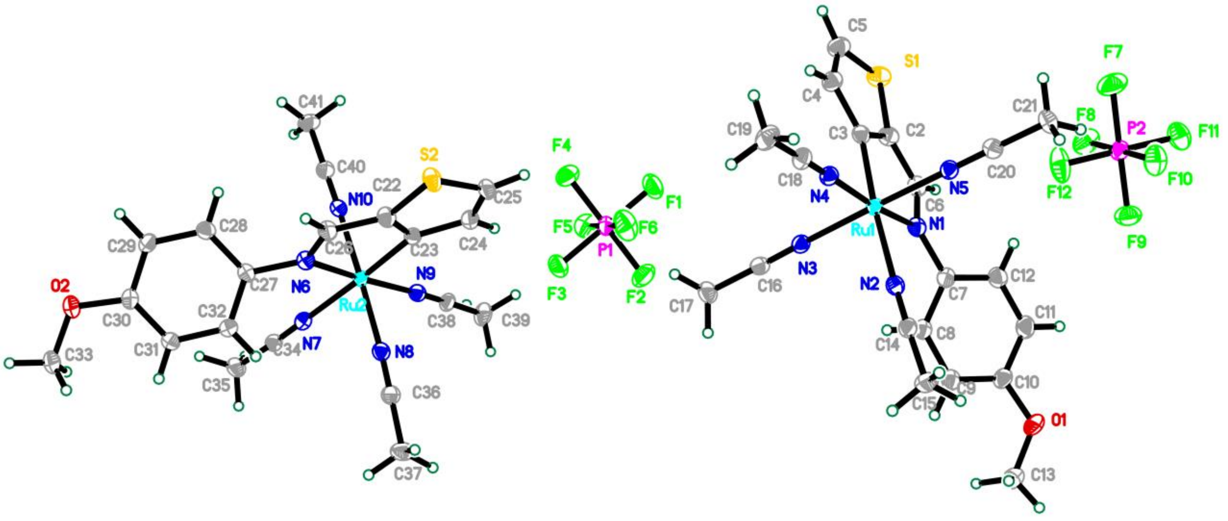

Figure S41. Crystal structure of **2l**.

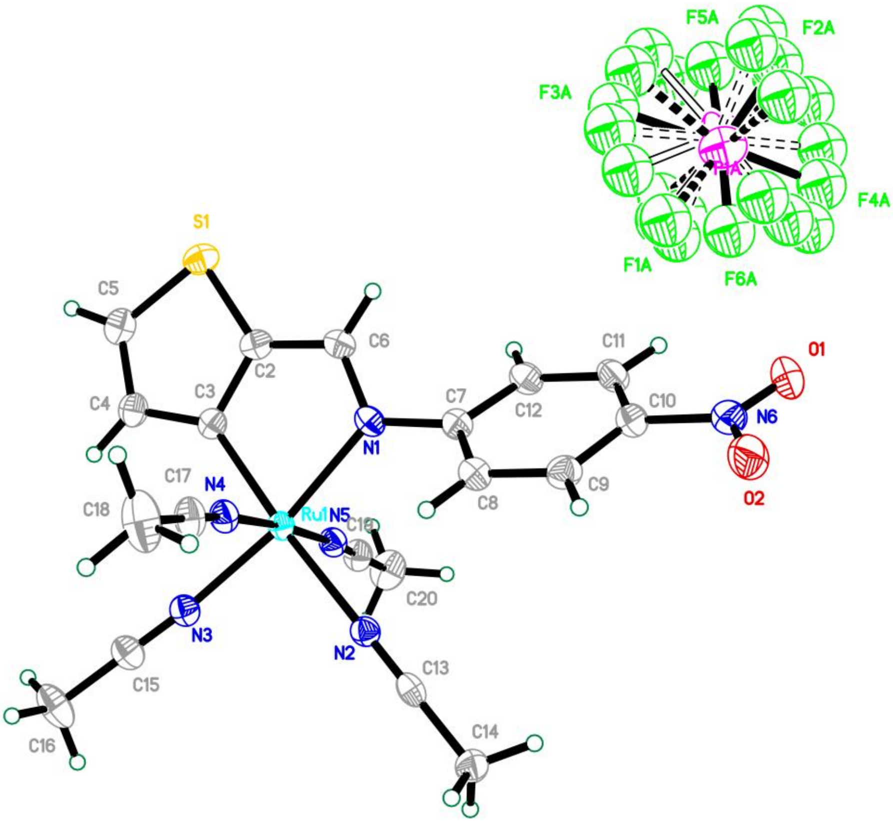

Figure S42. Crystal structure of **2m**.

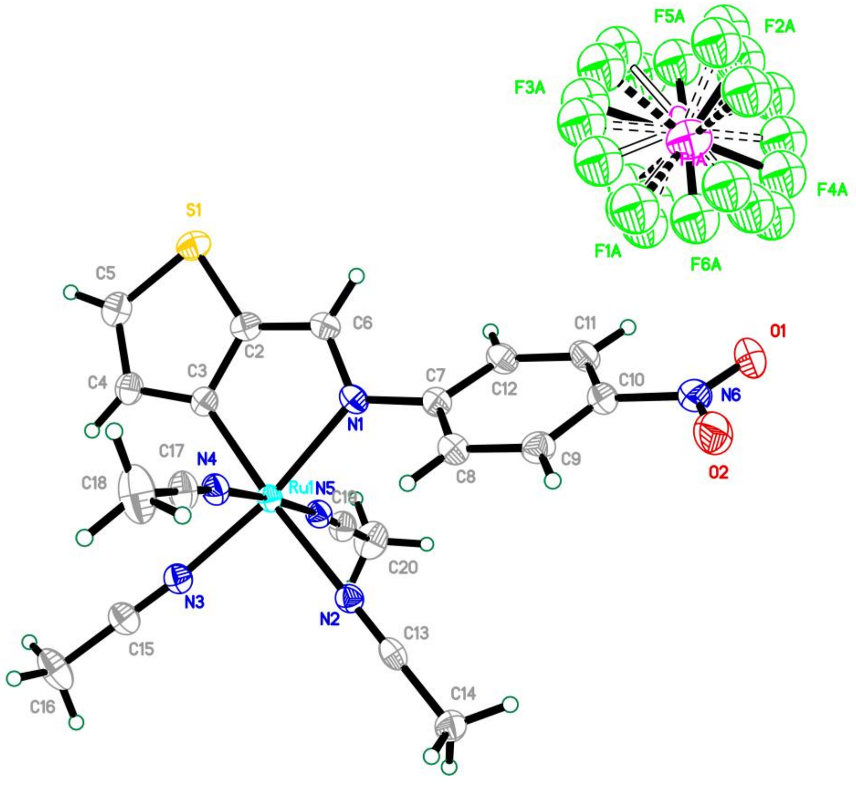

Figure S43. Crystal structure of **2n**.

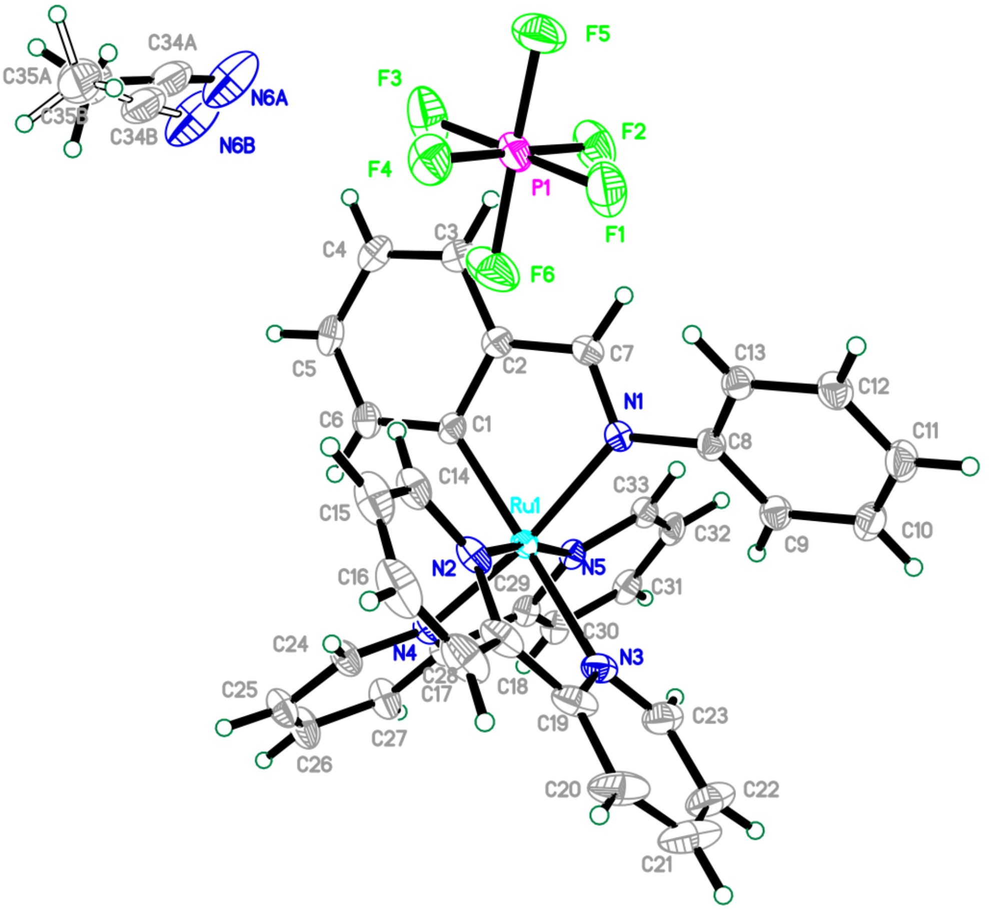

Figure S44. Crystal structure of **3a**.

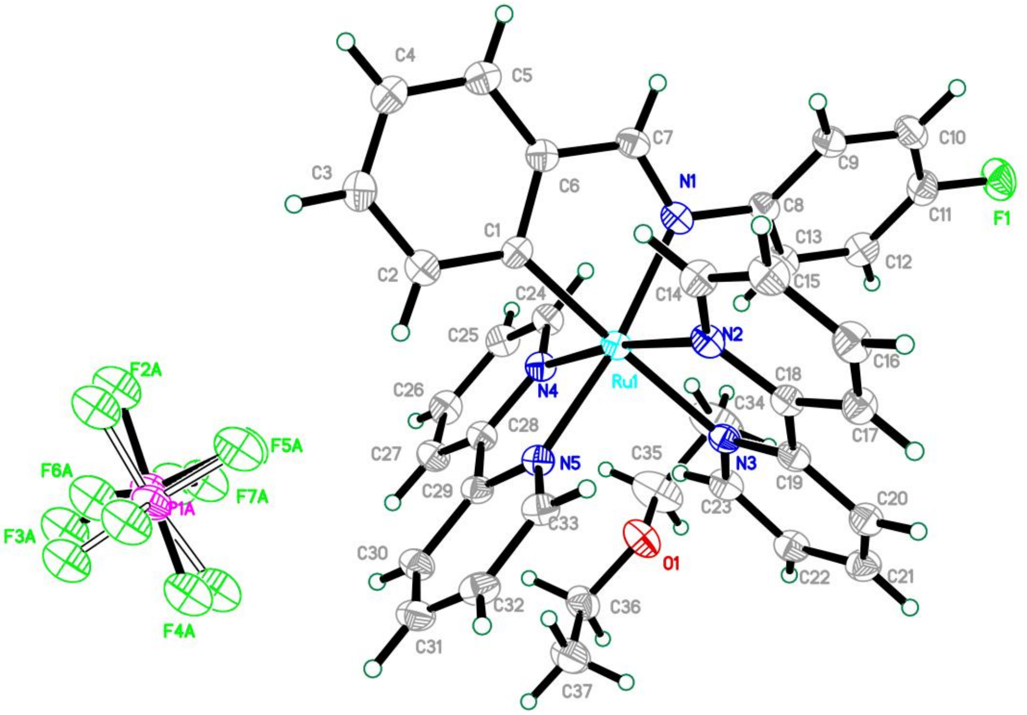

Figure S45. Crystal structure of **3b**.

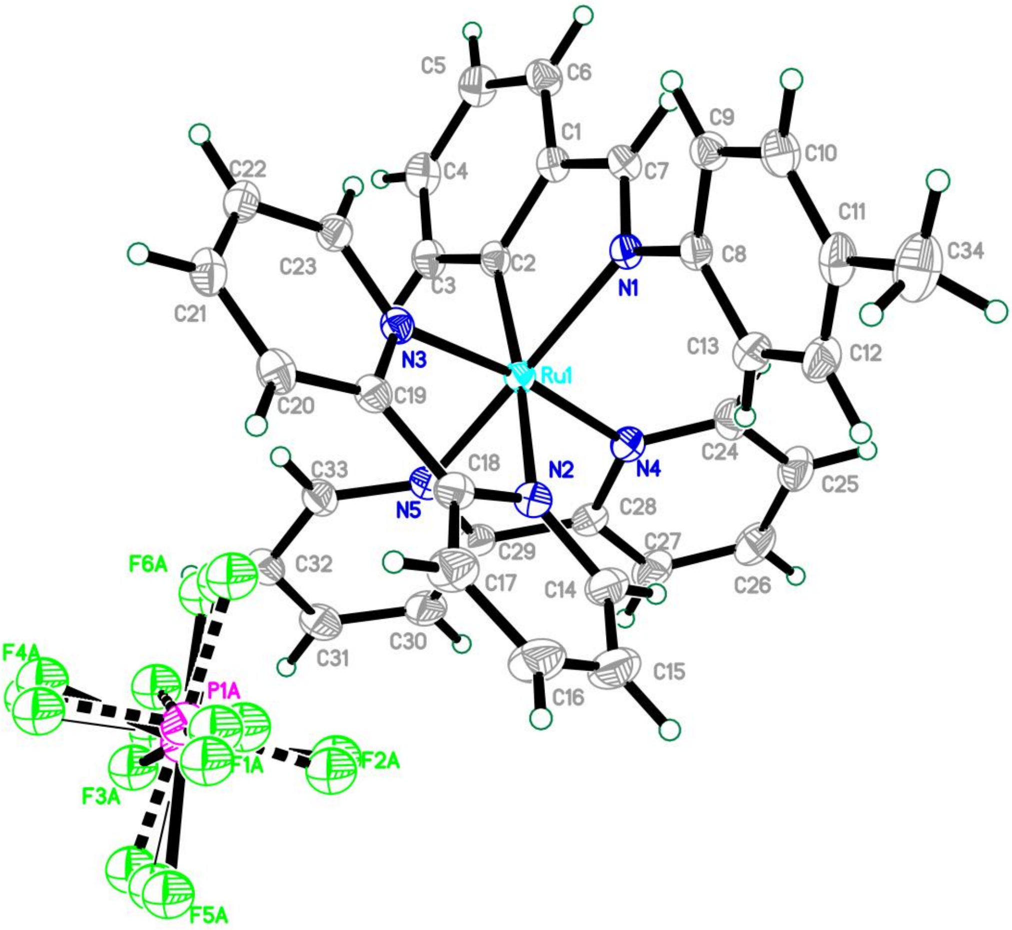

Figure S46. Crystal structure of **3c**.

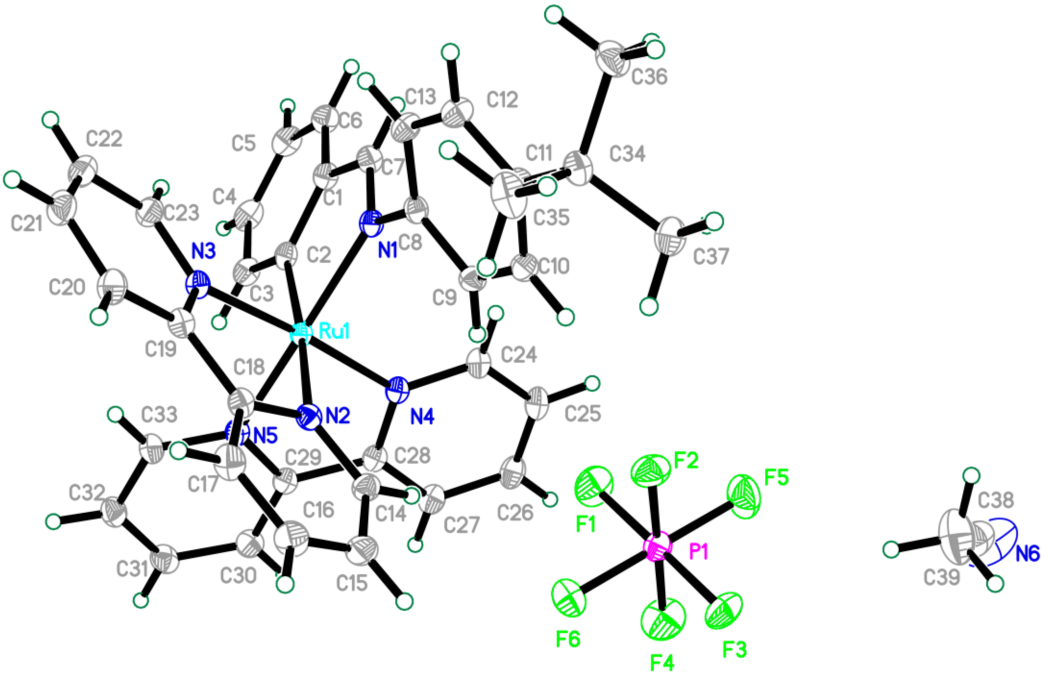

Figure S47. Crystal structure of **3e**.

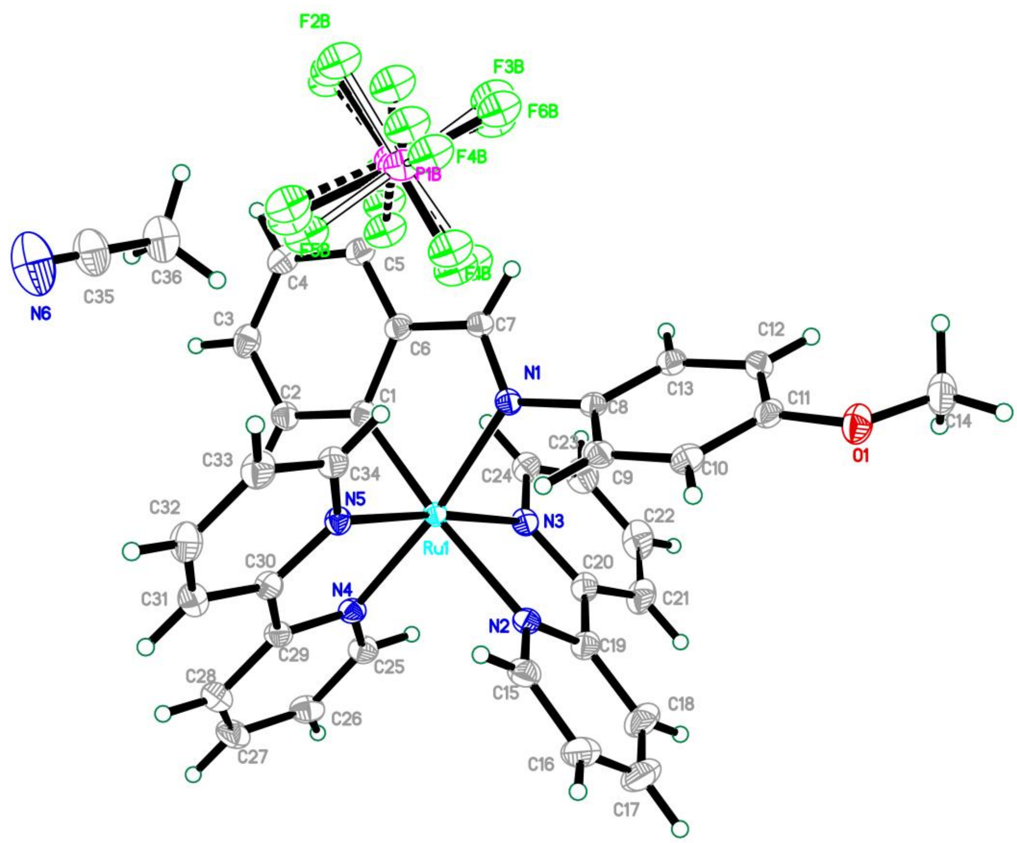

Figure S48. Crystal structure of **3f**.

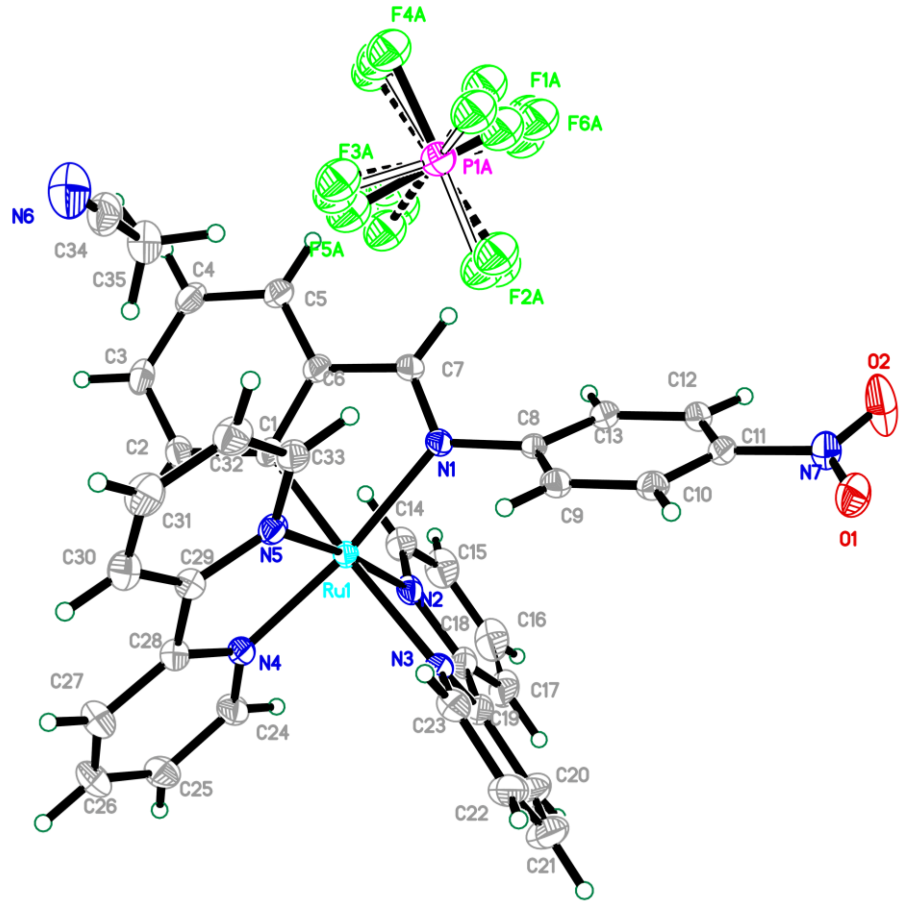

Figure S49. Crystal structure of **3h**.

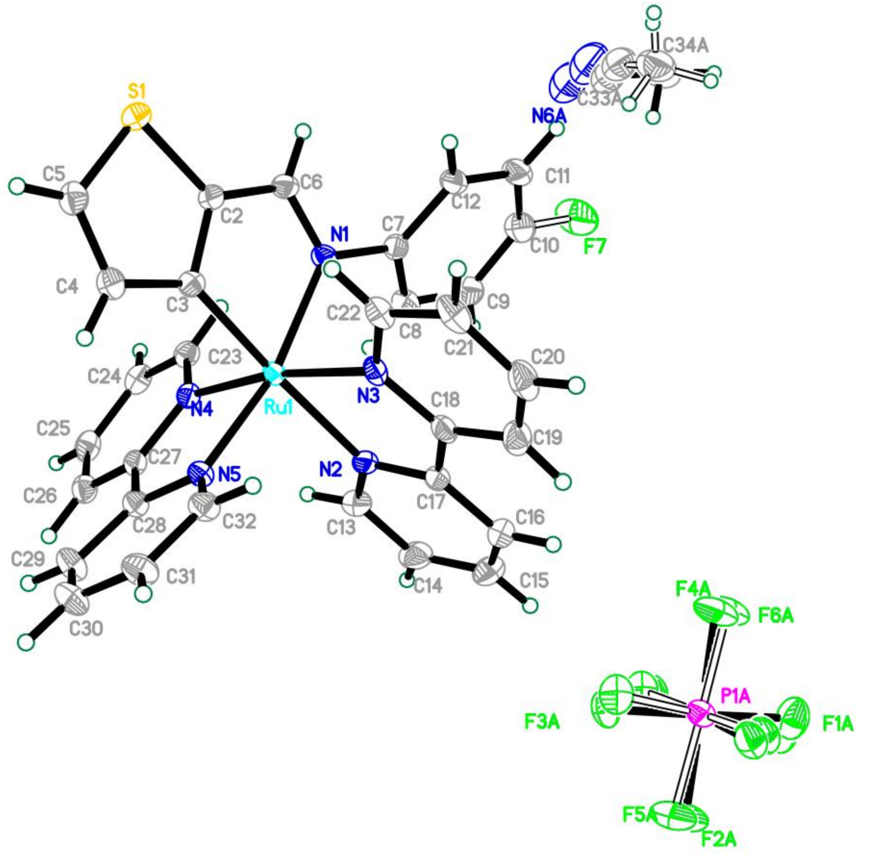

Figure S50. Crystal structure of **3j**.

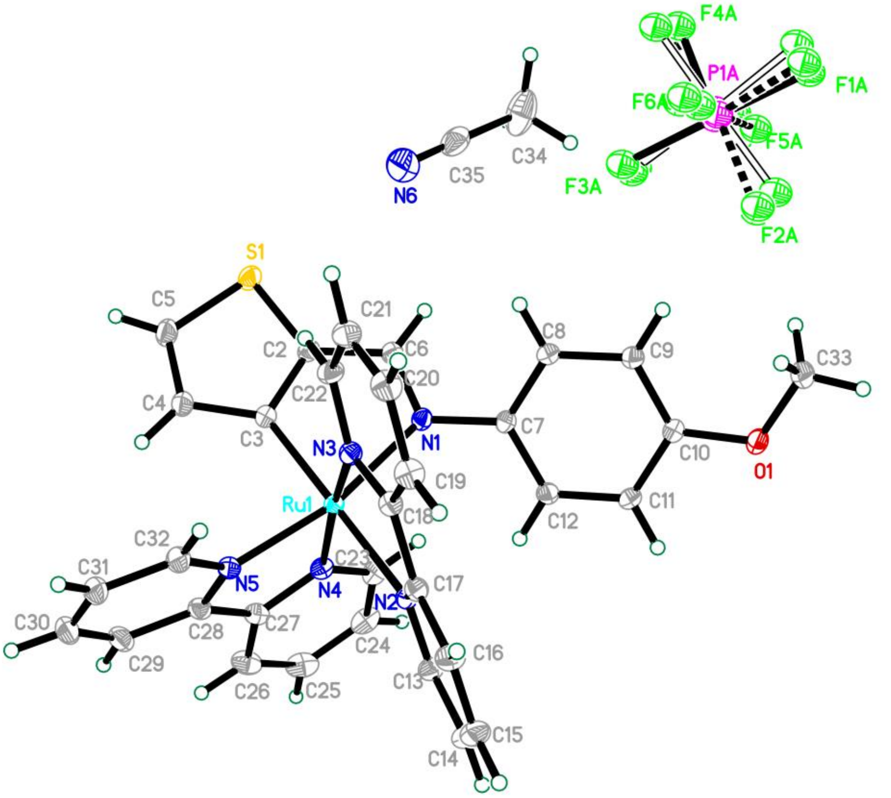

Figure S51. Crystal structure of **3l**.

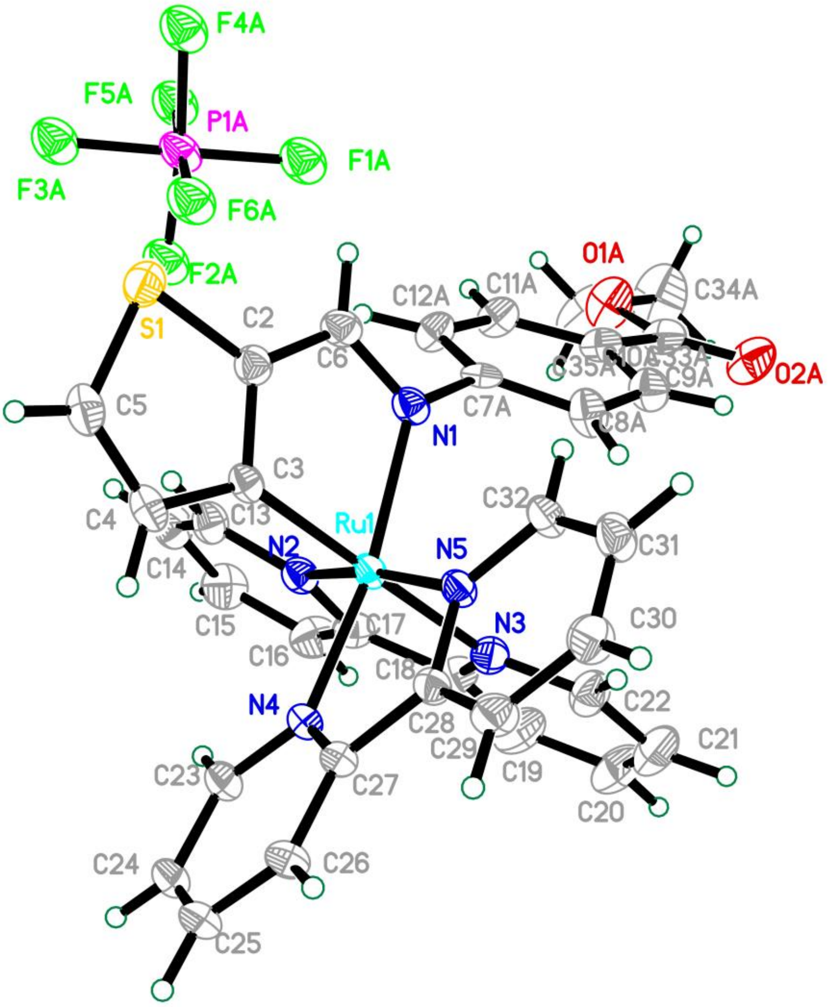

Figure S52. Crystal structure of **3m**.

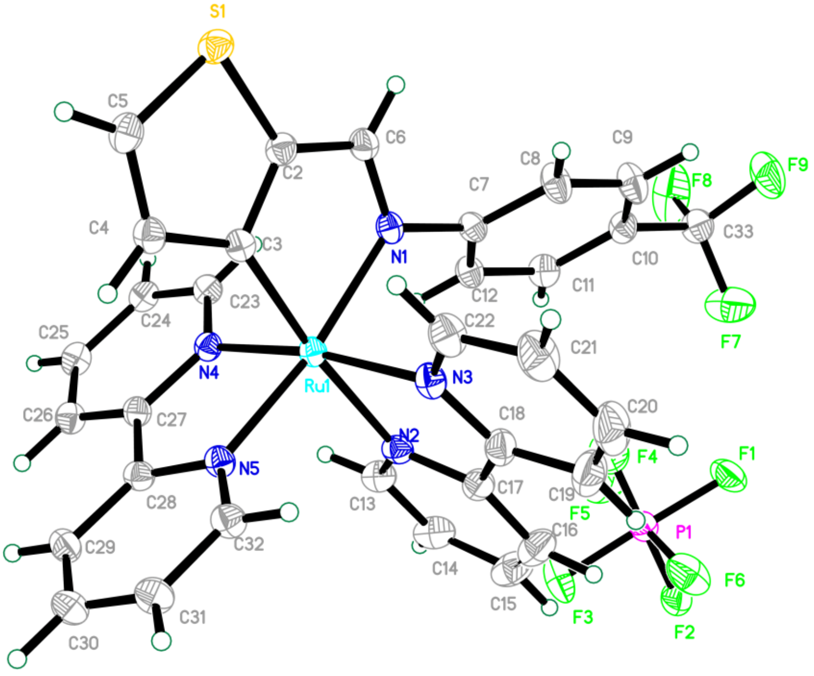

Figure S53. Crystal structure of **3o**.

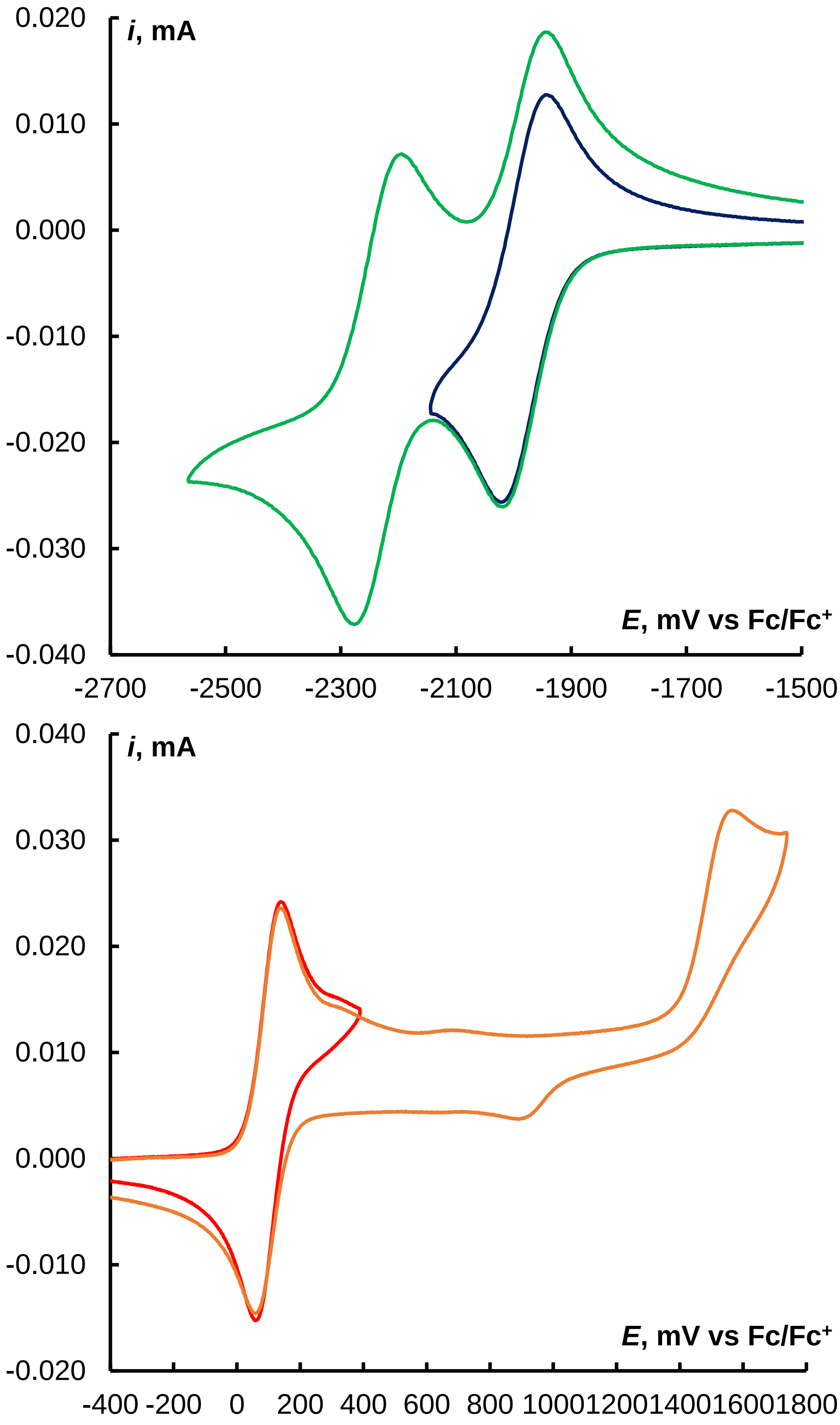

Figure S54. CV curves of **3a**.

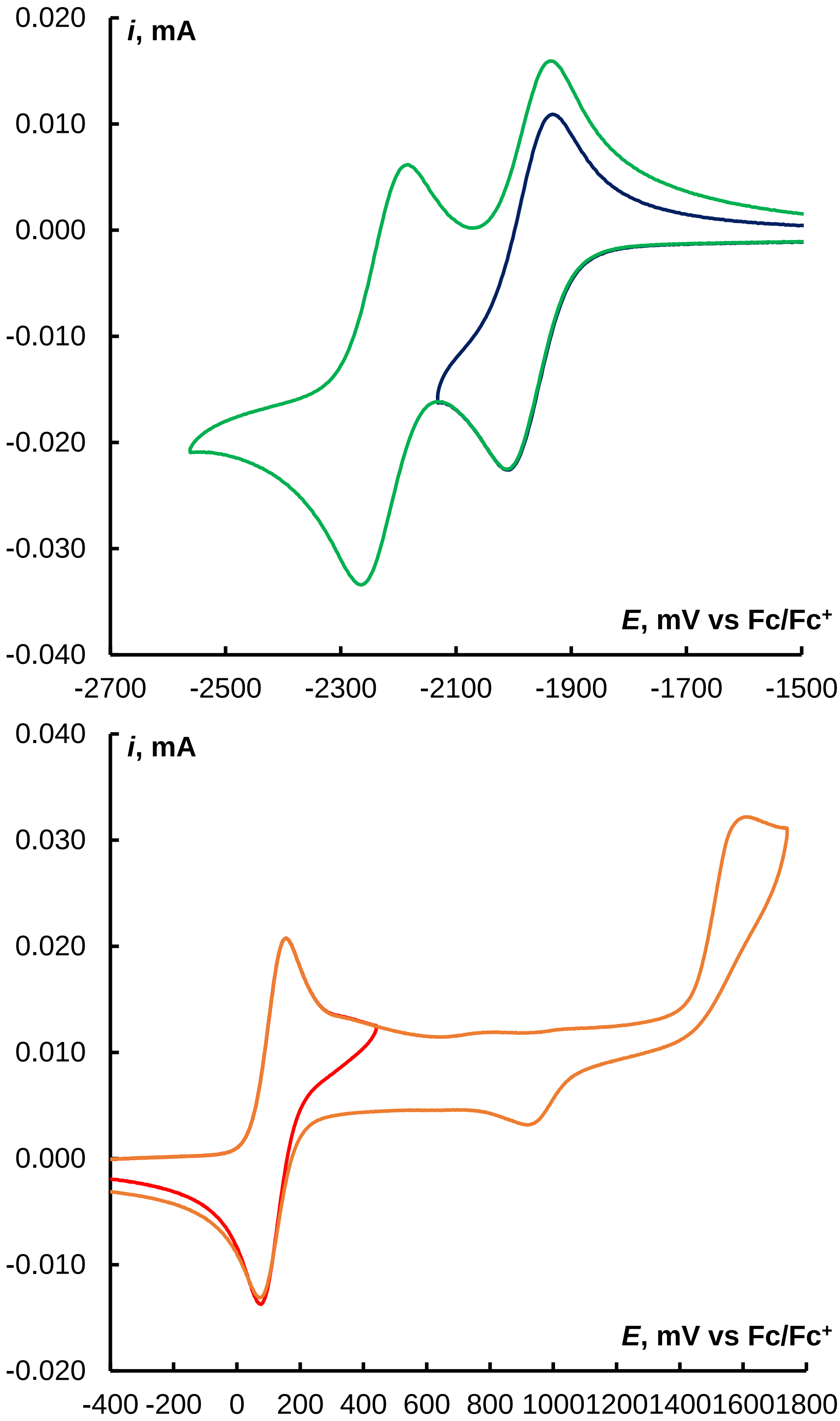

Figure S55. CV curves of **3b**.

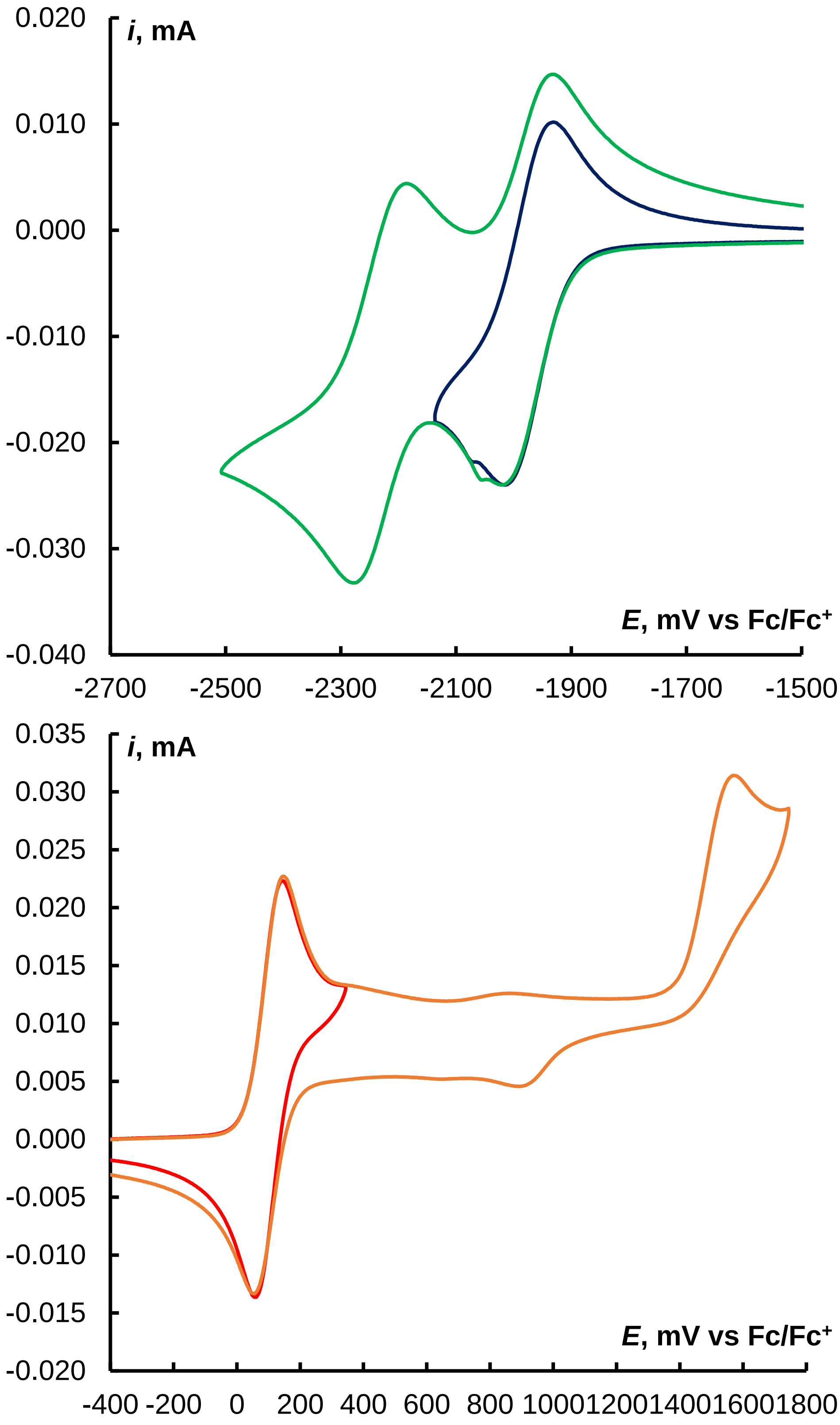

Figure S56. CV curves of **3c**.

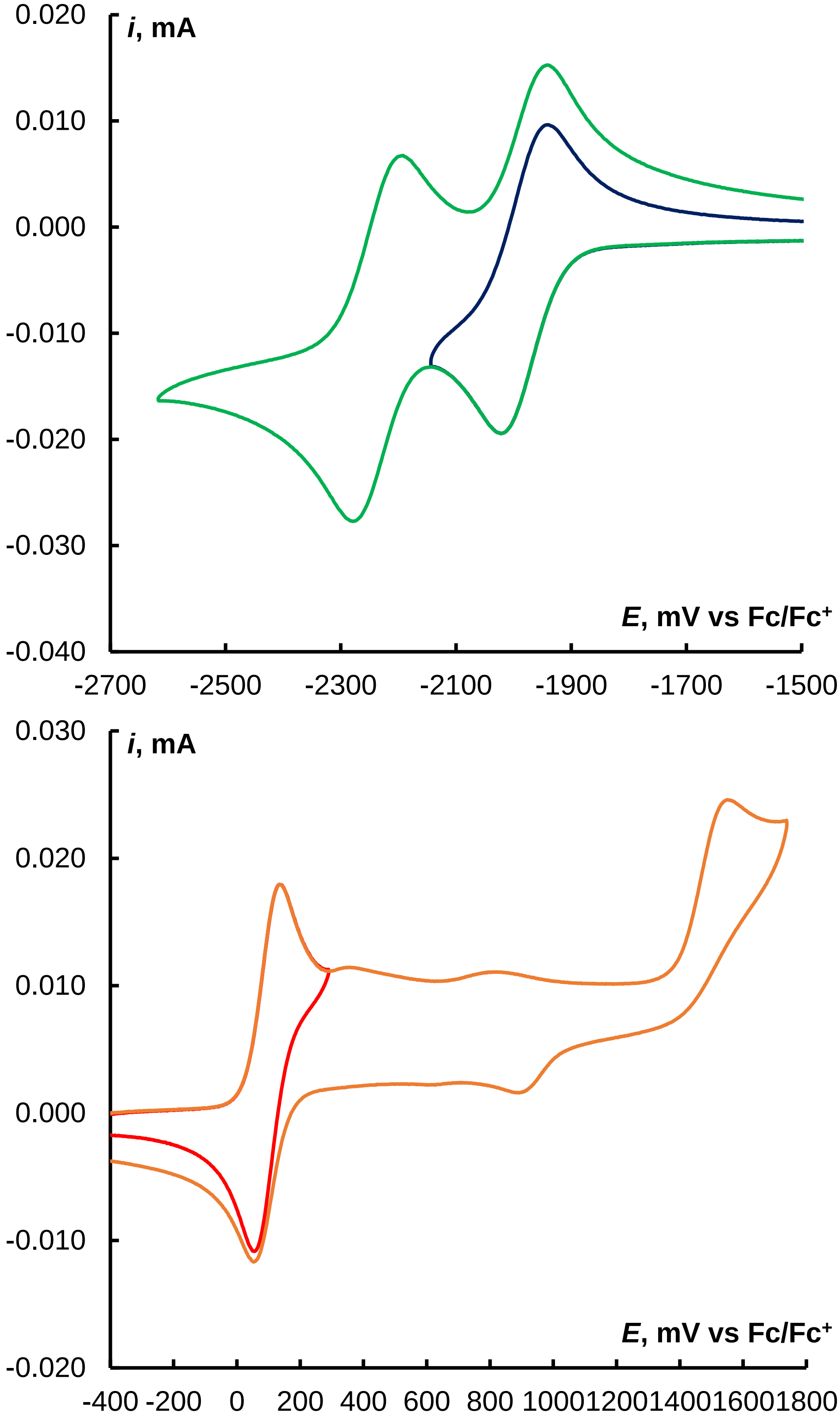

Figure S57. CV curves of **3d**.

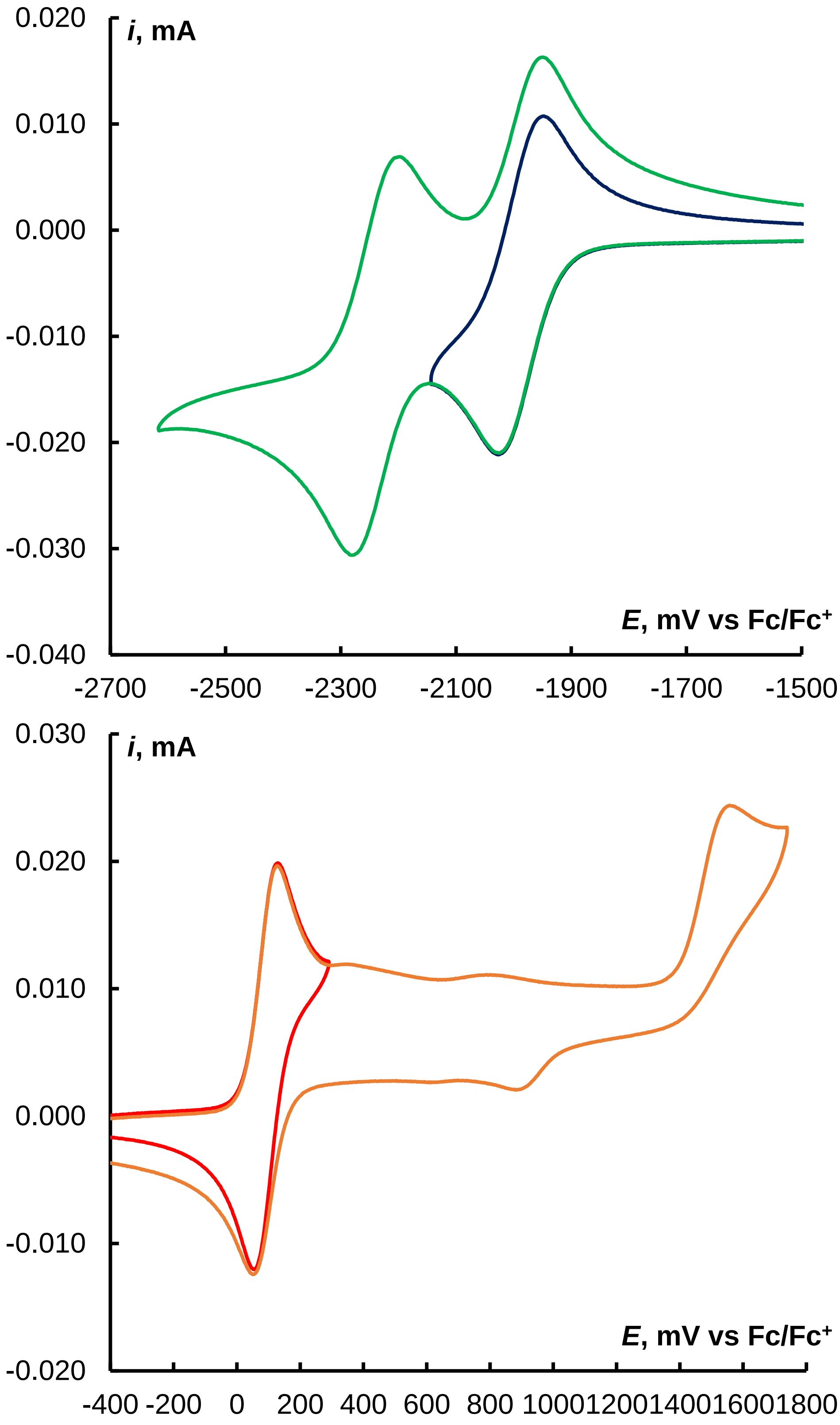

Figure S58. CV curves of **3e**.

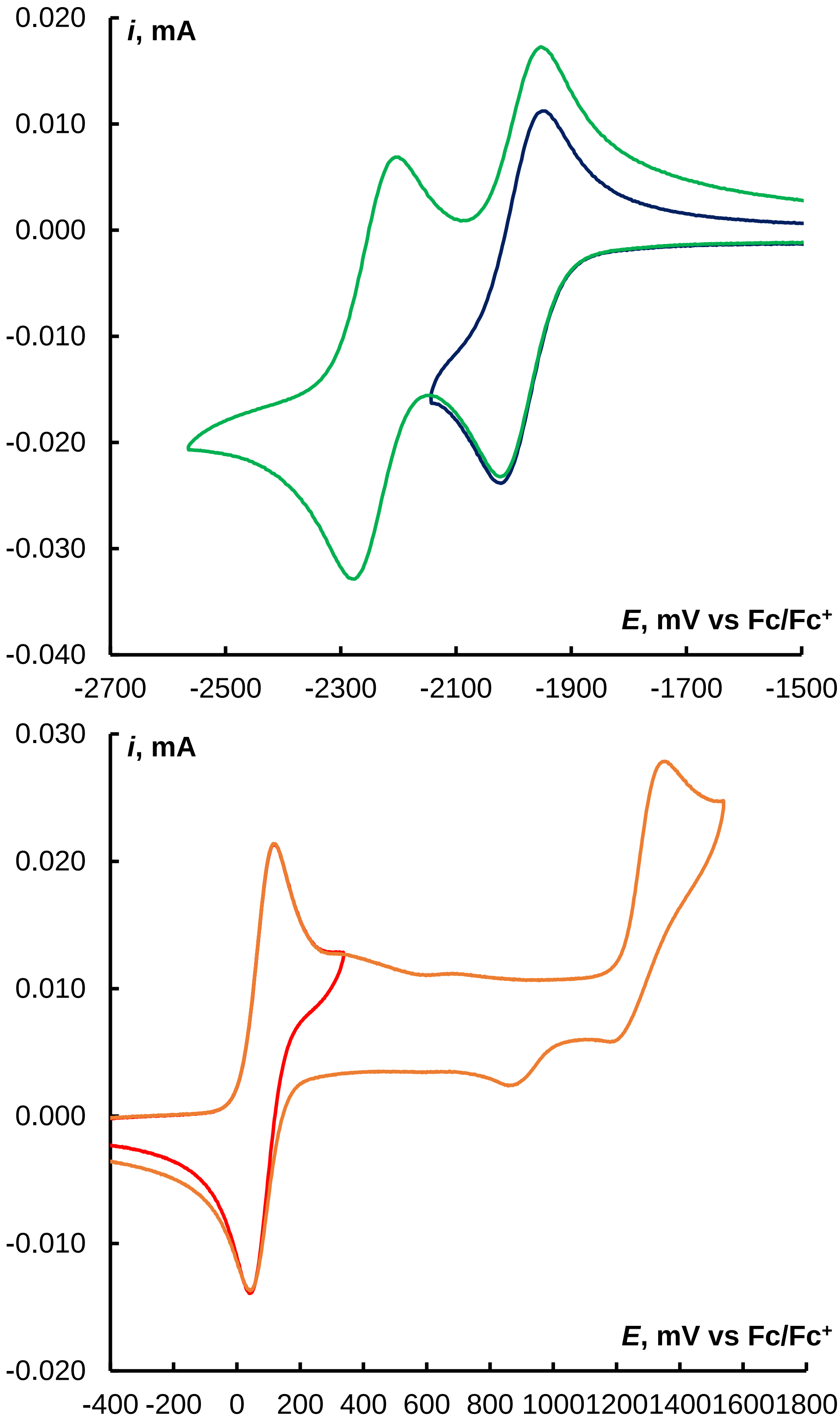

Figure S59. CV curves of **3f**.

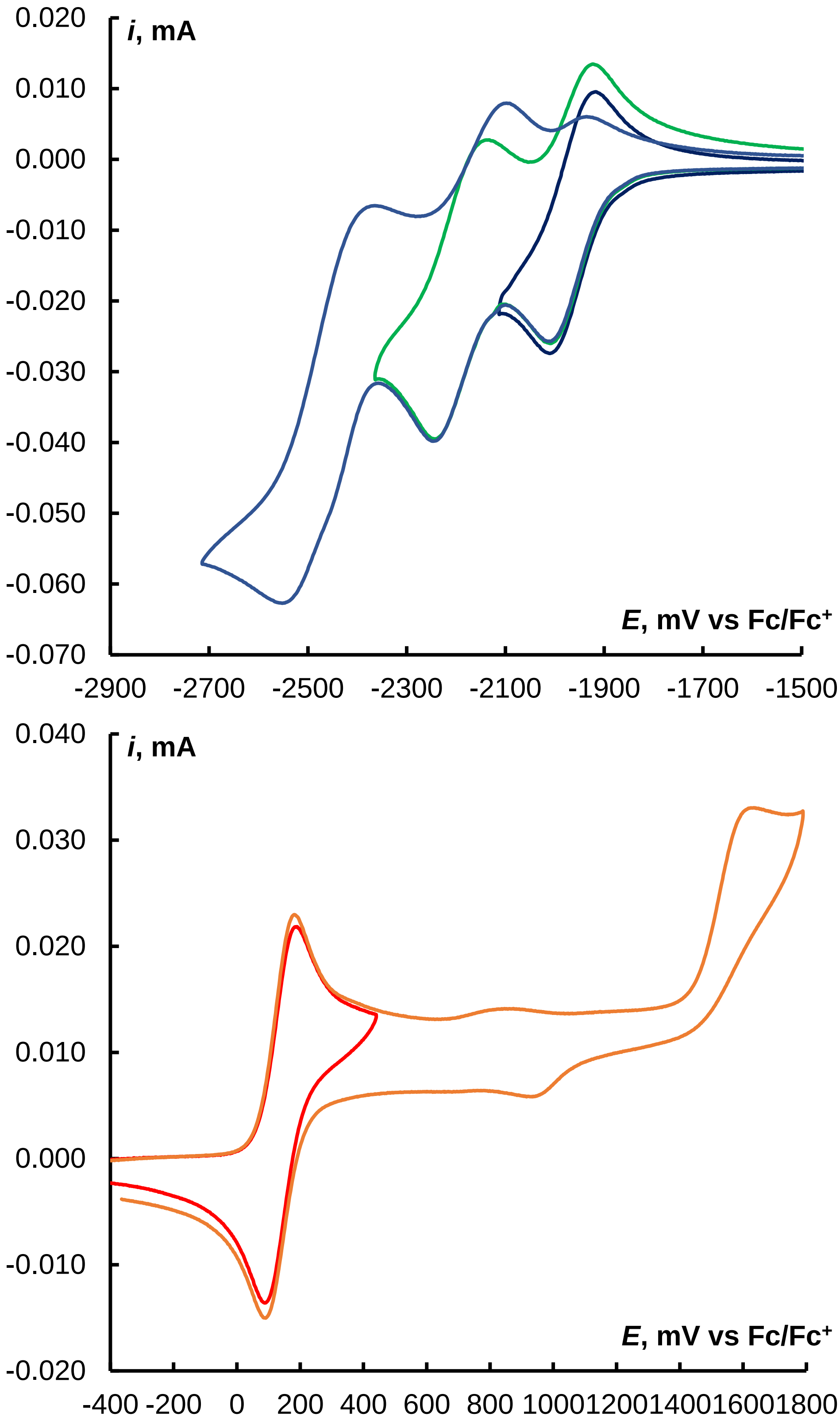

Figure S60. CV curves of **3g**.

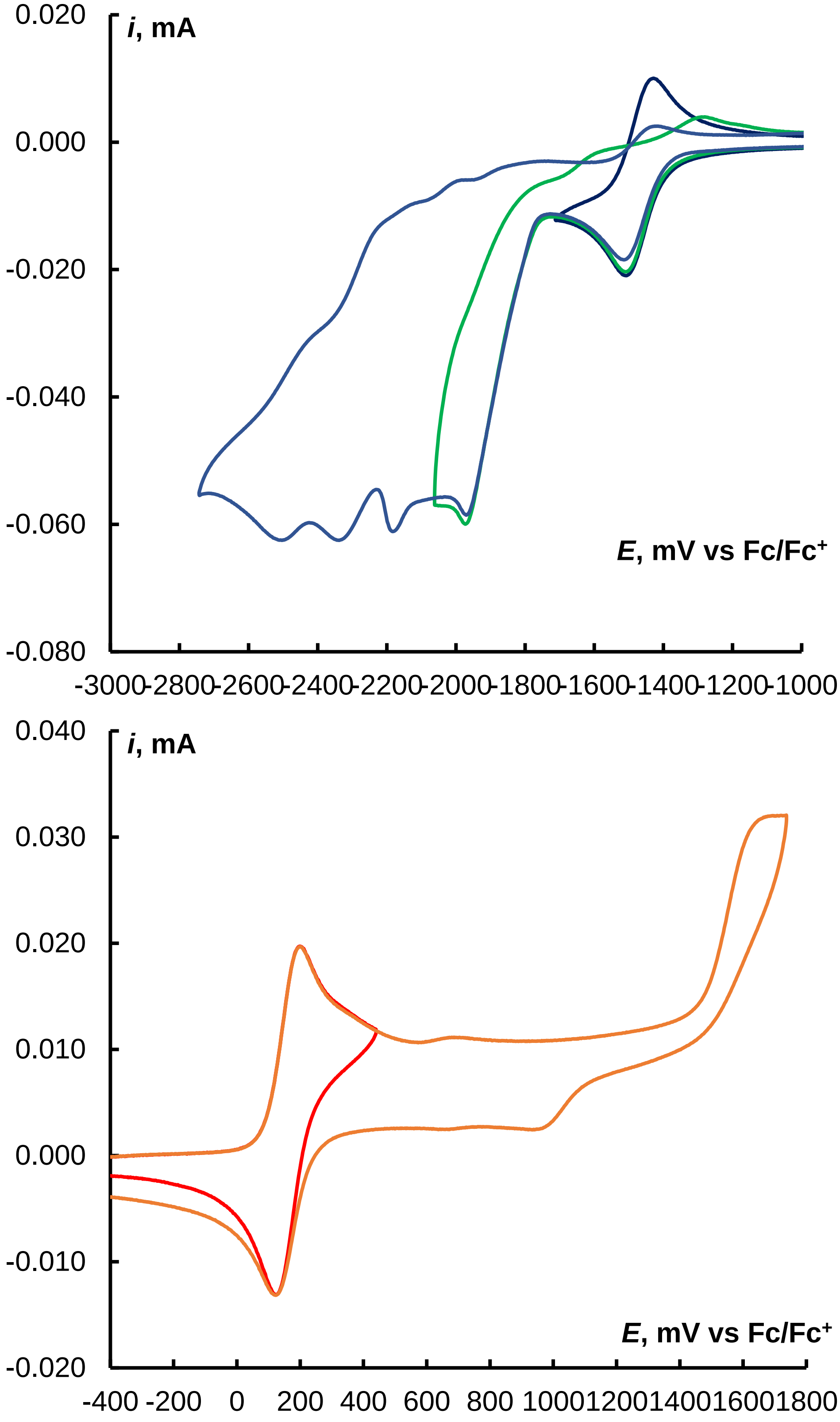

Figure S61. CV curves of **3h**.

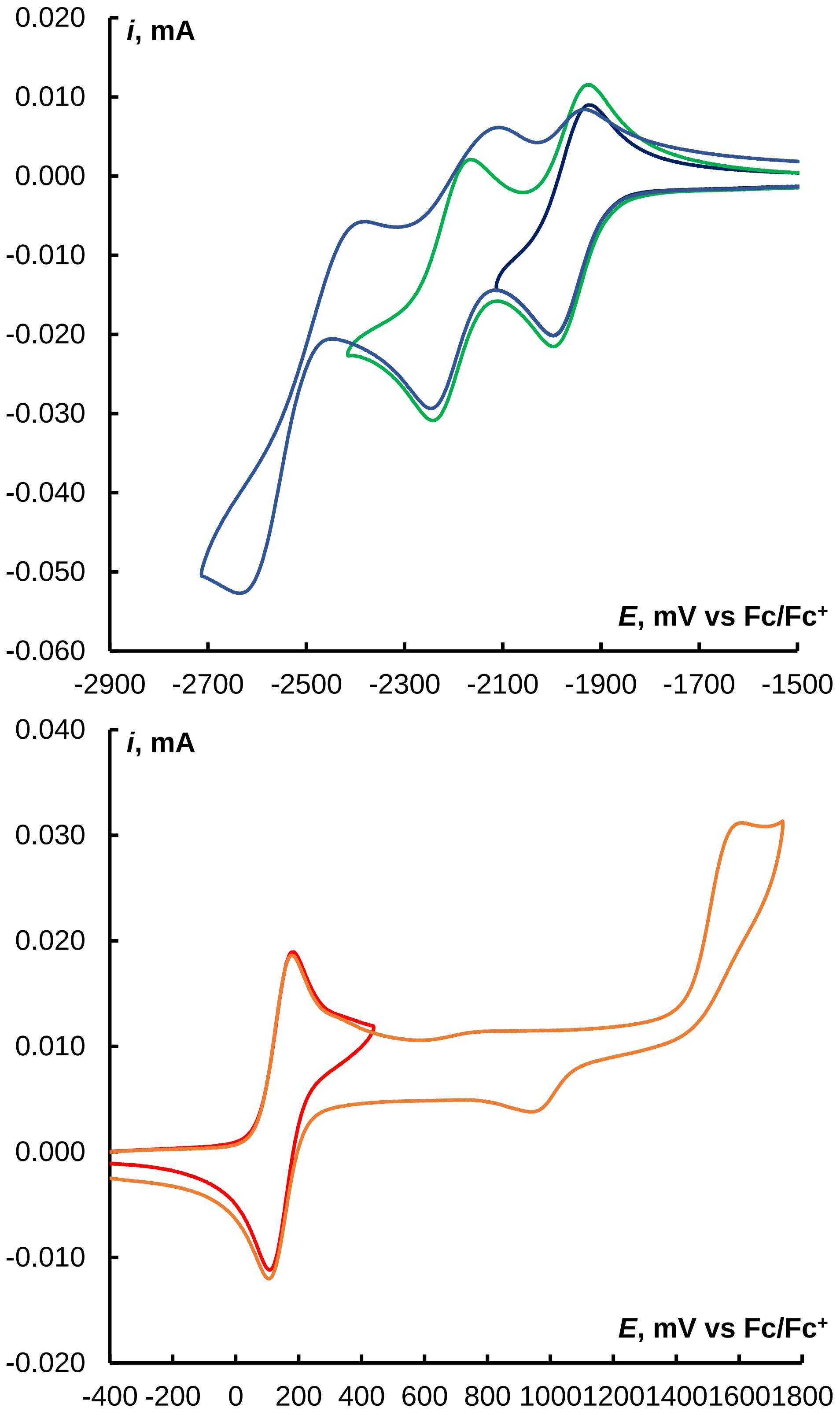

Figure S62. CV curves of **3i**.

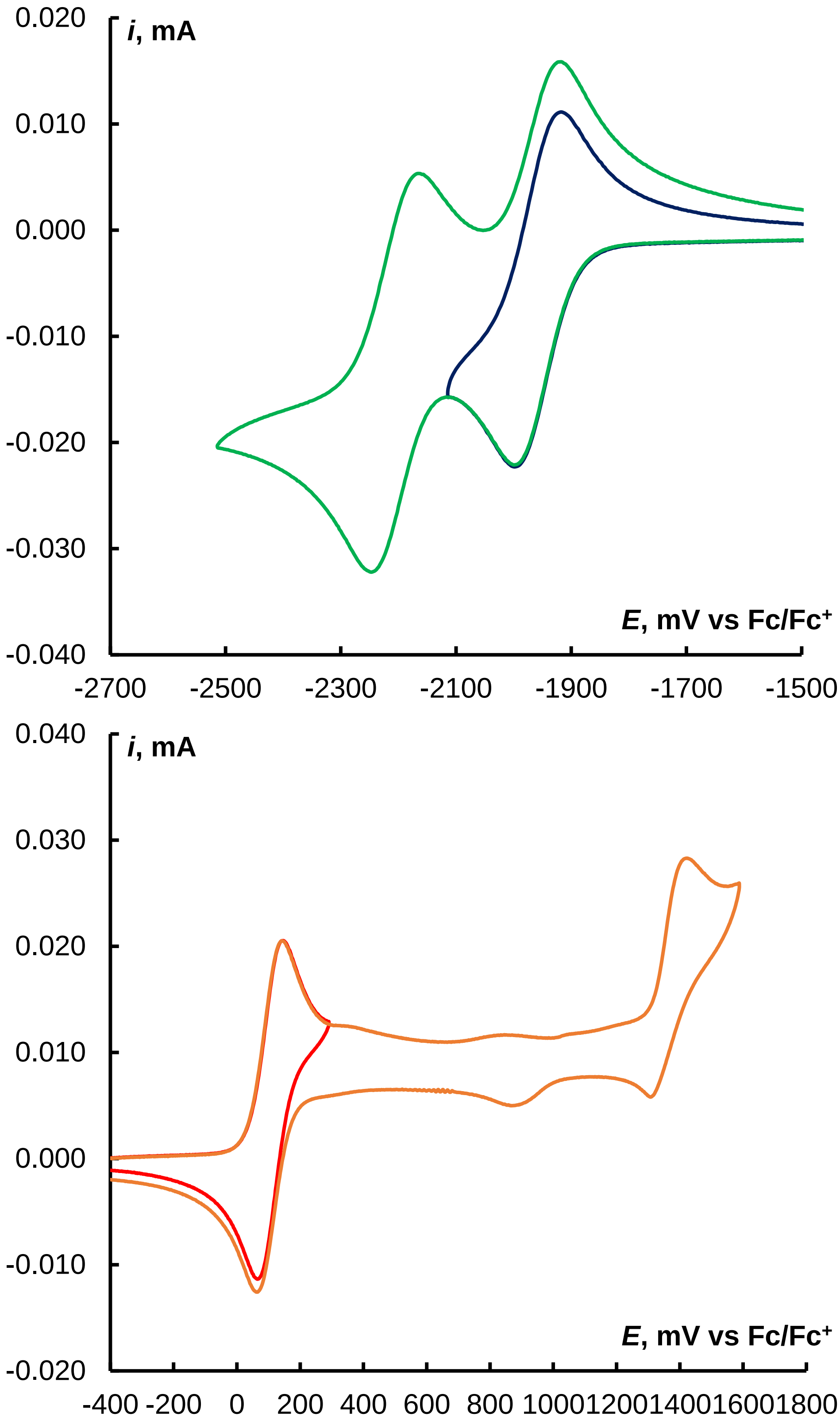

Figure S63. CV curves of **3j**.

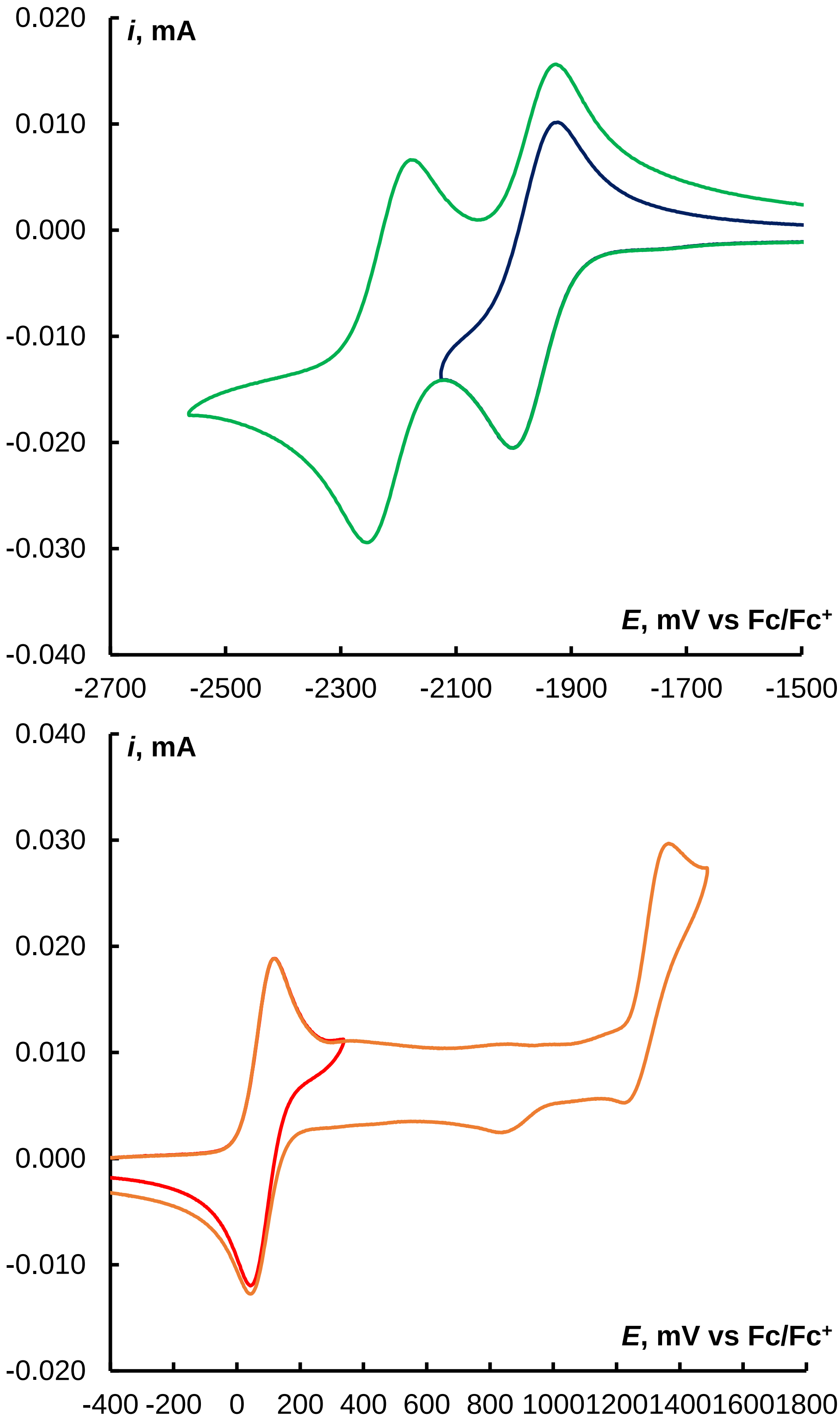

Figure S64. CV curves of **3k**.

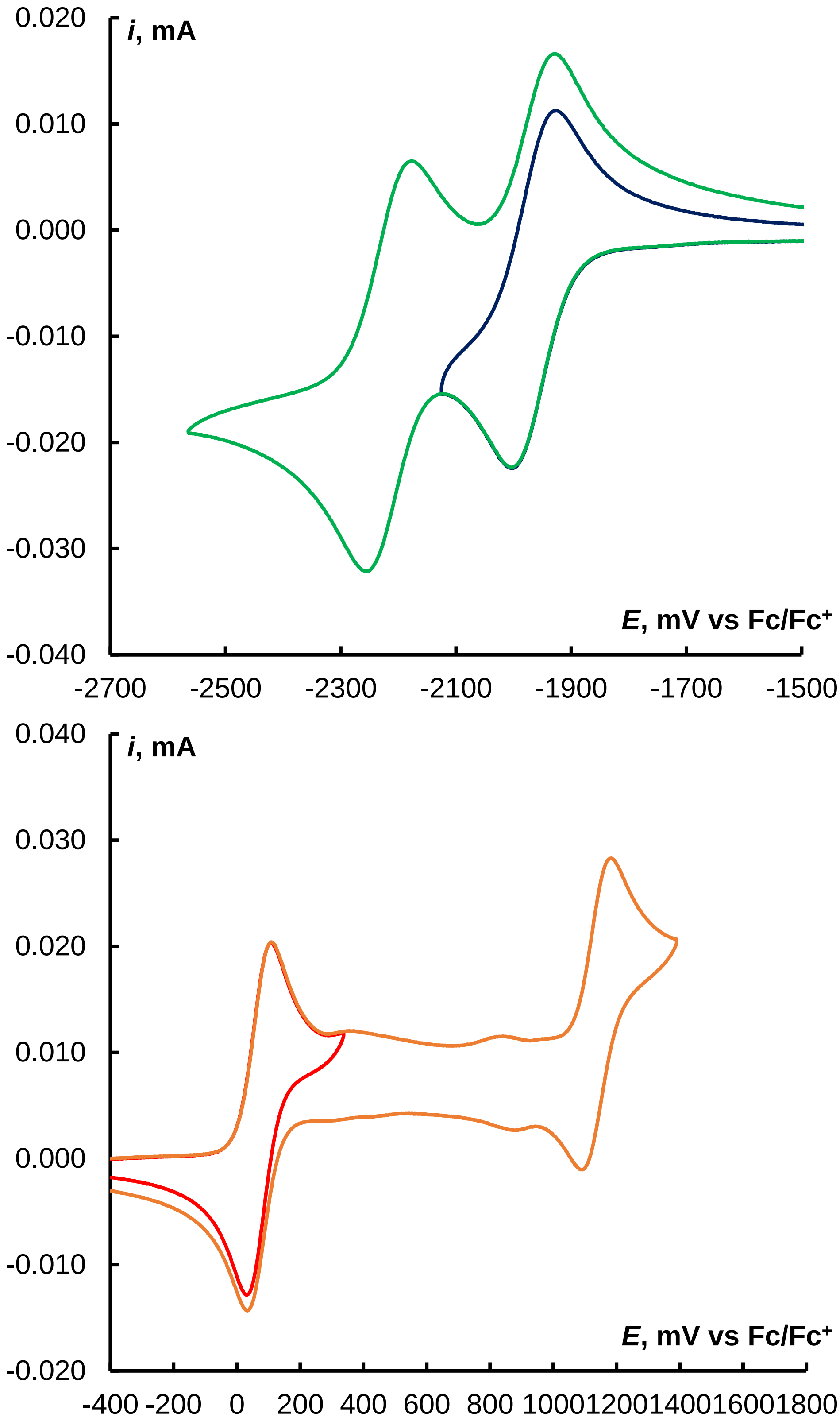

Figure S65. CV curves of **3l**.

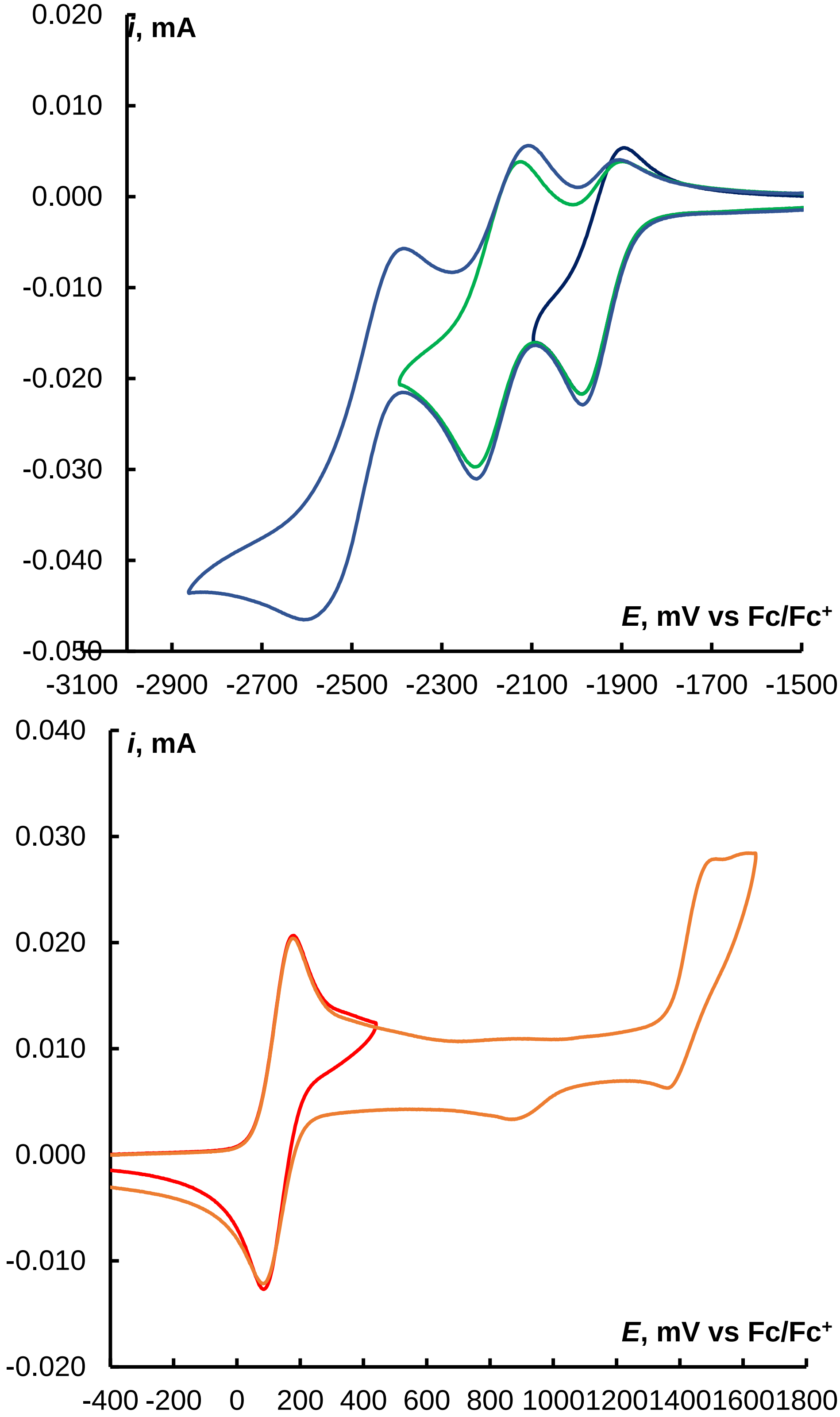

Figure S66. CV curves of **3m**.

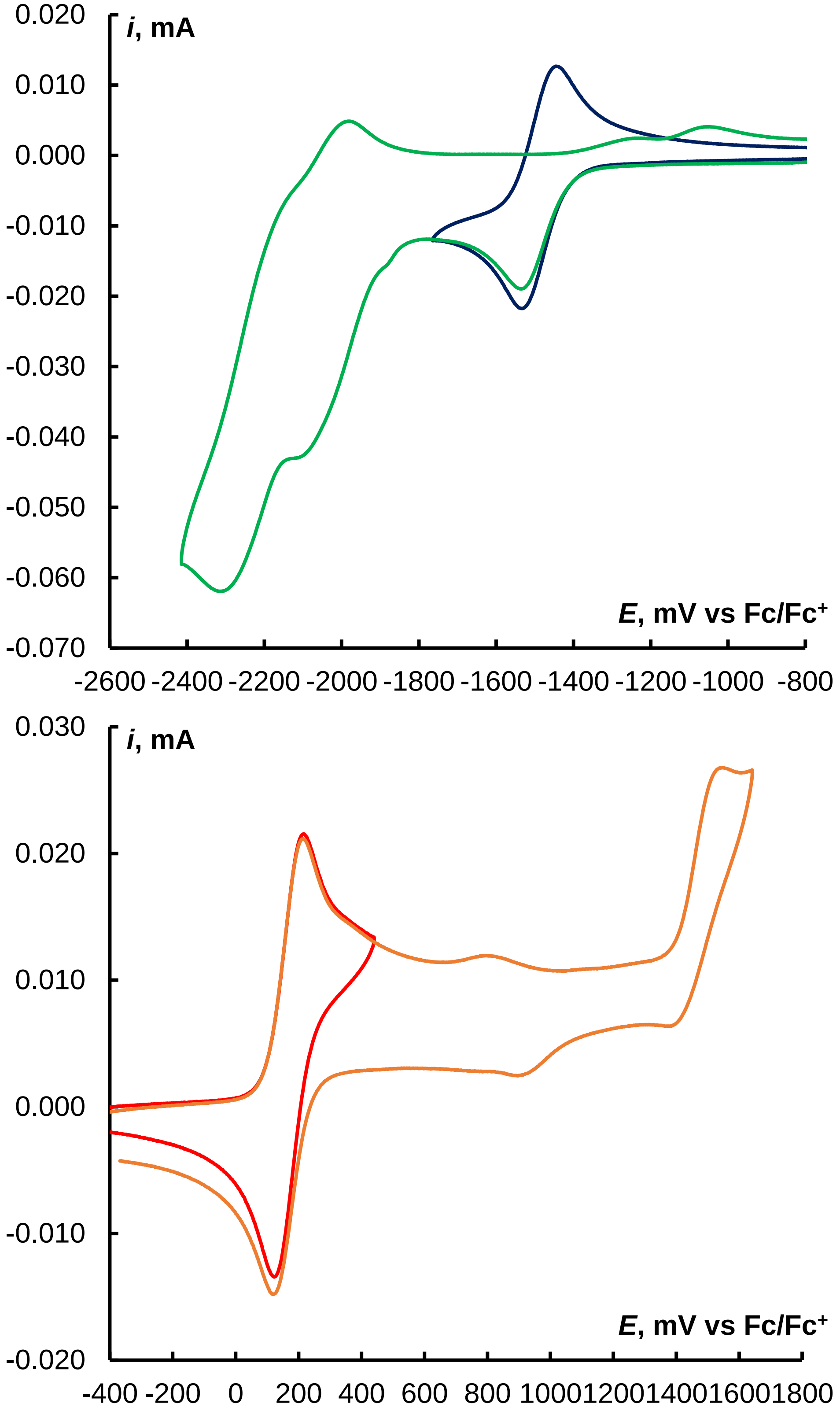

Figure S67. CV curves of **3n**.

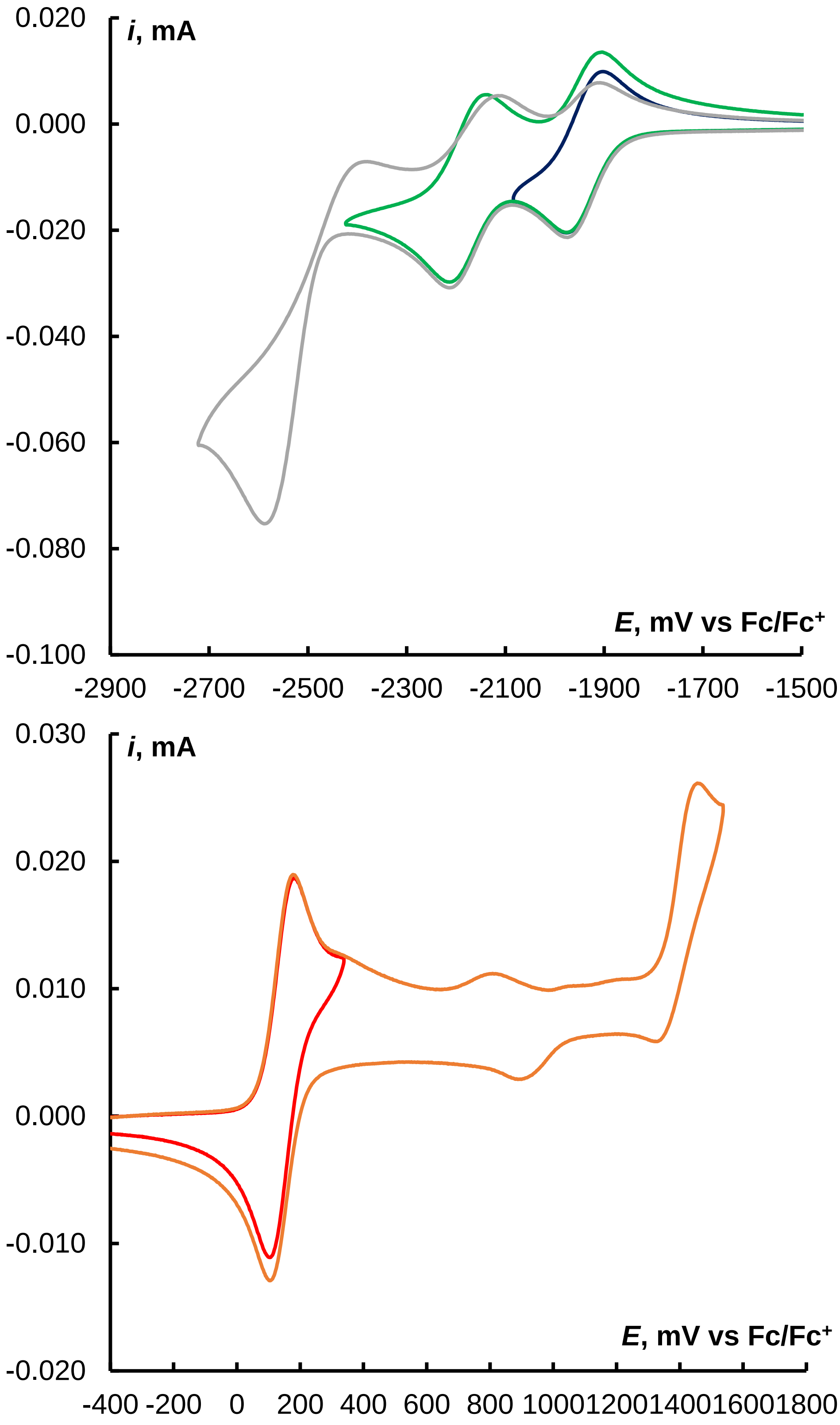

Figure S68. CV curves of **3o**.

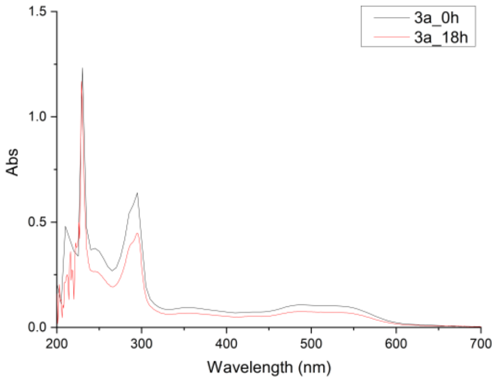
3a_0h
3a_18h
Abs
1.5
1.0
0.5
0.0
200
300
400
500
600
700
Wavelength (nm)

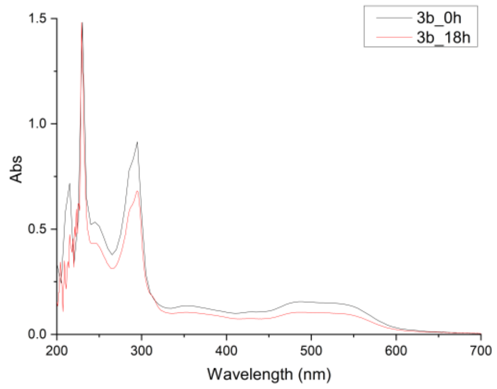
3b_0h
3b_18h
Abs
1.5
1.0
0.5
0.0
200
300
400
500
600
700
Wavelength (nm)

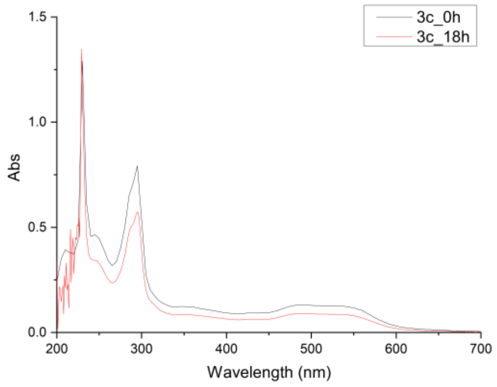
3c_0h
3c_18h
Abs
1.5
1.0
0.5
0.0
200
300
400
500
600
700
Wavelength (nm)

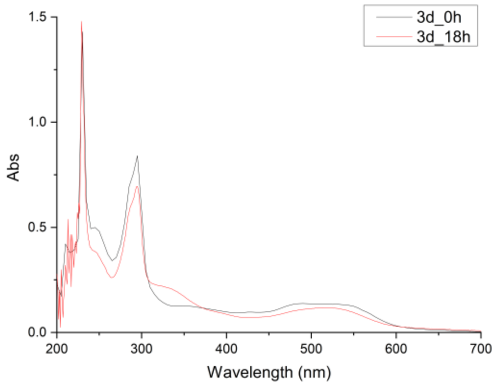
3d_0h
3d_18h
Abs
1.5
1.0
0.5
0.0
200
300
400
500
600
700
Wavelength (nm)

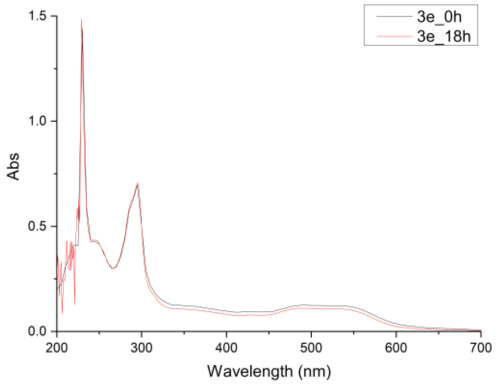
3e_0h
3e_18h
Abs
1.5
1.0
0.5
0.0
200
300
400
500
600
700
Wavelength (nm)

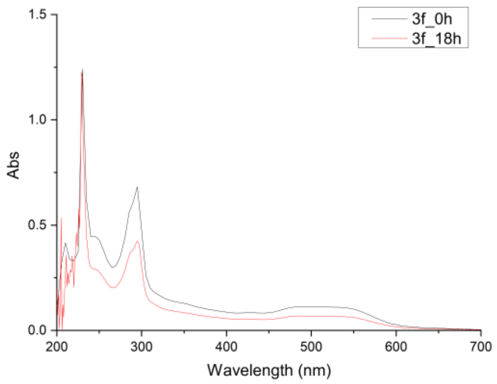
3f_0h
3f_18h
Abs
1.5
1.0
0.5
0.0
200
300
400
500
600
700
Wavelength (nm)

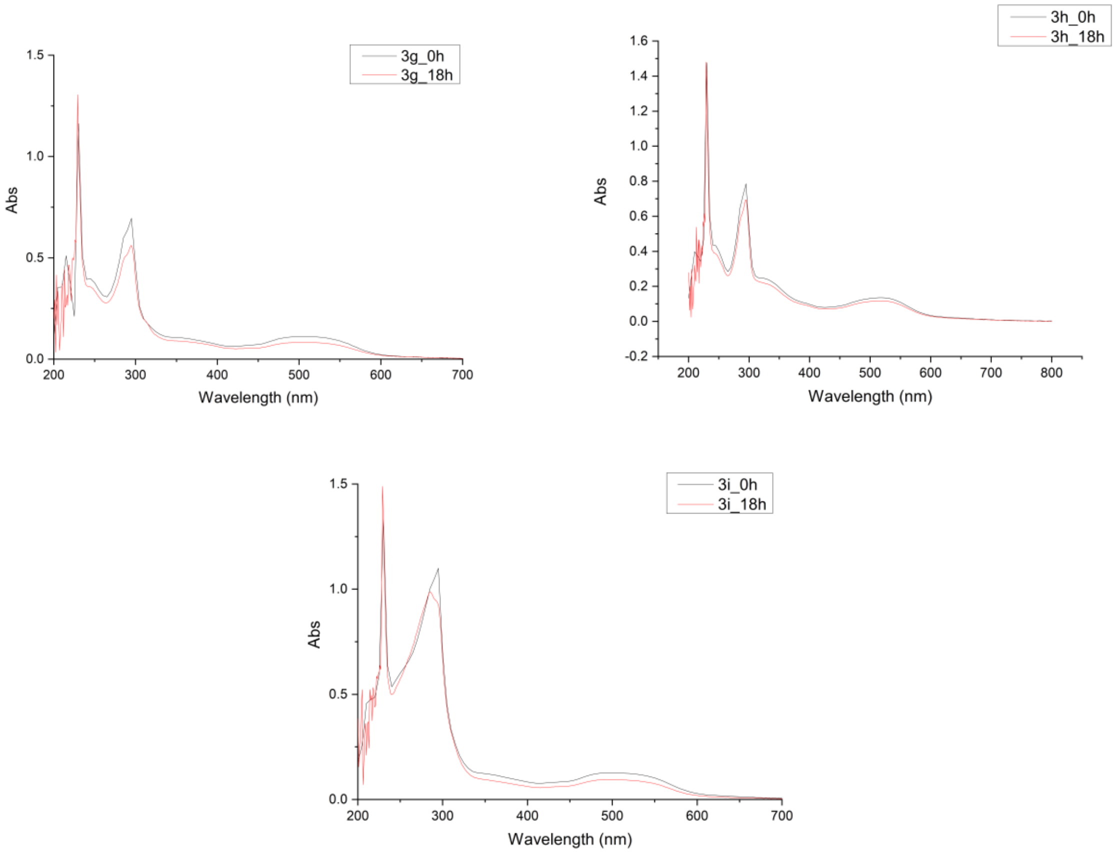

Figure S69. UV-vis spectra of **3a**-**3i** in PBS – 0.5% DMSO solution, concentration 1*10^{-5} M immediately after dissolution and after 18 h at room temperature.

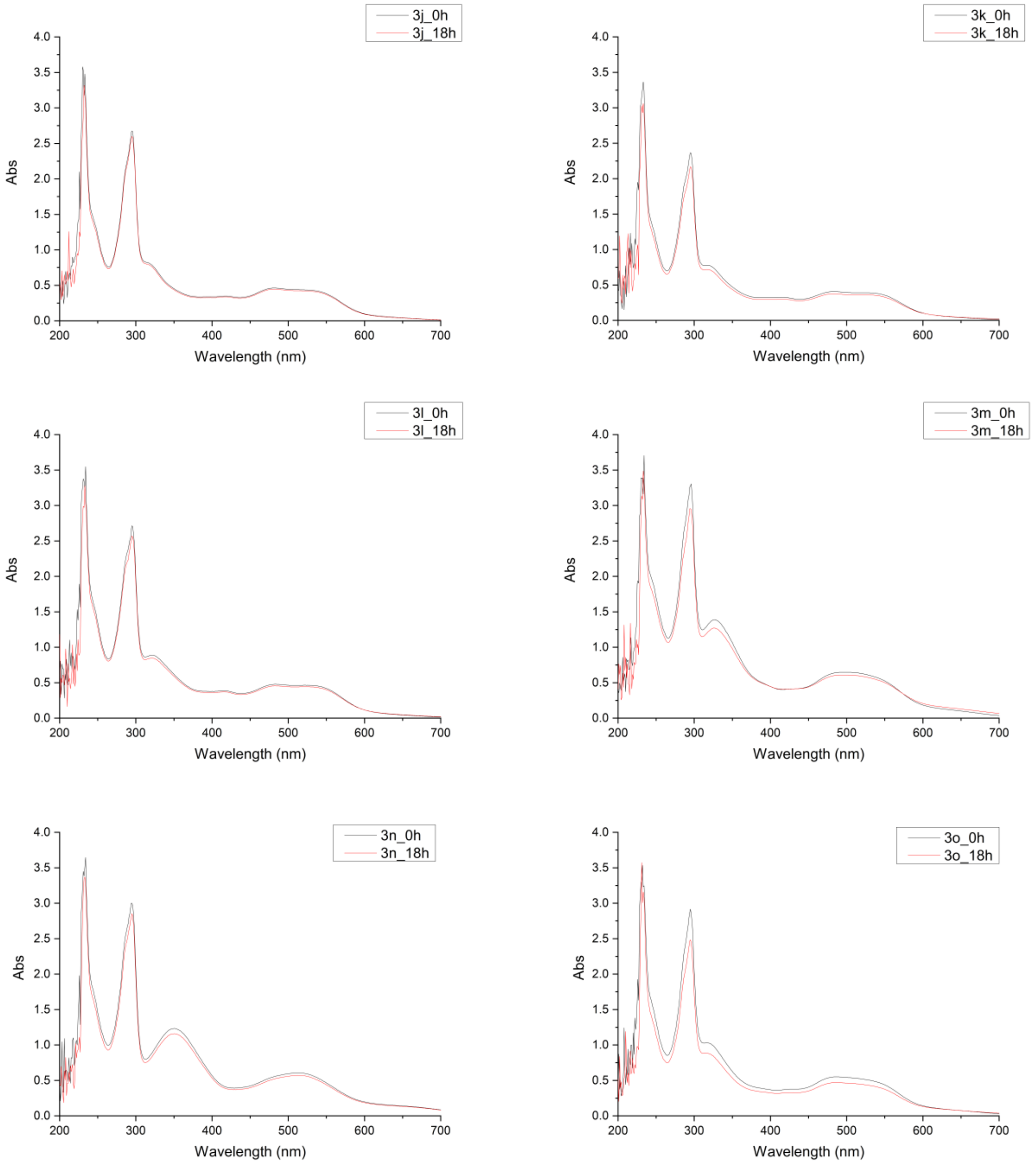

Figure S70. UV-vis spectra of **3j**-**3o** in PBS – 0.5% DMSO solution, concentration 5*10^{-5} M immediately after dissolution and after 18 h at room temperature.
